# Functional and Network PHAs via Stereoselective Polymerization and Tailored Post‐Transformation

**DOI:** 10.1002/anie.9747437

**Published:** 2026-06-30

**Authors:** Ruirui Li, Yingluo Zhao, Jun‐Jie Tian, Ethan C. Quinn, Yunpeng Gao, Maëlle T. Gace, Eugene Y.‐X. Chen

**Affiliations:** ^1^ Department of Chemistry Colorado State University Fort Collins Colorado USA

**Keywords:** crosslinked polymer, functionalized PHA, PHA, post‐functionalization, stereoselective polymerization

## Abstract

Incorporation of functional groups into poly(3‐hydroxyalkanoate)s (PHAs) is an important strategy to tailor their properties for specific applications, but both scopes of functional groups and the methods of transforming them into tailored PHA materials are currently limited and merit further exploration. Here, we report a catalyst‐controlled stereoselective ring‐opening polymerization of functionalized propiolactones for the synthesis of vinyl‐, allyl‐, and propargyl‐functionalized PHAs with high syndiotacticity (*P*_r_ up to 0.95) and a broad glass and melting transition window (*T*_g_ down to −31°C, *T*_m_ up to 126°C). Copolymerization of such lactones with β‐butyrolactone further enhances PHA's thermal robustness and mechanical toughness. Three different methods have been developed to further transform the functionalized PHAs into creep‐ and solvent‐resistant crosslinked PHA thermosets, dynamic‐supramolecular elastomeric PHA networks, and grafted PHAs with hydrophilic and bioactive molecules. PHA functionalization, also uncovers a rare example of PHA supramolecular stereocomplexes via blending an enantiomeric, vinyl‐functionalized PHA pair.

## Graphical abstract

Catalyst‐controlled stereoselective (co)polymerization of functionalized lactones produces vinyl‐, allyl‐, and propargyl‐functionalized PHAs with high syndiotacticity and a broad applicable temperature window, which can be further transformed into crosslinked PHA thermosets, elastic supramolecular networks, and functional grafts.

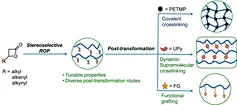

## 1 Introduction

**FIGURE 1:**
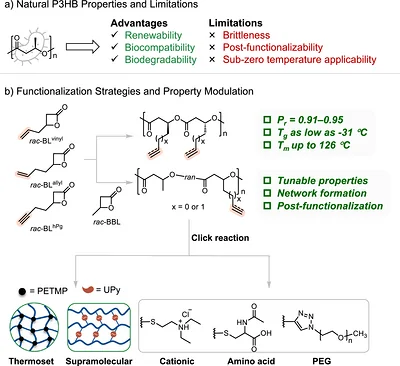
Stereoselective synthesis of functionalized PHAs and their post‐modification.

Poly(3‐hydroxyalkanoate)s (PHAs) have attracted growing interest for applications ranging from biomedical devices and packaging materials to sustainable plastics, often attributed to their renewability, biodegradability, and biocompatibility [1, 2, 3, 4, 5, 6, 7, 8]. Among them, bacterial isotactic (*R*)‐poly(3‐hydroxybutyrate) ((*R*)‐P3HB, the most representative member of the PHA family, exhibits a high melting temperature (*T*_m_) of 170–180 °C and a glass‐transition temperature (*T*_g_) ≈ 0°C), but its high crystallinity, mechanical brittleness, and poor melt processability largely limit practical utility [9, 10, 11, 12]. To overcome these limitations, extensive efforts have focused on stereomicrostructure engineering and copolymerization to modulate P3HB properties. In particular, metal‐catalyzed stereoselective ring‐opening polymerization (ROP) has emerged as a powerful strategy to access P3HB with tunable stereomicrostructures [13, 14, 15, 16, 17, 18] and for incorporating designer comonomers [19, 20, 21, 22, 23], enabling systematic modulation of thermal and mechanical properties of P3HB‐based PHAs. Despite these advances, two fundamental challenges persist. First, most PHAs remain devoid of reactive functional groups, and the hydrophobic P3HB backbone restricts their applicability in biomedical contexts, such as drug delivery, tissue engineering, and stimuli‐responsive materials. Second, although stereo microstructural modification can improve the mechanical performance of P3HB, the *T*_g_ of these materials generally remains above 0 °C, leading to embrittlement at low temperatures and limiting their use in cold‐environment applications (Figure 1a). As packaging materials, PHAs should desirably exhibit polyethylene‐like thermal properties, combining a low *T*_g_ (typically below −20°C) with a high *T*_m_ (above 100 °C), thus maintaining mechanical integrity and thermal stability under typical application temperature windows.

Functionalization of PHAs has been explored as an effective strategy to improve processability and mechanical performance, and to introduce additional functionalities, such as bioactivity, hydrophilicity, and crosslinking capability, which typically relies on the incorporation of specific functional groups, such as olefins [18, 24, 25, 26, 27, 28, 29, 30, 31], azide [32], and amines [33, 34]. In parallel, significant progress has been made in the stereoselective ROP of *racemic* functionalized β‐lactones bearing diverse pendant groups, including alkyl β‐malolactonates (*rac*‐MLA^R^) and 4‐alkoxymethylene‐β‐propiolactones (*rac*‐BPL^CH2OR^], enabling access to isotactic and syndiotactic PHAs with tunable stereo microstructures and thermal properties [18, 35, 36, 37, 38, 39, 40, 41, 42, 43, 44]. Notably, Guillaume and Carpentier reported the synthesis of syndiotactic poly(allyl‐β‐hydroxyalkanoate) poly(BL^allyl^) homopolymer [30] and poly(BL^Me^‐*co*‐BL^allyl^) copolymer [31] from *racemic* allyl‐β‐butyrolactone (*rac*‐BL^allyl^) and *racemic*‐β‐butyrolactone (*rac*‐BBL). The resulting poly(BL^allyl^), with *P*_r_ (probability of *racemic* linkages between monomer units) = 0.80–0.84, exhibited a low *T*_g_ of −44 °C, but remained amorphous due to limited stereoregularity. These studies primarily focused on stereoregulation, thermodynamic properties, and post‐functionalization with simple small molecules (e.g., hydroxylation, borane addition, and epoxidation).

Inspired by these prior works, we have embarked on a study on functionalized PHAs encompassing four key aspects (Figure 1b). First, vinyl‐, allyl‐, and homopropargyl (hPg)‐functionalized PHA homopolymers were synthesized via a well‐controlled yttrium‐catalyzed stereoselective ROP of *rac*‐BL^vinyl^, *rac*‐BL^allyl^, and *rac*‐BL^hPg^, respectively [36, 45, 46]. The resulting polymers display high syndiotacticity (*P*_r_ = 0.91–0.95), favorable thermal properties (*T*_m_ = 98–126 °C and *T*_g_ as low as −31 °C), and good mechanical performance. Second, incorporation of 4–10 mol% functionalized PHA units into the P3HB backbone significantly enhances chain flexibility, leading to markedly improved ductility and toughness. Third, the functionalized PHAs were successfully crosslinked through the thiol–ene click chemistry, affording covalent and dynamic‐supramolecular networks. Finally, post‐polymerization modification with hydrophilic, antimicrobial, and bioactive molecules highlights the potential of these functional PHA materials for biomedical applications.

## 2 Results and Discussion

### 2.1 Synthesis of Syndiotactic and Functionalized PHAs

Functionalized monomers *rac*‐BL^vinyl^, *rac*‐BL^allyl^, and *rac*‐BL^hPg^ were all synthesized on a multi‐gram scale from the corresponding epoxides and carbon monoxide (see Supporting Information for details). As previously established, discrete aminoalkoxy‐bis(phenolate) yttrium‐amido complexes exhibit excellent performance in controlling the stereoselective polymerization of *rac*‐BBL [45]. The tacticity of the resulting P3HB can be tuned by introducing *ortho*‐ and *para*‐substituents with varying steric hindrance on the bisphenolate ligand framework. In this study, *rac*‐BL^vinyl^ was first employed in polymerization studies (Table 1). The initial polymerization was carried out at 23°C in toluene with a monomer/catalyst/initiator ratio of [*rac*‐BL^vinyl^]/[Y]/[I] = 100/1/1 and benzyl alcohol (BnOH) as the initiator (I). Under these conditions, **Y1**, which carries the least sterically hindered *ortho*‐*tert*‐butyl substituents in the series, afforded syndio‐enriched P(BL^vinyl^) with a moderate *P*_r_ of 0.76, a low number‐average molar mass (*M*_n_) of 5.44 kDa, and a narrow dispersity (*Đ*) of 1.03 (Table 1, run 1). Notably, increasing the steric bulk on the bisphenolate ligand enhanced stereo control of polymerization (Table 1, runs 2–4). Specifically, the use of **Y4** bearing *ortho*‐trityl (CPh_3_) substituents resulted in a *P*_r_ value of 0.89 (Table 1, run 4). To produce polymers with high molar mass, the monomer‐to‐initiator ratio was increased to 1000:1:1. However, despite achieving quantitative monomer conversion, the resulting P(BL^vinyl^) still had only a moderate *M*_n_ of 26.9 kDa, but a very low *Đ* of 1.02 (Table 1, run 5), indicative of facile chain transfer to monomer. Subsequent investigations into the effect of the initiator revealed that using *^i^* PrOH led to a modest increase in molar mass (Table 1, runs 6‒7). These results showed **Y4**/*^i^* PrOH to be the optimal catalyst system for the syndioselective polymerization of BL^vinyl^.

**TABLE 1:** Selected results for syndioselective ROP of functionalized β‐lactones monomers.^a^

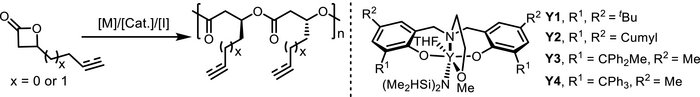										
Runs	[M]/[Cat.]/[I]	[M]	Cat.	[I]	Solvent	Temp. [°C]	Conv. [%]	*M*_n_ ^b^ [kDa]	*Ð* ^b^	*P*_r_ ^c^
1	100:1:1	*rac‐*BL^vinyl^	**Y1**	BnOH	PhMe (2 M)	23	>99	5.44	1.03	0.76
2	100:1:1	*rac‐*BL^vinyl^	**Y2**	BnOH	PhMe (2 M)	23	>99	7.76	1.03	0.80
3	100:1:1	*rac‐*BL^vinyl^	**Y3**	BnOH	PhMe (2 M)	23	>99	12.2	1.05	0.83
4	100:1:1	*rac‐*BL^vinyl^	**Y4**	BnOH	PhMe (2 M)	23	>99	9.53	1.02	0.89
5^d^, ^e^	1000:1:1	*rac‐*BL^vinyl^	**Y4**	BnOH	DCM (2 M)	40	>99	26.9	1.02	0.91
6^d^, ^e^	1000:1:1	*rac‐*BL^vinyl^	**Y4**	*^i^* PrOH	DCM (2 M)	40	>99	34.0	1.02	0.91
7^d^, ^f^	2000:1:1	*rac‐*BL^vinyl^	**Y4**	*^i^* PrOH	PhMe (2 M)	40	>99	42.6	1.02	0.90
8^d^, ^g^	1000:1:1	*rac‐*BL^allyl^	**Y4**	*^i^* PrOH	PhMe (2 M)	40	>99	65.1	1.03	0.93
9^d^, ^g^	2000:1:1	*rac‐*BL^allyl^	**Y4**	*^i^* PrOH	PhMe (2 M)	40	98	245	1.23	0.95
10^d^, ^h^	1000:1:1	*rac‐*BL^hPg^	**Y4**	*^i^* PrOH	PhMe (2 M)	40	98	70.5	1.23	0.91
11^d^, ^h^	2000:1:1	*rac‐*BL^hPg^	**Y4**	*^i^* PrOH	PhMe (3 M)	40	83	72.9	1.03	0.90

^a^ Monomer conversion (Conv.) determined by ^1^H NMR in CDCl_3_, *rac*‐BL^vinyl^, 0.112 g, 1 mmol, polymerization time = 12 h.

^b^ Weight‐average (*M*_w_) and number‐average (*M*_n_) molar mass and dispersity (*Ð* = *M*_w_ */M*_n_) determined via size exclusion chromatography (SEC) at 40 °C in CHCl_3_ coupled with a Wyatt DAWN HELEOS II multi (18)‐angle light scattering detector and a Wyatt Optilab TrEX dRI detector for absolute molar mass.

^c^ *P*_r_ probability of *racemic* linkages between BL^vinyl^ units.

^d^ Polymerization time, 24 h.

^e^ *rac*‐BL^vinyl^, 0.560 g, 5 mmol.

^f^ *rac*‐BL^vinyl^, 0.840 g, 7.5 mmol.

^g^ *rac*‐BL^allyl^, 0.630 g, 5 mmol.

^h^ *rac*‐BL^hPg^, 1.24 g, 10 mmol.

Next, we investigated performance of **Y4**/*^i^* PrOH in the stereoselective polymerization of *rac*‐BL^allyl^, obtaining P(BL^allyl^) a syndiotacticity (*P*_r_ = 0.93‒0.95, Table 1, runs 8‒9, and Table S3). At a [*rac*‐BL^allyl^]/[Y]/[I] ratio of 2000:1:1, the polymerization reached 98% monomer conversion, affording P(BL^allyl^) with high molar mass (*M*_n_ = 245 kDa, *Đ* = 1.23). Similarly, propargyl‐functionalized polymer P(BL^hPg^) was also synthesized with a high degree of stereo control (*P*_r_ = 0.90–0.93, Table 1, runs 10‒11, and Table S4). However, at the same 2000:1:1 ratio, the monomer conversion decreased to 83%, and the resulting polymer had a lower molar mass with *M*_n_ = 72.9 kDa, *Đ* = 1.03, highlighting the challenges for polymerizing lactone monomers functionalized with acidic terminal C─H bonds (i.e., terminal alkynes) into high‐molar‐mass PHAs.

### 2.2 Material Properties of Functionalized PHAs

**FIGURE 2:**
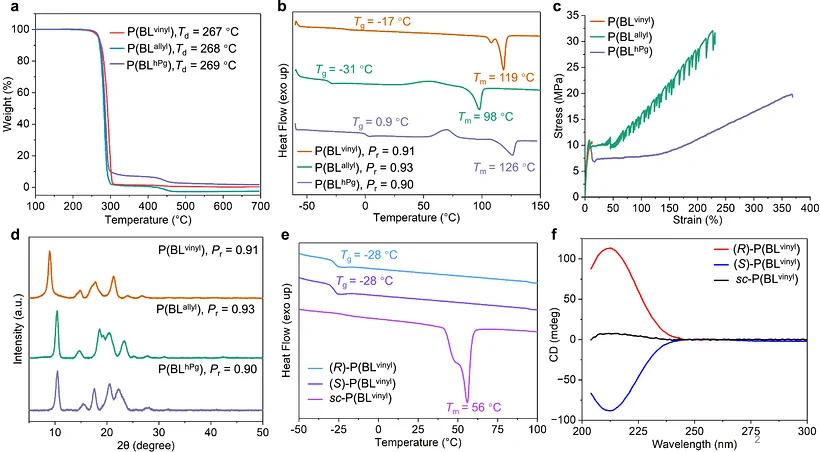
Characterization of vinyl‐, allyl‐, and propargyl‐functionalized PHA homopolymers. (a) Thermal gravimetric analysis of P(BL^vinyl^) (*P*_r_ = 0.90), P(BL^allyl^) (*P*_r_ = 0.93), and P(BL^hPg^) (*P*_r_ = 0.90). (b) Overlayed DSC curves of P(BL^vinyl^), P(BL^allyl^), and P(BL^hPg^). Scan rate: 10°C/min. (c) Representative stress‐strain curves of P(BL^vinyl^) (*M*_n_ = 42.6, *Đ* = 1.02), P(BL^allyl^) (*M*_n_ = 245, *Đ* = 1.23), and P(BL^hPg^) (*M*_n_ = 72.9, *Đ* = 1.03). (d) pXRD profiles of P(BL^vinyl^), P(BL^allyl^) and P(BL^hPg^), showing characteristic diffraction peaks in the range of 2θ ≈ 9–23°. (e) Overlayed DSC curves (first heating) of (*R*)‐P(BL^vinyl^), (*S*)‐P(BL^vinyl^), and *sc*‐P(BL^vinyl^). (f) Overlayed CD spectra of (*R*)‐P(BL^vinyl^), (*S*)‐P(BL^vinyl^), and *sc*‐P(BL^vinyl^).

Thermogravimetric analysis (TGA) thermograms showed that, the three (vinyl, allyl, and hPg) functionalized PHAs exhibited a decomposition temperature (*T*_d_, the decomposition temperature at 5% weight loss) of 267, 268, and 269°C, respectively (Figure 2a). Differential scanning calorimetry (DSC) analysis showed that, the syndio‐enriched P(BL^vinyl^) with *P*_r_ = 0.76 exhibited a *T*_g_ of −26 °C and *T*_m_ of 86 °C (Figure S53). As expected, *T*_m_ increased with the increase of the syndiotacticity of P(BL^vinyl^) (Figure S59). Notably, P(BL^vinyl^) with *P*_r_ = 0.91 displayed a *T*_m_ of 119 °C (Figure 2b). The *T*_g_ also increased slightly with increasing *P*_r_, from −26 °C (*P*_r_ = 0.76) to −17 °C (*P*_r_ = 0.91), suggesting that higher crystallinity restricts molecular chain mobility, thereby enhancing *T*_g_. When comparing across functional group (vinyl, allyl, and hPg) types, syndiotactic P(BL^allyl^) with *P*_r_ = 0.93 exhibited a lower *T*_g_ (−31 °C) and *T*_m_ (98 °C) than P(BL^vinyl^) (*T*_g_ = −17 °C, *T*_m_ = 119 °C). In contrast, P(BL^hPg^) (*P*_r_ = 0.91) showed higher thermal transitions, with a *T*_g_ close to 0 °C and a *T*_m_ of 126 °C, exceeding those of the vinyl and allyl counterparts (Figure 2b). The crystallization of P(BL^allyl^) and P(BL^hPg^) appears to be relatively slow, as evidenced by the presence of crystallization exotherms only during the second heating scan at 56 and 70 °C, respectively, suggesting a melting‐crystallization behavior rather than crystallization during the cooling process. In contrast, all atactic analogues P(BL^vinyl^), P(BL^allyl^), and P(BL^hPg^) are amorphous materials at room temperature, exhibiting only glass transitions without any detectable melting transitions. Their *T*_g_ values are −34, −44, and −1° C, respectively (Figures S50−S52). Overall, these differences highlight the notable influence of side‐chain length, flexibility, and rigidity on the thermal properties and crystallization behavior of these functionalized PHAs.

We further tested the mechanical properties of the obtained functionalized PHAs (Figure 2c). P(BL^vinyl^) (*M*_n_ = 42.6 kDa) was brittle, exhibiting a low elongation at break (*ε*_B_ = 10.6 ± 2.8%). In contrast, P(BL^hPg^) (*M*_n_ = 72.9 kDa) exhibited high ductility (*ε*_B_ = 345.7 ± 33.5%), coupled with an elastic modulus (*E*) of 293 ± 12.5 MPa, a yield strength (*σ*_y_) of = 10.0 ± 0.3 MPa, and an ultimate strength (*σ*_B_) of 19.3 ± 1.9 MPa. Similarly, P(BL^allyl^) (*M*_n_ = 245 kDa) also displayed a ductile behavior (*ε*_B_ = 244.2 ± 23.6%) with comparable *E* (260 ± 6.7 MPa) and *σ*_y_ (10.9 ± 0.2 MPa), but a twofold increase in ultimate strength (*σ*_B_ = 32.7 ± 1.1 MPa). Notably, the tensile stress–strain curves of P(BL^allyl^) displayed distinct periodic oscillations during neck propagation, a behavior referred to as the stress‐oscillation (SO) phenomenon, which is governed by factors including stretching temperature, strain rate, and semicrystalline morphology of the material [47, 48, 49, 50].

The powder x‐ray diffraction (pXRD) patterns were investigated to assess the crystalline nature of the functionalized PHAs (Figure 2d). The pXRD profile of P(BL^vinyl^) exhibited major diffraction peaks at 2θ ≈ 9.0°, 14.8°, 17.8°, and 21.3°. Similarly, P(BL^allyl^) showed prominent peaks at 2θ ≈ 10.4°, 14.7°, 18.6°, 20.4°, and 23.3°, while P(BL^hPg^) featured major peaks at 10.5°, 15.4°, 17.6°, 20.6°, and 22.3°. These diffraction patterns confirm that, all three functionalized PHAs are semi‐crystalline, with differences in peak positions and intensities likely arising from variations in side‐chain structure and tacticity.

Physical blending of enantiomeric chiral polyesters in a stoichiometric ratio is a well‐established strategy for constructing highly crystalline stereocomplex (*sc*) materials, which often exhibit higher *T*_m_ values and faster crystallization rates compared to their individual enantiomeric counterparts [51, 52, 53, 54, 55]. Worth noting here is that stereocomplexation in PHAs is rare; for example, (*R*)‐ and (*S*)‐P3HB do not form a *sc* [12]. To examine, if such a *sc* could be formed in the current PHAs, chiral (*R*)‐ and (*S*)‐BL^vinyl^ monomers were prepared for accessing enantiomeric isotactic P(BL^vinyl^) materials. Interestingly, the resulting isotactic PHAs, either (*R*)‐ or (*S*)‐P(BL^vinyl^), are amorphous, exhibiting only a *T*_g_ (−28 °C) and appearing as a viscous liquid at room temperature (Table S2 and Figures S60, S61). However, physical blending of (*R*)‐ and (*S*)‐P(BL^vinyl^) in a 1:1 ratio resulted in the formation of a semi crystalline material with *T*_m_ = 56 °C, an associated enthalpy of fusion (*ΔH*_f_) = 43 J/g (first heating scan) and *T*_g_ = −26 °C (Figures 2e and S62), suggesting formation of a stereocomplex, *sc*‐P(BL^vinyl^). Additionally, circular dichroism (CD) analysis revealed strong positive and negative Cotton effects near 220 nm for (*R*)‐P(BL^vinyl^) and (*S*)‐P(BL^vinyl^), respectively, whereas *sc*‐P(BL^vinyl^) displayed a nearly flat CD profile, characteristic of a stereocomplex (Figure 2f). Furthermore, pXRD analysis revealed that *sc*‐P(BL^vinyl^) exhibited distinct diffraction peaks at 2θ ≈ 10.3°, 19.2°, 21.7°, and 22.7°, along with several weaker reflections, indicative of the formation of a crystalline stereocomplex structure (Figure S93).

### 2.3 Functionalization of P3HB via Copolymerization

**FIGURE 3:**
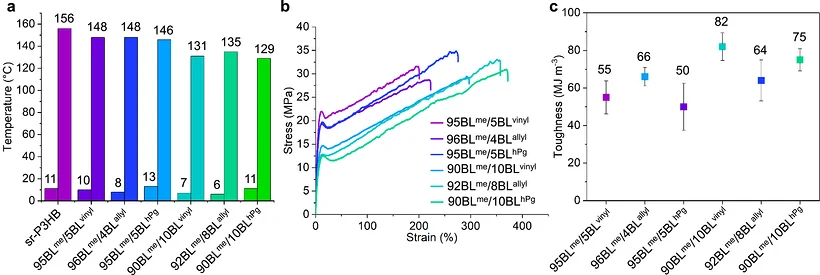
Thermal and mechanical properties of functionalized P3HB copolymers. Copolymers are simplified as xBL^Me^/yBL^vinyl^, xBL^Me^/yBL^allyl^, or xBL^Me^/yBL^hPg^ in the figures, corresponding to P(BL^Me^_x_‐*co*‐BL^vinyl^_y_), P(BL^Me^_x_‐*co*‐BL^allyl^_y_), and P(BL^Me^_x_‐*co*‐BL^hPg^_y_), respectively. (a) *T*_g_ (shorter columns) and *T*_m_ (taller columns) of copolymers with varying compositions. (b) Representative stress‐strain curves. (c) Calculated toughness of functionalized copolymers.

Introducing functional groups to P3HB can modify its thermal and mechanical properties, thus expanding its applications in various fields. In addition, installation of the functional groups provides opportunities for further post‐modifications of the resulting PHA copolymers (e.g., via crosslinking, grafting, etc.). With functionalized β‐propiolactones in hand, we synthesized olefin‐ and alkyne‐functionalized P3HB copolymers through the ring‐opening copolymerization of *rac*‐BBL with functionalized β‐lactone derivatives using **Y2** (Tables S6‒S8). The incorporation of functional groups ranged from 4% to 10%, as confirmed by ^1^H NMR analysis. The P3HB copolymers incorporating vinyl and allyl groups were achieved with high molar mass (*M*_n_ = 159‒202 kDa), whereas the propargyl‐functionalized copolymers were obtained with lower, but still appreciable molar mass (*M*_n_ = 79.8‒90.5 kDa). Analysis by TGA revealed that *T*_d_ values ranged from 265 to 273°C, showing minimal deviation from that of the syndio‐rich P3HB (*P*_r_ = 0.84), *sr*‐P3HB, (*T*_d,5%_ = 265°C) prepared with **Y2** (Figures S37‒S42). This result indicates that the incorporation of such functional groups (vinyl, allyl, and hPg) did not compromise the thermal stability of the PHA copolymers. While *sr*‐P3HB exhibited a *T*_m_ of 156°C and a *T*_g_ of 11 °C, incorporation of 5% or 10% vinyl groups experienced a reduced *T*_m_ to 148 or 131 °C and a similar *T*_g_ of 10 or 7 °C, respectively (Figure 3a). Similarly, P3HB functionalized with allyl and hPg groups exhibited similar trends, where thermal properties were tunable by varying the type and concentration of functional groups. Specifically, for allyl‐functionalized copolymers, P(BL^me^_96_‐*co*‐BL^allyl^_4_) and P(BL^me^_92_‐*co*‐BL^allyl^_8_) exhibited *T*_m_ = 148 and 135° C and *T*_g_ = 8 and 6 °C, respectively. For hPg‐functionalized copolymers, P(BL^me^_95_‐*co*‐BL^hPg^_5_) and P(BL^me^_10_‐*co*‐BL^hPg^_10_) showed *T*_m_ = 146 and 129 °C and *T*_g_ = 13 and 11 °C, respectively. These results demonstrate that increasing the incorporation of functional groups progressively lowers the crystallinity and thus *T*_m_ of the P3HB copolymers, but there is no significant modulation on the *T*_g_ values, which remained in a narrow range (6 to 13 °C).

All P3HB copolymers exhibited ductile behavior with *ε*_B_ between 200% and 400% (Figure 3b). The *σ*_B_ value was high and ranged from 29.8 to 33.4 MPa, depending on the type and content of the incorporated functional groups. The *ε*_B_ value increased with a higher amount of functional group incorporation; for example, P(BL^me^_95_‐*co*‐BL^vinyl^_5_) exhibited *ε*_B_ = 214.5 ± 30.3%, while P(BL^me^_90_‐*co*‐BL^vinyl^_10_) showed a significantly higher *ε*_B_ = 300.0 ± 17.0%. Meanwhile, the *σ*_y_ and *E* decreased as the percentage of functional groups increased. Specifically, P(BL^me^_95_‐*co*‐BL^vinyl^_5_) gave a *σ*_y_ of 21.8 ± 0.5 MPa and *E* of 633 ± 21.0 MPa, which decreased to *σ*_y_ = 14.7 ± 0.4 MPa and *E* = 109 ± 6.1 MPa with 10% vinyl group incorporation. On the other hand, the toughness (*U*_T_) improved with increasing functional group incorporation. For instance, P(BL^me^_90_‐*co*‐BL^vinyl^_10_) had a toughness of 66 ± 4.9 MJ·mˉ^3^, compared to 55 ± 8.8 MJ·mˉ^3^ for P(BL^me^_95_‐*co*‐BL^vinyl^_5_). Similarly, P(BL^me^_90_‐*co*‐BL^hPg^_10_) showed an enhanced toughness to 75 ± 5.9 MJ·mˉ^3^, higher than 64 ± 10.9 MJ·mˉ^3^ for P(BL^me^_95_‐*co*‐BL^hPg^_5_). Notably, the P3HB copolymer with 8% allyl group incorporation, P(BL^me^_92_‐*co*‐BL^allyl^_8_), demonstrated the highest toughness among all PHA copolymers (*U*_T_ = 82 ± 7.4 MJ·mˉ^3^), underscoring the impact of functional group content on mechanical performance (Figure 3c).

Overall, these results showed that the introduction of functional groups enhances molecular chain flexibility, reduces crystallinity, and substantially improves the ductility and toughness of the P3HB materials. However, such mechanical enhancements appear to be applicable only to syndio‐rich P3HB, but not to more crystalline, syndiotactic P3HB. This observation was revealed by the synthesized copolymer P(BL^Me^_92_‐*co*‐BL^allyl^_8_) with higher syndiotacticity and crystallinity using **Y4**, which produced syndiotactic P3HB with *P*_r_ = 0.91 and *T*_m_ = 184 °C. The syndiotactic P3HB copolymer exhibited a *T*_m_ of 160 °C, but showed a brittle behavior, just like the unfunctionalized syndiotactic P3HB (Figures S77 and S103).

### 2.4 Crosslinking to Network PHAs

**FIGURE 4:**
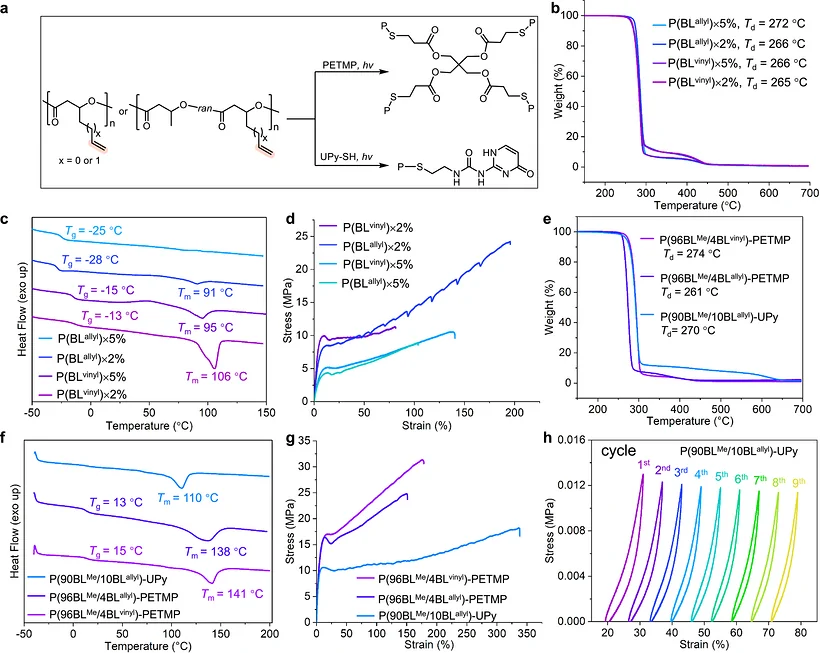
Characterization of crosslinked PHAs. (a) Diagram of crosslinking reactions. (b) TGA of crosslinked homopolymers. (c) Overlayed DSC curves of crosslinked homopolymer. Scan rate: 10 °C/min (d) Representative stress‐strain curves of crosslinked homopolymer. (e) TGA of crosslinked copolymer. (f) Overlayed DSC curves of crosslinked copolymer. P(90BL^me^/10BL^allyl^)‐UPy (first heating scan). Scan rate: 10 °C/min. (g) Representative stress‐strain curves of crosslinked PHAs. (h) Hysteresis tensile curves for P(90BL^me^/10BL^allyl^)‐UPy, strain rate = 5 mm/min, ambient condition.

The incorporation of functional groups (vinyl, allyl, or hPg) into the PHA backbone provides reactive sites for crosslinking (e.g., by the thiol‐ene reaction), leading to crosslinked PHA networks for enhanced dimensional stability, solvent resistance, and mechanical integrity under stress. The crosslinking reaction was carried out with pentaerythritol tetra(3‐mercaptopropionate) (PETMP) as the thiol‐based crosslinker via the thiol‐ene reaction, enabling the formation of a three‐dimensional polymer network (Figure 4a). Successful crosslinking was evidenced by the insolubility of the resulting PHA network in a range of common organic solvents (acetone, dichloromethane, ethyl acetate, tetrahydrofuran, toluene, and *N*,*N*‐dimethylformamide) over a period of one year at room temperature (Figure S114), the high gel fractions (75.0%–89.9%) obtained after refluxing in selected solvents at 50°C or 80°C for 48 h (Table S11) and the decreased intensity for the alkene absorption at 1641 cmˉ^1^ characterized by FT‐IR spectroscopy (Figures S115‒S118). In a non‐covalent crosslinking approach to a network PHA, we incorporated supramolecular dynamic bonds [56, 57] into PHAs. Such strategies typically enhance the mechanical performance of polymers and may also impart unique functional properties [58]. Among the various supramolecular functional groups explored to date, ureidopyrimidinone (UPy) is particularly attractive owing to its exceptionally strong and reversible quadruple hydrogen‐bonding interactions and robust self‐association capability [59, 60]. Utilizing these features, we installed UPy motifs into P(BL^me^_90_‐*co*‐BL^allyl^_10_) copolymer (Figure 4a).

TGA analysis revealed the *T*_d_ ranging from 265‒272 °C for the crosslinked PHA networks, which did not differ much from that of the corresponding uncrosslinked PHAs (Figure 2a *vs* 4b). DSC analysis revealed that *T*_m_ decreased with increasing the degree of crosslinking, while *T*_g_ showed the opposite trend. For instance, the *T*_m_ of P(BL^vinyl^) decreased from 119 to 106°C and 95°C for the samples with 2% and 5% crosslinking, respectively (Figure 4c). A similar decreasing trend in *T*_m_ was also observed for P(BL^allyl^) as the crosslinking density increased, with P(BL^allyl^)×5% exhibiting no detectable melting transition in the second heating scan of DSC, due to interruption of crystallization by crosslinking.

Mechanical properties of the crosslinked PHAs were also investigated. Upon crosslinking, P(BL^vinyl^) became more ductile compared to its uncrosslinked counterpart (Figure 4d). However, both the yield stress and ultimate tensile strength decreased as the degree of crosslinking increased, due to reduced crystallinity. A similar trend was observed for crosslinked P(BL^allyl^), but the difference was that P(BL^allyl^)×2% exhibited a higher *ε*_B_ = 198.2 ± 24.7% than P(BL^allyl^)×5% (*ε*_B_ = 108.1 ± 13.7%), because of excessive crosslinking that restricts chain mobility. Additionally, the tensile curves of the crosslinked P(BL^allyl^) became smoother and showed less oscillation compared to the uncrosslinked P(BL^allyl^), presumably due to the decrease in crystallinity.

For crosslinked P3HB copolymers, TGA showed a little effect of crosslinking on the *T*_d_ of the copolymers (Figure 4e). Effects of crosslinking of the copolymers on *T*_m_ and *T*_g_ (Figure 4f) also followed the same trend as observed for the PHA homopolymers. Mechanical tests revealed that P(BL^me^_96_‐*co*‐BL^vinyl^_4_)‐PETMP exhibited appreciable ductility with *ε*_B_ = 165.4 ± 35.7%, high ultimate tensile strength with *σ*_B_ = 31.1 ± 4.7 MPa, and a high toughness with *U*_T_ = 38.3 ± 12.0 MJ·m^−3^ (Figure 4g). In comparison, P(BL^me^_96_‐*co*‐BL^allyl^_4_)‐PETMP exhibited a lower toughness with *U*_T_ = 24.9 ± 13.7 MJ·m^−3^, ultimate strength (*σ*_B_ = 26.2 ± 2.1 MPa). Frequency‐dependent rheological measurements of the PETMP‐crosslinked copolymers revealed a nearly constant storage modulus at 180°C, indicating a stable and predominantly elastic network. The loss modulus increased slightly with frequency, while the complex viscosity decreased markedly, demonstrating pronounced shear‐thinning behavior (Figures S119, S120). Both PHA networks exhibited comparable viscoelastic responses. Creep experiments at 180 °C showed a creep strain below 1.5% (Figures S121, S122), indicating excellent resistance to deformation under thermal and mechanical stress. These results confirmed the formation of a robust yet processable viscoelastic network.

Non‐covalently crosslinked P(BL^me^_90_‐*co*‐BL^allyl^_10_)‐UPy showed good ductility (*ε*_B_ = 319.5 ± 148.6%) with a markedly enhanced tensile strength (*σ*_B_ = 14.7 ± 4.9 MPa, Figure 4g) compared with the corresponding non‐functionalized copolymer (*σ*_B_ = 3.6 ± 0.4 MPa, Figure S112). Notably, hysteresis tensile experiments showed reversible stress–strain behavior (Figure 4h), indicating that P(BL^me^_90_‐*co*‐BL^allyl^_10_)‐UPy exhibits elastomeric characteristics. Collectively, these results highlight UPy‐imparted supramolecular interactions as an effective strategy to endow P3HB‐based PHAs with enhanced mechanical strength and elastomeric performance by forming a non‐covalent, dynamic, reversible network.

### 2.5 Post‐Transformation to Functional PHAs

**SCHEME 1:**
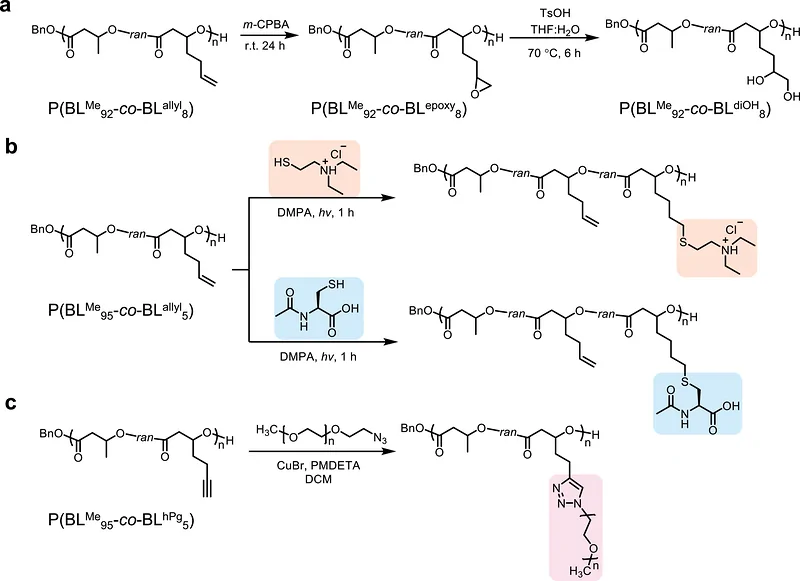
Outlined three methods for post‐transformation of functionalized P3HB‐based copolymer into functional PHAs.

Thanks to its biocompatibility, P3HB has attracted considerable research interest for potential biomedical applications. However, its inherent hydrophobicity poses significant limitations, particularly in areas, such as cell compatibility and drug delivery. Therefore, introducing the alkene and alkyne groups to P3HB will provide opportunities for post‐modification of the resulting P3HB copolymers. To preliminarily demonstrate, such potentials, we oxidized the alkene groups in allyl‐functionalized P3HB copolymer into epoxide groups, which were subsequently hydrolyzed to hydroxyl groups (Figure S19), thereby increasing the hydrophilicity of the PHA (Scheme 1a) [61]. DSC analysis revealed that while the *T*_m_ remained largely unchanged, the crystallization rate decreased (Figure S89), suggesting disrupted chain packing. In addition, we synthesized functionalized atactic copolymers (Table S9) and successfully reacted them with 2‐diethylaminoethanethiol hydrochloride via the thiol‐ene click reaction to form cationic PHAs (Scheme 1b, Figure S20), introducing quaternary ammonium moieties structurally analogous to those reported for gene delivery [62] and antimicrobial [63, 64] applications. Furthermore, we incorporated amino acid molecules into the allyl‐functionalized P3HB copolymer (Scheme 1b, Figure S21), yielding pendant functional groups with structural features relevant to drug carrier applications. Lastly, the hPg‐functionalized PHA was conjugated with azido‐terminated polyethylene glycol (azido‐PEG) via the CuAAC click chemistry (Scheme 1c, Figure S22), introducing PEG chains known to improve hydrophilicity and reduce nonspecific protein adsorption [65]. Taken together, these post‐functionalization strategies demonstrate the chemical versatility of the functional PHA platform and highlight its potential for biomedical applications.

## 3 Conclusion

This work establishes a synthetic framework to access diversely functionalized PHAs, which are post‐transformed into robust covalently crosslinked and elastomeric supramolecular PHA network materials. The catalyst‐controlled stereoselective polymerization of functionalized propiolactones readily leads to a series of vinyl‐, allyl‐, and propargyl‐functionalized PHAs with a varied degree of syndio tacticity up to *P*_r_ = 0.95, a high molar mass up to *M*_n_ = 245 kDa, a broad thermal transition window of *T*_g_ down to −31 °C and *T*_m_ up to 126°C, and diverse mechanical performance of ultimate strength up to *σ*_B_ = 32.7 MPa and ductility of *ε*_B_ > 240%. Accessing enantiomeric, isotactic vinyl‐functionalized PHAs uncovers a rare example of PHA stereocomplexes: Whereas, either (*R*)‐ or (*S*)‐P(BL^vinyl^) is an amorphous, viscous liquid, their 1:1 mixture forms a crystalline stereocomplex. Functionalization of P3HB can be efficiently achieved by copolymerization of functionalized propiolactones with β‐butyrolactone, further, enhancing PHA's thermal and mechanical properties with toughness up to *U*_T_ = 82 ± 7.4 MJ·mˉ^3^. Post‐transformation of the functionalized PHA homopolymers and copolymers utilizes three different approaches, including covalent crosslinking, supramolecular hydrogen‐bonding, and small‐molecule grafting, which created three types of network or functional PHA materials: Molecularly engineered thermally robust, solvent‐, and creep‐resistant crosslinked PHA thermosets; reversible, elastic PHA network materials, and hydrophilic, or bioactive PHA materials.

## Author Contributions

**Ruirui Li**: conceptualization, investigation, writing – original draft, methodology, validation, visualization, data curation. **Yingluo Zhao**: investigation, methodology, validation, data curation. **Jun‐Jie Tian**: investigation and data curation. **Ethan C. Quinn**: investigation and data curation. **Yunpeng Gao**: investigation and data curation. **Maëlle T. Gace**: investigation and data curation. **Eugene Y.‐X. Chen**: conceptualization, funding acquisition, writing – review and editing, supervision, resources, project administration.

## Conflicts of Interest

The authors declare no conflicts of interest.

## Supporting information




**Supporting File**: The authors have cited additional references within the Supporting Information.

## Data Availability

The data that supports the findings of this study are available in the supplementary material of this article.
