# Supplementary material for: Functional and Network PHAs via Stereoselective Polymerization and Tailored Post‐Transformation
Source: Angew Chem Int Ed Engl. 2026 Jun 30;65(36):e9747437. doi: 10.1002/anie.9747437 (PMC13528502; doi:10.1002/anie.9747437)
Supplement: Supplementary file 1 [file ANIE-65-e9747437-s001.pdf]

Supporting Information

Functional and Network PHAs via Stereoselective Polymerization and Tailored Post-transformation

*R. Li, Y. Zhao, J.-J. Tian, E. C. Quinn, Y, Gao, M. T. Gace, and E. Y.-X. Chen**

Table of Contents

Materials and General Methods 3

General Polymerization Procedures 3

General Crosslinking Procedures 4

Post-Functionalization of Copolymers 5

Spectroscopic Characterizations 6

Thermal Analysis 7

Mechanical Analysis 7

Monomer Synthesis 8

Stereocomplexation Procedure 11

Supplementary Figures 13

Figure S1–S3. ^{1}H and ^{13}C NMR spectra of monomers. 13

Figure S4–S22. ^{1}H and ^{13}C NMR spectra of functionlized PHAs. 15

Figure S23–S33. SEC traces of functionlized PHAs 27

Figure S34–S49. TGA and DTG curves of functionlized PHAs 32

Figure S50–S89. DSC curves of functionlized PHAs 40

Figure S90–S94. pXRD profiles of functionlized PHAs 60

Figure S95–S113. Tensile curves of functionlized PHAs 63

Figure S114. Solubility test of the crosslinked network in common organic solvents 72

Figure S115–S118. FT-IR spectra of crosslinked PHAs. 73

Figure S119–120. Frequency-dependent viscoelastic properties of crosslinked PHAs 75

Figure S121–122. Creep and recovery of crosslinked PHAs . 76

Supplementary Tables 77

Table S1–S4. Syndioselective ROP of functionlized β-lactone derivatives 77

Table S5. Synthesis of atactic functionalized homopolymer 80

Table S6–S9. Copolymerization of *rac*-β-butyrolactone with functionalized β-lactone derivatives. 80

Table S10. ROP of *rac*-β-butyrolactone. 83

Table S11. Gel Fraction analysis of crosslinked P(BL^{me}_{96}-*co*-BL^{allyl}_{4})-PETMP. 83

Table S12–S29. Tensile stress-strain data of functionlized PHAs dog-bone-shaped specimens. 83

References 90

Materials and General Methods

All synthesis and manipulations requiring dry inert atmosphere were performed under a nitrogen atmosphere using standard Schlenk techniques or in a N_2 or Ar-supplied glovebox. HPLC-grade organic solvents were first sparged extensively with nitrogen during filling 20 L solvent reservoirs and then dried by passage through activated alumina (for tetrahydrofuran (THF) and dichloromethane (DCM)) followed by passage through Q-5 supported copper catalyst (for toluene and hexanes) stainless steel columns. For the toluene used in polymerization reactions, HPLC-grade toluene was degassed and dried over sodium for 12 h, followed by distillation, and stored over activated Davison 4 Å molecular sieves. For the DCM used in polymerization reactions, HPLC-grade DCM was degassed and dried over CaH_2 for 12 h, followed by distillation, and stored over activated Davison 4 Å molecular sieves. Benzyl alcohol and isopropyl alcohol purified by distillation over CaH_2 and stored over activated Davison 4 Å molecular sieves. Diethyl ether was purchased from Fisher Scientific Co and was used after distillation over CaH_2. All other chemicals were purchased from their respective commercial sources: magnesium, epichlorohydrin, vinyl magnesium bromide, copper(I) bromide, cuprous iodide, dicobalt octacarbonyl, 2-diethylaminoethanethiol hydrochloride, *N*-acetyl-L-cysteine, 2,2-dimethoxy-2-phenylacetophenone, pentaerythritol tetrakis(3-mercaptopropionate), *meta*-chloroperoxybenzoic acid, *N*,*N*,*N*',*N*'',*N*''-pentamethyldiethylenetriamine and 1,2-epoxy-5-hexene were purchased from Fisher Scientific Co and used as received; propargyl bromide and *p*-toluenesulfonic acid monohydrate were purchased from TCI Chemical Co and used as received; azide-terminated poly(ethylene glycol) ($PEG\text{-}N_3$) were purchased from Sigma-Aldrich Chemical Co and used as received The yttrium complexes[1-4] and chromium complex[5] used in this study were synthesized according to reported procedures was synthesized according to previous literature.

General Polymerization Procedures

Metal-catalyzed ring-opening polymerization was performed in 10 mL Schlenk flasks inside an inert glovebox at an ambitious temperature. A mixture of yttrium catalyst and initiator (BnOH or ^{i}PrOH) in dichloromethane or toluene was stirred at ambient temperature for 15 min, and then a predetermined amount of monomers was added. The sealed reactors were taken out from the glove box and stirred at specified temperature. After the desired time, the polymerization was quenched by the addition of benzoic acid in $CHCl_3$ (10 mg/mL) and dissolved with $CHCl_3$, followed by

precipitation in methanol for 2–3 times. After filtration, the white polymer solid was dried in vacuo at 60 °C to a constant weight.

General Cross-Linking Procedures

Cross-linking of homopolymer: P(BL^{vinyl}) (850 mg, containing 7.59 mmol of alkene groups) was dissolved in anhydrous THF (30 mL) with stirring at room temperature. To the homogeneous solution, pentaerythritol tetrakis(3-mercaptopropionate) (PETMP) was added in amounts corresponding to 2 mol% and 5 mol% equivalents relative to alkene groups (12.9 µL and 32.2 µL, respectively), to control the degree of cross-linking. Subsequently, 2,2-dimethoxy-2-phenylacetophenone (DMPA, 3.80 mmol, 0.5 equiv. relative to alkene) was added as the photoinitiator. The mixture was irradiated with 365 nm UV light for 60 minutes at room temperature to induce thiol–ene cross-linking. After the reaction, most of the THF was removed under reduced pressure by rotary evaporation. The resulting product was washed with anhydrous methanol (3 × ~17 mL, 50 mL total) and dried under vacuum at 60 °C to a constant weight. Cross-linked P(BL^{allyl}) was synthesized using an analogous procedure.

Cross-linking of copolymer:

*mmol alkene is calculated according to literature[6]

1) finding mmol copolymer in a sample of given mass via average molecular weight:

$MW_{copolymer}$ = (MW(*rac*-BBL))*(X(*rac*-BBL)+(MW(*rac*-BL^{allyl}))*(X(*rac*-BL^{allyl}))

Example: P(BL$^{Me}_{96}$-*co*-BL$^{allyl}_{4}$) = (86.03)(0.96)+(126.07)(0.04) = 87.63 g/mol

(0.868 g copolymer)*(1 mol/87.63 g)*1000 = 9.905 mmol P(BL$^{Me}_{96}$-*co*-BL$^{allyl}_{4}$)

2) finding mmol alkene in the sample by: (mmol copolymer)*(mol % alkene)

(9.905 mmol P(BL$^{Me}_{96}$-*co*-BL$^{allyl}_{4}$))*(0.04 alkene) = 0.396 mmol alkene

Synthesis of P(BL$^{Me}_{96}$-co-BL$^{allyl}_{4}$)-PETMP: To a vial charged with P(BL$^{Me}_{96}$-*co*-BL$^{allyl}_{4}$**)** (868 mg, containing 0.396 mmol alkene groups), anhydrous THF (30 mL) was added, and the mixture was stirred at 70 °C until complete dissolution of the polymer. Subsequently, pentaerythritol tetrakis(3-mercaptopropionate) (PETMP, 37.8 µL, 0.099 mmol) was added to achieve the desired cross-linking ratio (0.25 equiv. per alkene). Then, DMPA (0.198 mmol, 0.5 equiv. relative to alkene)

was introduced as the photoinitiator. The resulting solution was irradiated with 365 nm UV light for 60 min at room temperature to initiate thiol–ene cross-linking. After completion, most of the solvent was removed by rotary evaporation. The crude cross-linked product was washed with anhydrous methanol (3 × ~17 mL, 50 mL total) and dried under vacuum at 60 °C to a constant weight. Cross-linked P(BL^{Me}_{96}-*co*-BL^{vinyl}_{4}) was synthesized using the same procedure.

Synthesis of P(BL^{Me}_{90}-co-BL^{allyl}_{10})-Upy: In a nitrogen-filled glovebox, P(BL^{Me}_{90}-*co*-BL^{allyl}_{10}) (1.00 g, containing 1.12 mmol allyl groups) was dissolved in anhydrous dichloromethane (DCM, 10 mL). To the stirred solution, UPy-SH (255 mg, 1.12 mmol, 1.0 equiv. relative to allyl groups) and 2,2-dimethoxy-2-phenylacetophenone (DMPA, 20 mg, 2 wt% relative to polymer) were added in one portion. The reaction mixture was irradiated with 365 nm UV light for 60 min at room temperature to initiate the thiol–ene click reaction. After completion, the reaction mixture was poured into cold methanol to precipitate the polymer, which was collected by centrifugation and dried under vacuum at 60 °C to constant weight, affording P(BL^{Me}_{90}-*co*-BL^{allyl}_{10})-UPy as a solid.

Post-Functionalization of Copolymers

Epoxidation of P(BL^{Me}_{92}-*co*-BL^{allyl}_{8}) *Copolymers*: P(BL^{Me}_{92}-*co*-BL^{allyl}_{8}) (500 mg, containing 0.448 mmol allyl group) was dissolved in DCM (10 mL). To the stirred solution, *meta*-chloroperoxybenzoic acid (*m*-CPBA, 232 mg, 1.344 mmol, 3.0 equiv. relative to allyl groups) was added in one portion. The reaction mixture was stirred at room temperature for 24 h. After completion, the mixture was precipitated into methanol two times to remove unreacted *m*-CPBA. The resulting polymer, P(BL^{Me}_{92}-*co*-BL^{epoxy}_{8}), was collected by filtration and dried under vacuum at 60 °C to a constant weight.

Dihydroxylation of P(BL^{Me}_{92}-*co*-BL^{epoxy}_{8}) *Copolymers:* P(BL^{Me}_{92}-*co*-BL^{epopxy}_{8}) (150 mg, containing 0.13 mmol epoxy groups) was dissolved in a mixed solvent of THF and deionized water (5 mL:5 mL). To the stirred solution, *p*-toluenesulfonic acid monohydrate (TsOH•H_2O, 24.7 mg, 0.13 mmol, 1.0 equiv. relative to epoxy groups) was added in one portion. The reaction mixture was heated at 70 °C and stirred for 7 h to promote acid-catalyzed ring-opening of the epoxides After completion, the mixture was precipitated into methanol two times to remove unreacted TsOH•H_2O. The resulting polymer, P(BL^{Me}_{92}-*co*-BL^{diOH}_{8}), was collected by filtration and dried under vacuum at 60 °C to a constant weight.

Grafting of 2-Diethylaminoethanethiol Hydrochloride onto P(BL^{Me}_{95}-*co*-BL^{allyl}_{5}) *via Thiol–Ene*

Reaction: In a nitrogen-filled glovebox, P(BL^{Me}$_{95}$-*co*-BL^{allyl}$_{5}$) (133 mg, containing 0.076 mmol allyl groups) was dissolved in CD_3Cl (2 mL). To the stirred solution, 2-diethylaminoethanethiol hydrochloride (12.8 mg, 0.076 mmol, 1.0 equiv.) and DMPA (2.7 mg, 2 wt%) were added in one portion. The mixture was irradiated with 365 nm UV light for 60 min at room temperature to initiate the thiol–ene reaction. After completion, most of the solvent was removed under reduced pressure. The crude product was precipitated into cold hexane and collected by centrifugation, then dried under vacuum at 60 °C to a constant weight.

Grafting of N-Acetyl-L-cysteine onto P(BL^{Me}$_{95}$-*co*-BL^{allyl}$_{5}$) *via Thiol–Ene Reaction:* In a nitrogen-filled glovebox, P(BL^{Me}$_{95}$-*co*-BL^{allyl}$_{5}$) (113 mg, containing 0.064 mmol propargyl groups) was dissolved in anhydrous THF (2 mL). To the stirred solution, *N*-acetyl-*L*-cysteine (10.4 mg, 0.064 mmol, 1.0 equiv.) and DMPA (2.3 mg, 2 wt%) were added in one portion. The mixture was irradiated with 365 nm UV light for 60 minutes at room temperature to initiate the thiol–ene reaction. After completion, most of the solvent was removed under reduced pressure. The crude product was precipitated into cold hexane and collected by centrifugation, then dried under vacuum at 60 °C to a constant weight.

Grafting of PEG onto P(BL^{Me}$_{95}$-*co*-BL^{hPg}$_{5}$) *via CuAAC Reaction:* In a nitrogen-filled glovebox, P(BL^{Me}$_{95}$-*co*-BL^{hPg}$_{5}$) (110 mg, containing 0.057 mmol propargyl groups) was dissolved in degassed DCM (2 mL). To the stirred solution, copper(I) bromide (8.2 mg, 0.057 mmol, 1.0 equiv.), *N*,*N*,*N*',*N*'',*N*''-pentamethyldiethylenetriamine (19.8 mg, 0.114 mmol, 2.0 equiv.), and azide-terminated poly(ethylene glycol) (PEG-N_3, $M_n \approx 2000$ Da, 138.8 mg, 0.057 mmol, 1.0 equiv.) were added in one portion. The reaction mixture was stirred at room temperature for 24 h to allow copper-catalyzed azide–alkyne cycloaddition (CuAAC) to proceed. Upon completion, the crude mixture was precipitated into cold hexane two times, filtered through a short pad of silica gel two times, washed with DCM, to remove residual copper salts. The final product was obtained by solvent removal and dried under vacuum at 60 °C to a constant weight.

Spectroscopic Characterizations

Molar Mass Measurements: Measurements of polymer absolute weight-average molar mass (M_w), number-average molar mass (M_n), and molecular weight dispersity ($Đ = M_w/M_n$) were performed via size-exclusion chromatography (SEC). The SEC instrument consisted of an Agilent HPLC system equipped with one guard column and two PLgel 5 μm mixed-C gel permeation columns

and coupled with a Wyatt DAWN HELEOS II multi (18)-angle light scattering detector and a Wyatt Optilab TrEX dRI detector. The analysis was performed at 40 °C using $CHCl_3$ as the eluent at a flow rate of 1.0 mL min^{-1}, using Wyatt ASTRA v.7.1.2 molar mass characterization software. Wyatt Technology Astra software was used for data analysis.

NMR spectra were recorded on a Varian Inova or Bruker AV-III 400 MHz spectrometer (400 MHz, ^{1}H; 101 MHz, ^{13}C) at 298 K. Chemical shifts (δ) are reported in ppm with the solvent resonance employed as the internal standard (chloroform-d_1 at 7.26 ppm for ^{1}H NMR and 77.2 ppm for ^{13}C NMR Signals are reported as integration, multiplicity (s = singlet, d = doublet, t = triplet, q = quartet, m = multiple, br = broad signal), coupling constant (s) in Hz, and assignment.

X-ray powder diffraction patterns (pXRD) of the polymers were obtained with a Bruker D8 Discover DaVinci automatic diffractometer with Cu Ka (l = 1.5418 Å) radiation (scan of 2q = 5–60° with a step size of 0.02° and count time of 1 sec/step.

Circular dichroism (CD) spectra were collected using an Aviv model 202 CD spectrometer. CD analysis was conducted on polymer solutions with concentrations of 2.0 mg mL^{-1}. Samples were prepared by dissolving the polymers in HFIP and filtering through a 0.2 μm filter.

Fourier transform infrared (FT-IR) spectroscopy was performed on a Nicolet iS-50 FT-IR Fourier Transform Infrared Spectrometer with a single pass ATR (diamond and ZnSe crystals).

Thermal Analysis

Melting transition (T_m) and glass transition (T_g) temperatures were measured by differential scanning calorimetry (DSC) on an Auto Q20, TA Instrument. All T_m and T_g values were obtained from the second scan unless indicated otherwise. Both heating rate and cooling rate were 10 °C/min unless indicated otherwise. Decomposition temperatures (T_d, defined by the temperature of 5% weight loss) and maximum rate decomposition temperatures (T_{max}) of the polymers were measured by thermal gravimetric analysis (TGA) on a Q50 TGA Analyzer, TA Instrument. Polymer samples were heated from ambient temperature to 700 °C at a heating rate of 10 °C/min. Values of T_{max} were obtained from derivative (wt %/°C) *vs.* temperature (°C) plots.

Mechanical Analysis

Tensile stress/strain testing was performed by an Instron 5966 universal testing system (10 kN load cell) on dog-bone-shaped test specimens (ASTM D638 standard; Type V) prepared via

compression molding using a Carver Bench Top Laboratory Press (Model 4386) equipped with a two-column hydraulic unit (Carver, Model 3912, maximum force 24000 psi) unless indicated otherwise. Isolated polymer materials were loaded between non-stick Teflon paper sheets into a stainless-steel mold with inset dimensions 30 × 73.5 × 0.38 mm fabricated inhouse and compressed between two 6" × 6" steel electrically heated platens clamp force 3000 psi, at temperature 10 °C higher than each material's respective T_m. Specimens for analysis were generated via compression molding and cut using an ASTM D638-5-IMP cutting die (Qualitest) to standard dimensions. Mechanical behavior was averaged for all the specimens measured for each individual species investigated. Thickness (0.33 ± 0.01 mm), width (3.18 mm), and grip length (26.4 ± 0.2 mm) of the measured dog-bone specimens were measured for normalization of data by the Bluehill measurement software (Instron). Test specimens were affixed into the screw-tight grip frame. Tensile stress and strain were measured to the point of material break at a grip extension speed of 5.0 mm min-1 at ambient conditions.

Monomer Synthesis

1) Preparation of *rac*-BL^{vinyl}

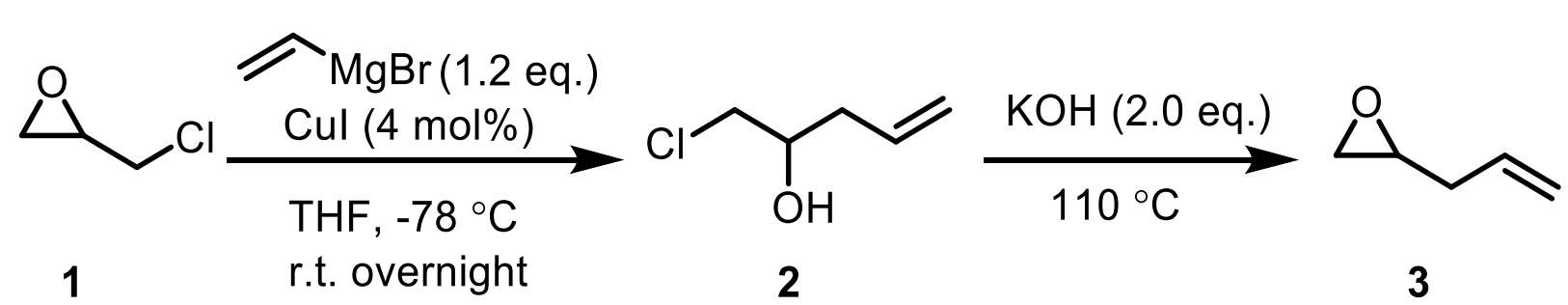

The synthesis was carried out according to a literature procedure.[7-8] Under nitrogen atmosphere, a round flask was charged with copper(I) iodide (1.9 g, 4 mol%), vinyl magnesium bromide (0.7 M in THF, 428 mL, 300 mmol) and then cooled to -78 °C. A solution of epichlorohydrin (23 g, 250 mmol) in THF (400 mL) was added slowly to the above reagent over 30 min, and then the reaction mixture was allowed to slowly reach to room temperature and stirred overnight. The reaction mixture was quenched with a saturated aqueous solution of NH_4Cl. The organic layer was washed with brine, dried over Na_2SO_4, and concentrated to afford the crude homoallylic alcohol, which on vacuum distillation provided homoallylic alcohol 1-chloropent-4-en-2-ol as a colorless liquid (21 g, 70% yield). KOH (9.82 g, 350 mmol) was added to the homoallylic alcohol in a flask fitted with a distillation head. The mixture was heated, and the product was obtained through atmospheric distillation as a colorless liquid (13.2 g, 90%).

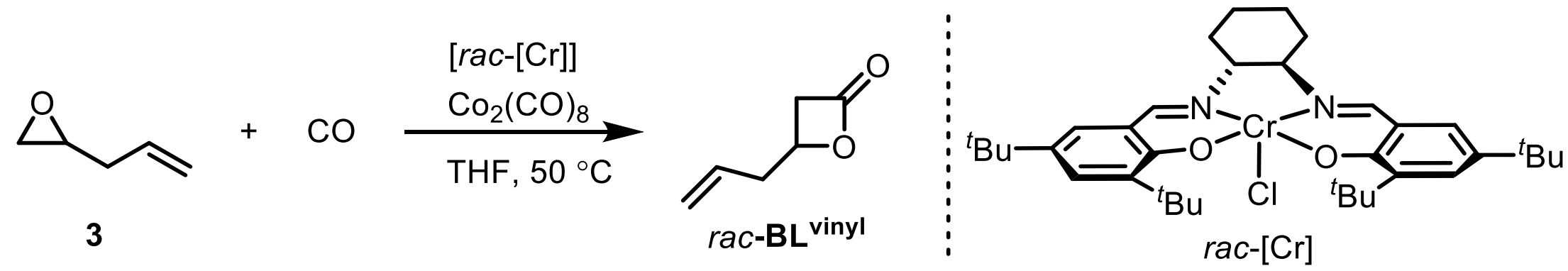

The synthesis was carried out according to a literature procedure.[9] Under nitrogen, catalyst [Cr] (569 mg, 0.9 mmol), $Co_2(CO)_8$ (306 mmol, 0.9 mmol), **3** (7.58 g, 90 mmol), THF (22.5 mL) was added to the pre-dried 100 mL autoclave equipped with a magnetic stirrer. Then the autoclave was pressured with 60 bar CO, and it was heated to 50 °C for 24 h. After cooling to room temperature, CO was released carefully. After removing the solvent (rotary evaporation at room temperature because of the product's low boiling point), *rac*-BL^{vinyl} was distilled over calcium hydride under nitrogen under reduced pressure at about 60 °C, receiving the product with dry ice acetone bath (60% yield). Colorless oil. ^{1}H NMR (400 MHz, $CDCl_3$) δ 5.85-5.72 (m, 1H), 5.23 (s, 1H), 5.21-5.16 (m, 1H), 4.59 (qd, *J* = 6.1, 4.4 Hz, 1H), 3.50 (dd, *J* = 16.3, 5.8 Hz, 1H), 3.14 (dd, *J* = 16.3, 4.3 Hz, 1H), 2.85-2.31 (m, 2H). ^{13}C NMR (101 MHz, $CDCl_3$) δ 167.9, 130.8, 119.8, 69.9, 42.5, 38.5.

Enantiomeric (*R*)-BL^{vinyl} and (*S*)-BL^{vinyl} were synthesized using the same procedure, with (*R*)-epichlorhydrin or (*S*)-epichlorhydrin as starting material.

2) Preparation of *rac*-BL^{hPg}

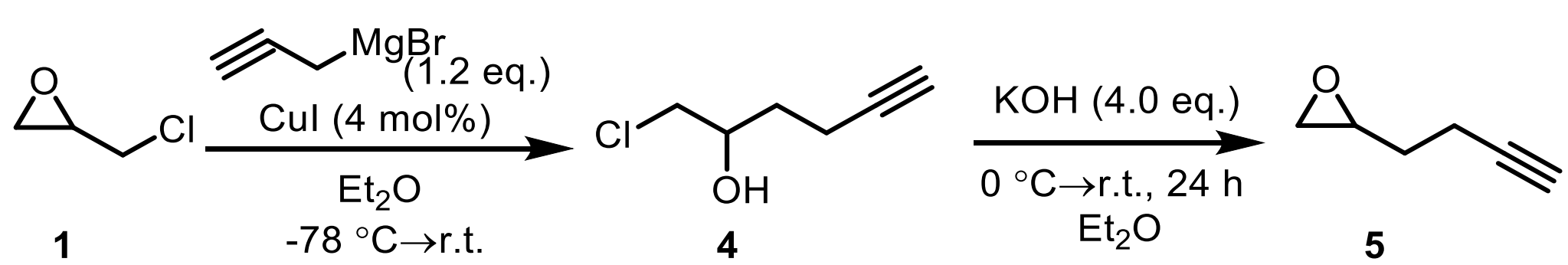

Propargylmagnesium bromide[10] was prepared as follows: Magnesium turnings (8.72 g, 359 mmol) and $HgCl_2$ (600 mg, 2.21 mmol) were added to a 500 mL round bottom flask fitted with a constant-pressure dropping funnel, a condenser, and a magnetic stirring bar. The entire set up was flamed-dried under nitrogen and allowed to cool to room temperature. Anhydrous Et_2O (100 mL) was added followed by propargyl bromide (6.67 mmol, 793 mg) and the mixture was initiated the Grignard formation. The reaction was cooled to 0 °C and a solution of propargyl bromide (427.13 mmol, 51.67 g, 1.2 eq.) in Et_2O (100 mL) was added over 1 h. The reaction was stirred at 0 °C until the magnesium turnings disappeared, then proceed directly to the next step.

Under nitrogen atmosphere, a round flask was charged with copper(I) iodide (1.42 g, 4 mol%),

anhydrous Et_2O (30 mL) was added and then cooled to -78 °C. Then added propargylmagnesium bromide solution (359 mmol, 1.2 eq.) obtained from the above steps. After that, a solution of epichlorohydrin **1** (27.76 g, 300 mmol, 1.0 eq.) in Et_2O (100 mL) was added slowly to the above reagent over 30 minutes, and then the reaction mixture was allowed to slowly reach to room temperature and stirred overnight. The reaction mixture was quenched with a saturated aqueous solution of NH_4Cl. The organic layer was washed with brine, dried over Na_2SO_4, and concentrated to afford the crude homoallylic alcohol, which on vacuum distillation provided product **4** as a colorless liquid (34 g, 257 mmol). To a solution of compound **4** in diethyl ether (Et_2O, 200 mL), KOH (57.68 g, 1.03 mol, 4.0 equiv.) powder was added at 0 °C under stirring. The mixture was then allowed to warm to room temperature and stirred for 24 h. After completion, the reaction mixture was filtered to remove inorganic solids, and the residue was washed with Et_2O (100 mL). The combined organic phases were concentrated by atmospheric distillation to remove Et_2O. The crude product was then purified by atmospheric distillation (oil bath temperature: 155 °C) to afford the desired compound as a colorless oil (18.2 g).

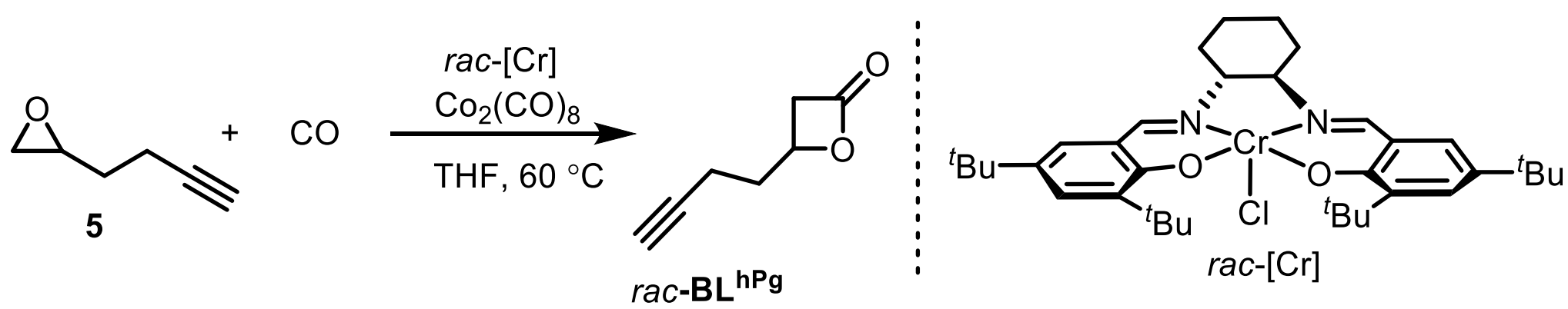

Under nitrogen atmosphere, catalyst [Cr] (653.4 mg, 1 mmol), $Co_2(CO)_8$ (336.6 mmol, 1 mmol), **5** (9.6 g, 100 mmol), THF (30 mL) was added to the pre-dried 100 mL autoclave equipped with a magnetic stirrer. Then the autoclave was pressured with 60 bar CO, and it was heated to 60 °C for 48 h. After cooling to room temperature, CO was released carefully. After removing the solvent, the product was purified by chromatography (hexane: Et_2O = 5:1). Then, it was distilled under reduced pressure using calcium hydride in an oil bath at about 110 °C, and the obtained monomer was used for polymerization reaction (4.0 g, colorless oil). Colorless oil. ^{1}H NMR (400 MHz, $CDCl_3$) δ 4.86-4.60 (m, 1H), 3.58 (dd, J = 16.4, 5.8 Hz, 1H), 3.18 (dd, J = 16.4, 4.3 Hz, 1H), 2.53-2.27 (m, 2H), 2.20-1.87 (m, 3H). ^{13}C NMR (101 MHz, $CDCl_3$) δ 167.9, 82.0, 69.9, 69.9, 43.0, 33.3, 14.5.

3) Preparation of *rac*-BL^{allyl}

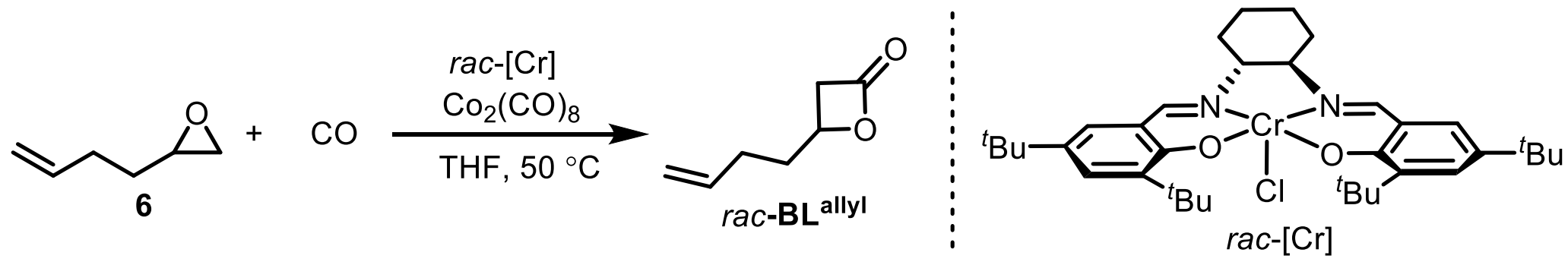

Under nitrogen atmosphere, catalyst [Cr] (189.6 mg, 0.3 mmol), $Co_2(CO)_8$ (102 mg, 0.3 mmol), **6** (15.2 g, 150 mmol), THF (30 mL) was added to the pre-dried 100 mL autoclave equipped with a magnetic stirrer. Then the autoclave was pressured with 60 bar CO, and it was heated to 50 °C for 48 h. After cooling to room temperature, CO was released carefully. After removing the solvent, the product was purified by chromatography (hexane: Et_2O = 4:1). Then, it was distilled under reduced pressure using calcium hydride in an oil bath at about 110 °C, and the obtained monomer was used for polymerization reaction (10 g, colorless oil). ^1H NMR (400 MHz, $CDCl_3$) δ 6.03-5.64 (m, 1H), 5.26-4.89 (m, 2H), 4.65-4.36 (m, 1H), 3.50 (dd, J = 16.3, 5.7 Hz, 1H), 3.07 (dd, J = 16.3, 4.0 Hz, 1H), 2.19 (qq, J = 14.3, 6.9 Hz, 2H), 1.96 (dq, J = 14.2, 7.3 Hz, 1H), 1.84 (dq, J = 14.0, 6.9 Hz, 1H).

4) Preparation of UPy-SH

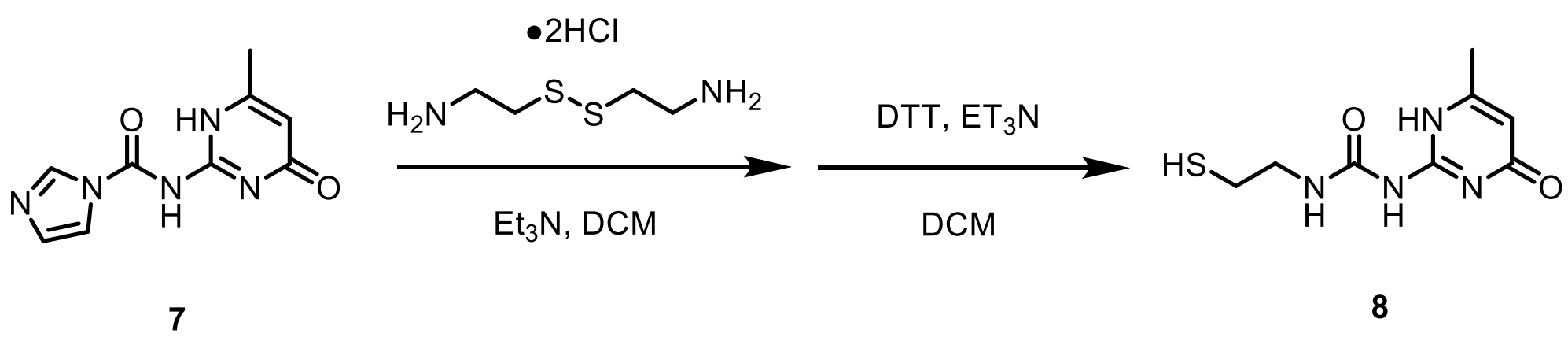

Compound **7** (5.00 g, 22.8 mmol) and triethylamine (0.91 mL) were added to cystamine dihydrochloride (2.05 g, 9.13 mmol) in DCM (91.0 mL). The reaction mixture was stirred at room temperature for 5 h, after which the formed solid was collected by filtration, washed with DCM, and dried under vacuum. Without further purification, the resulting material was treated with DL-dithiothreitol (148 mg, 0.96 mmol) and triethylamine (64.8 mg, 0.64 mmol, 1.0 equiv.) in DCM (50.0 mL) and stirred at 35 °C overnight. The reaction mixture was washed with deionized water and dried over Na_2SO_4. After removal of the solvent under reduced pressure, product 8 was obtained as a solid (0.62 g, 2.74 mmol, overall yield from compound 3: 12%). ^1H NMR (400 MHz, $CDCl_3$) δ 13.10 (s, 1H), 12.01 (s, 1H) 10.38 (s, 1H), 5.77 (s, 1H), 3.40 (t, J = 6.5 Hz, 2H), 2.76 (t, J = 6.7 Hz, 2H), 2.20 (s, 3H), 1.48 (t, J = 6.4 Hz, 1H).

Stereocomplexation Procedure

Stereocomplexes were prepared from a mixture of *(R)*-P(BL^{vinyl})+(*S*)-P(BL^{vinyl}) in a 1:1 molar ratio (approximately 300 mg total). The oily polymer sample was dissolved in $CHCl_3$ (8 mL) and filtered through a plastic frit (0.45 µm pore size nylon filter), followed by precipitation in methanol. The obtained mixture was dried in vacuo at 60 °C to a constant weight. Due to the slow crystallization rate, the mixture was left at room temperature for one month, allowing it to form a well-defined stereocomplex. *(R)*-P(BL^{vinyl}) with M_n = 10.3 kDa, $Đ$ = 1.09, P_m = 0.92; *(S)*-P(BL^{vinyl}) with M_n = 7.3 kDa, $Đ$ = 1.12, P_m = 0.93.

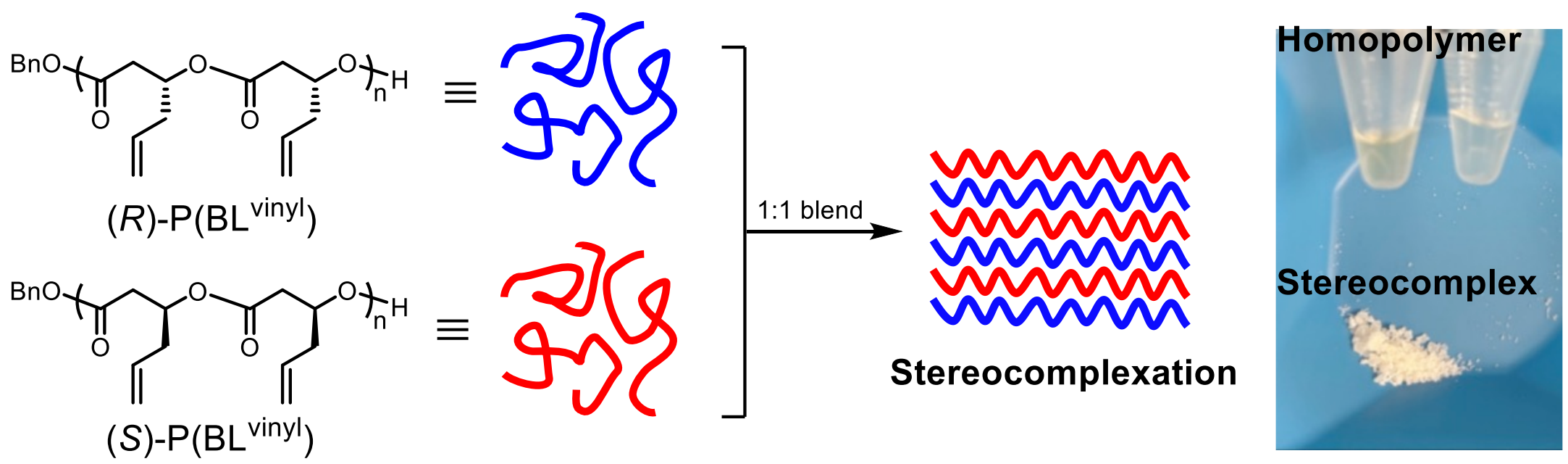

Supplementary Figures

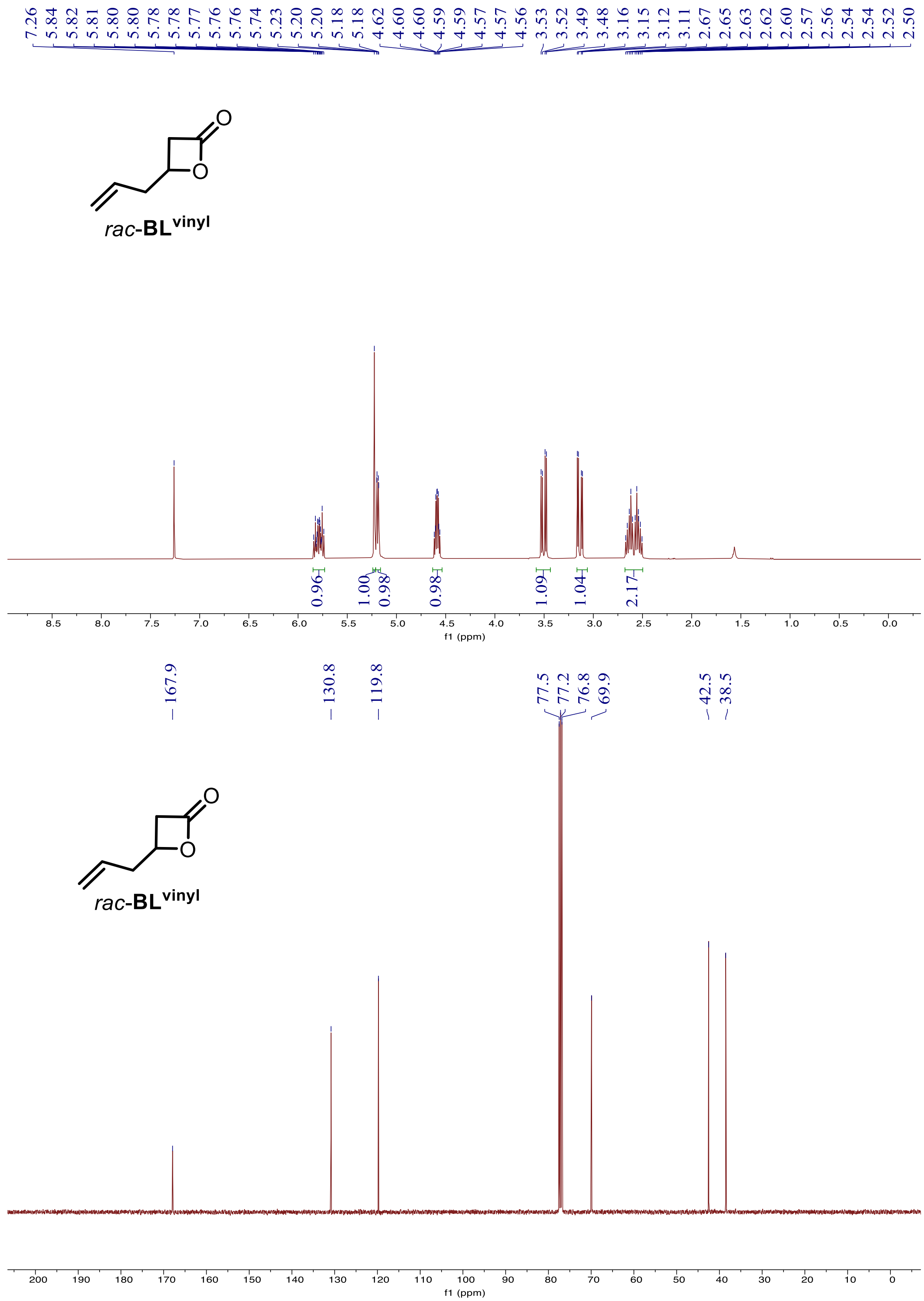

Figure S1. ^{1}H NMR and ^{13}C NMR ($CDCl_3$, 25 °C) spectra of *rac*-BL^{vinyl}.

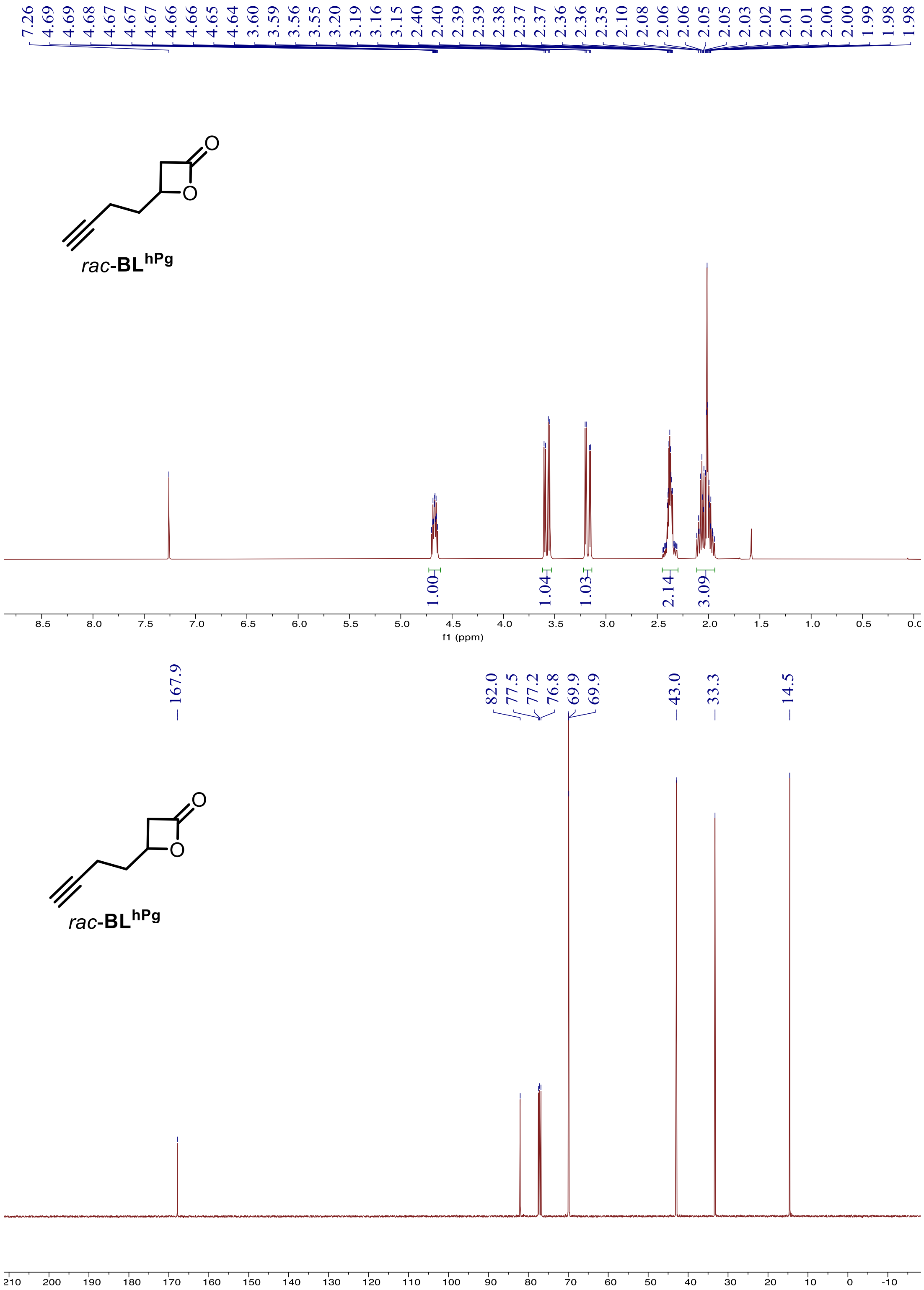

Figure S2. ^{1}H NMR and ^{13}C NMR ($CDCl_3$, 25 °C) spectra of *rac*-BL^{hPg}.

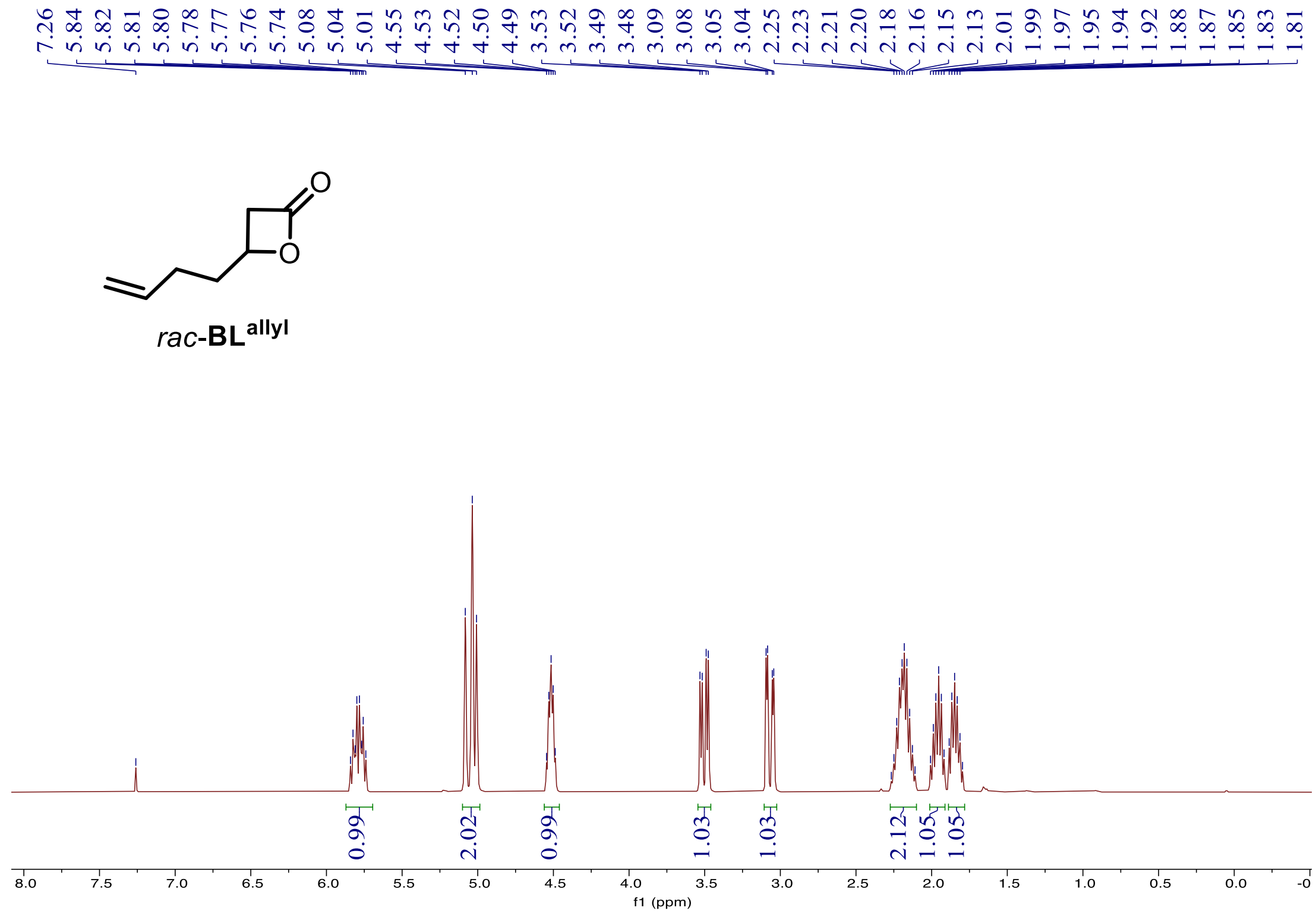

Figure S3. ^{1}H NMR ($CDCl_3$, 25 °C) spectrum of *rac*-BL^{allyl}.

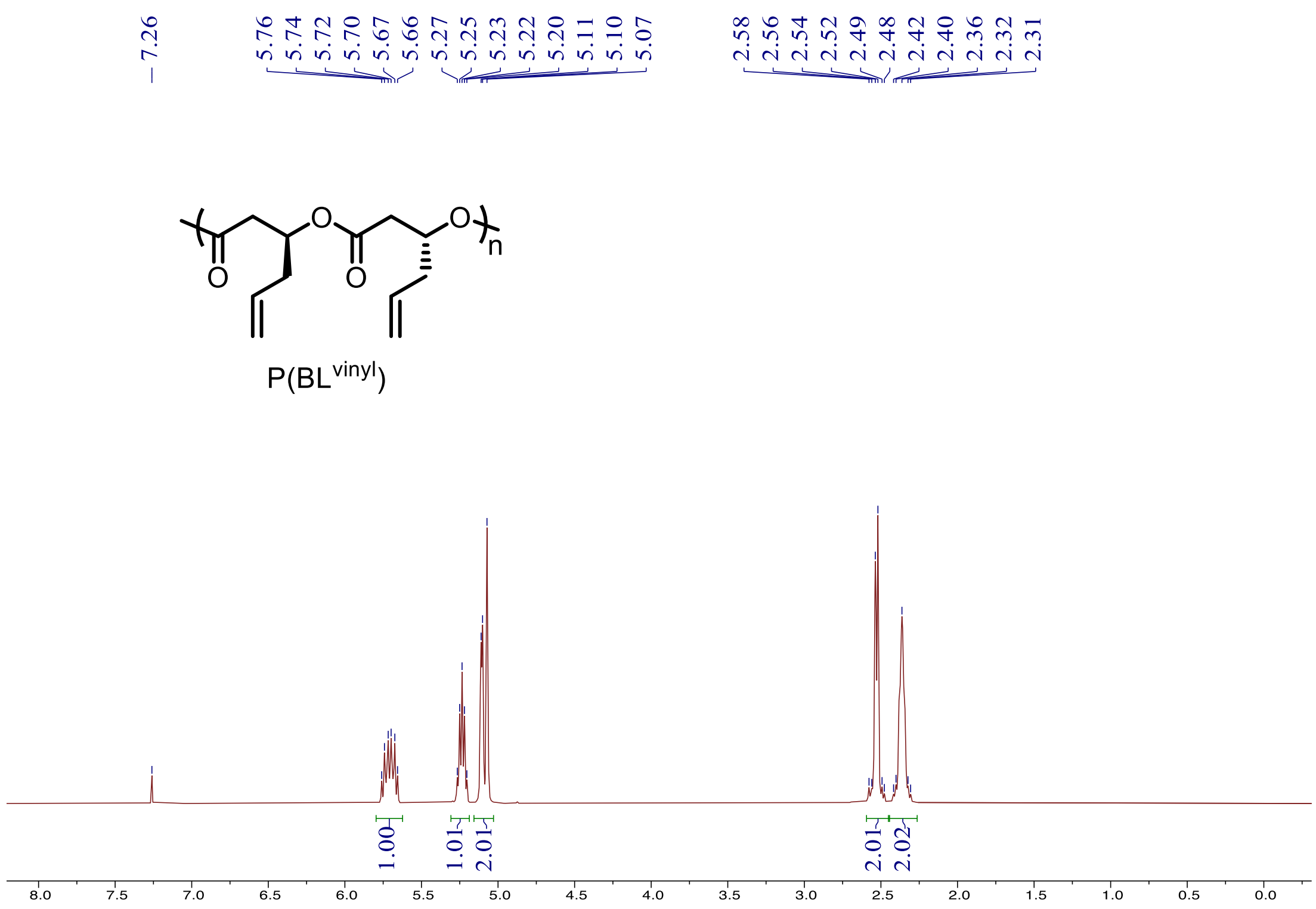

Figure S4. ^{1}H NMR ($CDCl_3$, 25 °C) spectrum of P(BL^{vinyl}).

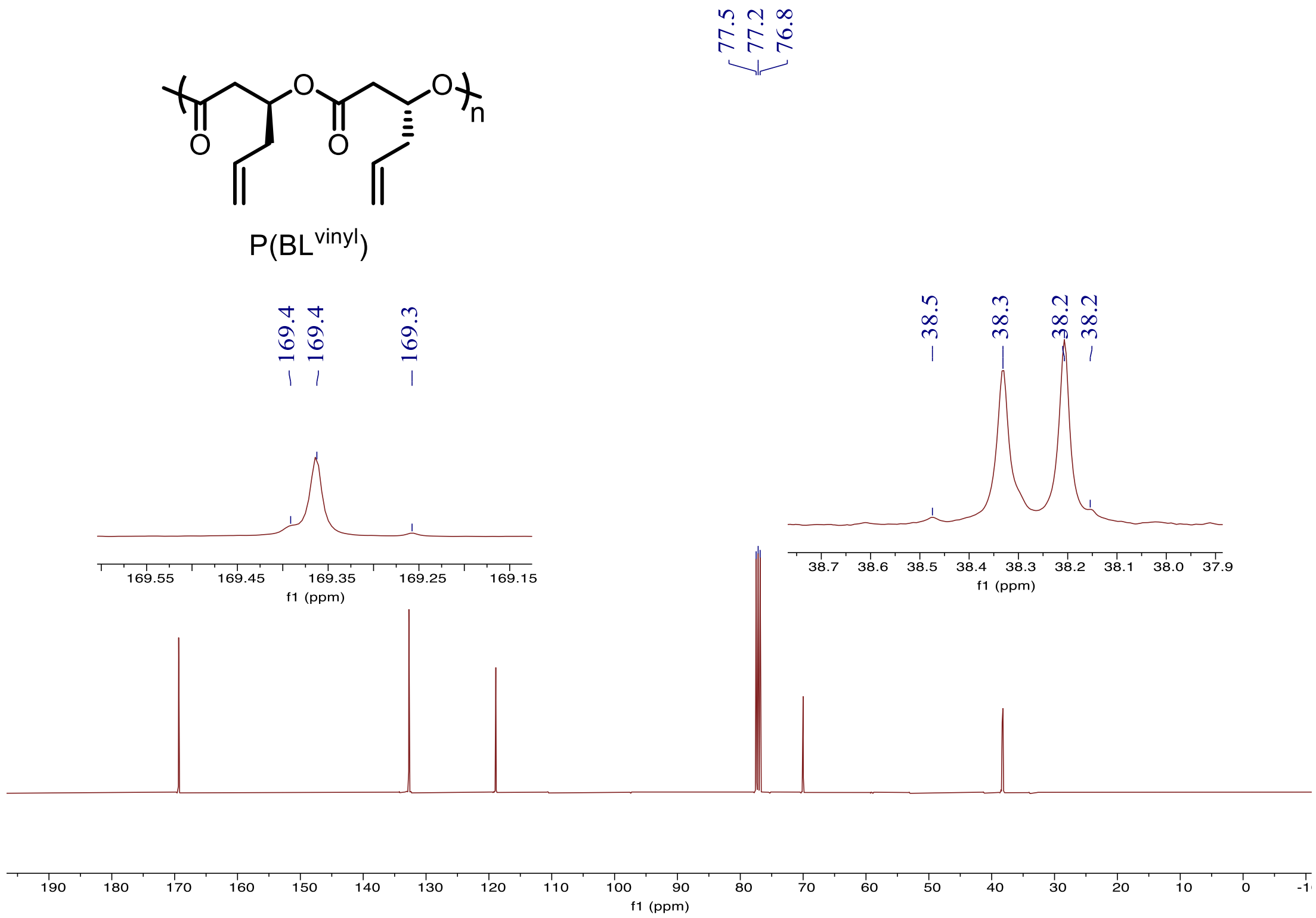

Figure S5. ^{13}C NMR ($CDCl_3$, 25 °C) spectrum of P(BL^{vinyl}), $P_r = 0.91$.

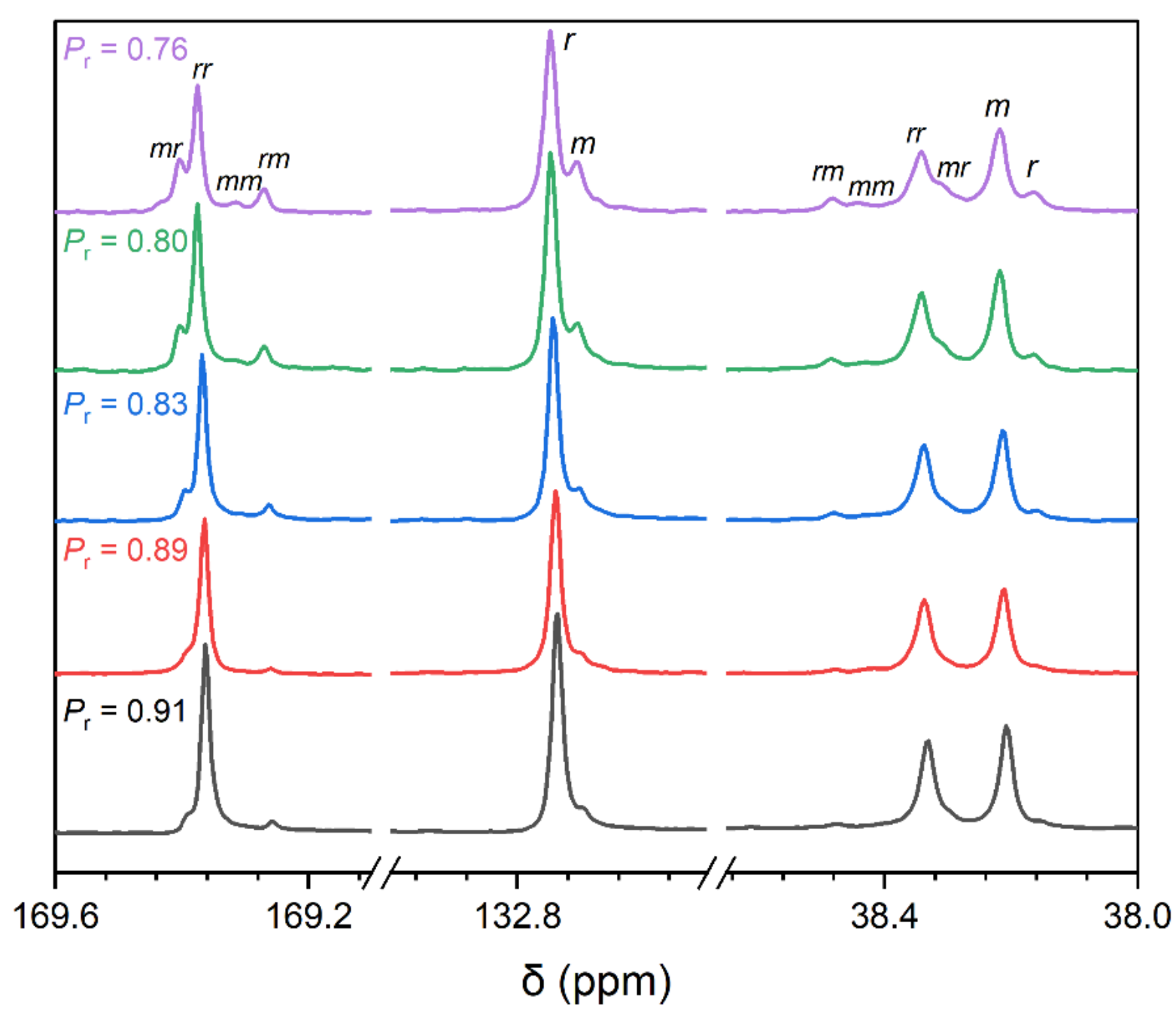

Figure S6. Stereomicrostructures of P(BL^{vinyl}). Overlayed ^{13}C NMR spectra ($CDCl_3$) in the carbonyl, alkene carbon and methylene (CH_2-main-chain; CH_2-vinyl side-chain) regions.

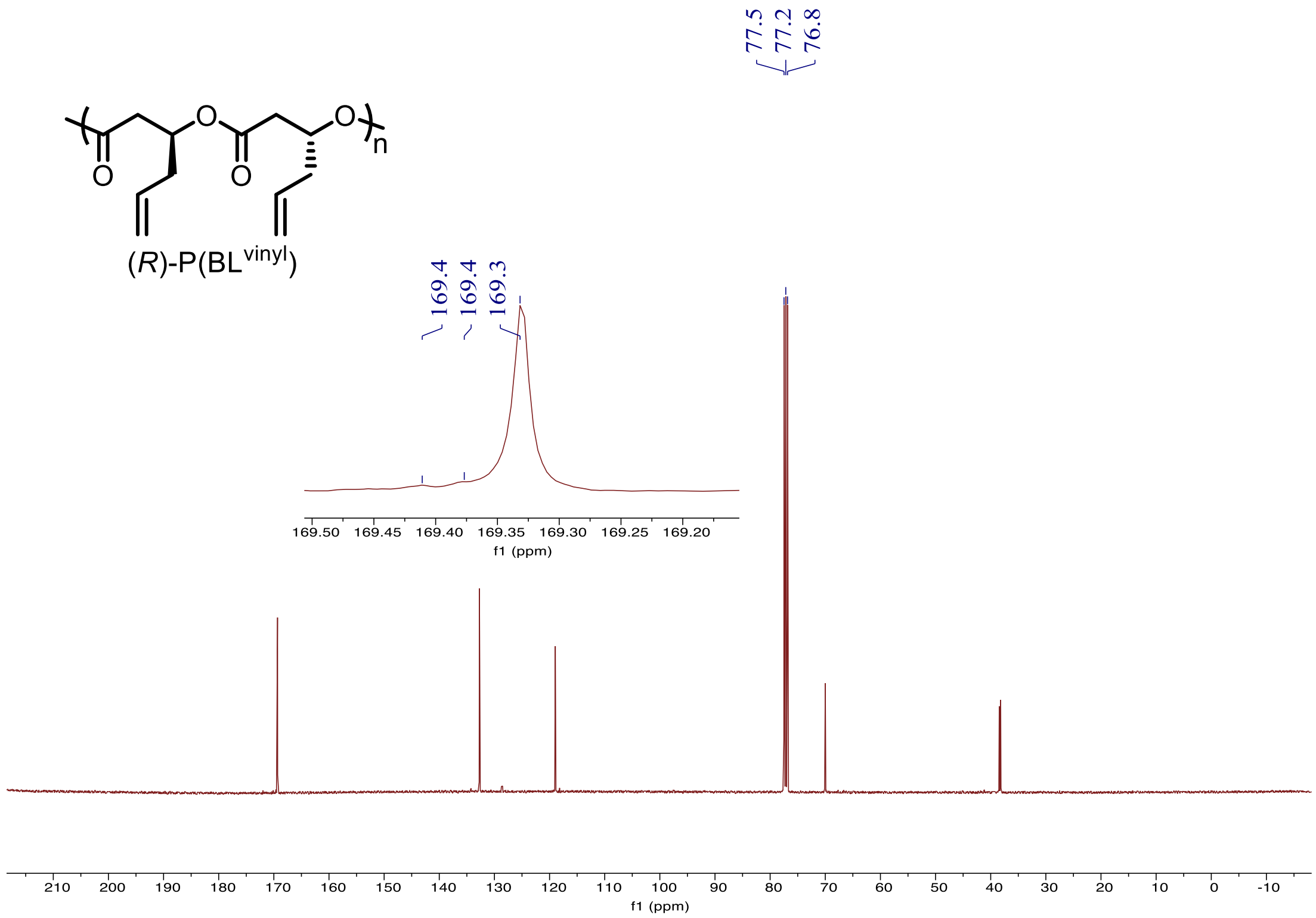

Figure S7. ^{13}C NMR ($CDCl_3$, 25 °C) spectrum of (*R*)-P(BL^{vinyl}), $P_m = 0.92$.

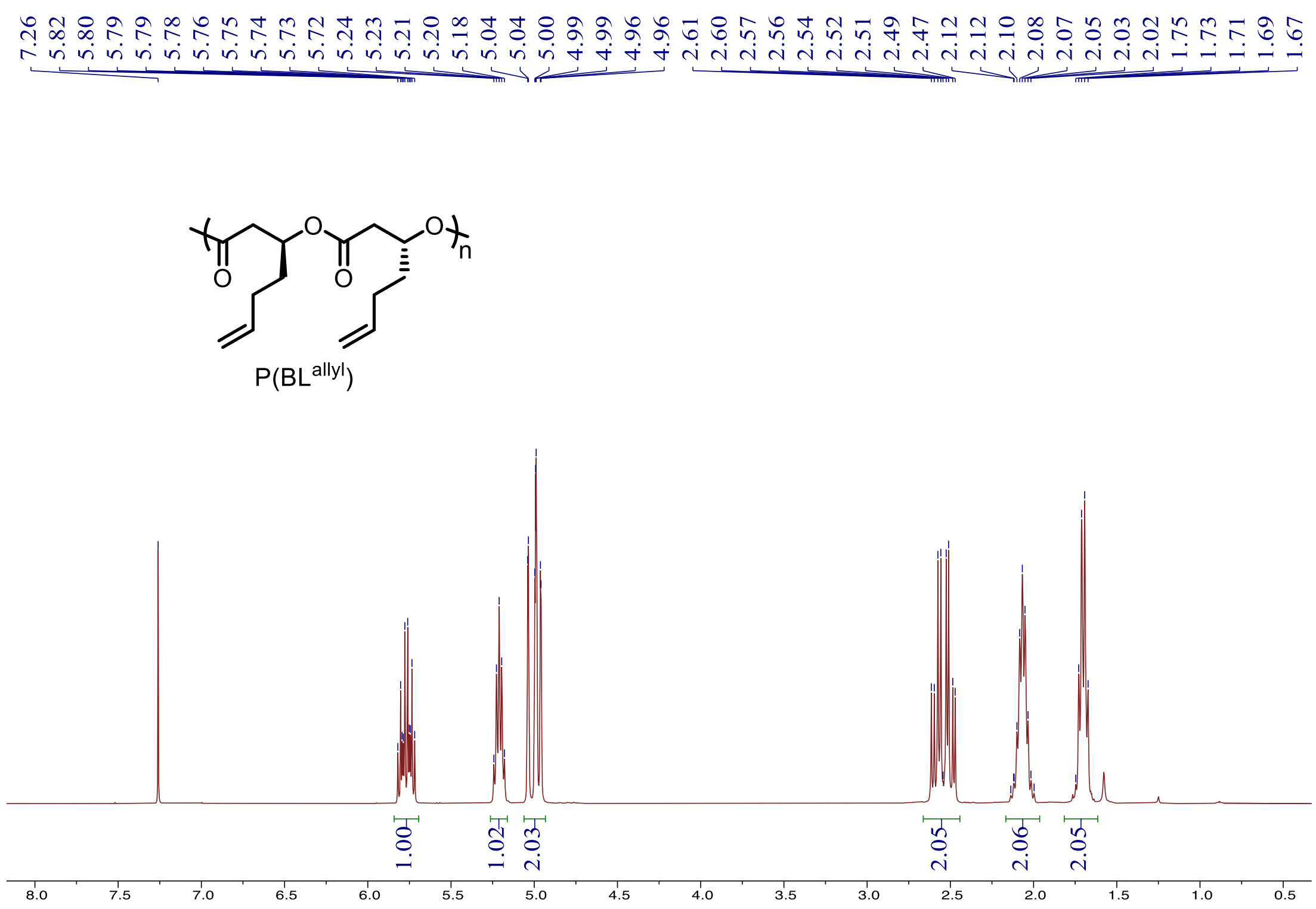

Figure S8. ^{1}H NMR ($CDCl_3$, 25 °C) spectrum of P(BL^{allyl}).

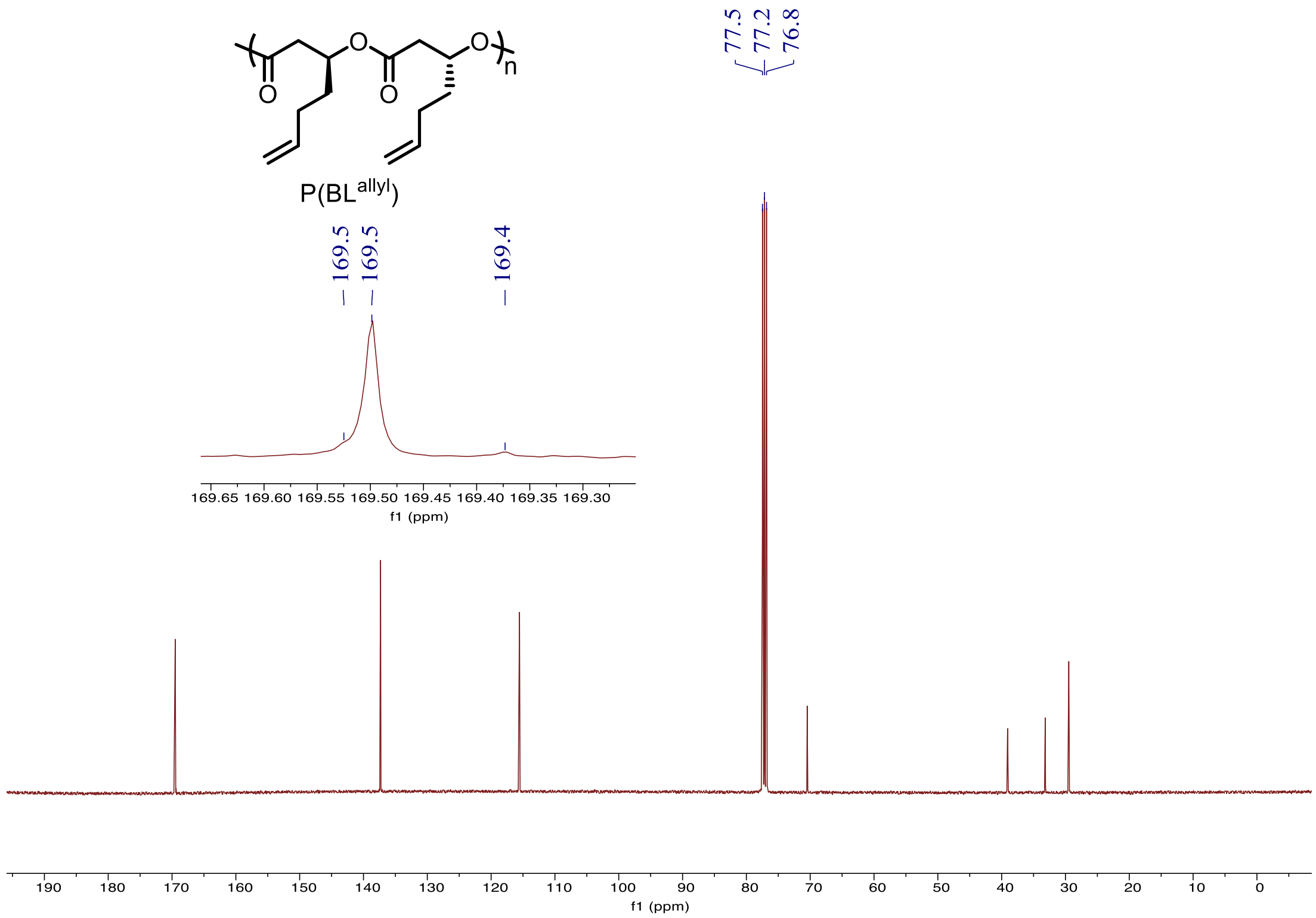

Figure S9. ^{13}C NMR ($CDCl_3$, 25 °C) spectrum of P(BL^{allyl}), $P_r = 0.95$.

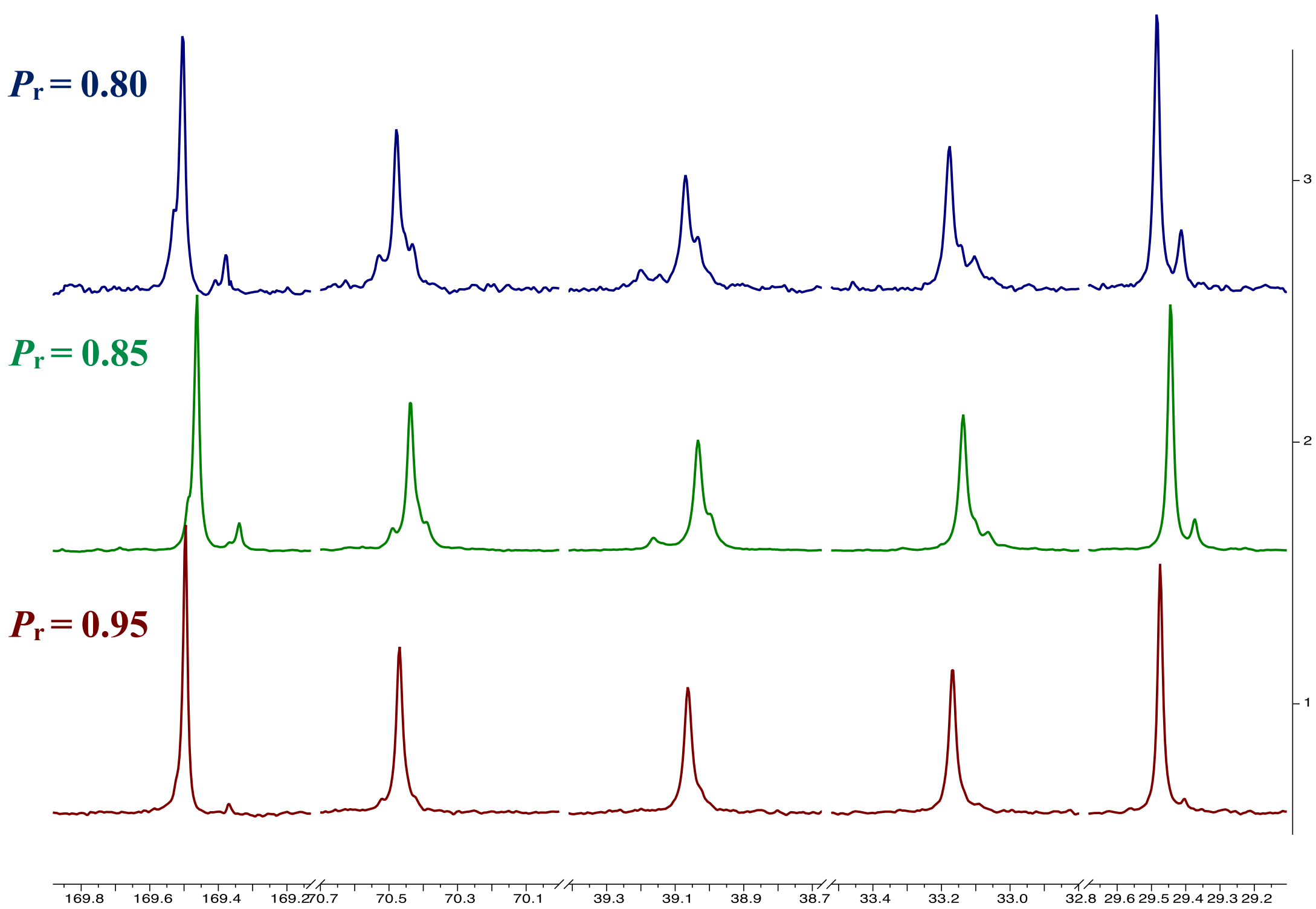

Figure S10. Stereomicrostructures of P(BL^{allyl}). Overlayed ^{13}C NMR ($CDCl_3$, 25 °C) spectra of P(BL^{allyl}) with different syndiotacticity.

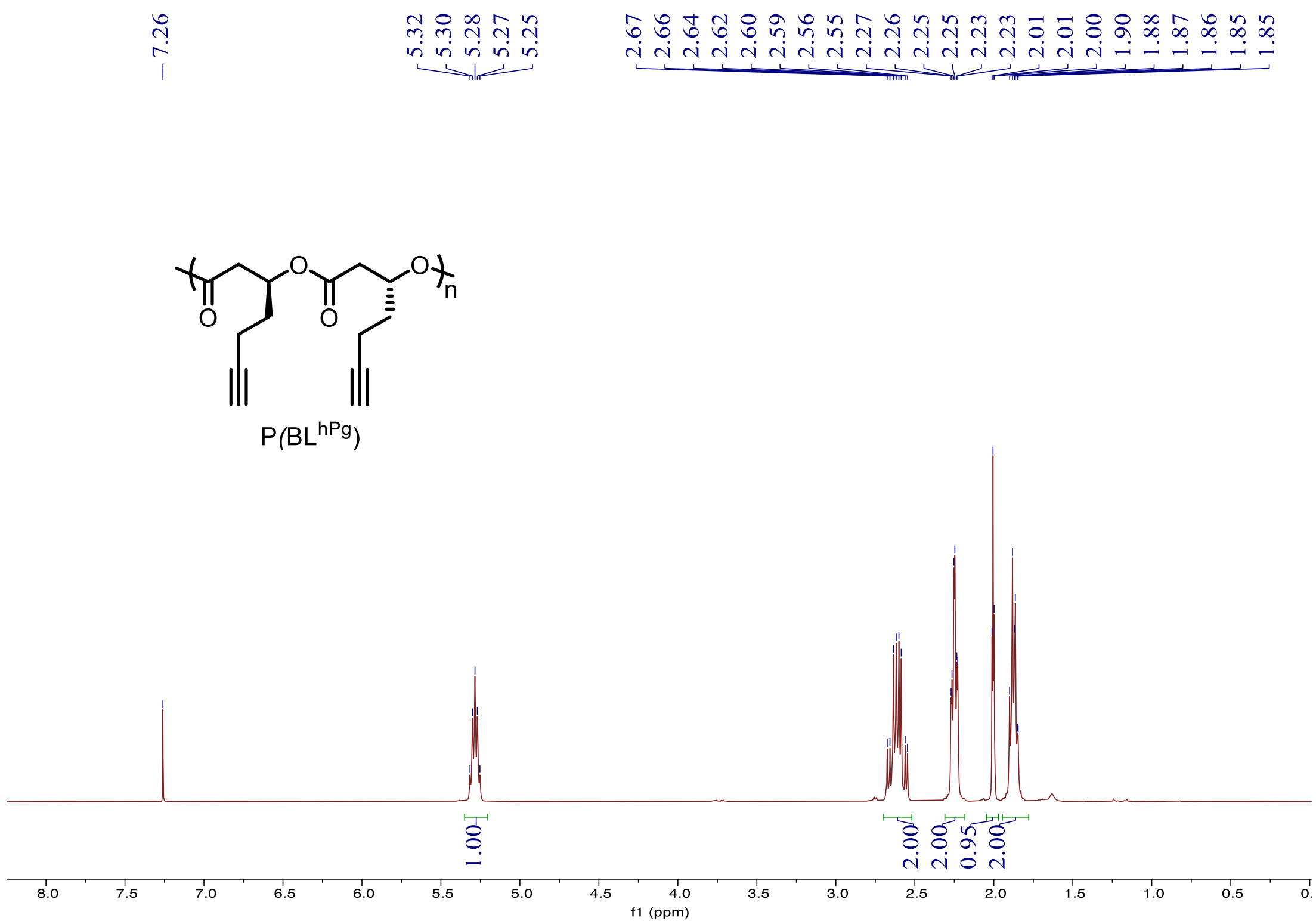

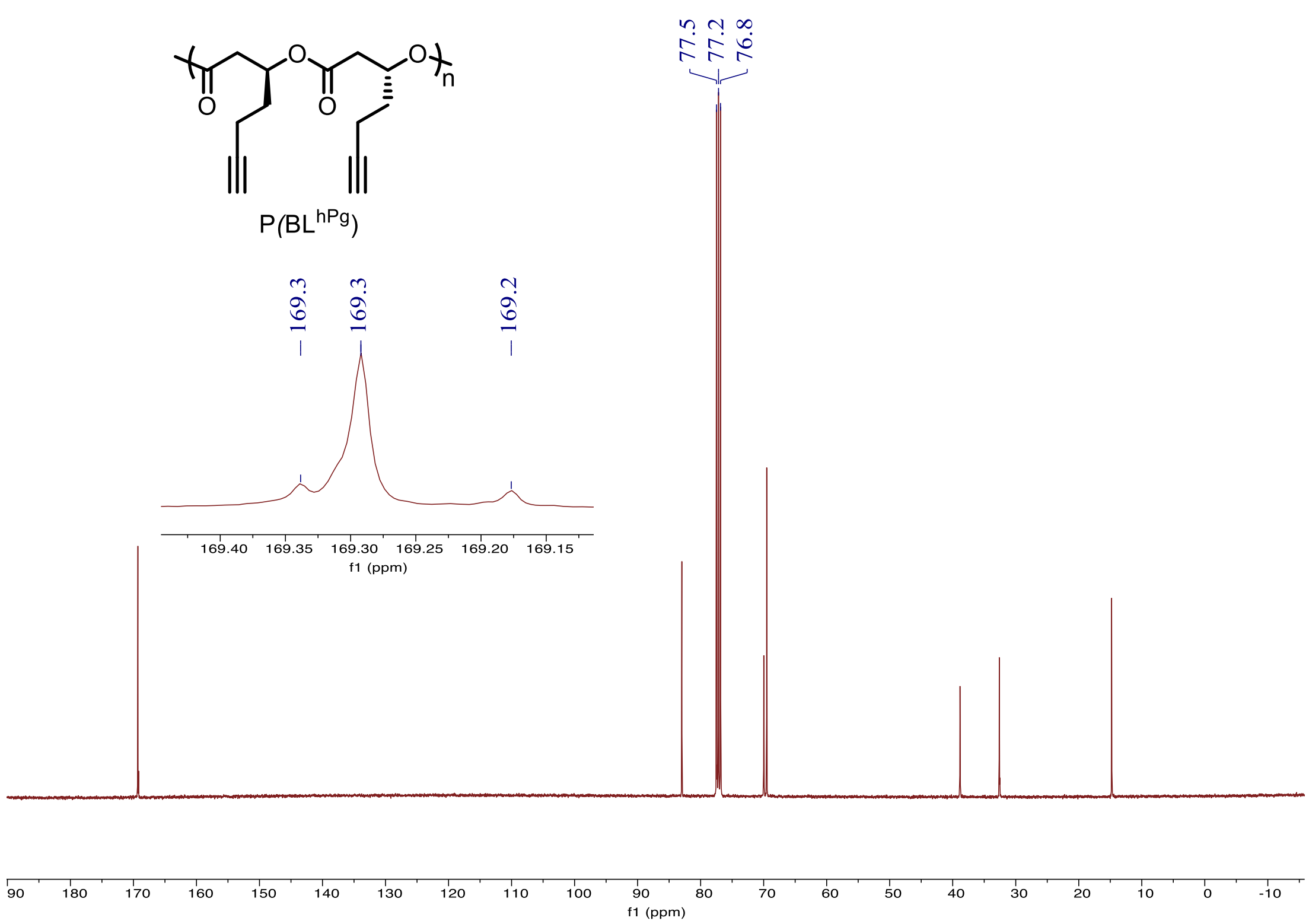

Figure S11. ^{1}H NMR and ^{13}C NMR ($CDCl_3$, 25 °C) spectra of P(BL^{hPg}), P_r = 0.90.

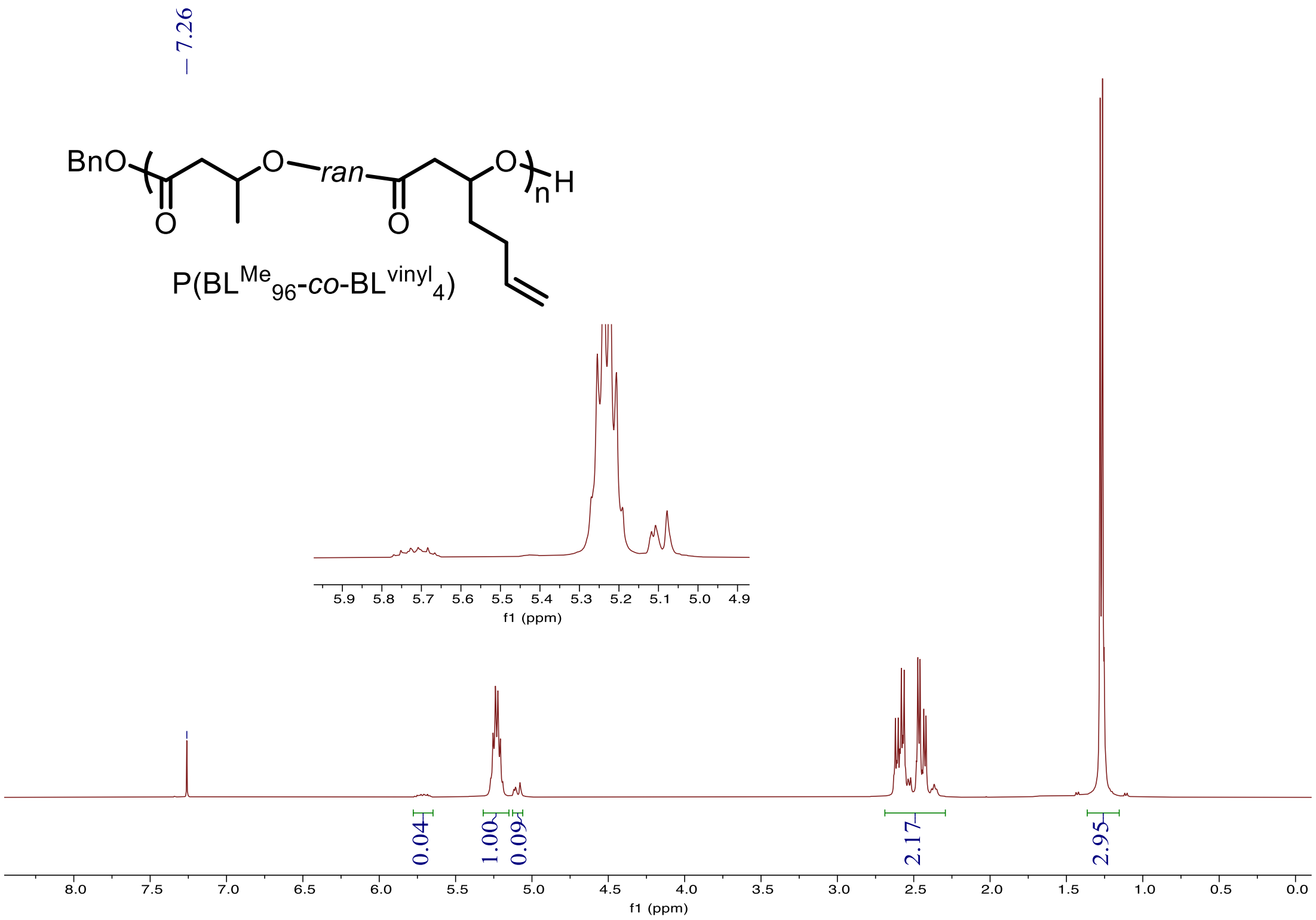

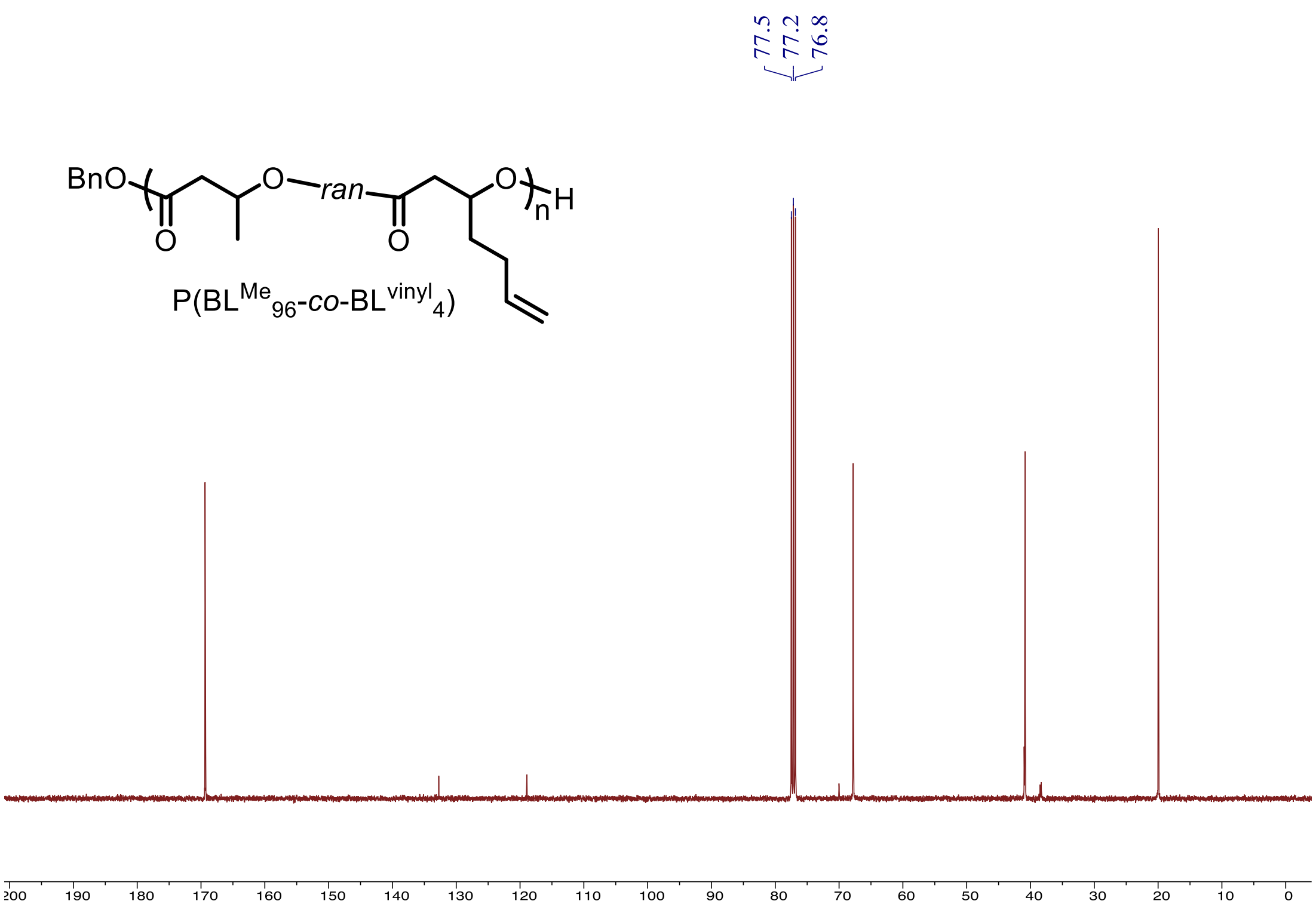

Figure S12. ^{1}H NMR and ^{13}C NMR ($CDCl_3$, 25 °C) spectra of P(BL$^{Me}_{96}$-*co*-BL$^{vinyl}_{4}$).

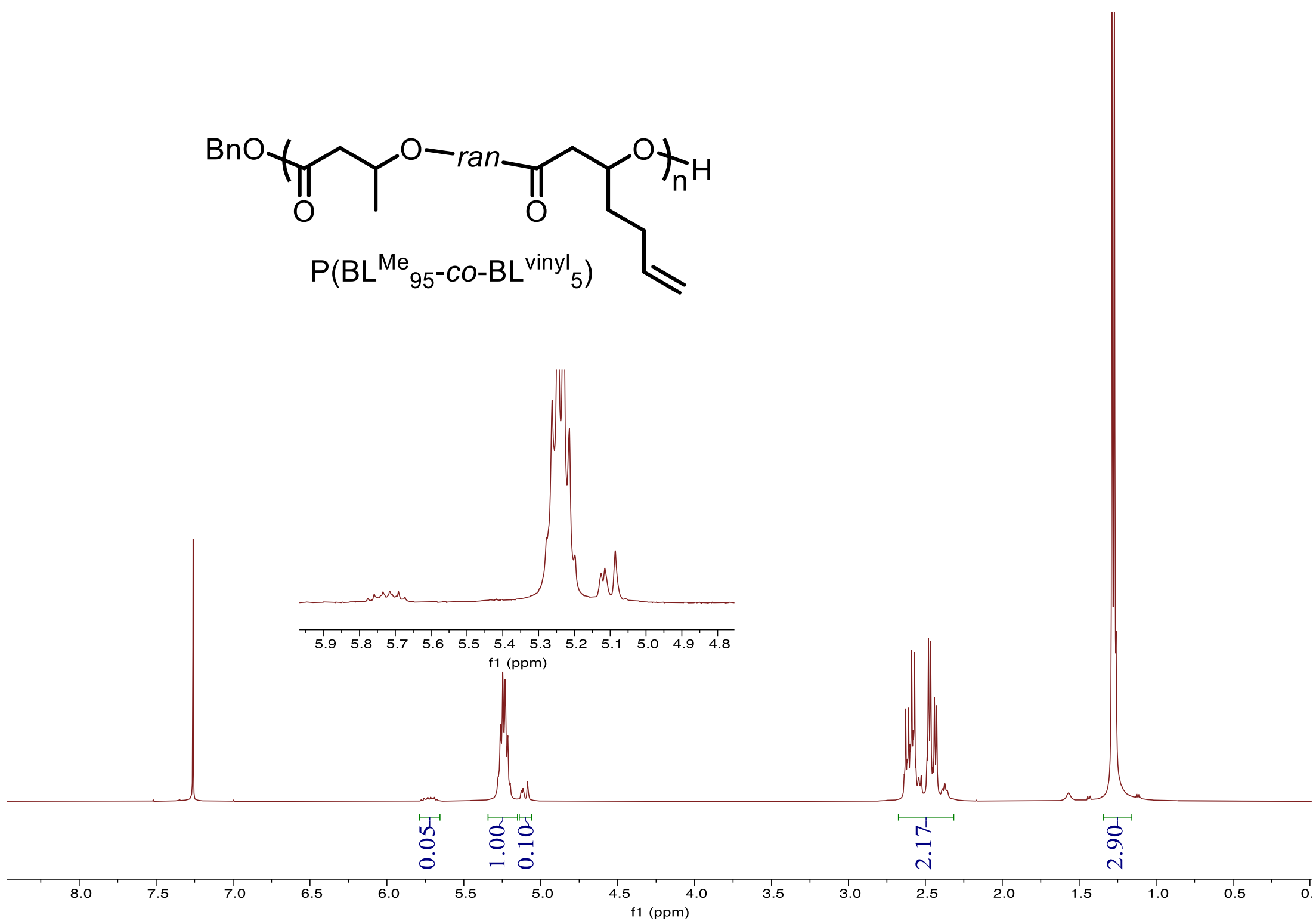

Figure S13. ^{1}H NMR ($CDCl_3$, 25 °C) spectrum of P(BL^{Me}$_{95}$-*co*-BL^{vinyl}$_5$).

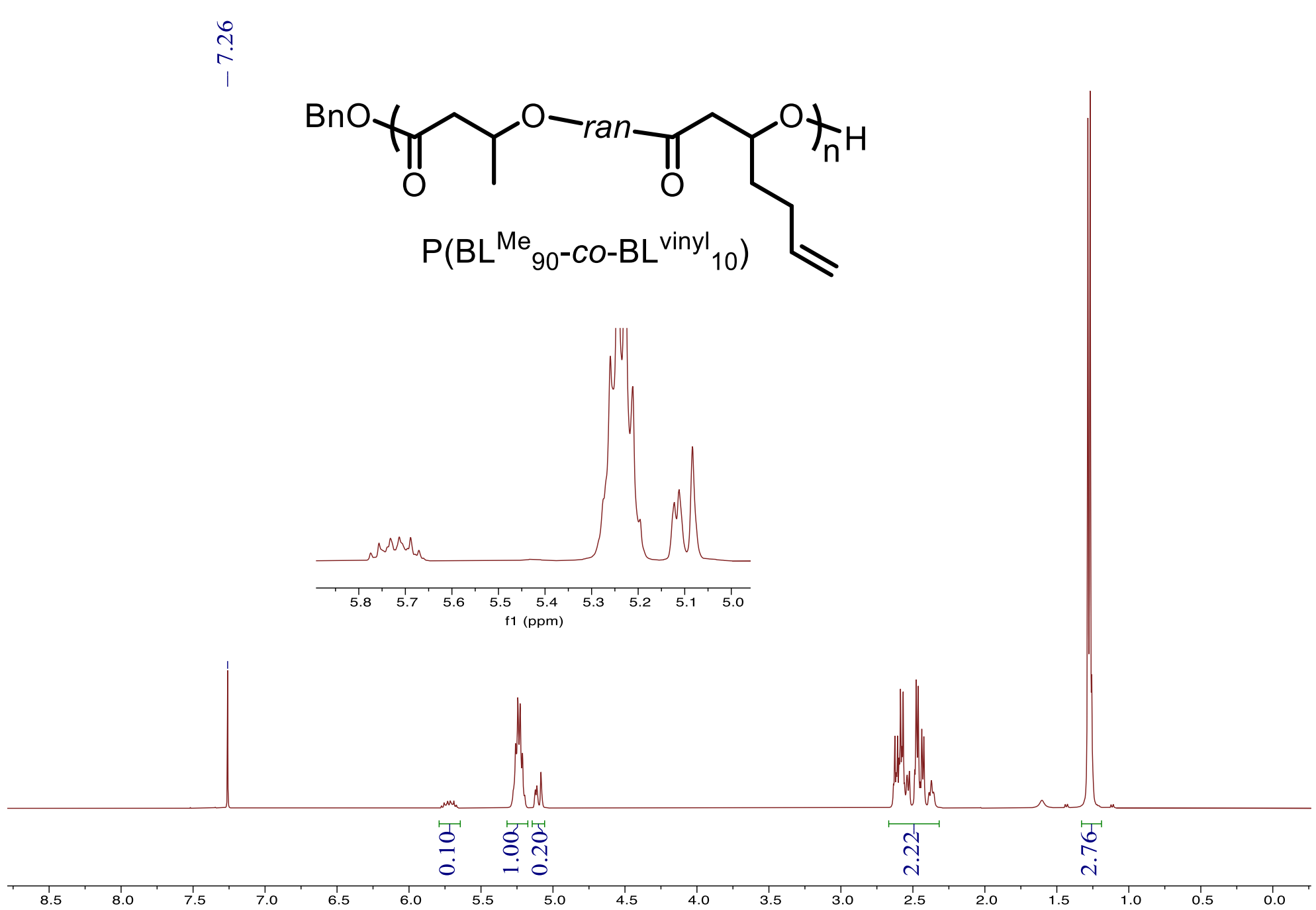

Figure S14. ^{1}H NMR ($CDCl_3$, 25 °C) spectrum of P(BL^{Me}$_{90}$-*co*-BL^{vinyl}$_{10}$).

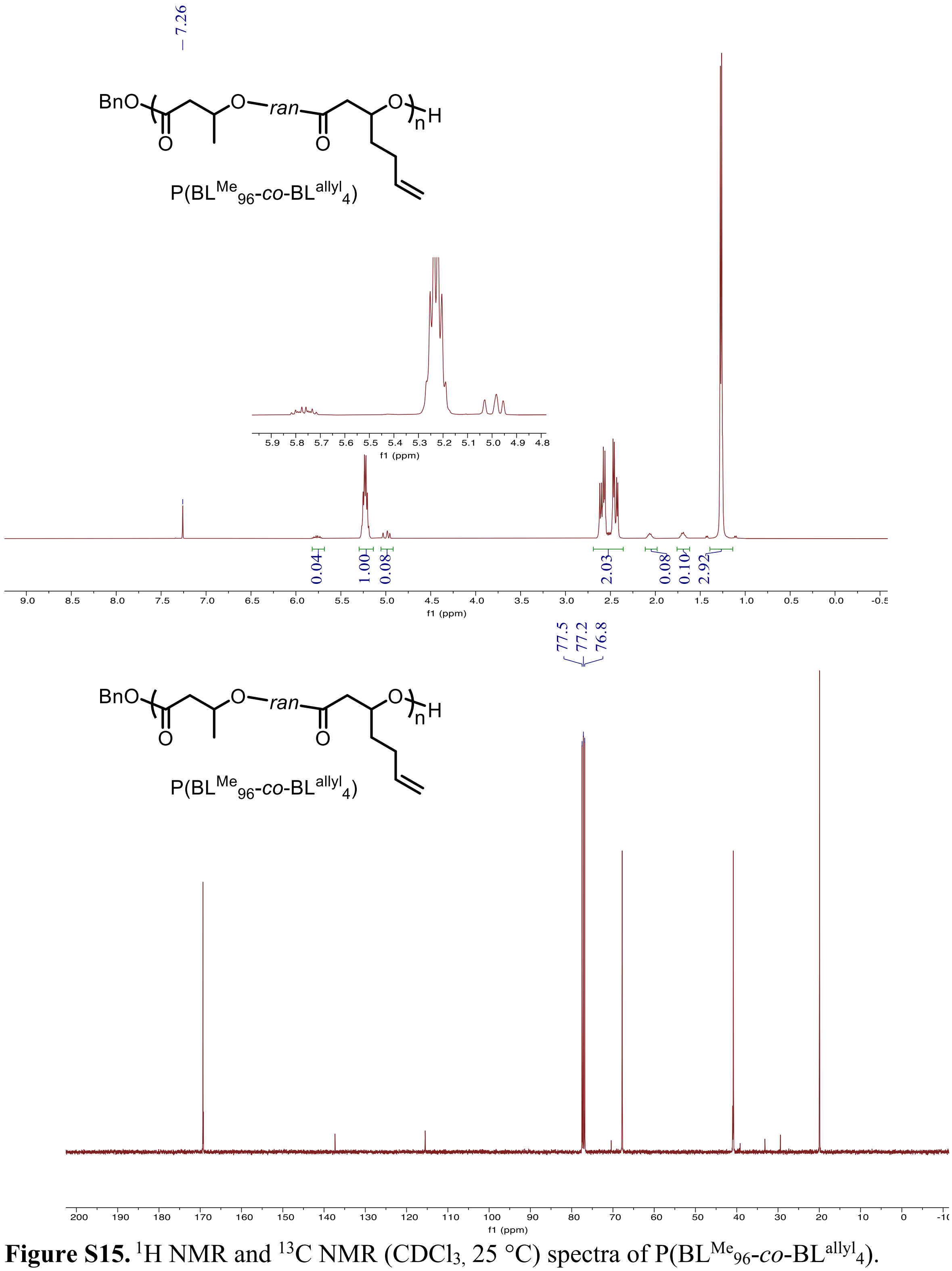

Figure S15. ^{1}H NMR and ^{13}C NMR ($CDCl_3$, 25 °C) spectra of P(BL$^{Me}_{96}$-*co*-BL$^{allyl}_{4}$).

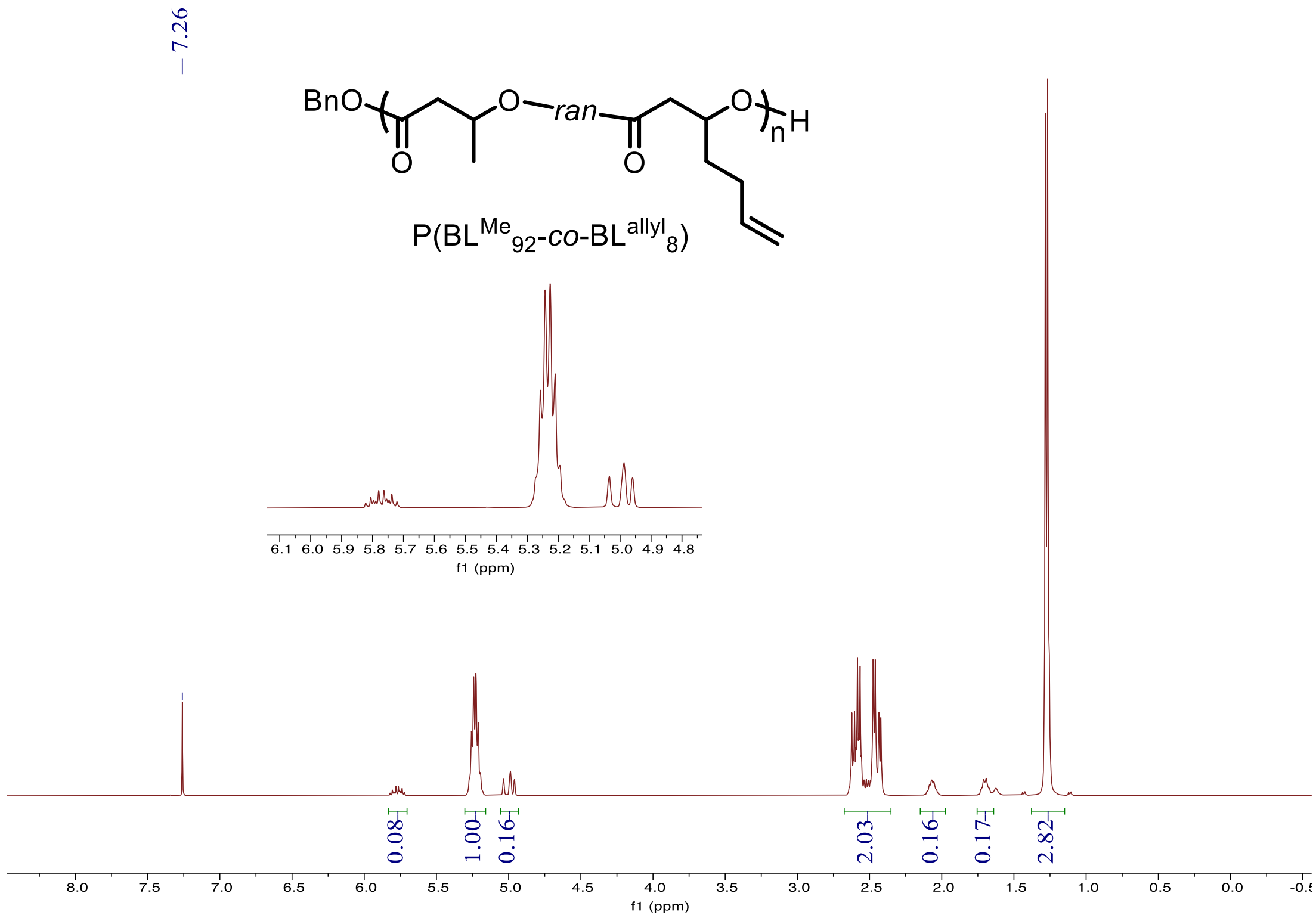

Figure S16. ^{1}H NMR ($CDCl_3$, 25 °C) spectrum of P(BL^{Me}_{92}-*co*-BL^{allyl}_{8}).

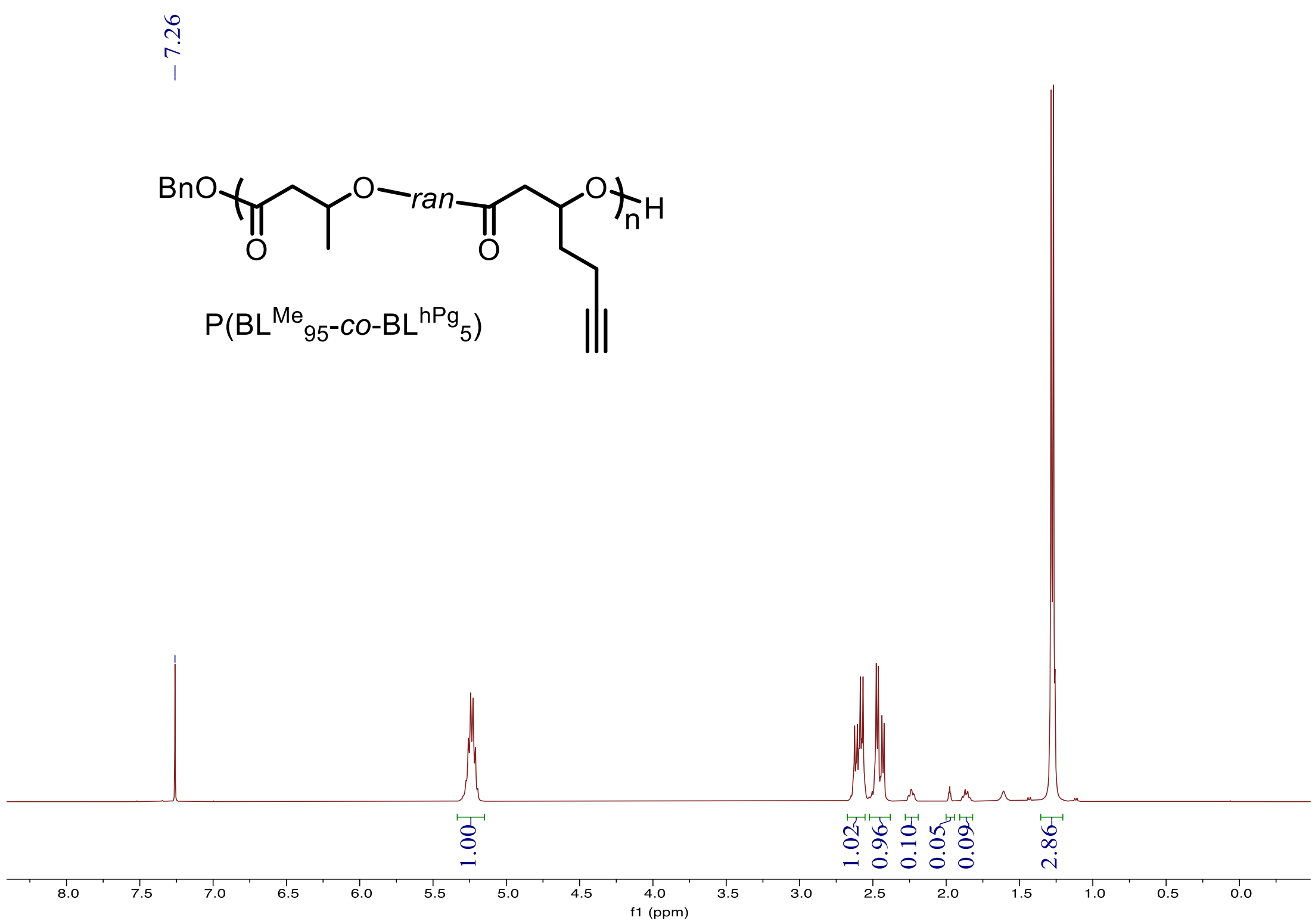

Figure S17. ^{1}H NMR and ($CDCl_3$, 25 °C) spectrum of P(BL^{Me}_{95}-*co*-BL^{hPg}_{5}).

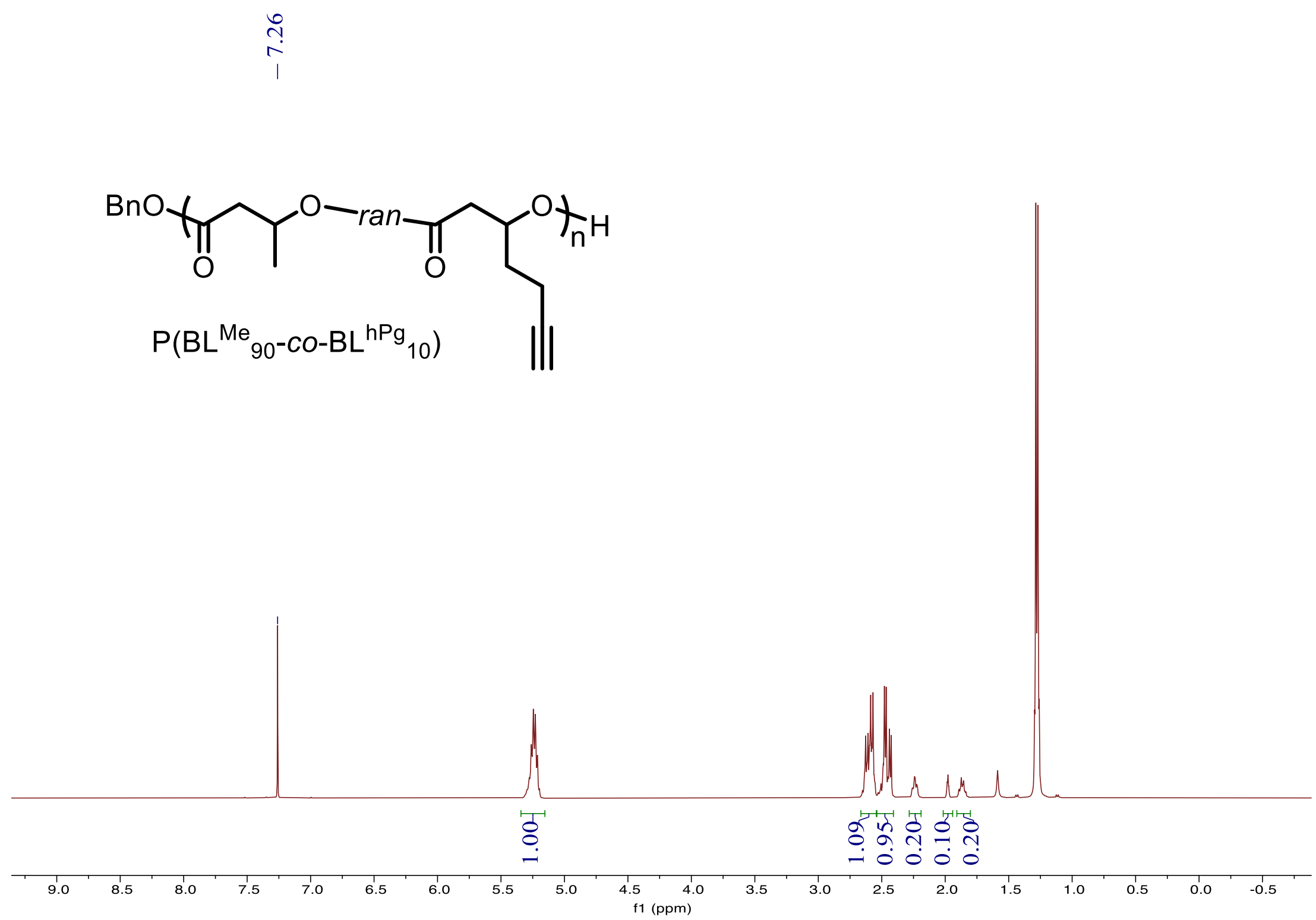

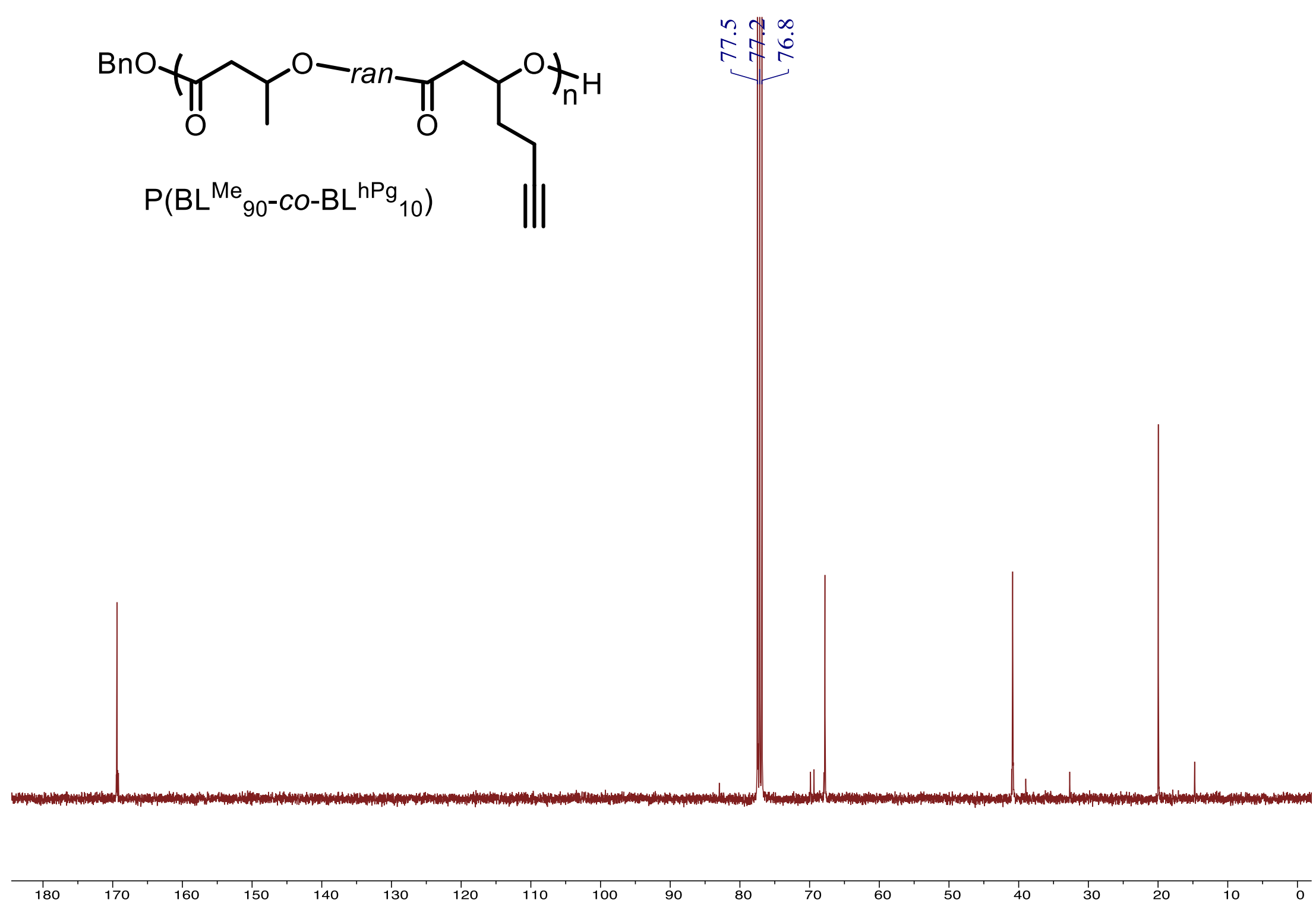

Figure S18. ^{1}H and ^{13}C NMR ($CDCl_3$, 25 °C) spectra of P($BL^{Me}{}_{90}$-*co*-$BL^{hPg}{}_{10}$).

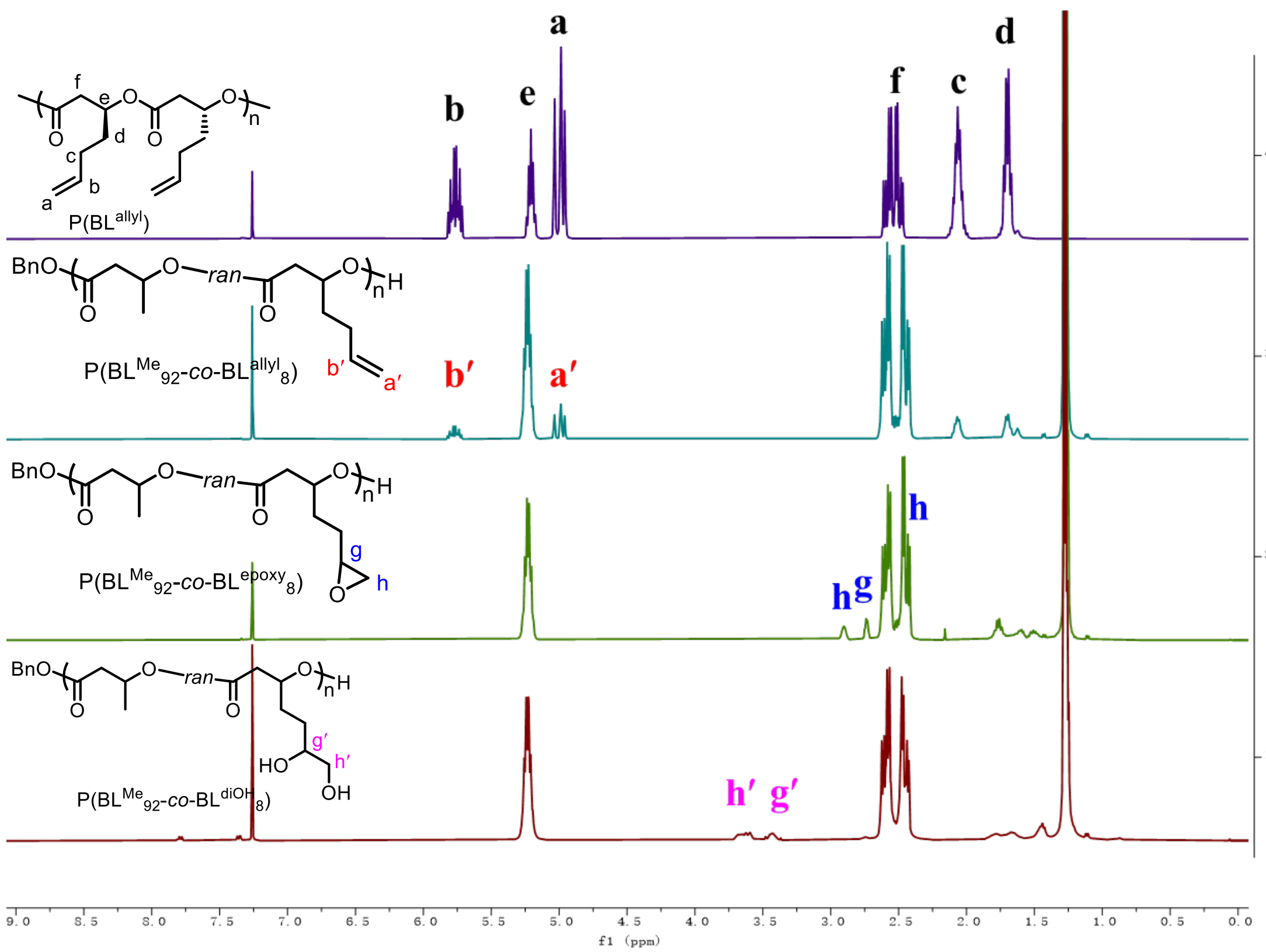

Figure S19. Overlayed ^{1}H NMR ($CDCl_3$, 25 °C) spectra of functionalized copolymer.

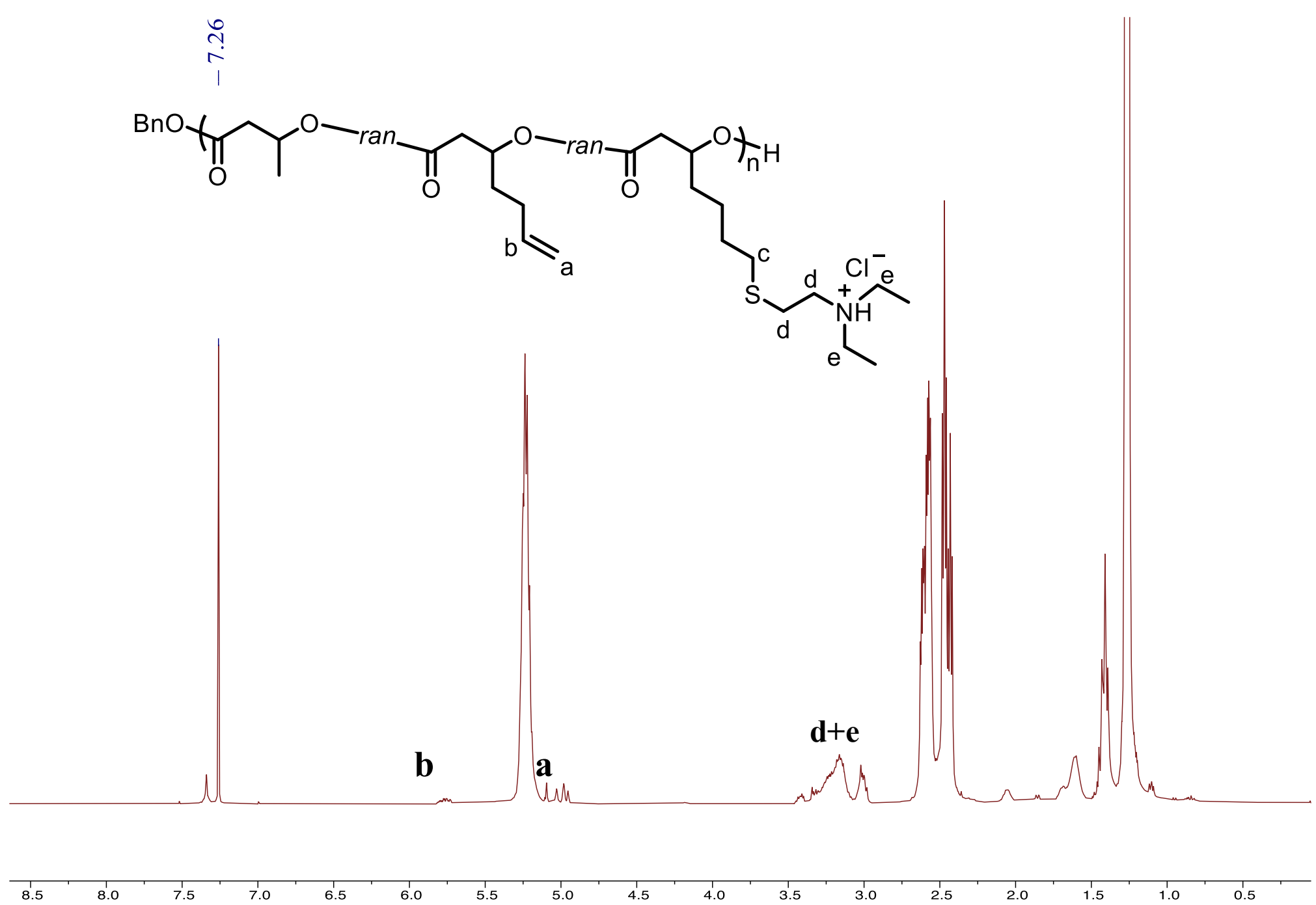

Figure S20. ^{1}H NMR ($CDCl_3$, 25 °C) spectrum of amine functionalized copolymer.

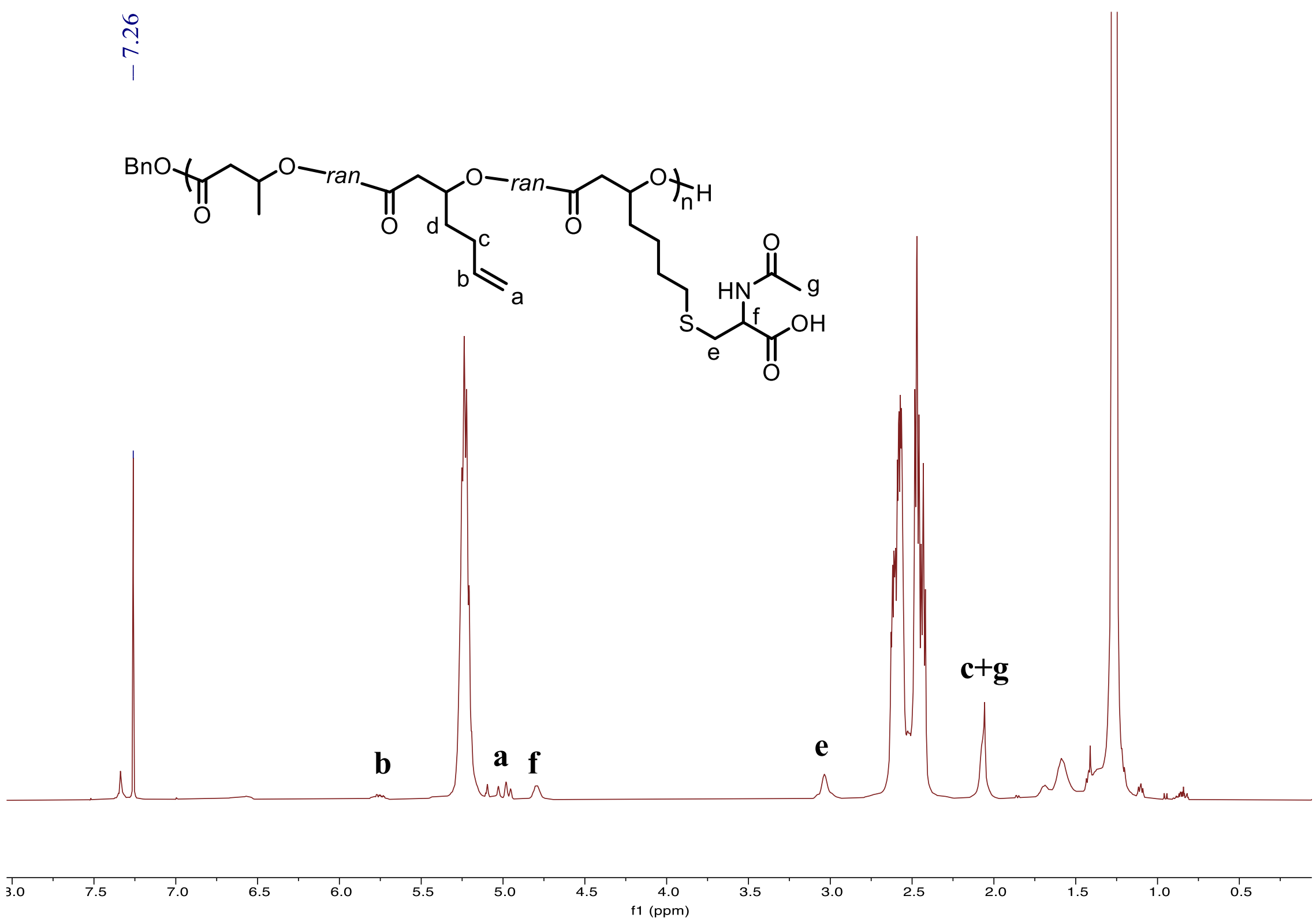

Figure S21. ^{1}H NMR ($CDCl_3$, 25 °C) spectrum of amino acid functionalized copolymer.

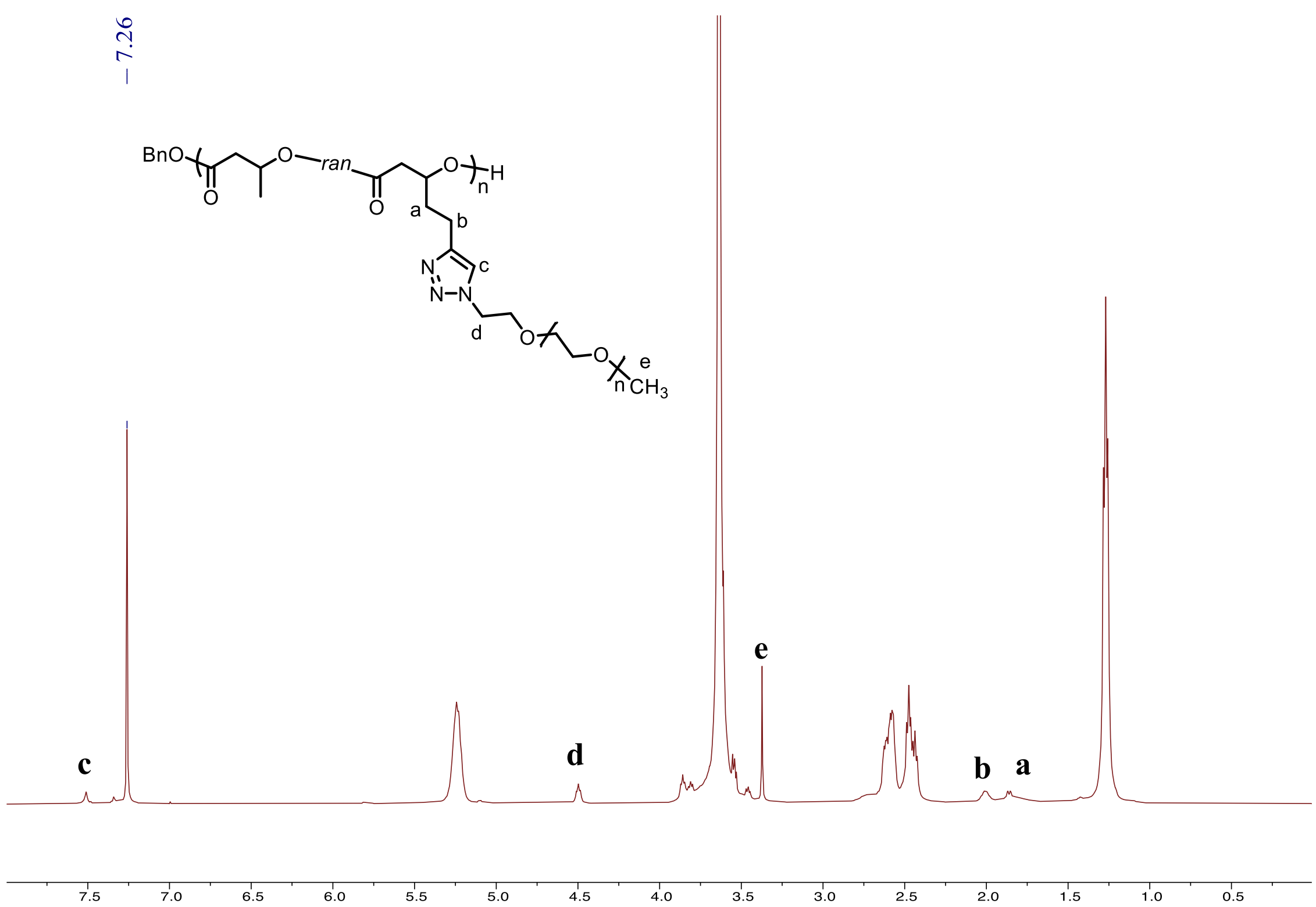

Figure S22. ^{1}H NMR ($CDCl_3$, 25 °C) spectrum of PEG graft copolymer.

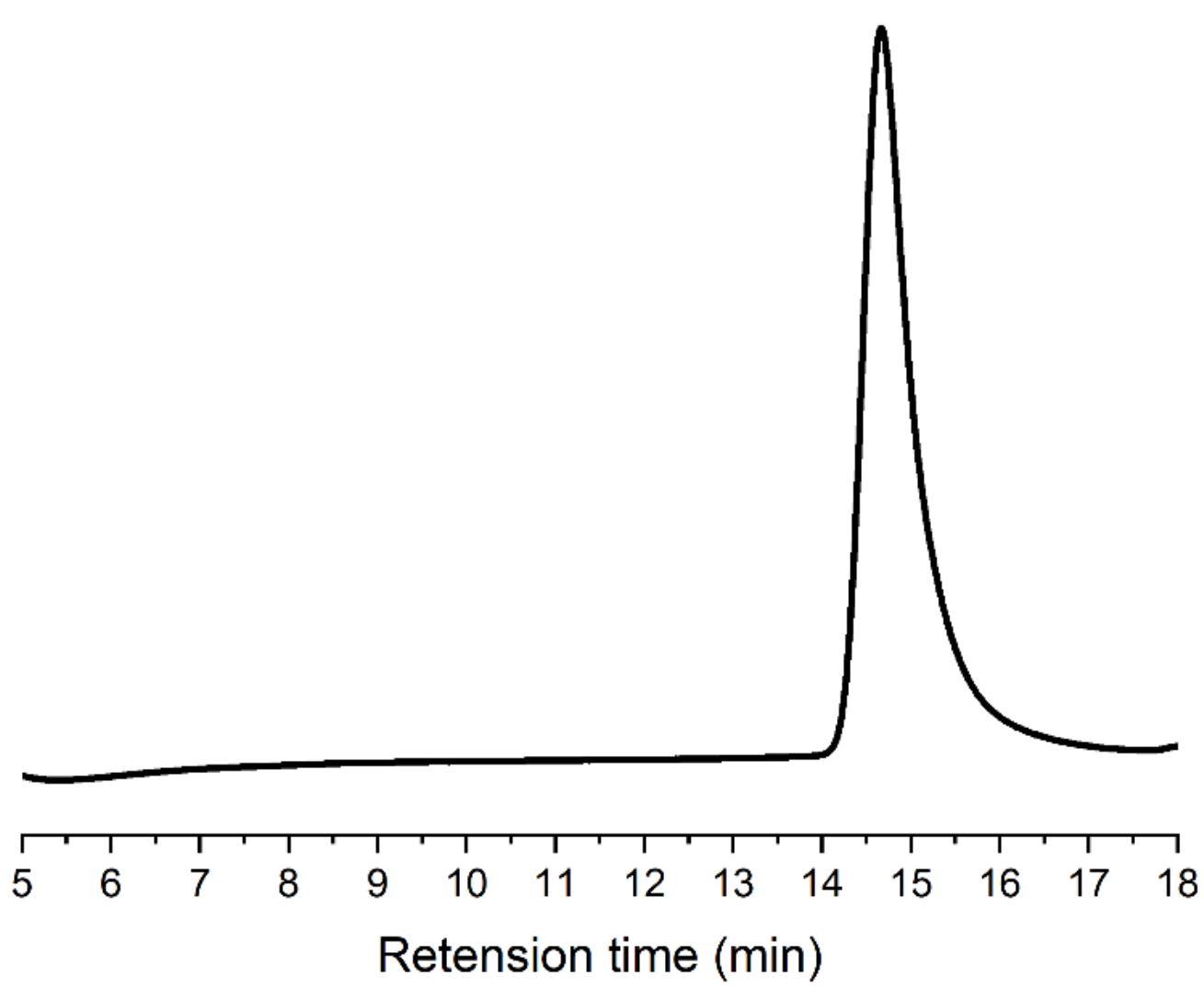

Figure S23. SEC trace of P(BL^{vinyl}) (M_n = 42.6 kDa, $Đ$ = 1.02).

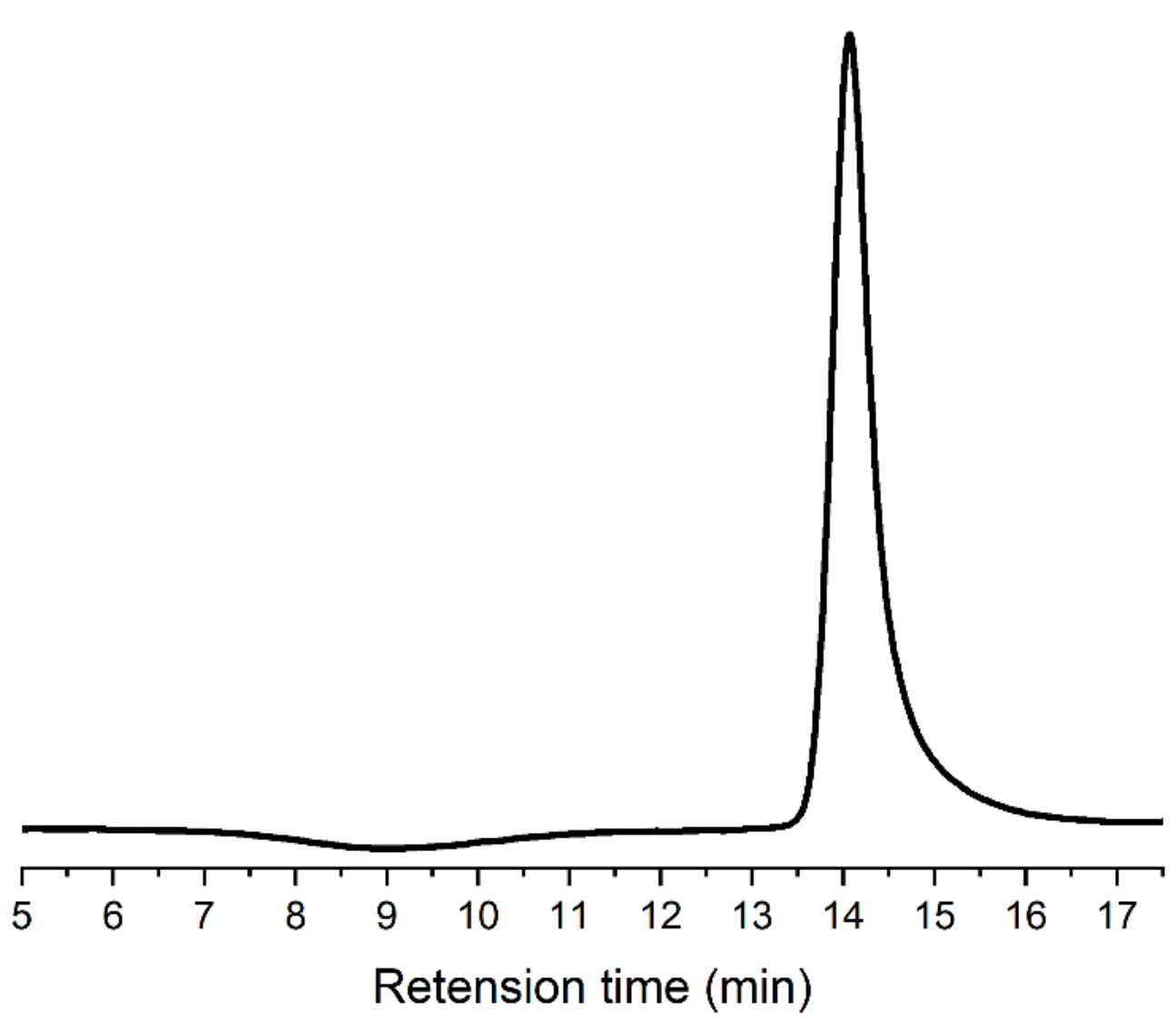

Figure S24. SEC trace of P(BL^{allyl}) (M_n = 118 kDa, $Đ$ = 1.03).

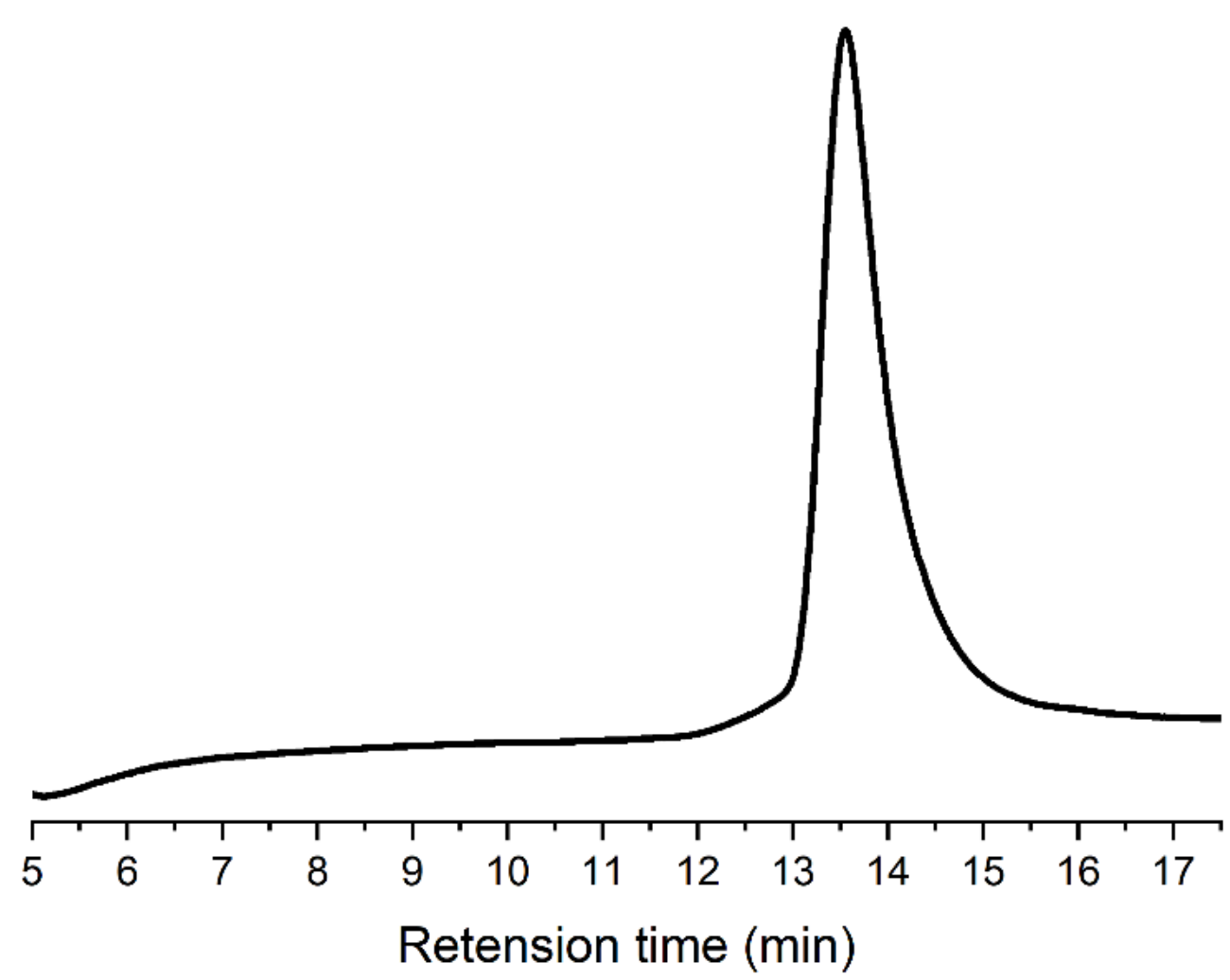

Figure S25. SEC trace of P(BL^{allyl}) (M_n = 245 kDa, $Đ$ = 1.23).

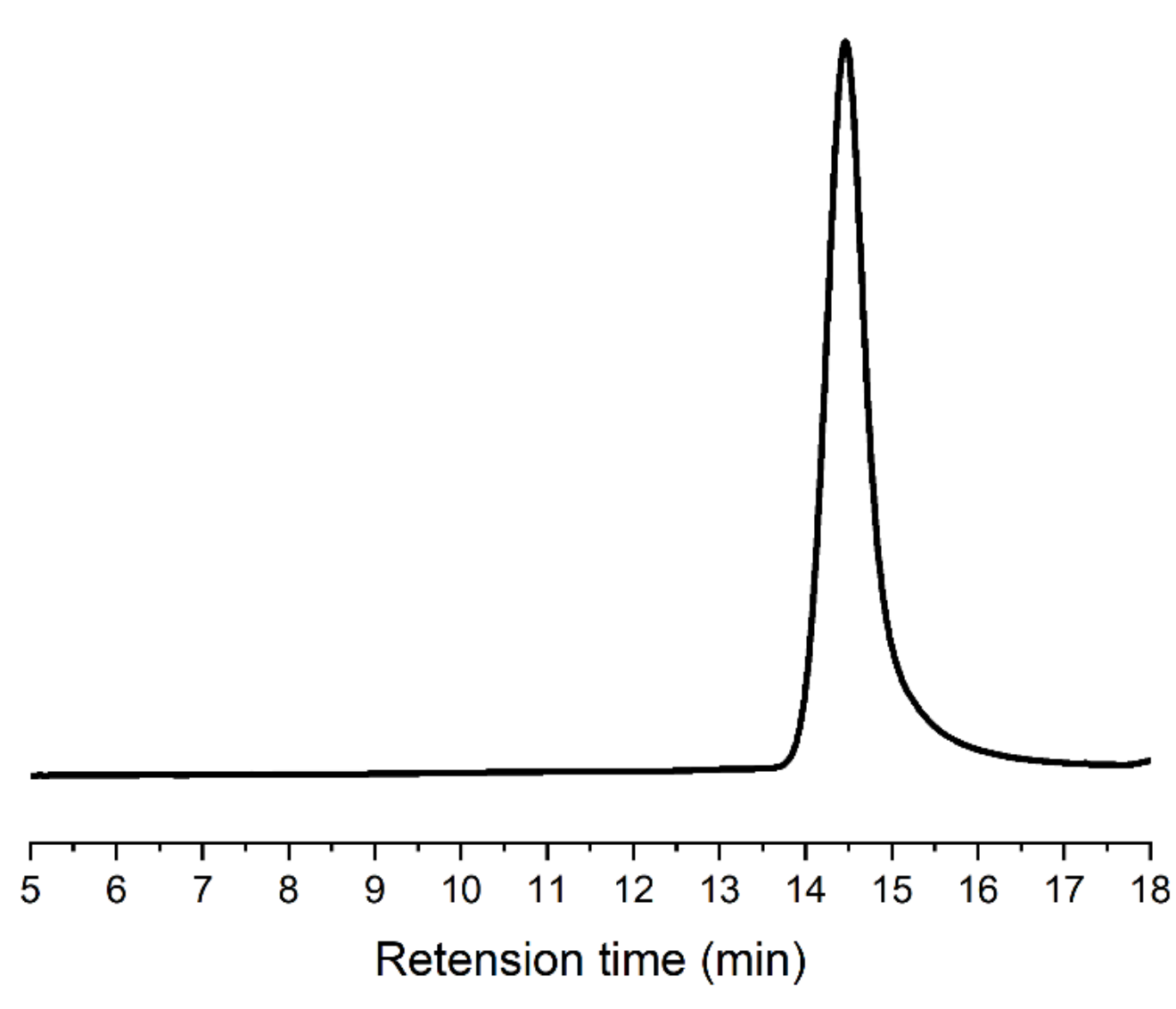

Figure S26. SEC trace of P(BL^{hPg}) (M_n = 72.9 kDa, $Đ$ = 1.03).

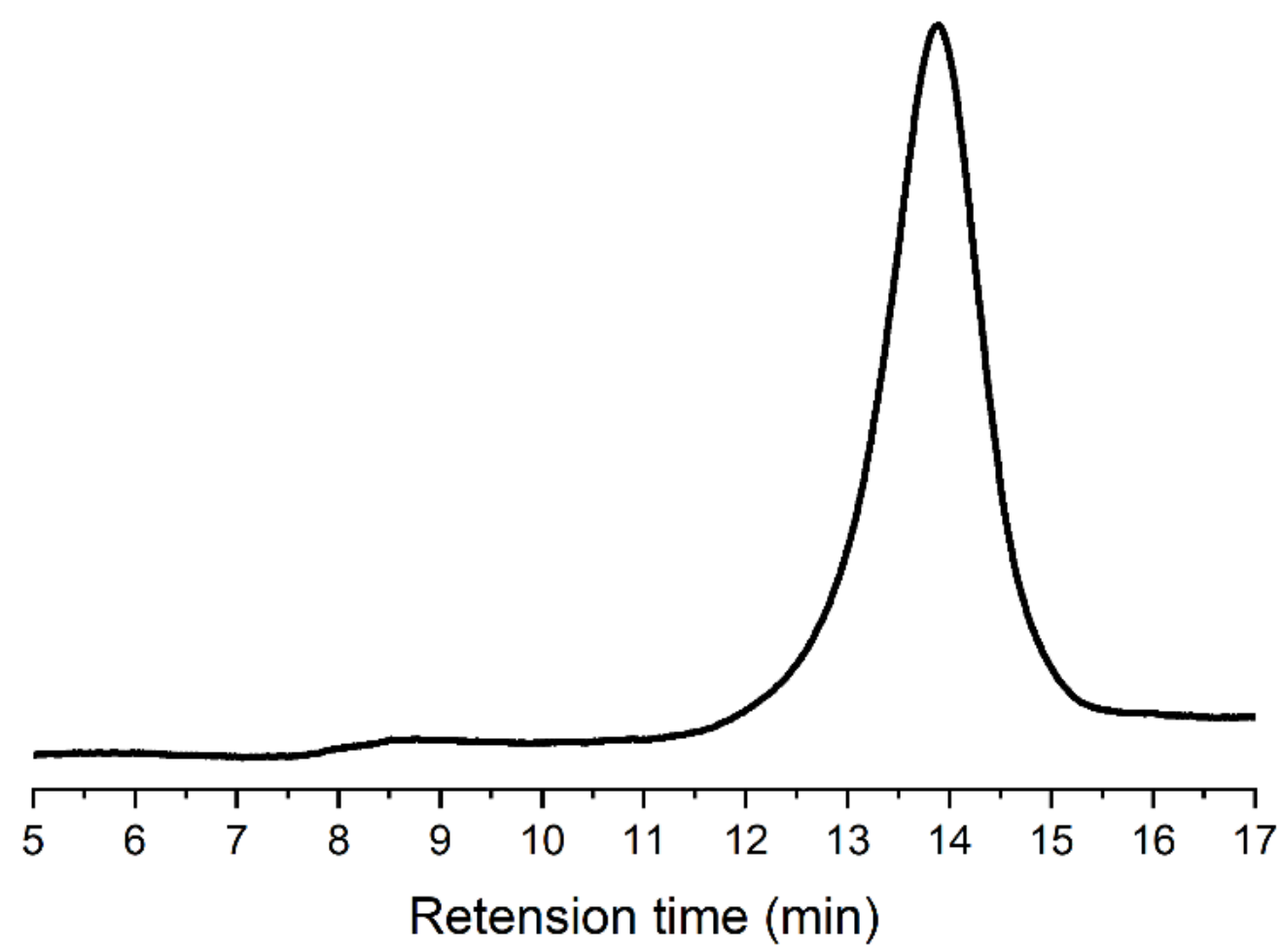

Figure S27. SEC trace of P(BL^{Me}$_{95}$-*co*-BL^{vinyl}$_{5}$) (M_n = 169 kDa, $Đ$ = 1.08).

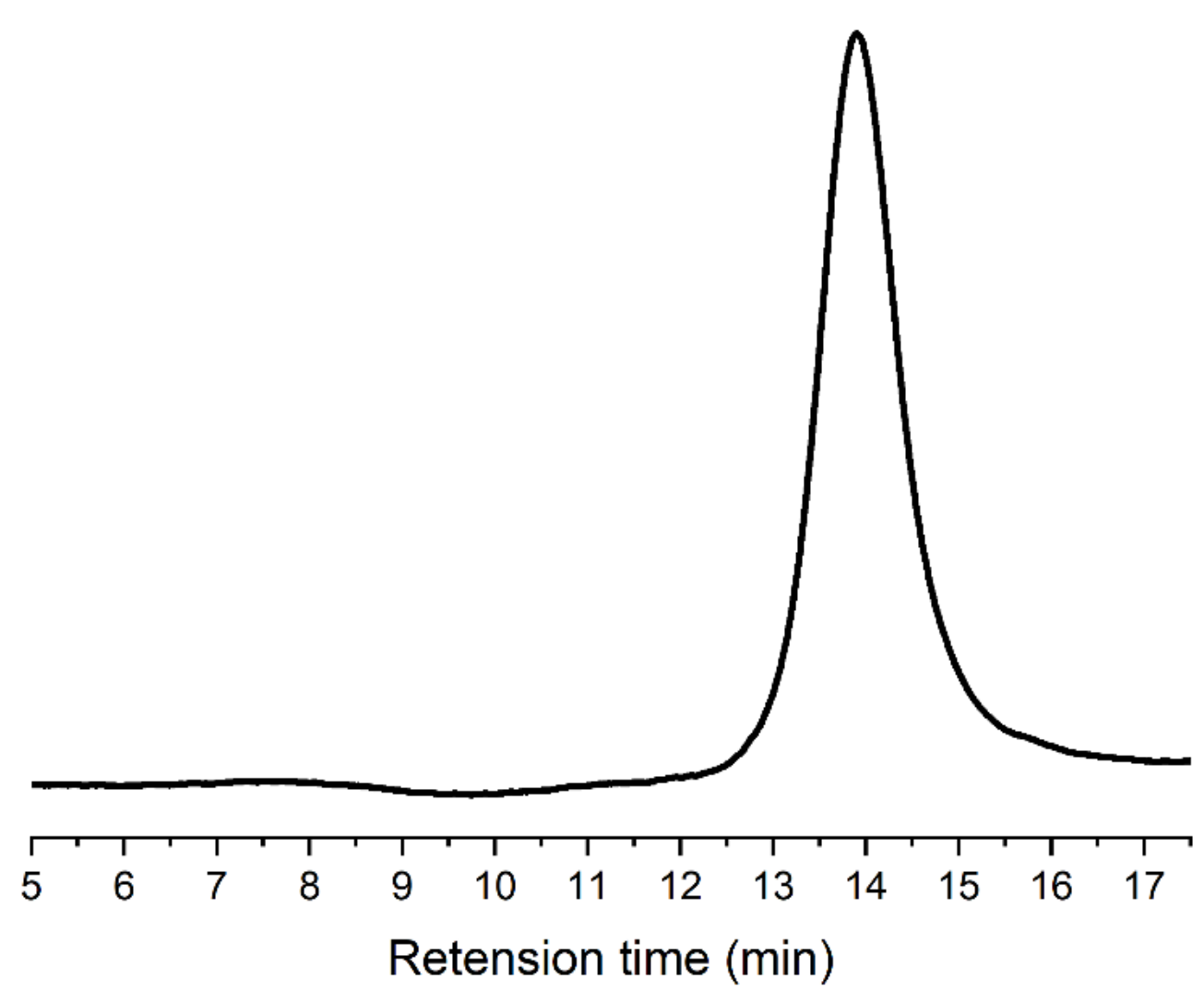

Figure S28. SEC trace of P(BL^{Me}$_{90}$-*co*-BL^{vinyl}$_{10}$) (M_n = 159 kDa, $Đ$ = 1.05).

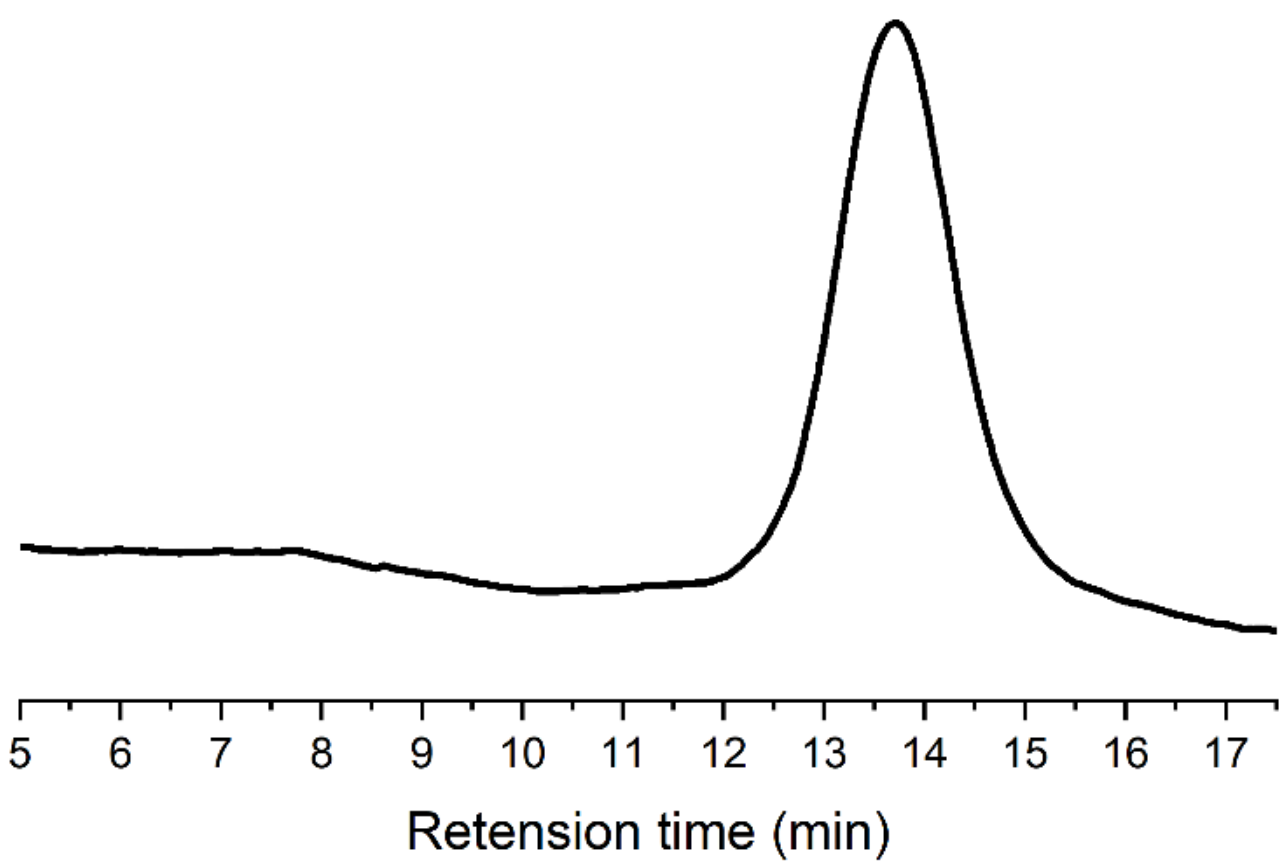

Figure S29. SEC trace of $P(BL^{Me}_{96}$-*co*-$BL^{allyl}_{4})$ (M_n = 161 kDa, $Đ$ = 1.13).

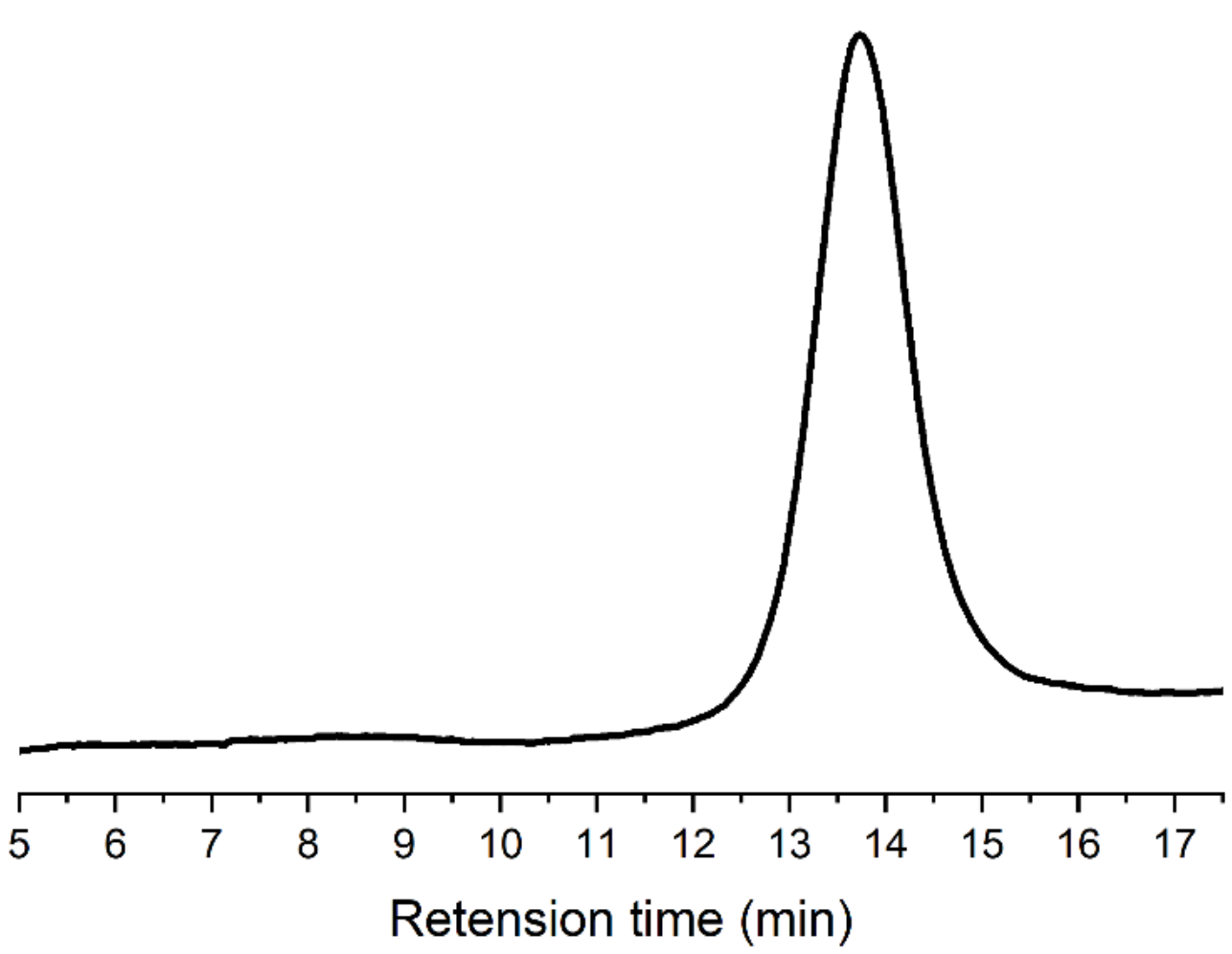

Figure S30. SEC trace of $P(BL^{Me}_{92}$-*co*-$BL^{allyl}_{8})$ (M_n = 202 kDa, $Đ$ = 1.05).

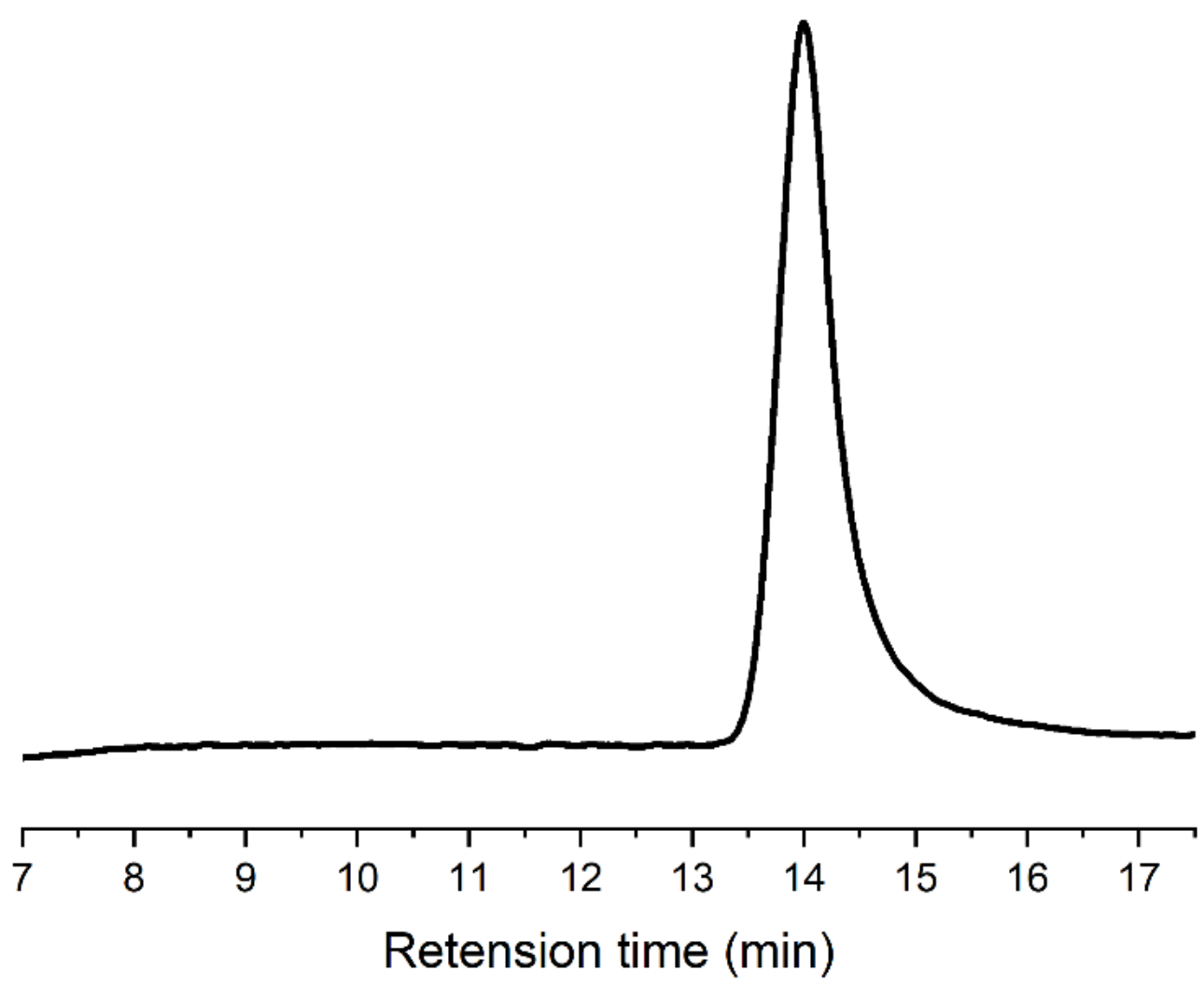

Figure S31. SEC trace of P(BL^{Me}_{92}-*co*-BL^{allyl}_{8}) (M_n = 121 kDa, $Đ$ = 1.01).

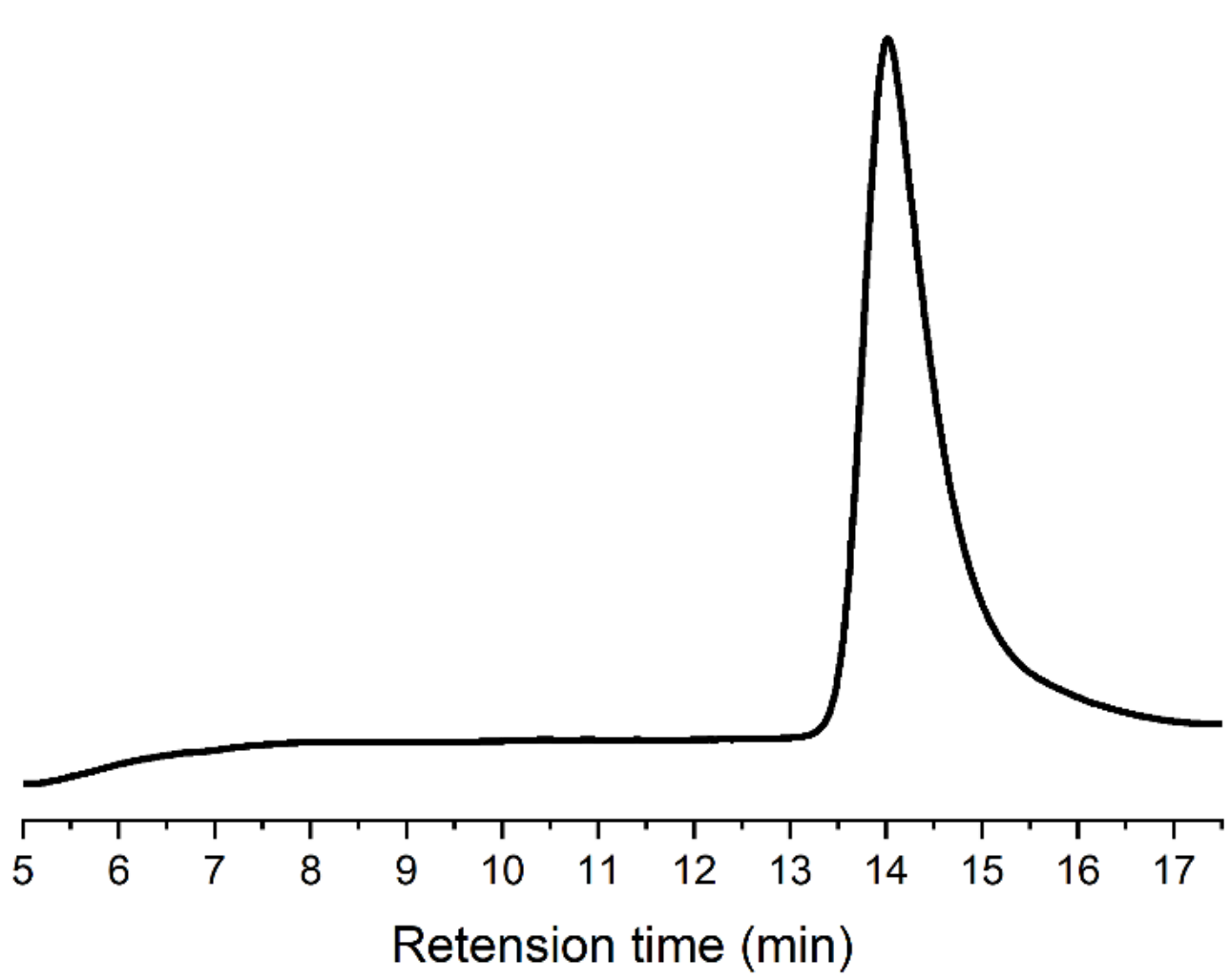

Figure S32. SEC trace of P(BL^{Me}_{95}-*co*-BL^{hPg}_{5}) (M_n = 90.5 kDa, $Đ$ = 1.08).

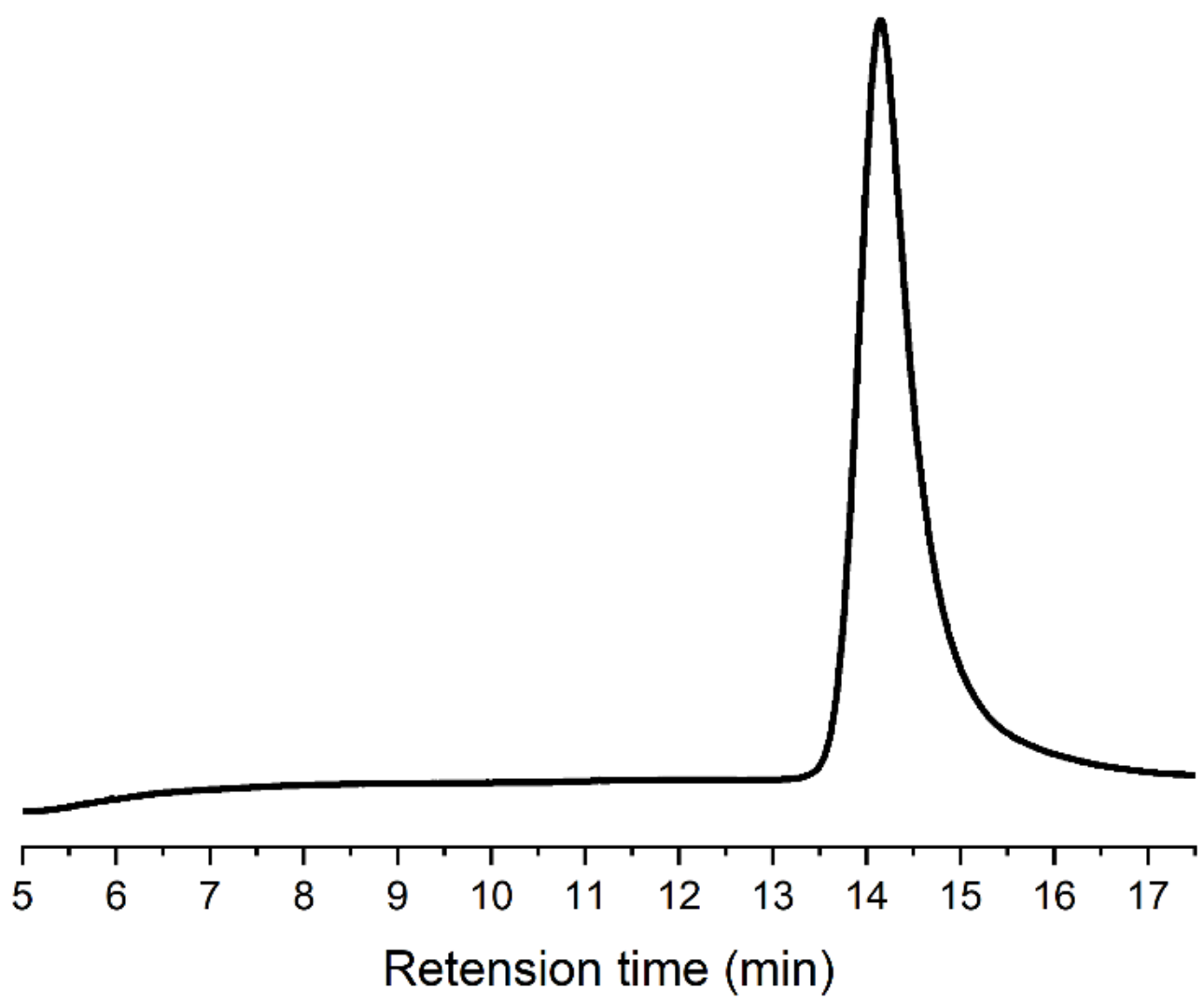

Figure S33. SEC trace of P($BL^{Me}{}_{90}$-*co*-$BL^{hPg}{}_{10}$) (M_n = 79.8 kDa, $Đ$ = 1.07).

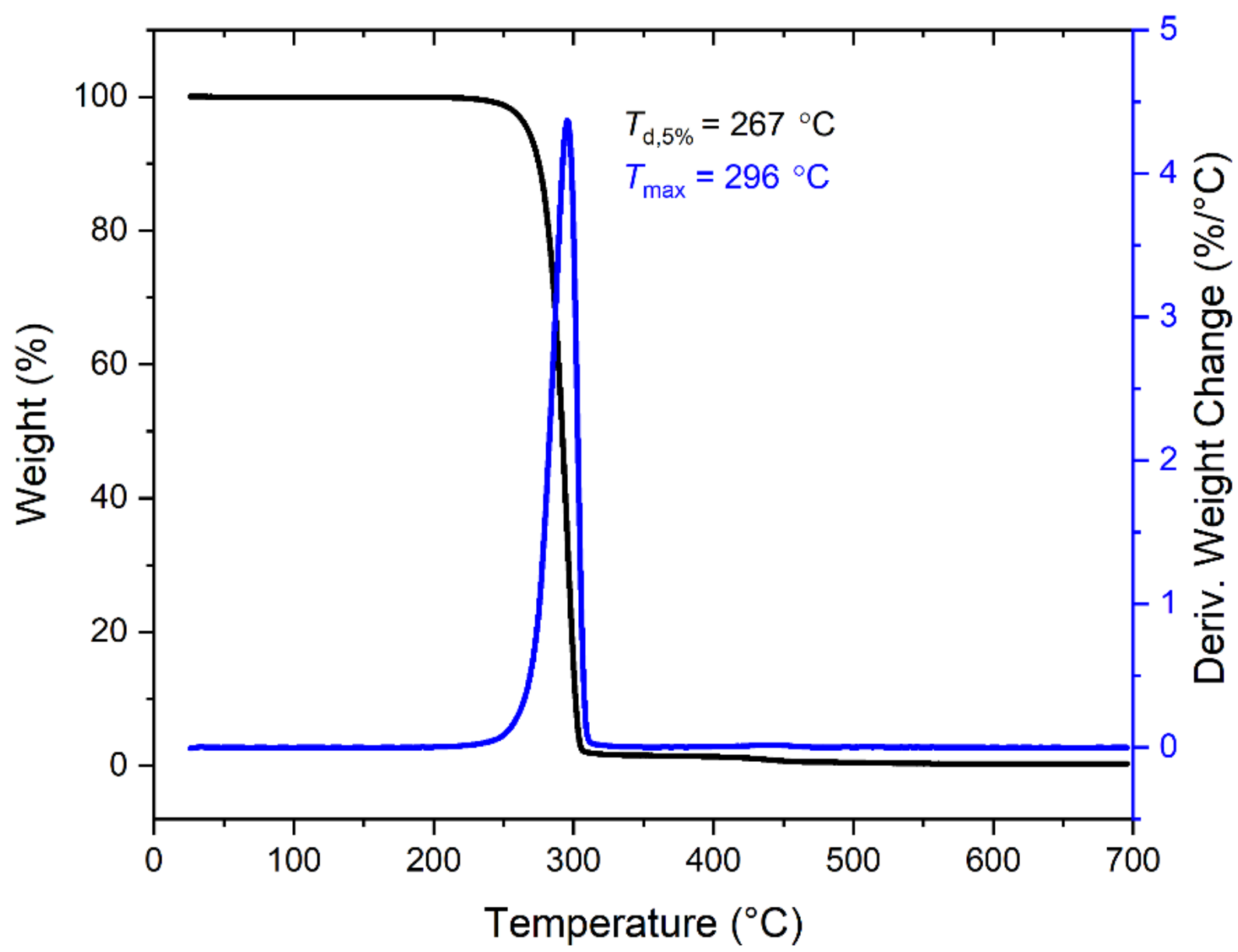

Figure S34. TGA and DTG curves of P(BL^{vinyl}) (M_n = 42.6 kDa, $Đ$ = 1.02).

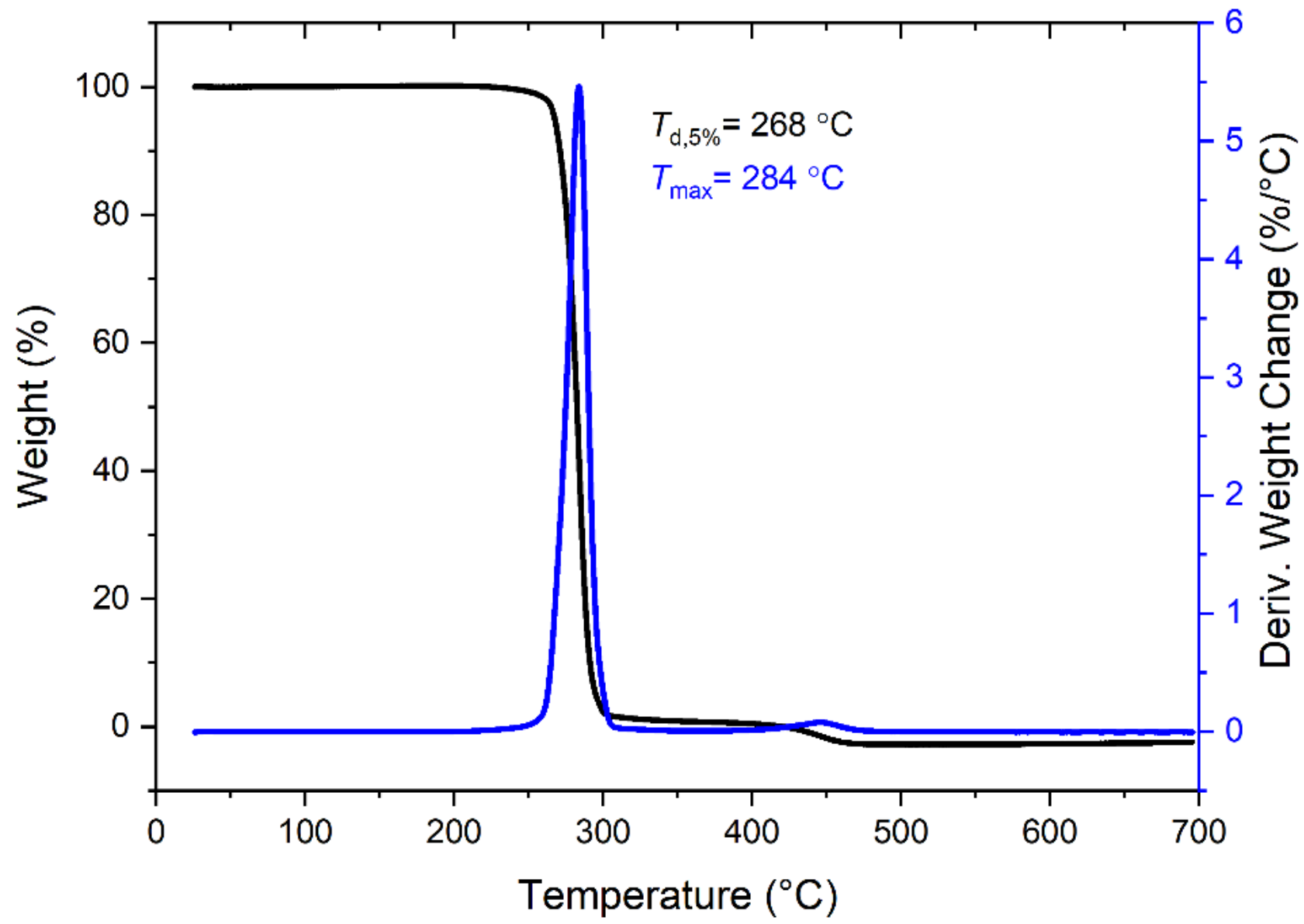

Figure S35. TGA and DTG curves of P(BL^{allyl}) (M_n = 103 kDa, $Đ$ = 1.08).

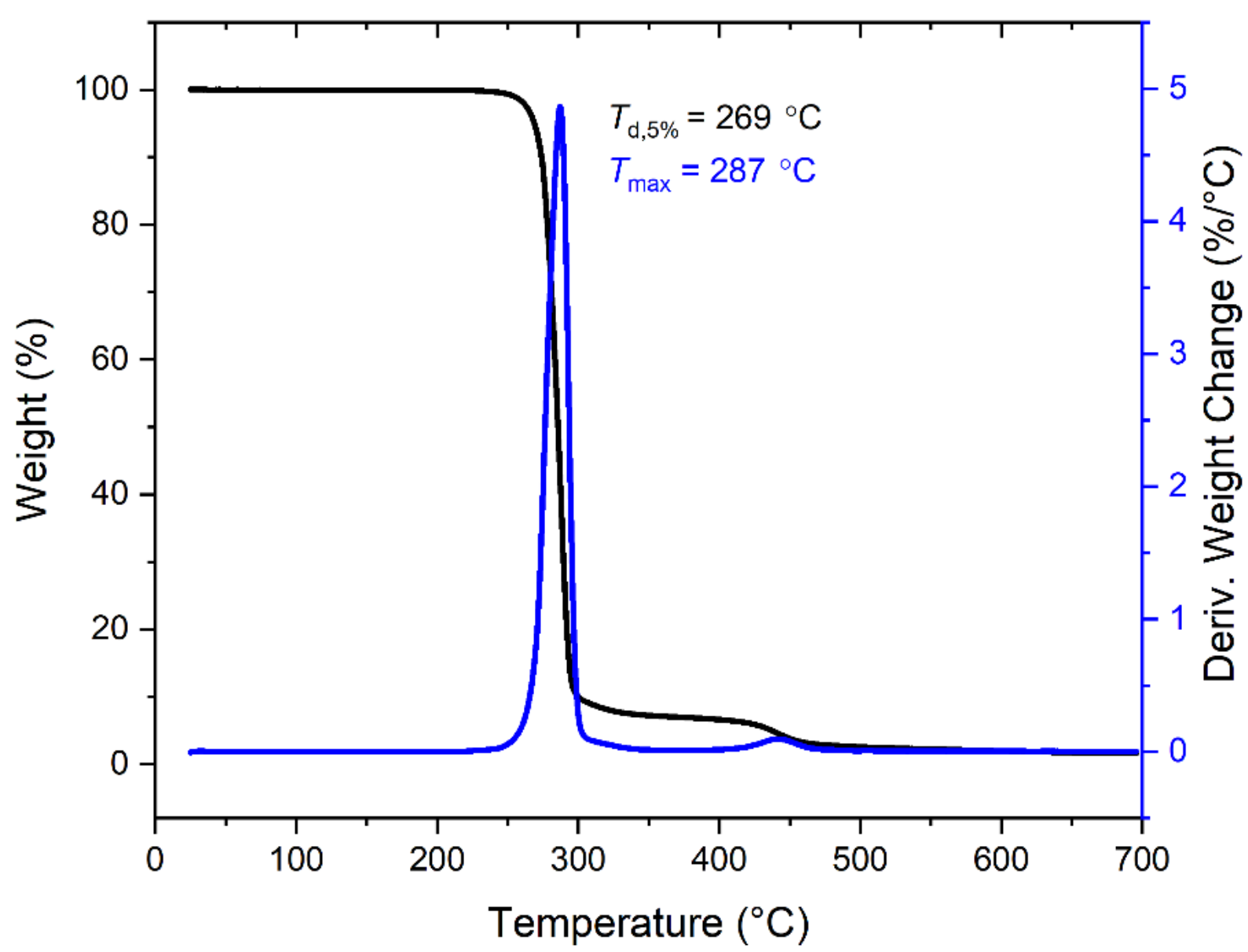

Figure S36. TGA and DTG curves of P(BL^{hPg}) (M_n = 76.9 kDa, $Đ$ = 1.03).

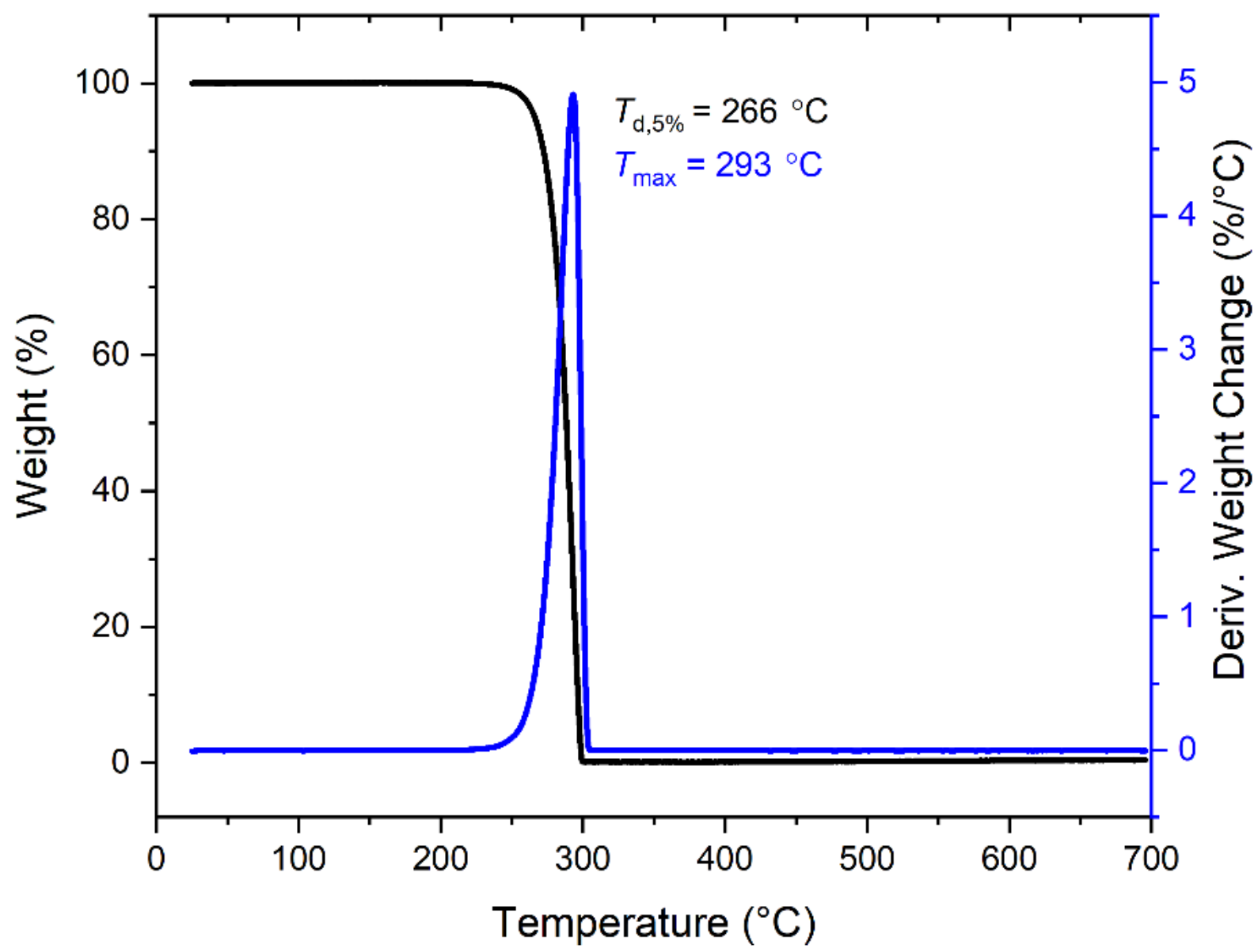

Figure S37. TGA and DTG curves of P(BL^{Me}$_{95}$-*co*-BL^{vinyl}$_{5}$) (M_n = 169 kDa, $Đ$ = 1.08).

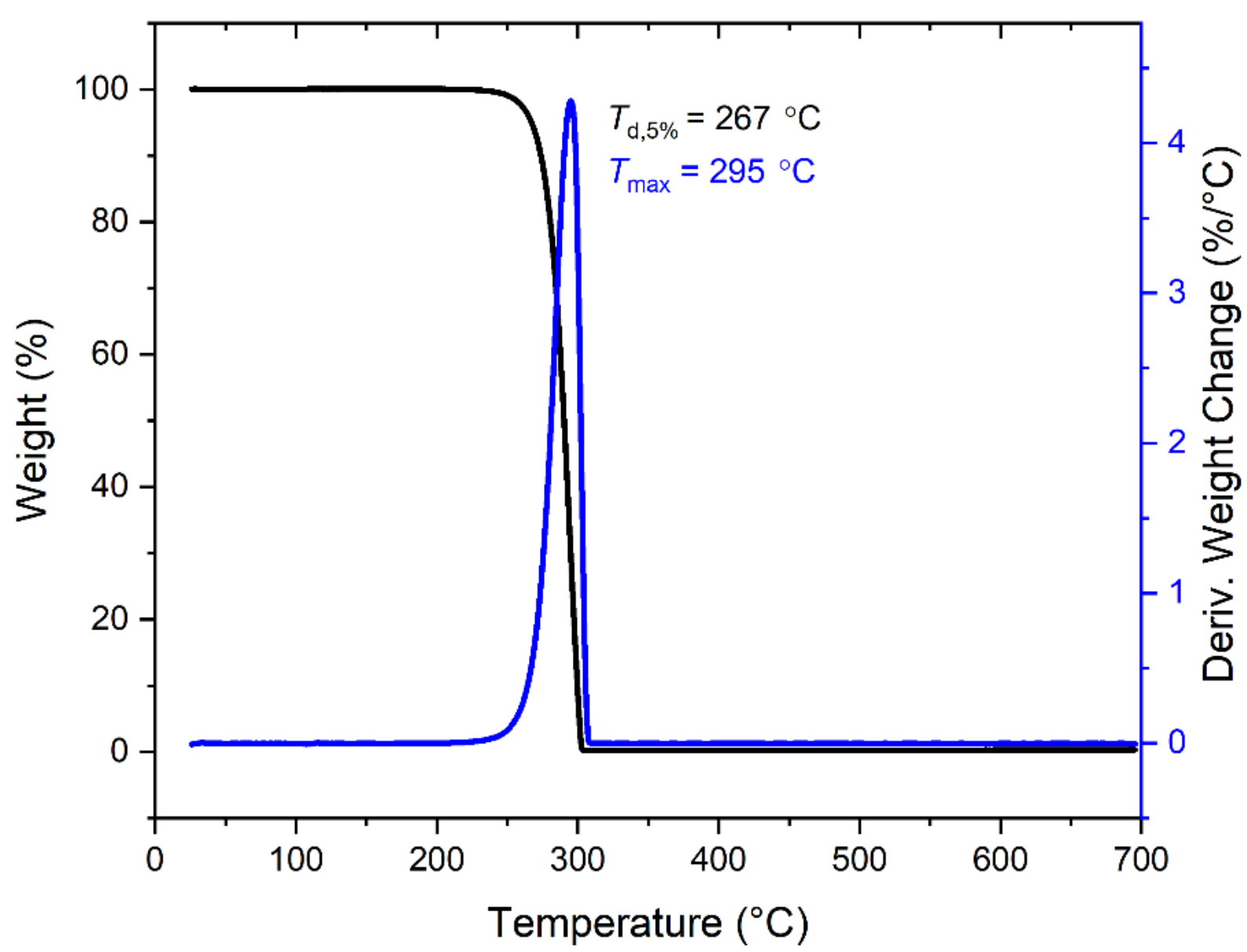

Figure S38. TGA and DTG curves of P(BL^{Me}$_{90}$-*co*-BL^{vinyl}$_{10}$) (M_n = 159 kDa, $Đ$ = 1.05).

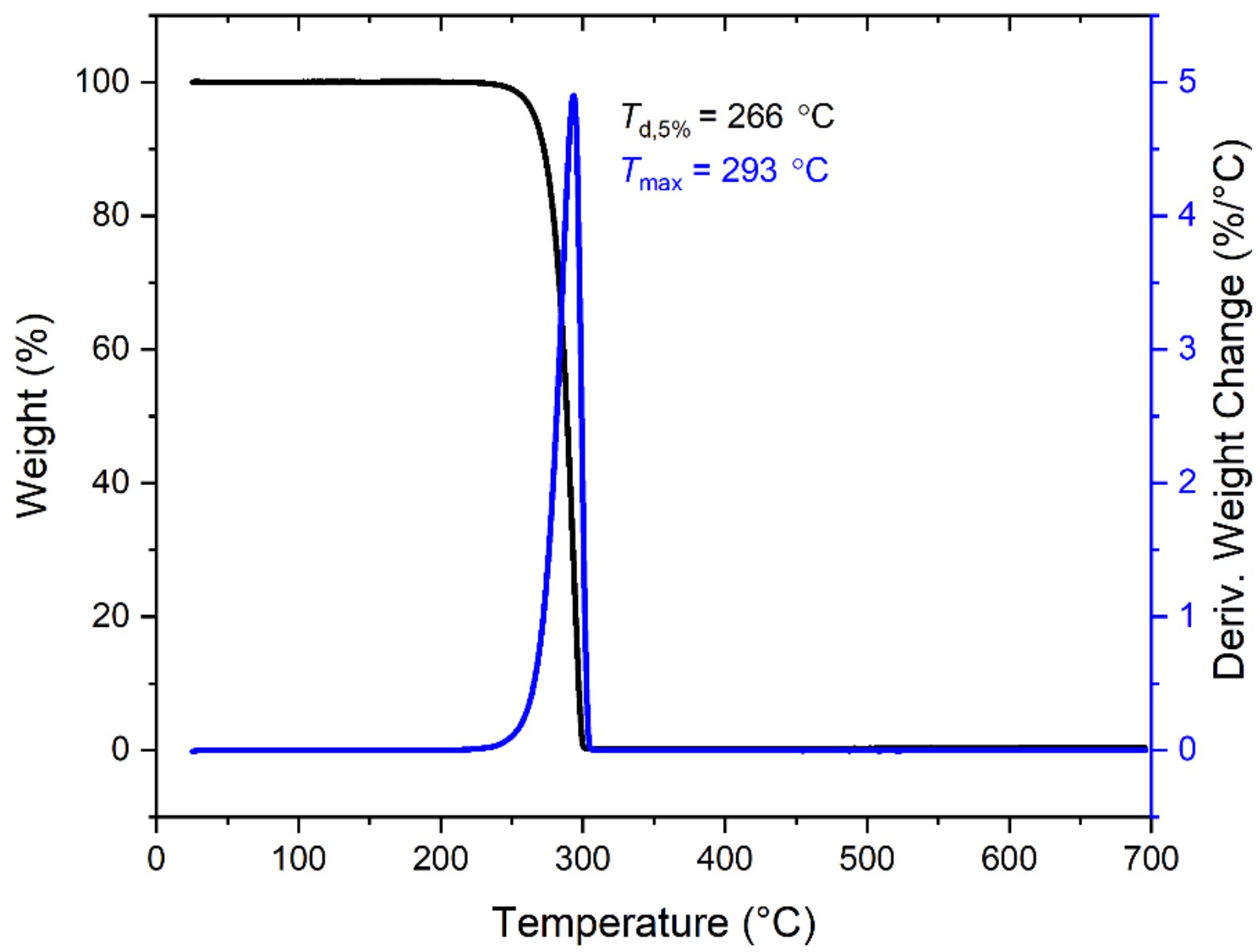

Figure S39. TGA and DTG curves of P(BL$^{Me}{}_{96}$-*co*-BL$^{allyl}{}_{4}$) (M_n = 161 kDa, $Đ$ = 1.13).

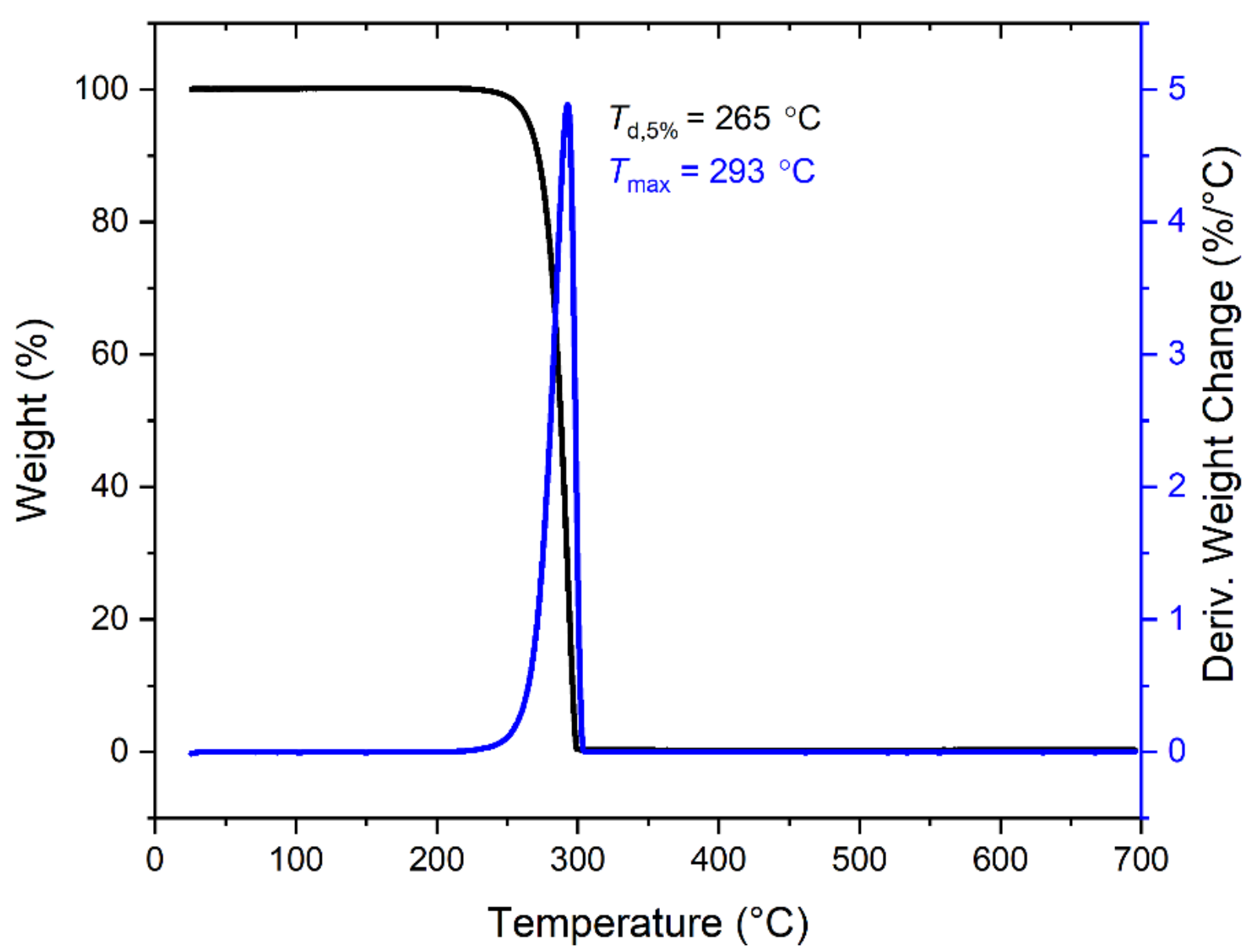

Figure S40. TGA and DTG curves of P(BL$^{Me}{}_{92}$-*co*-BL$^{allyl}{}_{8}$) (M_n = 202 kDa, $Đ$ = 1.05).

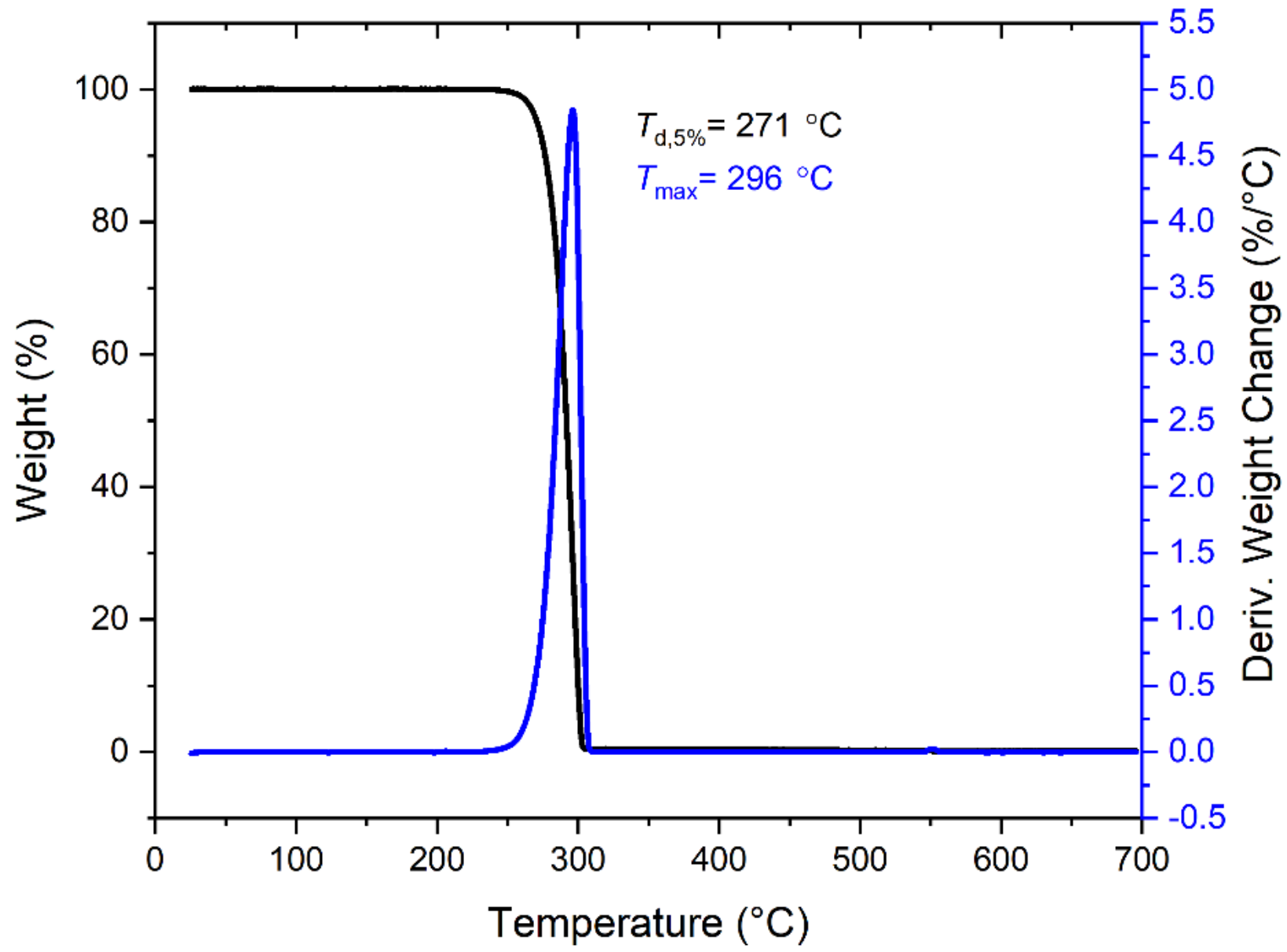

Figure S41. TGA and DTG curves of P(BL^{Me}$_{95}$-*co*-BL^{hPg}$_{5}$) (M_n = 90.5 kDa, $Đ$ = 1.08).

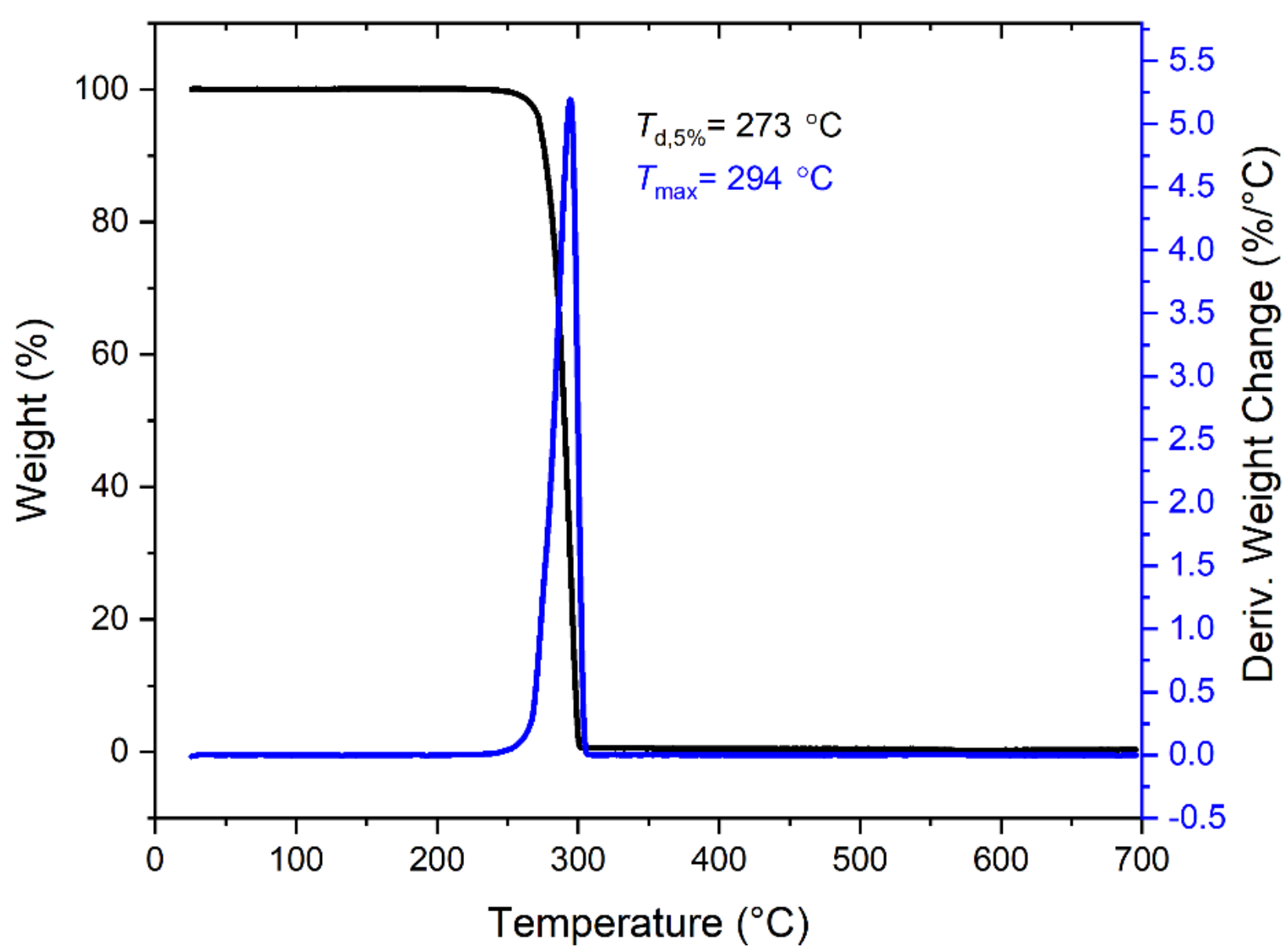

Figure S42. TGA and DTG curves of P(BL^{Me}$_{90}$-*co*-BL^{hPg}$_{10}$) (M_n = 79.8 kDa, $Đ$ = 1.07).

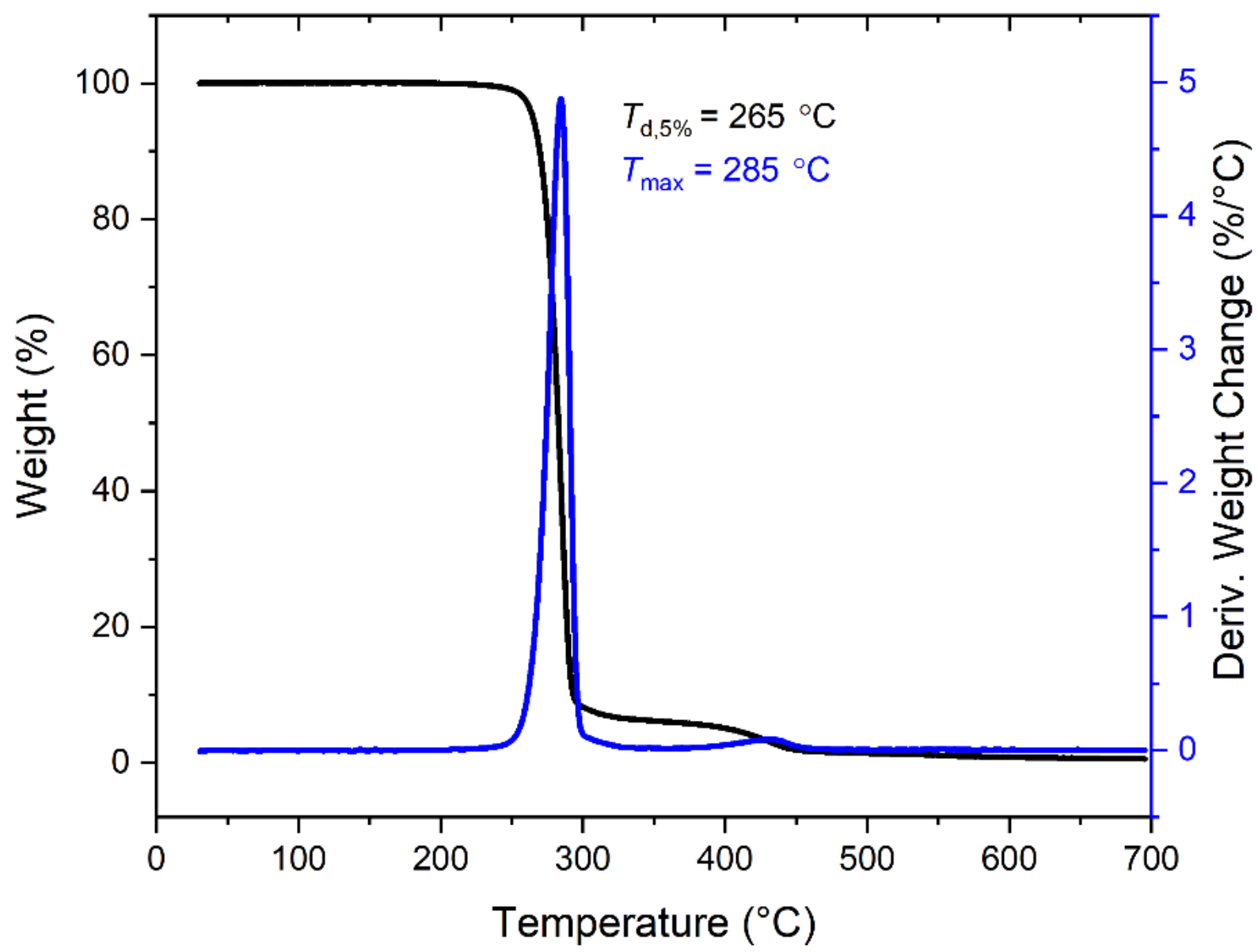

Figure S43. TGA and DTG curves of P(BL^{vinyl})×2%.

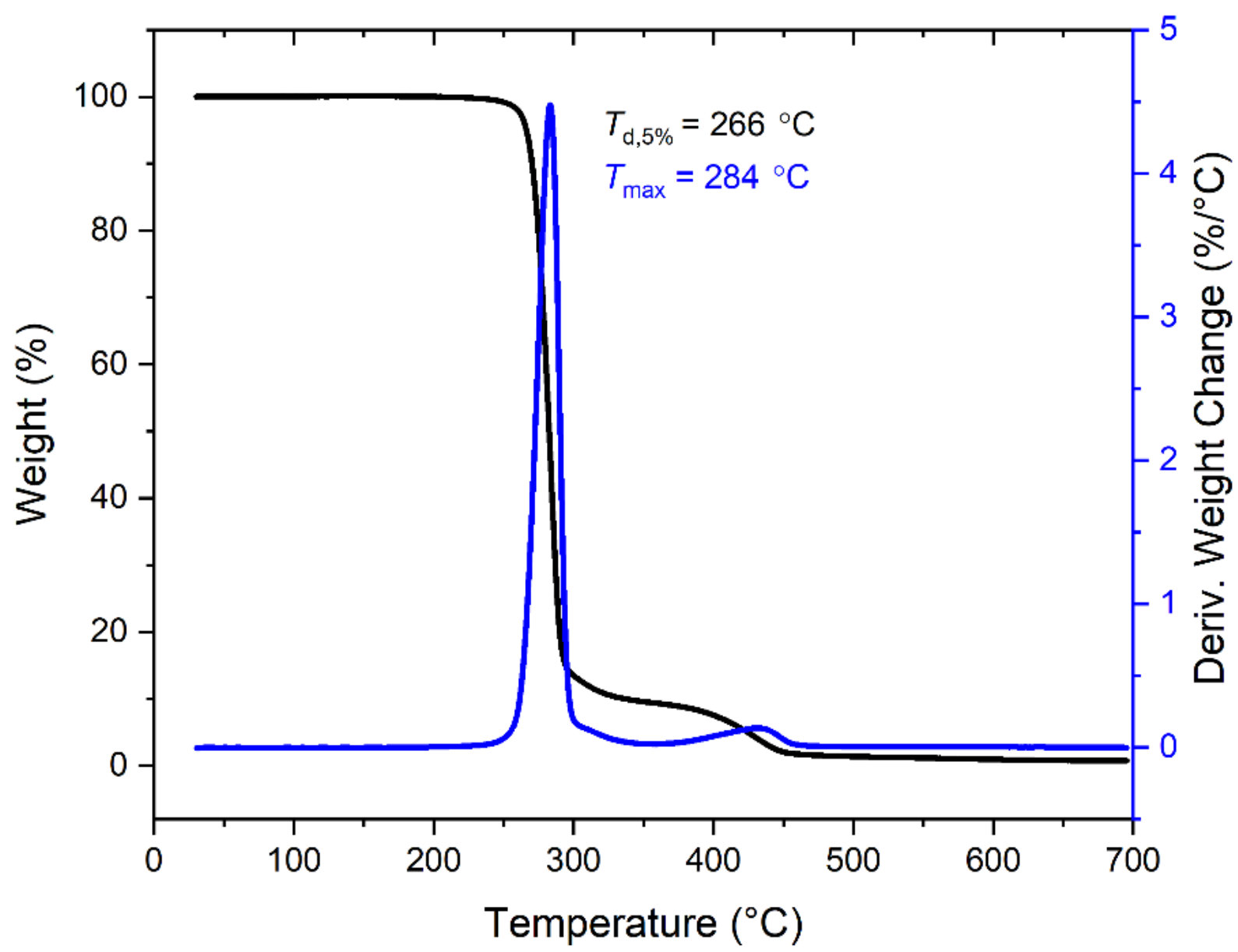

Figure S44. TGA and DTG curves of P(BL^{vinyl})×5%.

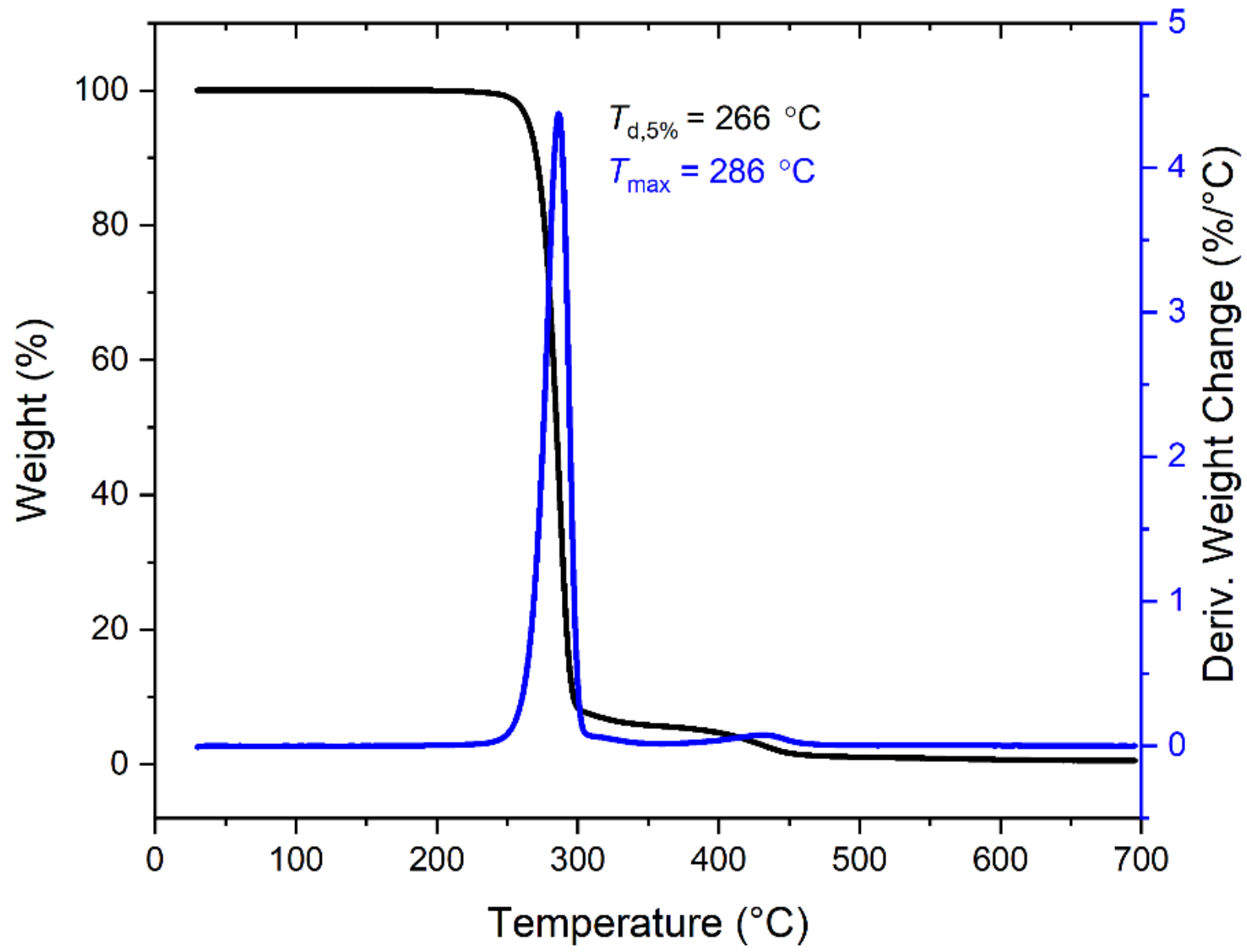

Figure S45. TGA and DTG curves of P(BL^{allyl})×2%.

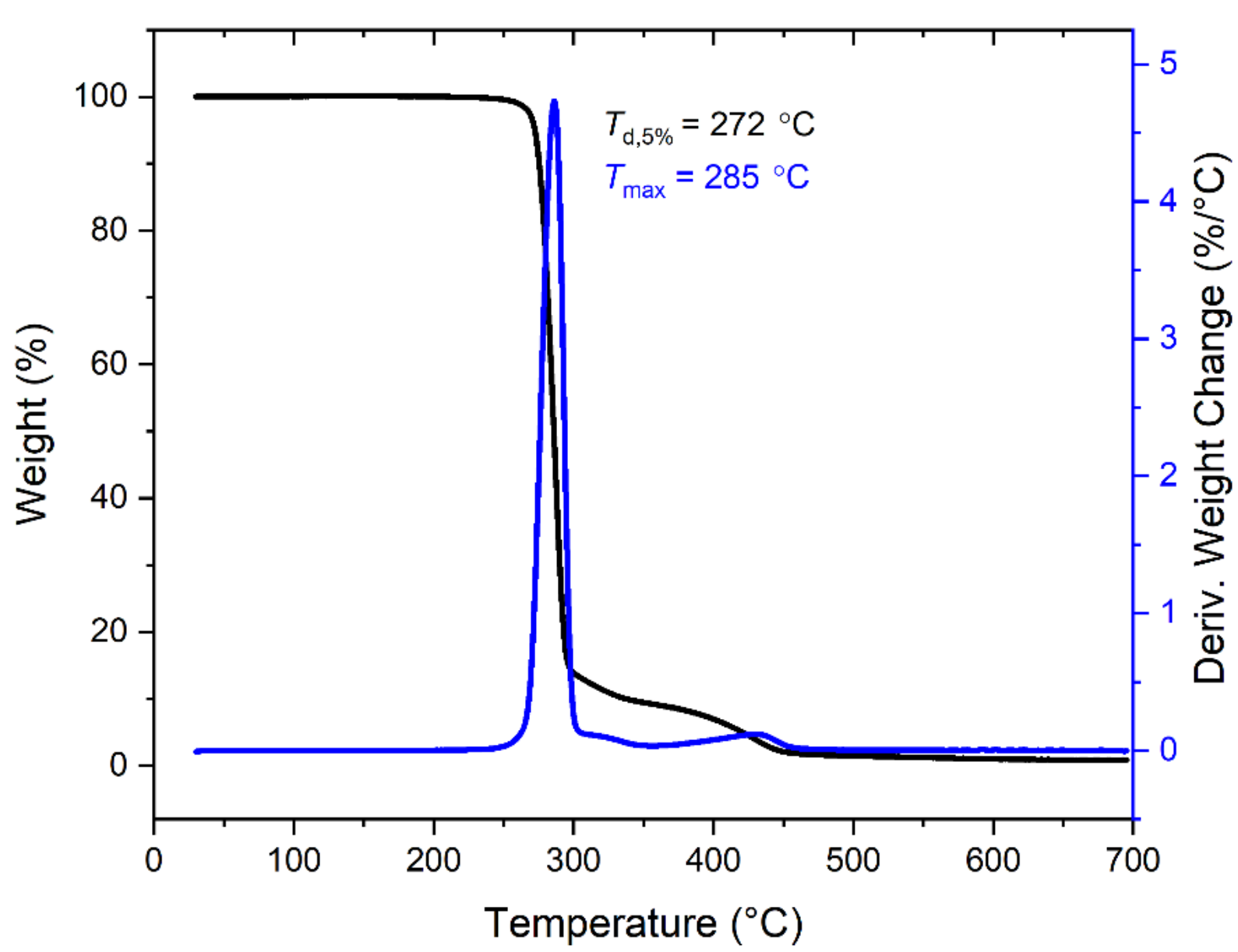

Figure S46. TGA and DTG curves of P(BL^{allyl})×5%.

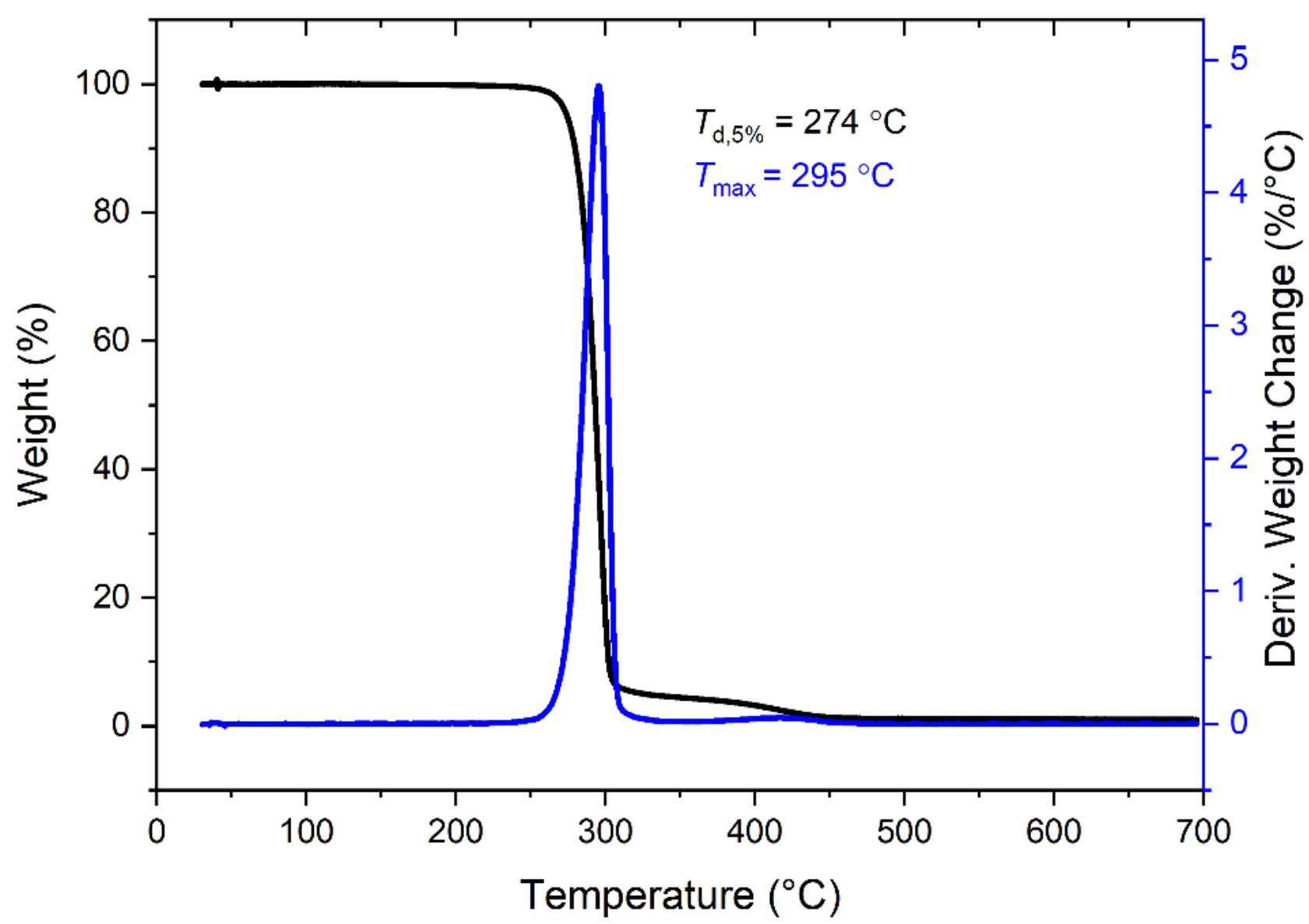

Figure S47. TGA and DTG curves of P(BL^{Me}$_{96}$-*co*-BL^{vinyl}$_{4}$)-PETMP.

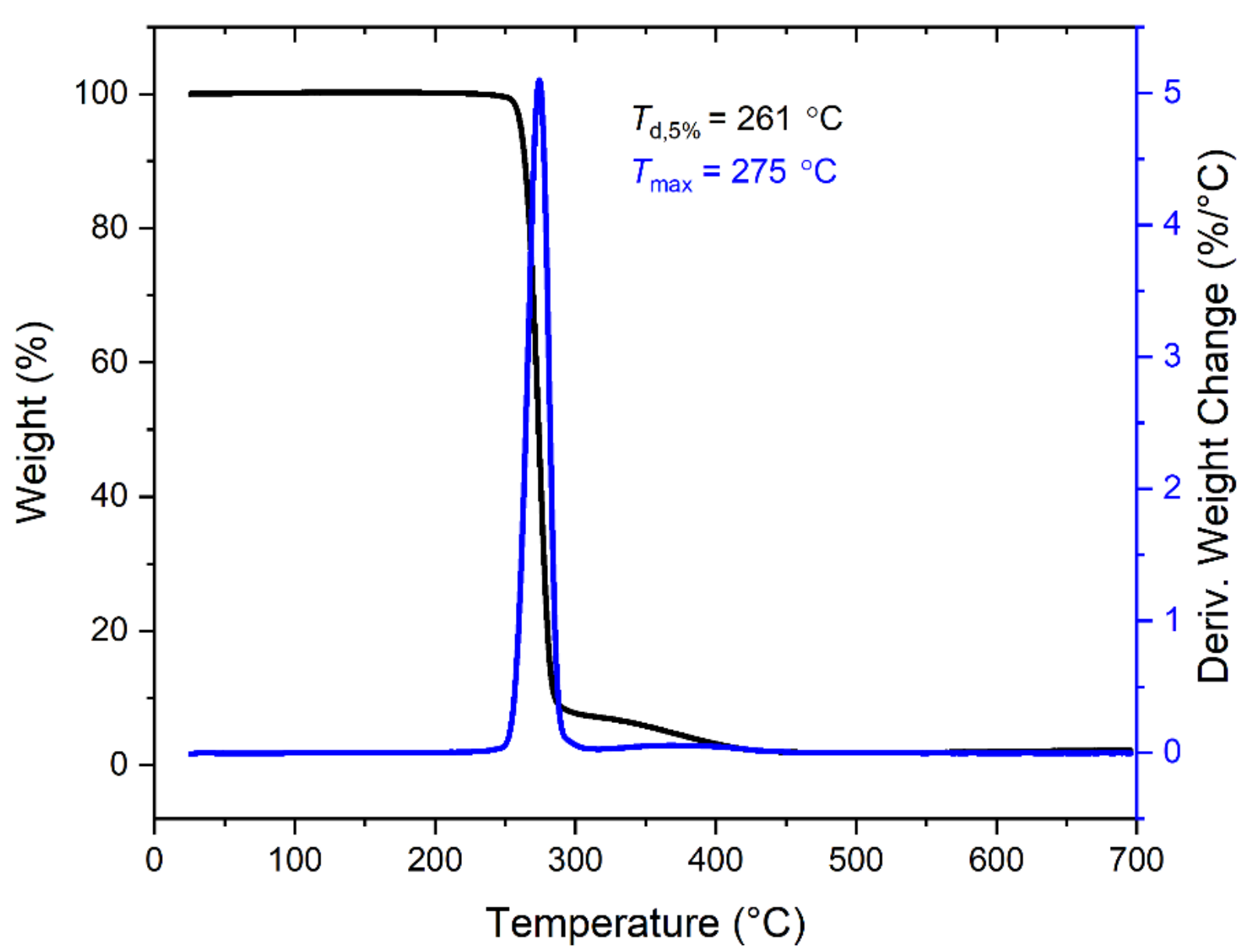

Figure S48. TGA and DTG curves of P(BL^{Me}$_{96}$-*co*-BL^{allyl}$_{4}$)-PETMP.

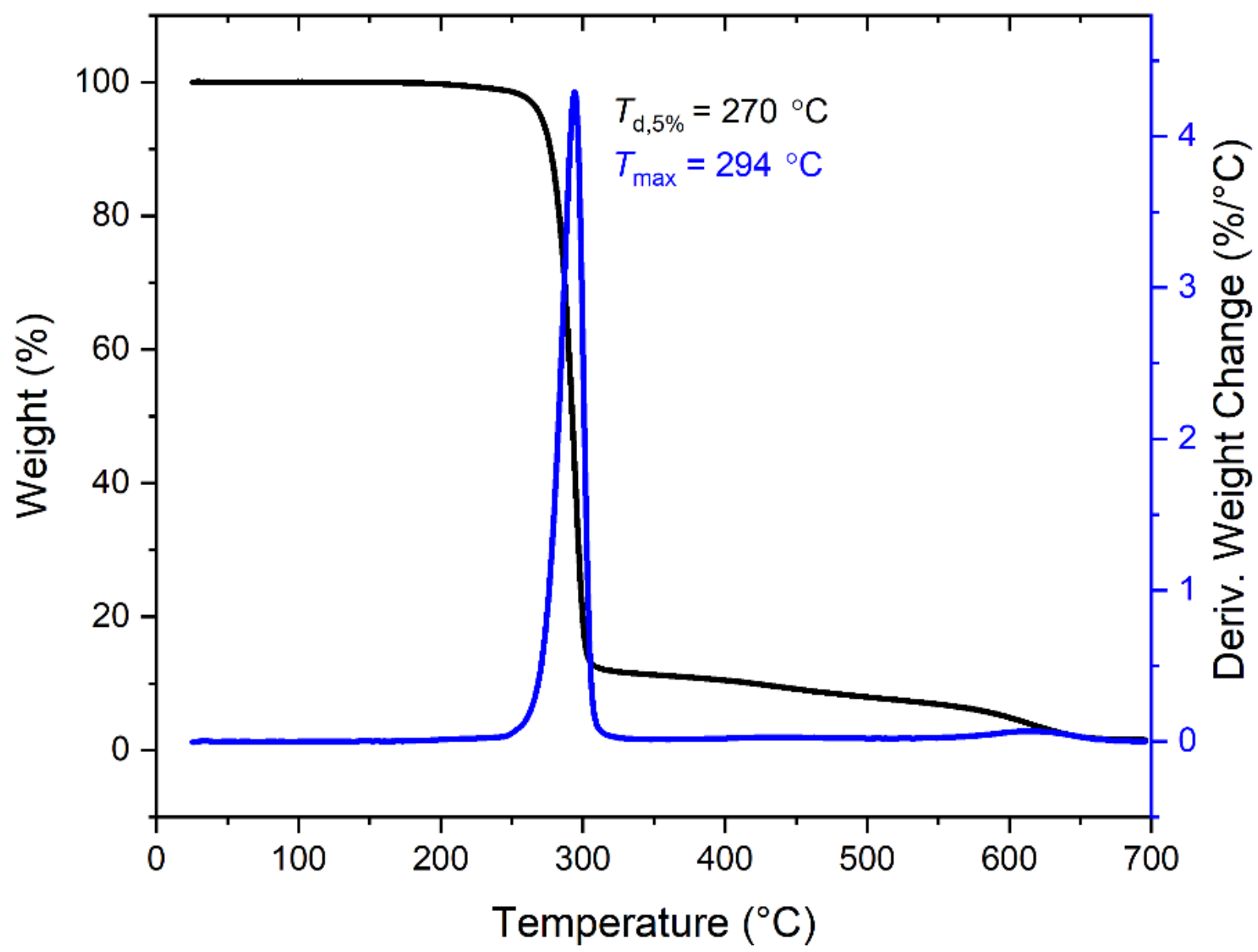

Figure S49. TGA and DTG curves of P(BL^{Me}$_{90}$-*co*-BL^{allyl}$_{10}$)-UPy.

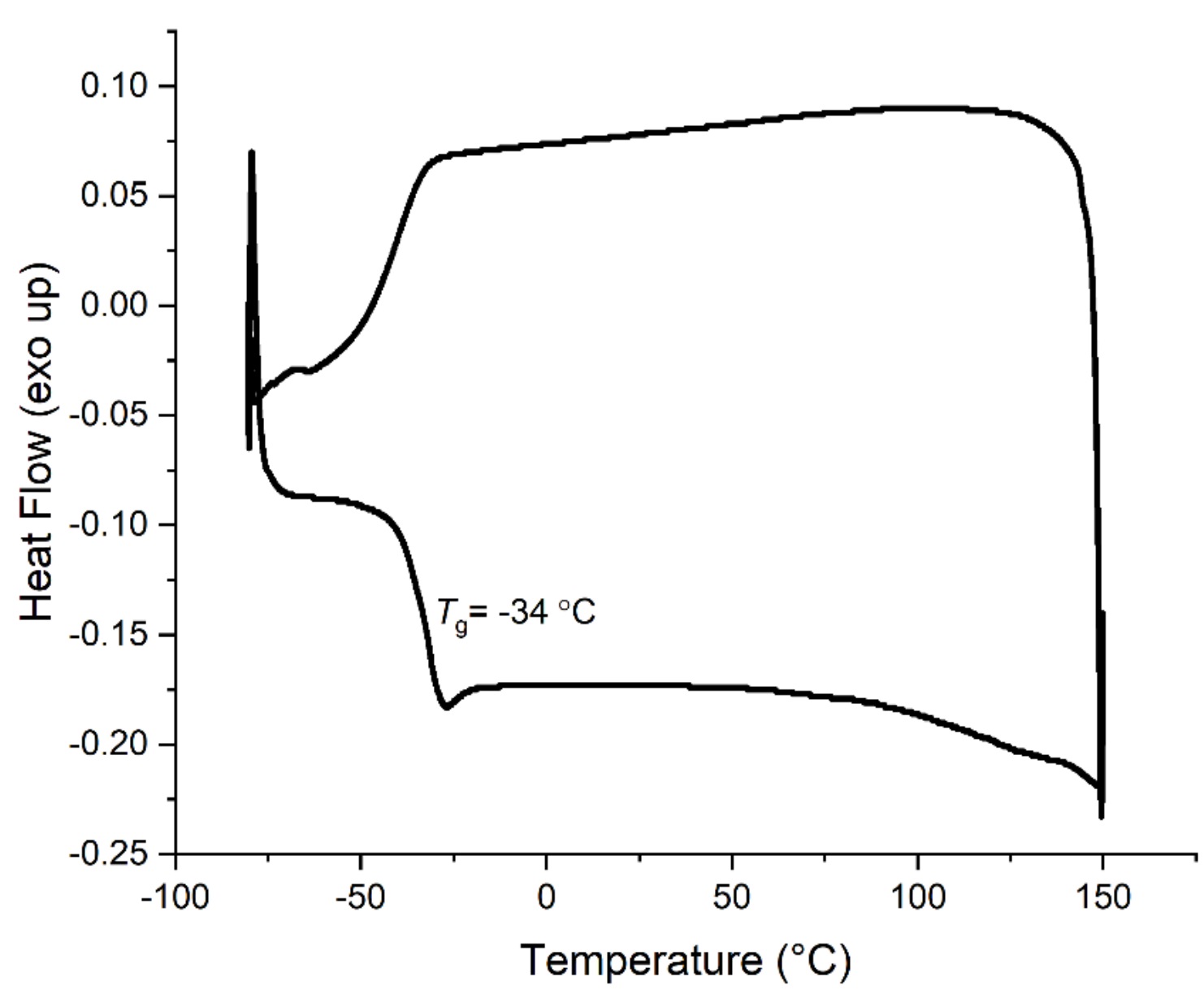

Figure S50. DSC curves of 1st cooling and 2nd heating scans of atactic P(BL^{vinyl}) (M_n = 3.57 kDa, $Đ$ = 1.12) prepared with [*rac*-BL^{vinyl}]/[^{t}Bu-P$_4$]/[BnOH] = 100:1:1. Scan rate: 10 °C/min.

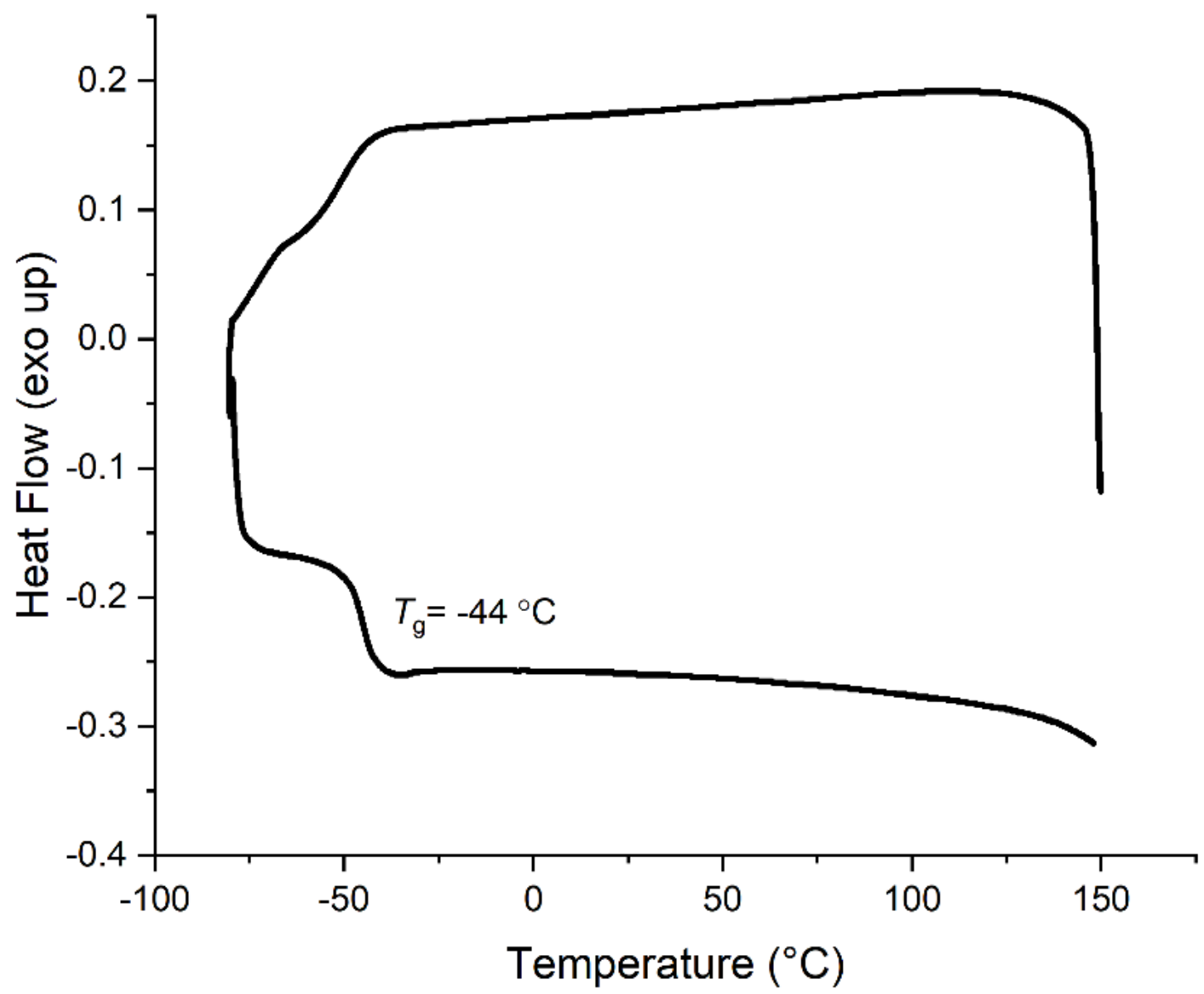

Figure S51. DSC curves of 1st cooling and 2nd heating scans of atactic P(BL^{allyl}) (M_n = 7.03 kDa, $Đ$ = 1.17) prepared with [*rac*-BL^{allyl}]/[^{t}Bu-P$_4$]/[BnOH] = 100:1:1. Scan rate: 10 °C/min.

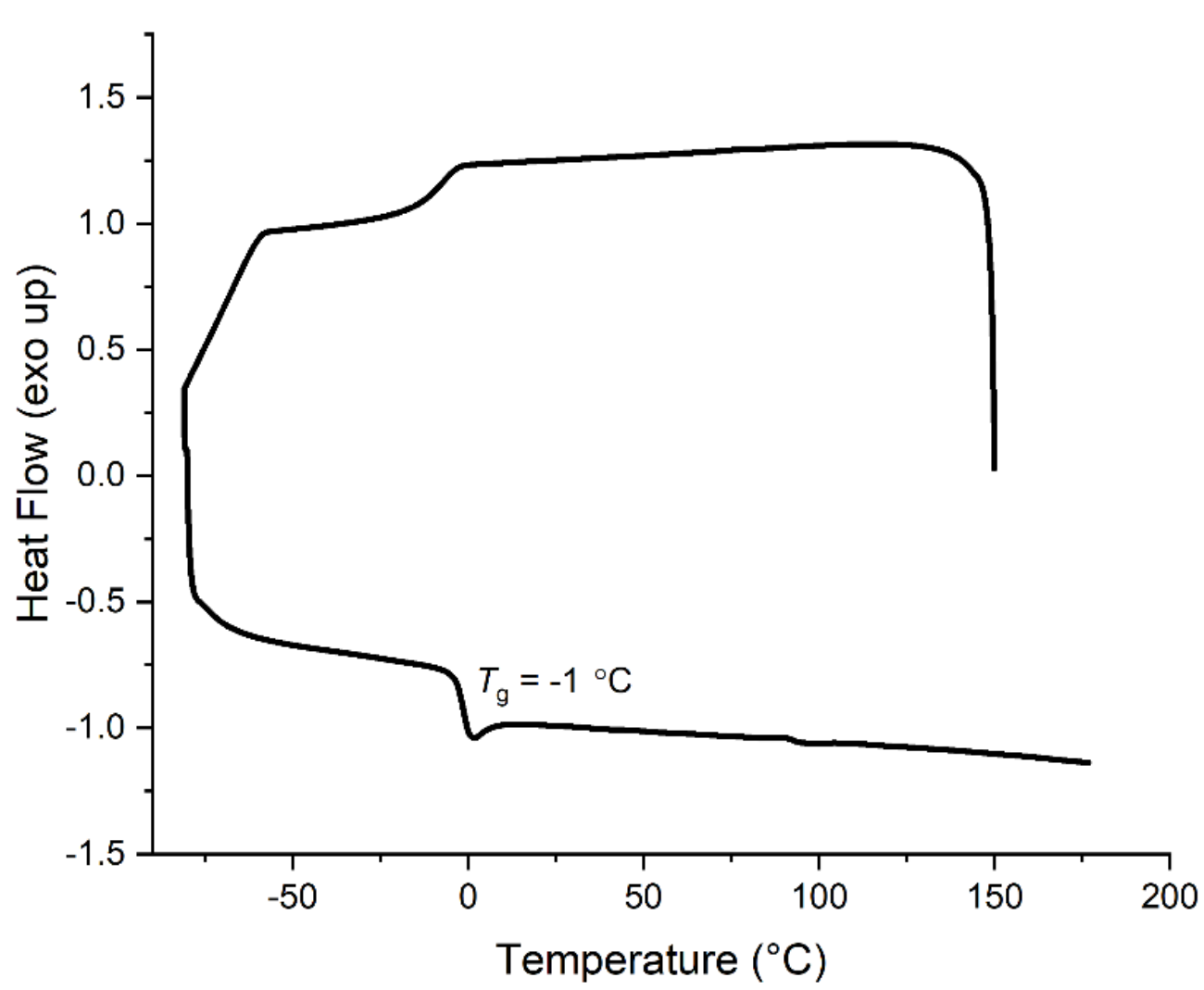

Figure S52. DSC curves of 1st cooling and 2nd heating scans of atactic P(BL^{hPg}) (M_n = 9.50 kDa, $Đ$ = 1.06) prepared with [*rac*-BL^{hPg}]/[I^{t}Bu]/[BnOH] = 100:1:1. Scan rate: 10 °C/min.

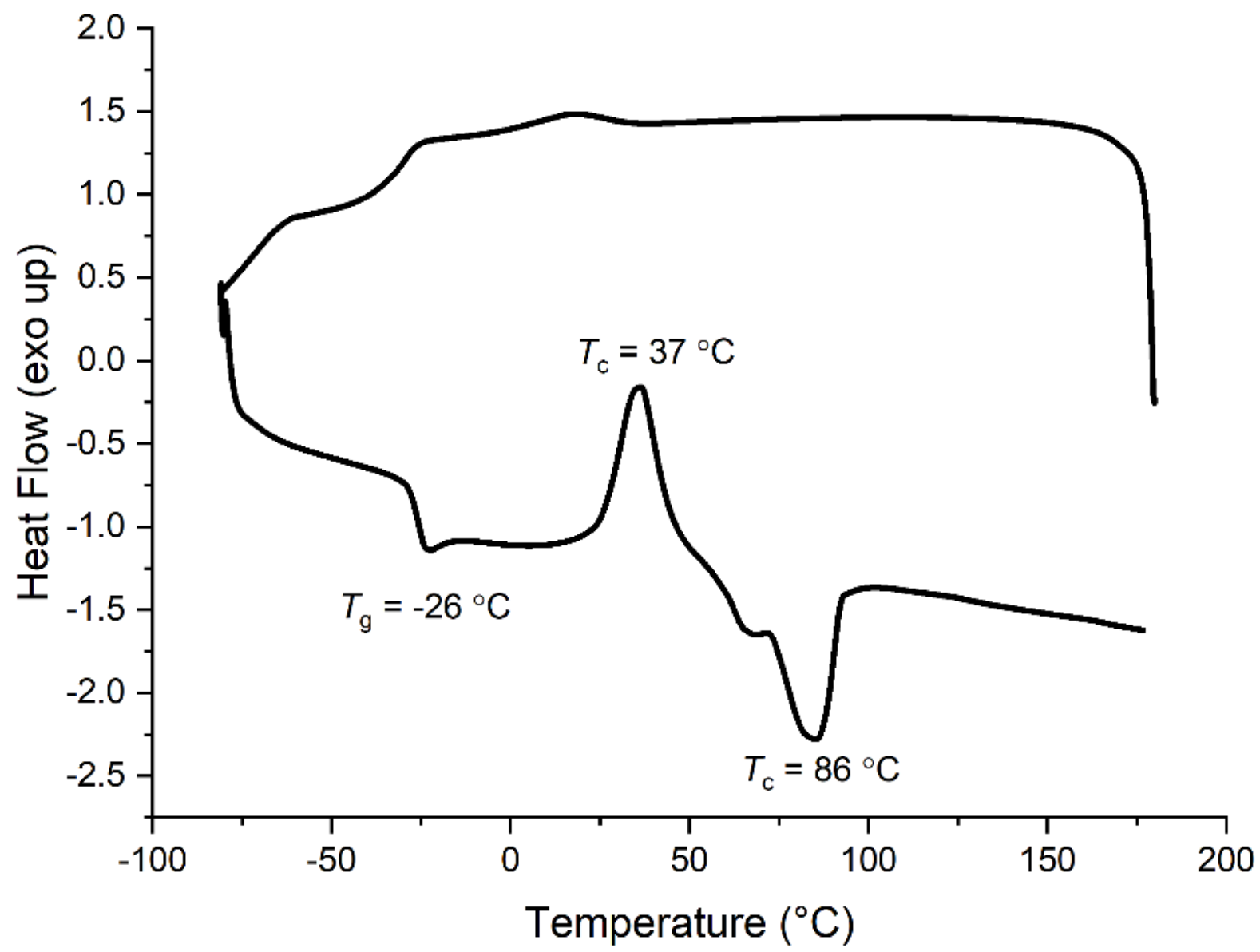

Figure S53. DSC curves of 1st cooling and 2nd heating scans of P(BL^{vinyl}) (M_n = 5.44 kDa, $Đ$ = 1.03, P_r = 0.76). Scan rate: 10 °C/min.

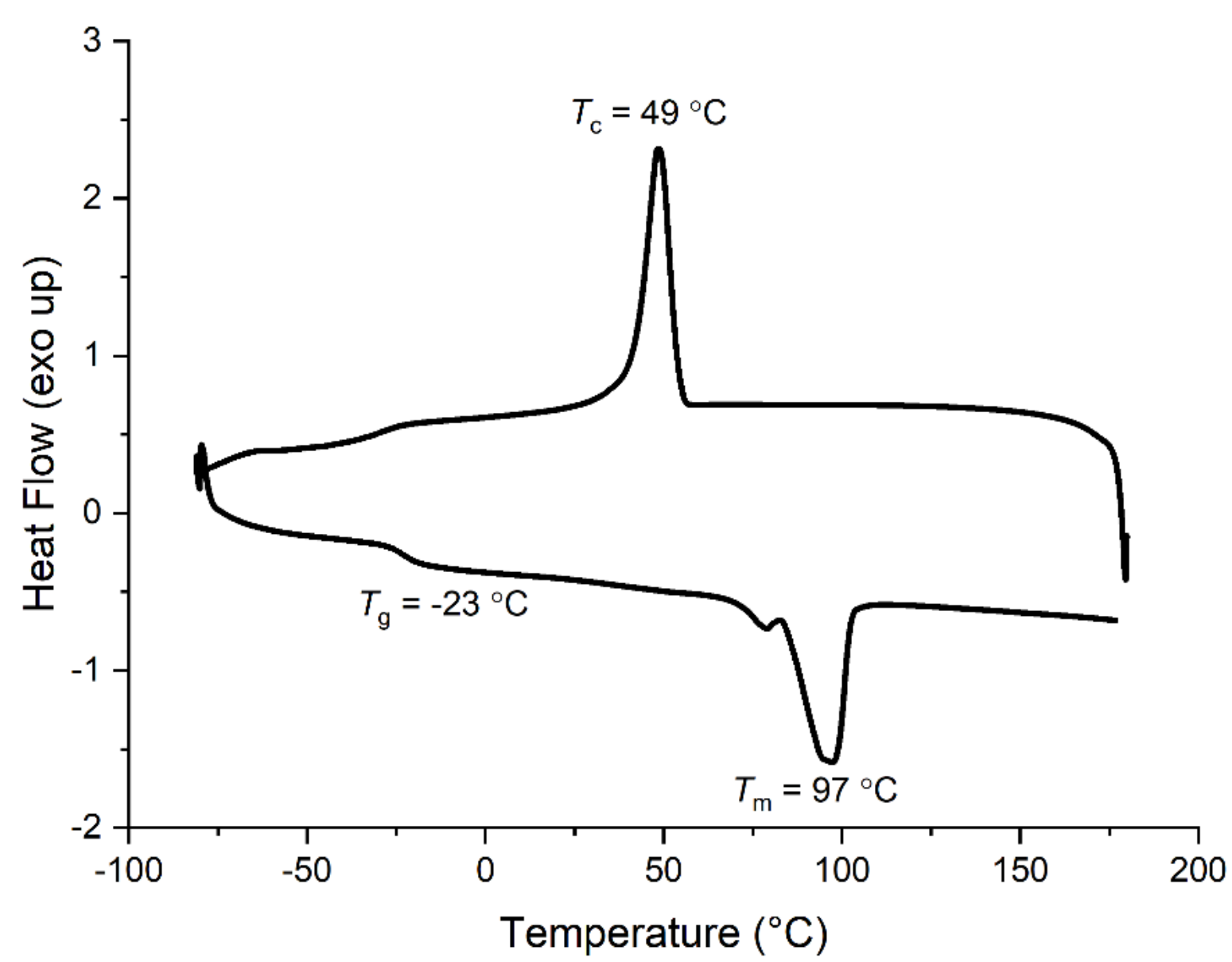

Figure S54. DSC curves of 1st cooling and 2nd heating scans of P(BL^{vinyl}) (M_n = 7.76 kDa, $Đ$ = 1.03, P_r = 0.80). Scan rate: 10 °C/min.

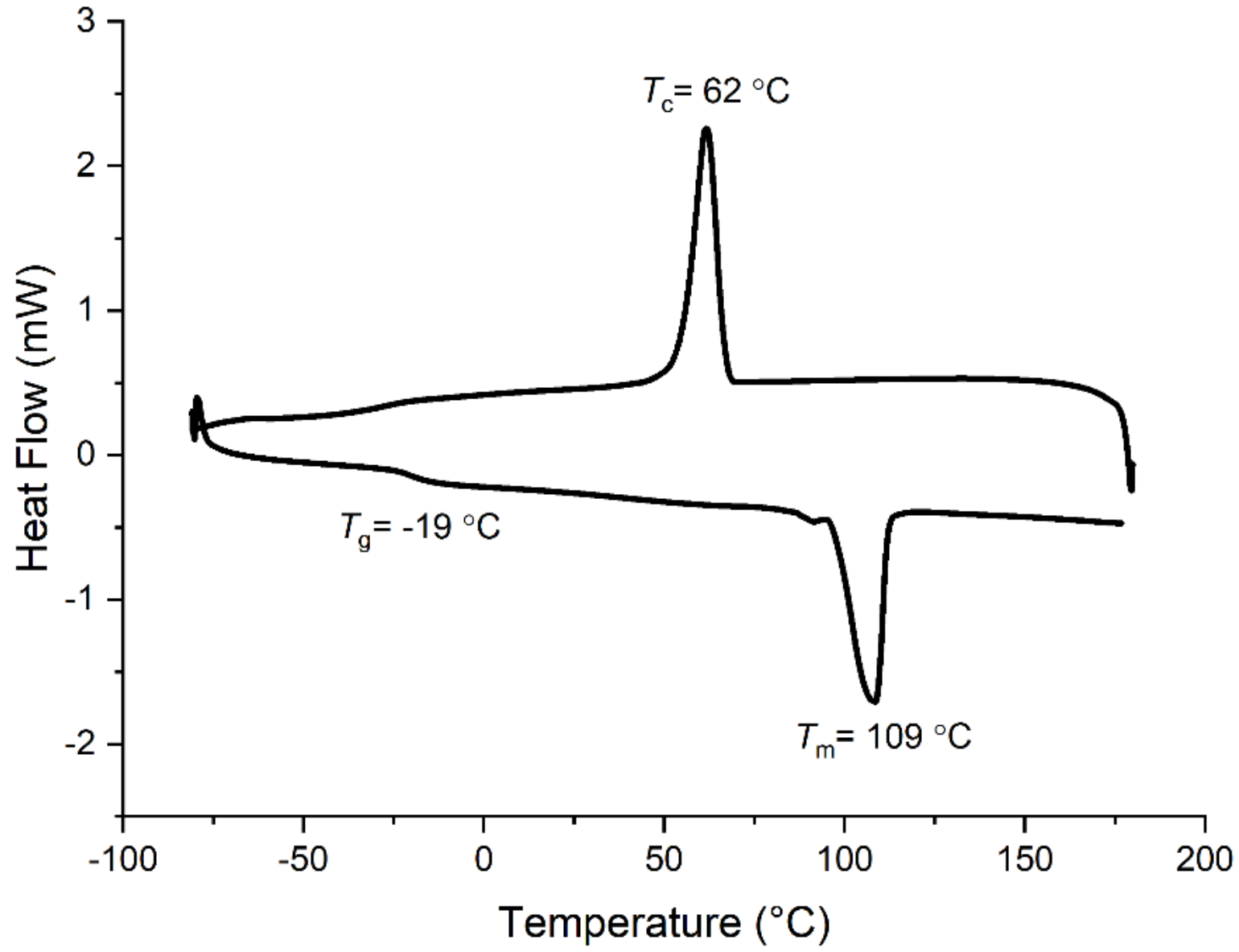

Figure S55. DSC curves of 1st cooling and 2nd heating scans of P(BL^{vinyl}) (M_n = 12.2 kDa, $Đ$ = 1.05, P_r = 0.83). Scan rate: 10 °C/min.

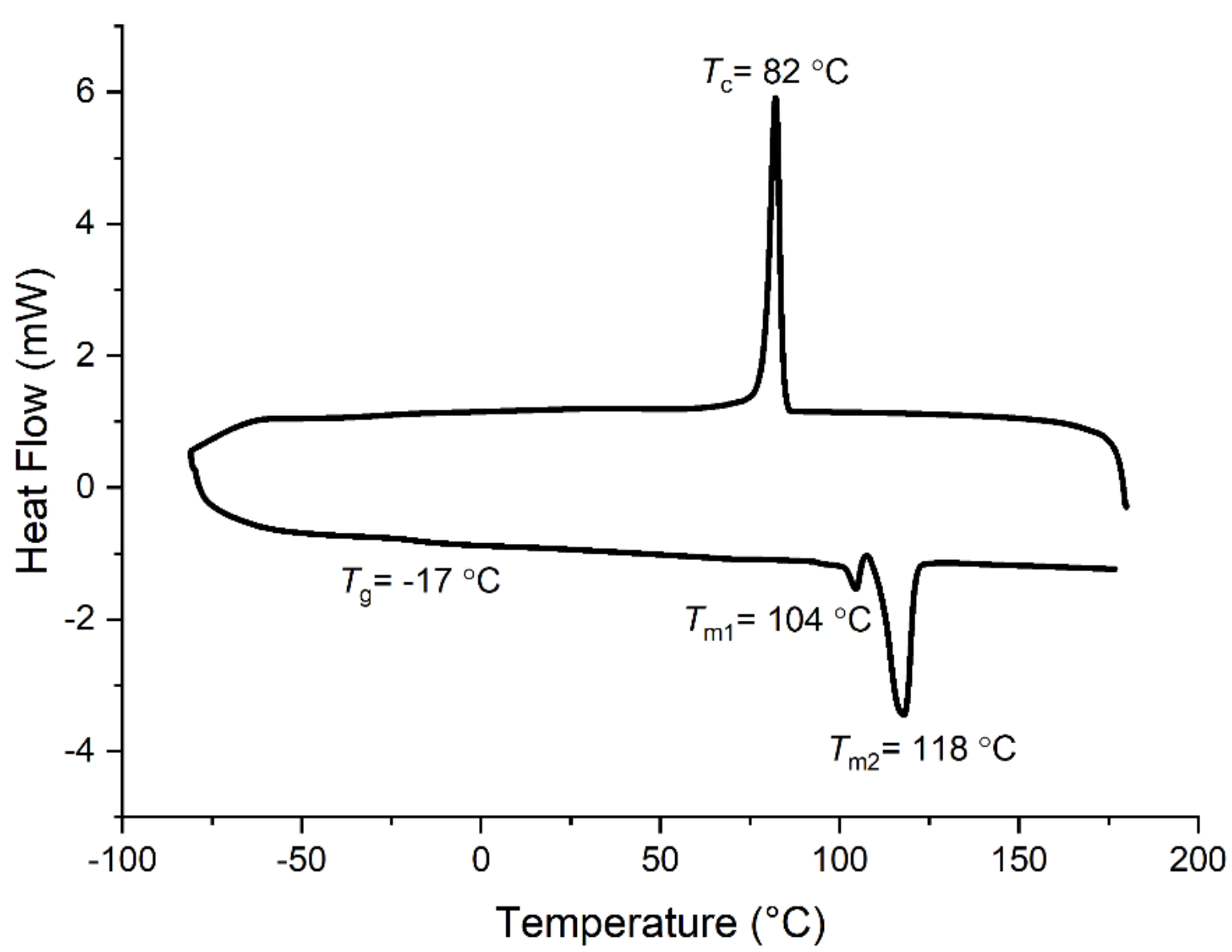

Figure S56. DSC curves of 1st cooling and 2nd heating scans of P(BL^{vinyl}) (M_n = 9.53 kDa, $Đ$ = 1.02, P_r = 0.89). Scan rate: 10 °C/min.

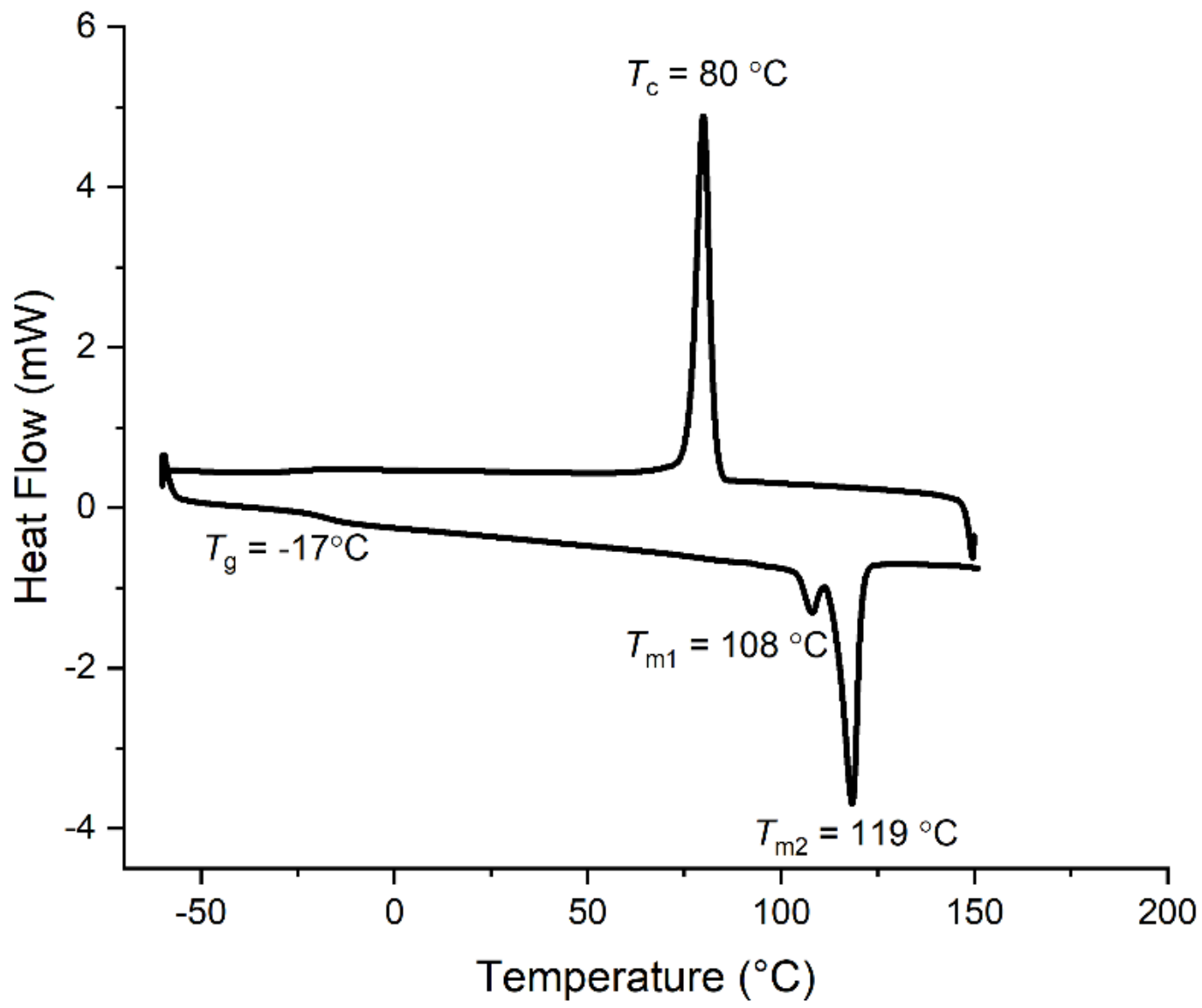

Figure S57. DSC curves of 1st cooling and 2nd heating scans of P(BL^{vinyl}) (M_n = 34.0 kDa, $Đ$ = 1.02, P_r = 0.91). Scan rate: 10 °C/min.

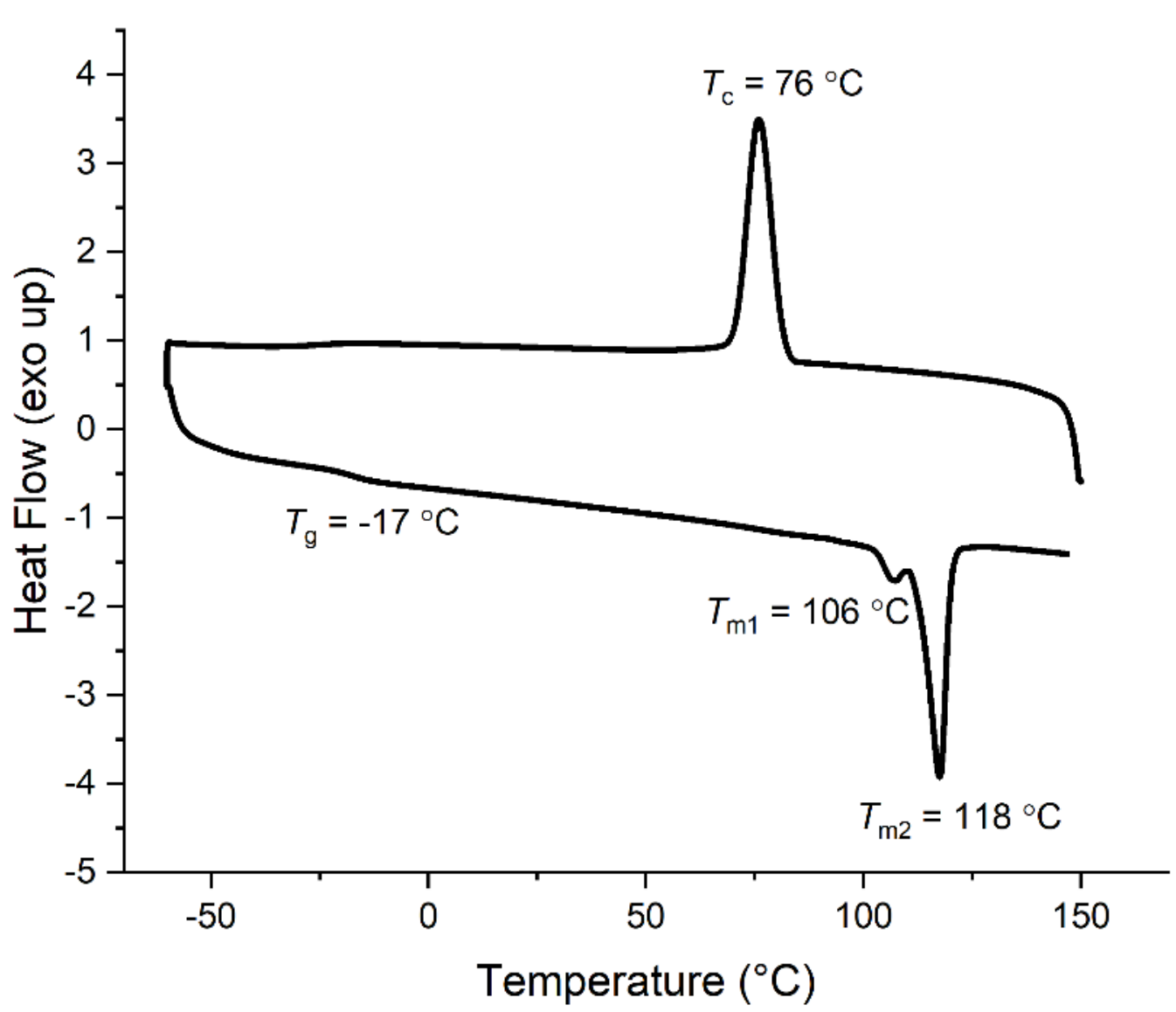

Figure S58. DSC curves of 1st cooling and 2nd heating scans of P(BL^{vinyl}) (M_n = 42.6 kDa, $Đ$ = 1.02, P_r = 0.90). Scan rate: 10 °C/min.

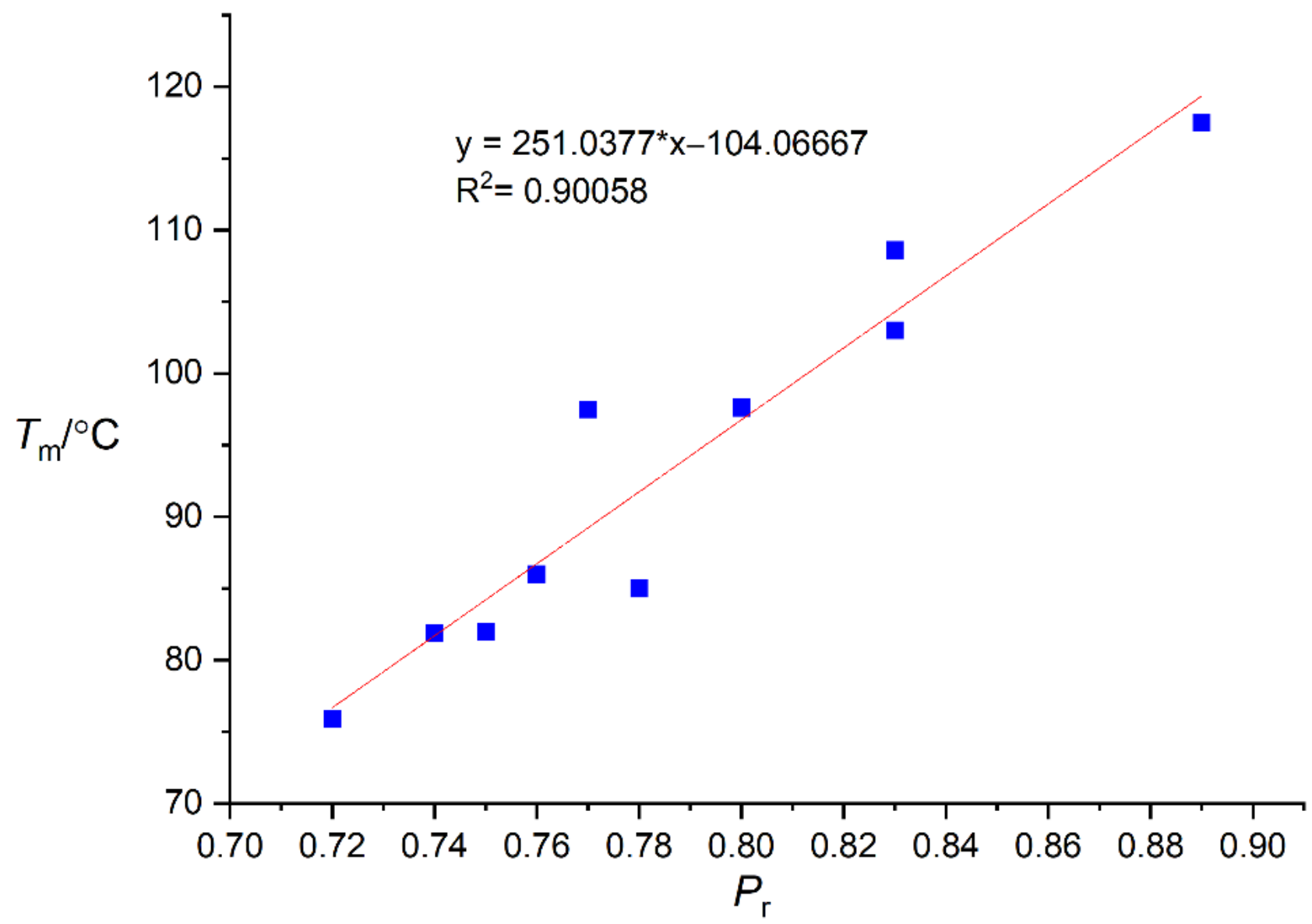

Figure S59. Plot of T_m values *vs* syndiotacticity degree for P(BL^{vinyl}).

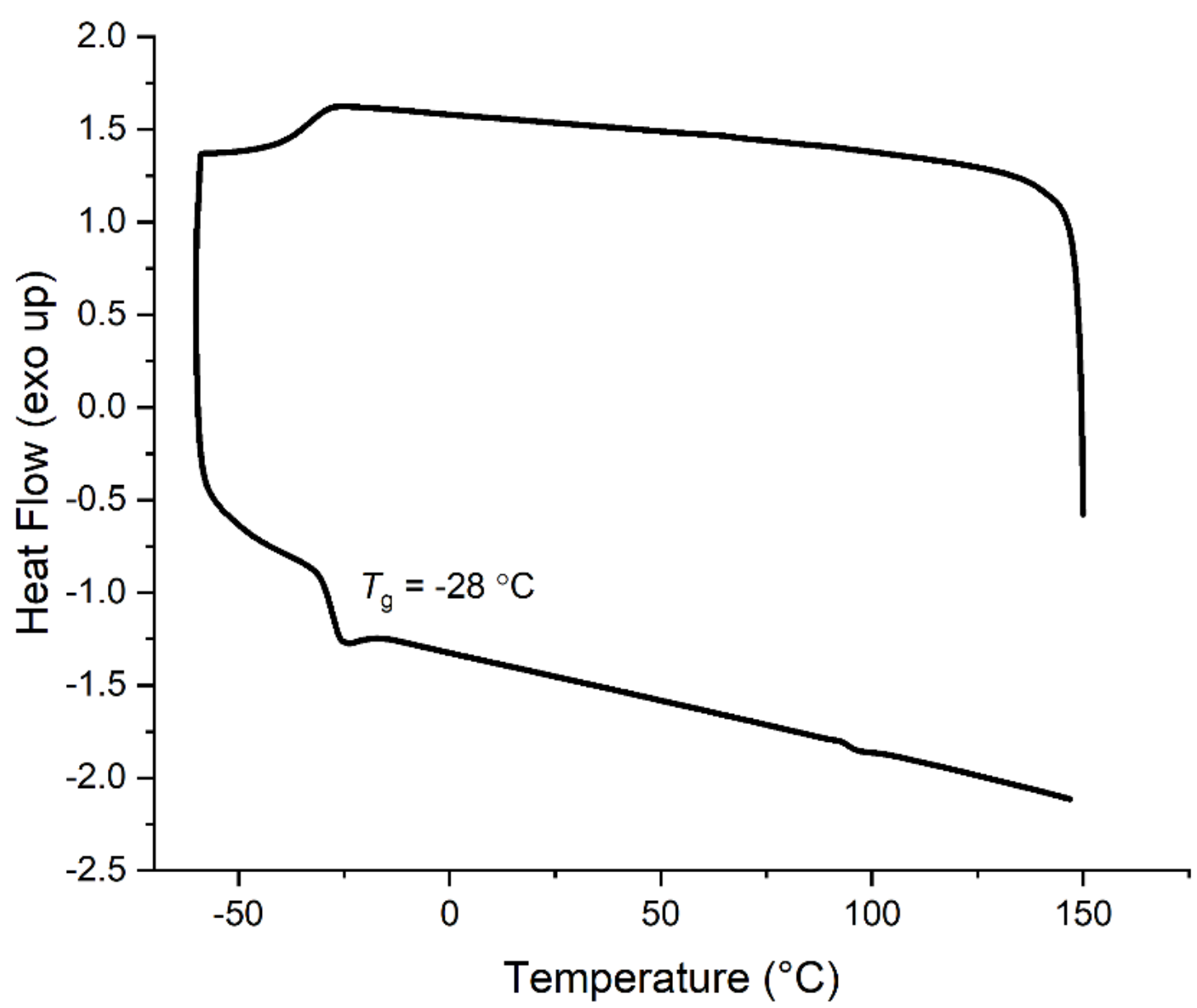

Figure S60. DSC curves of 1st cooling and 2nd heating scans of (*S*)-P(BL^{vinyl}) (M_n = 7.3 kDa, $Đ$ = 1.12, P_m = 0.93). Scan rate: 10 °C/min.

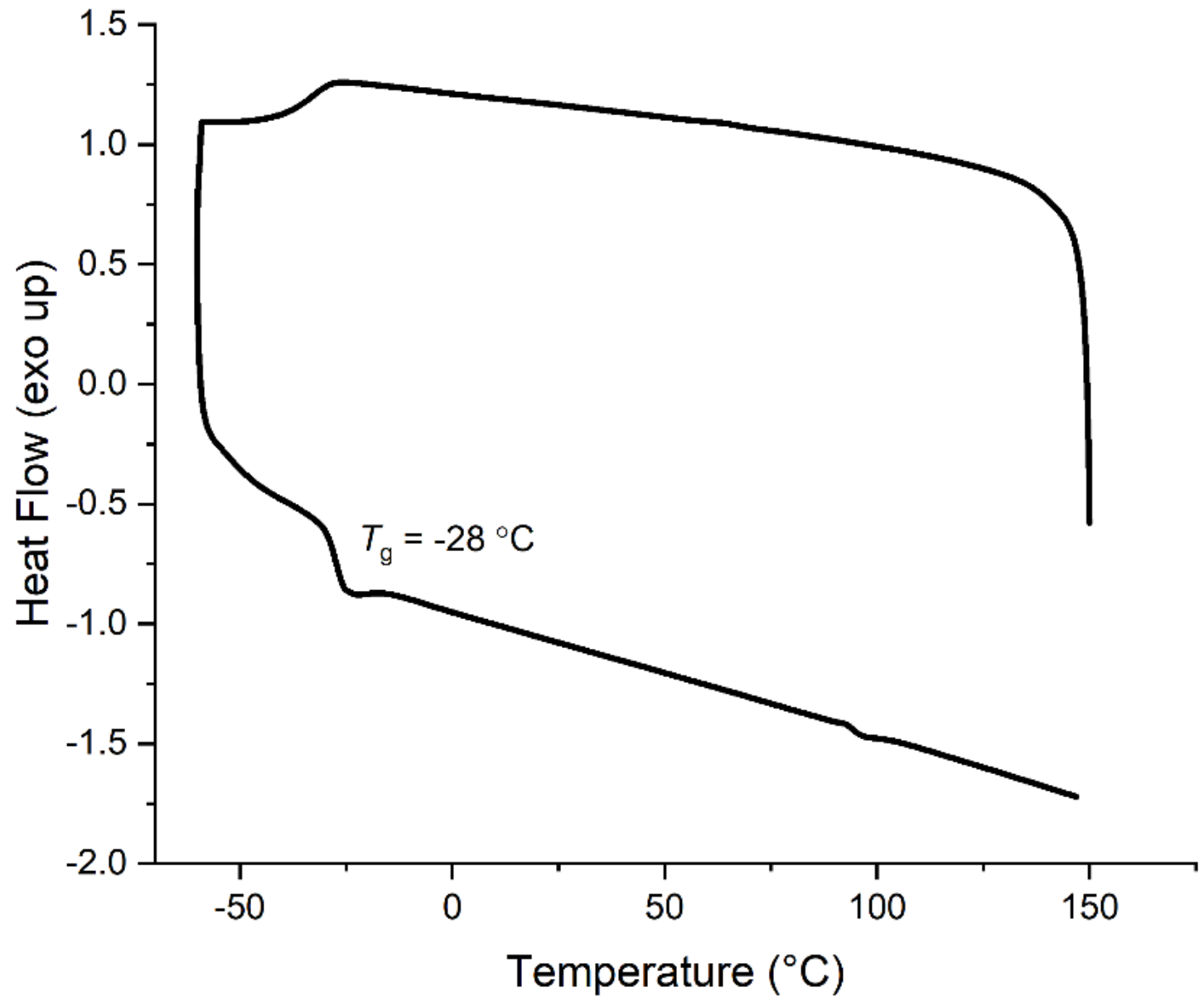

Figure S61. DSC curves of 1st cooling and 2nd heating scans of (*R*)-P(BL^{vinyl}) (M_n = 10.3 kDa, $Đ$ = 1.09, P_m = 0.92). Scan rate: 10 °C/min.

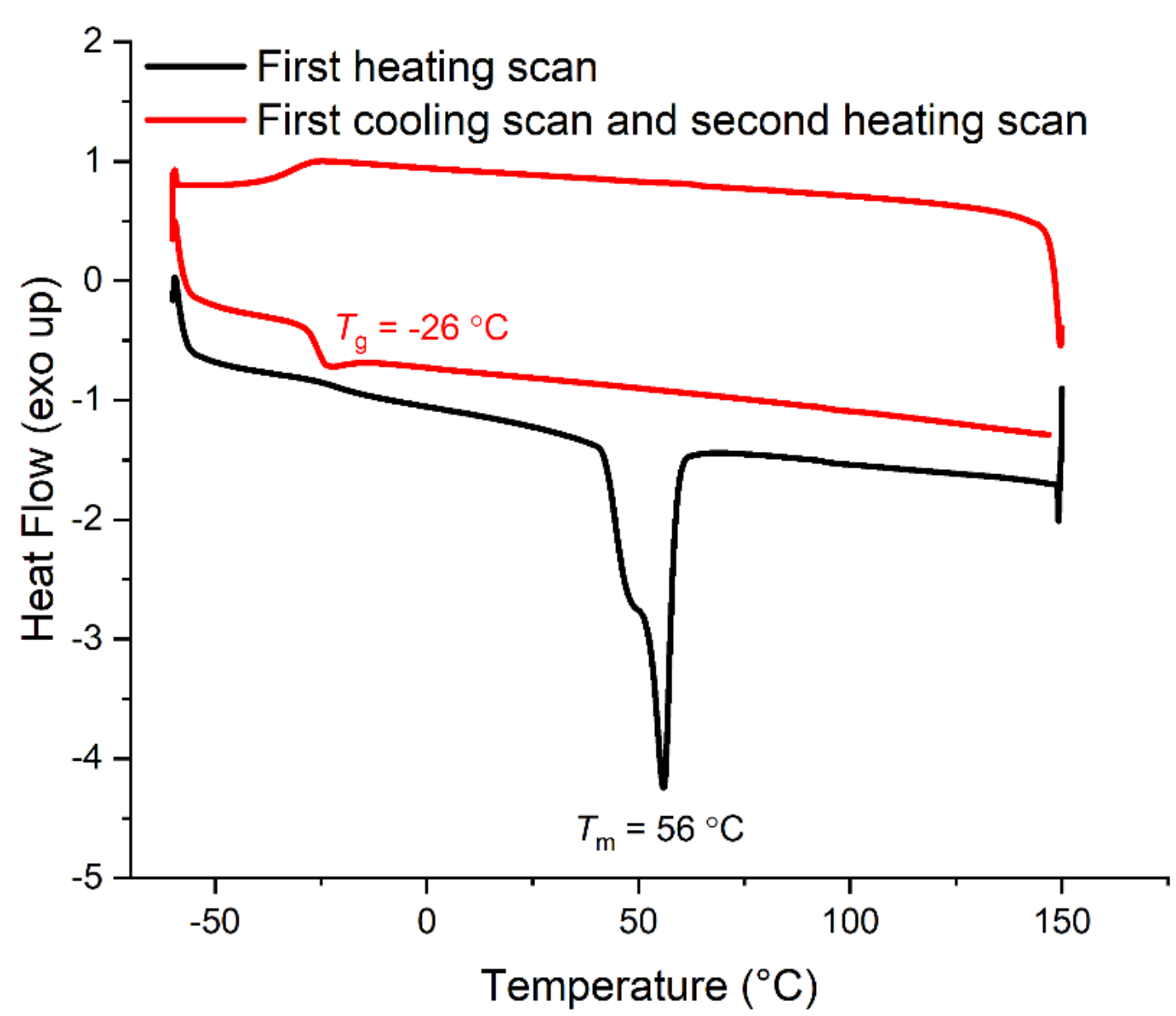

Figure S62. DSC curves of *sc*-P(BL^{vinyl}). Scan rate: 10 °C/min.

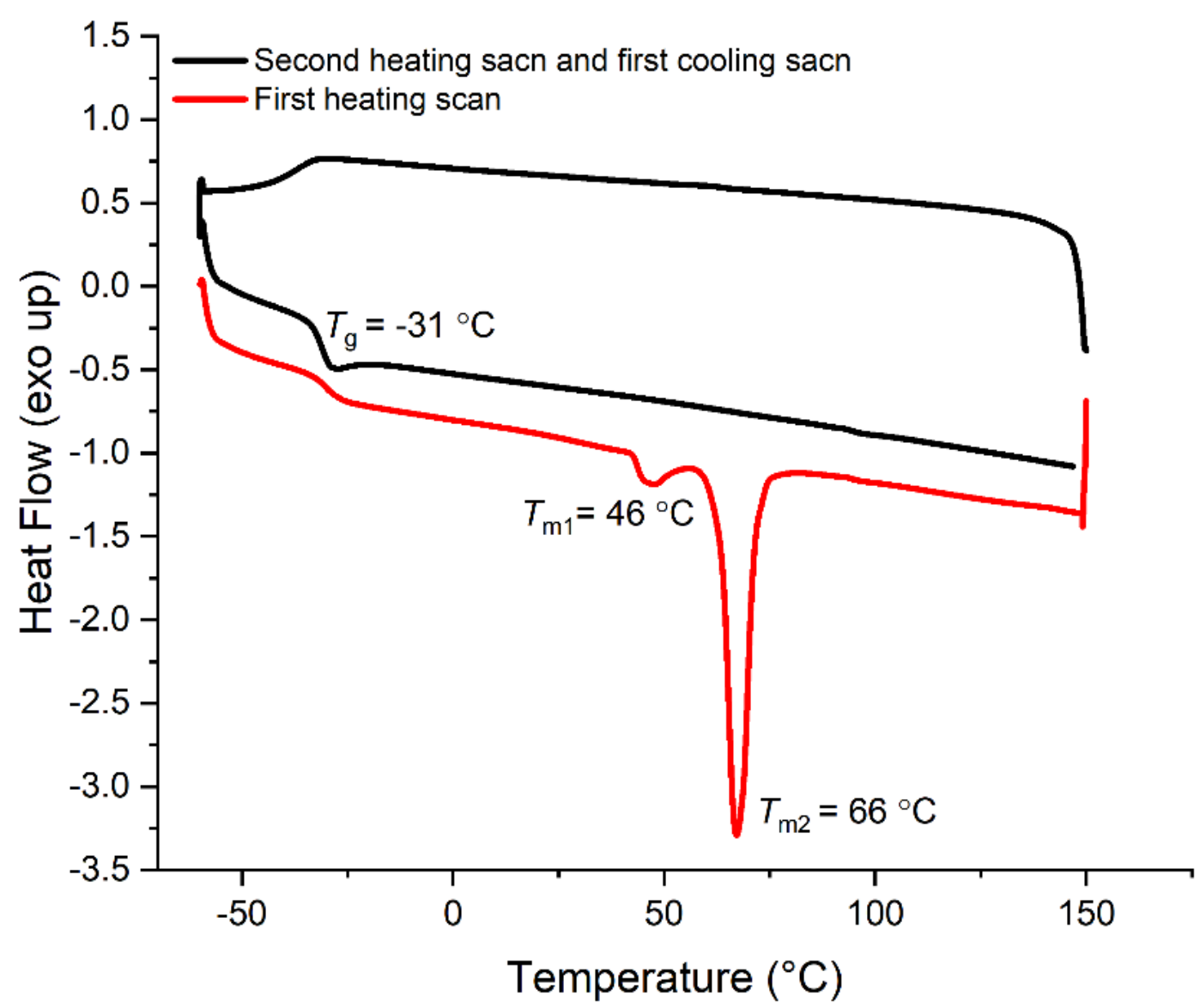

Figure S63. DSC curves P(BL^{allyl}) (M_n = 14.9 kDa, $Đ$ = 1.02, P_r = 0.80). Scan rate: 10 °C/min.

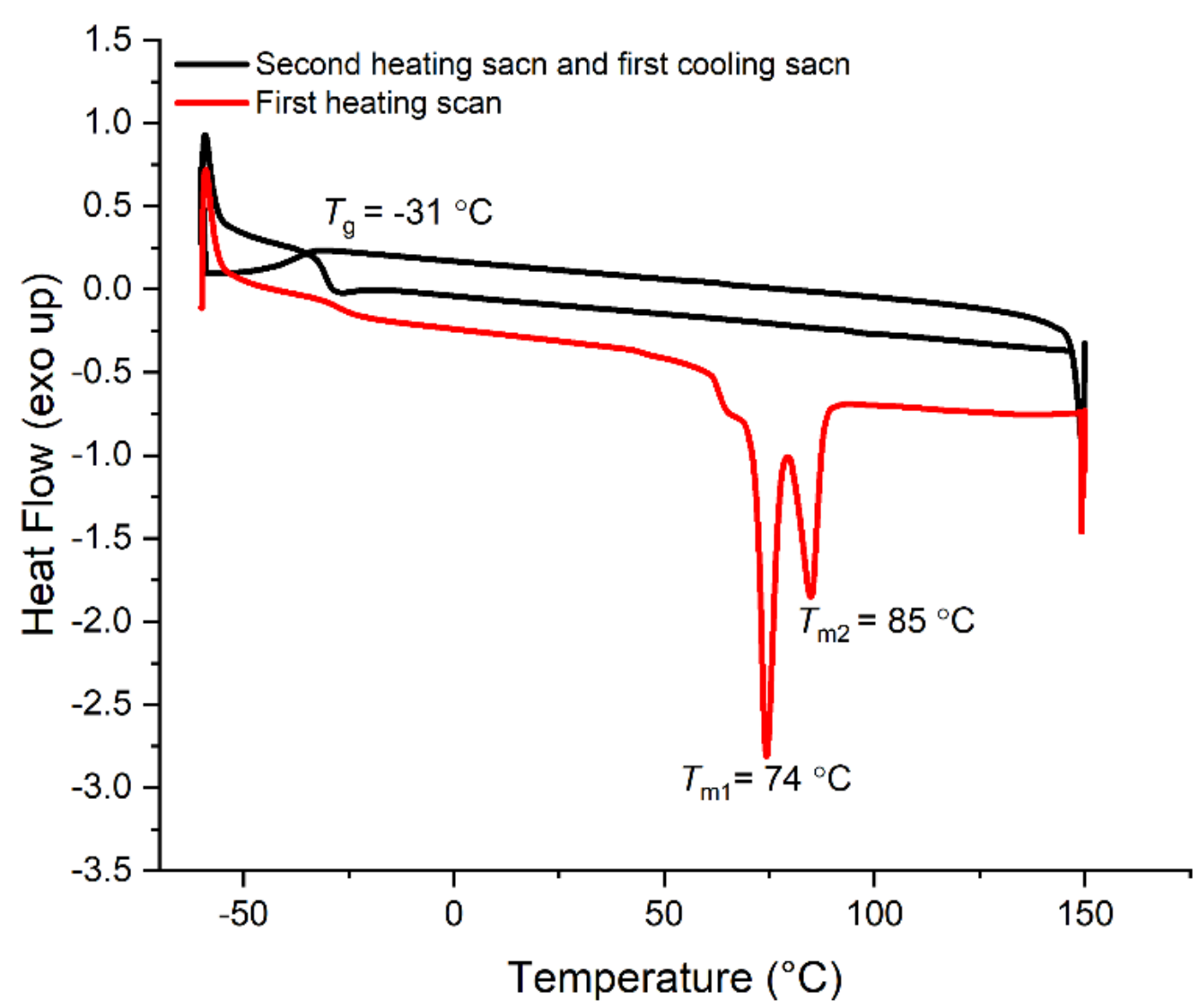

Figure S64. DSC curves P(BL^{allyl}) (M_n = 15.5 kDa, $Đ$ = 1.01, P_r = 0.85). Scan rate: 10 °C/min.

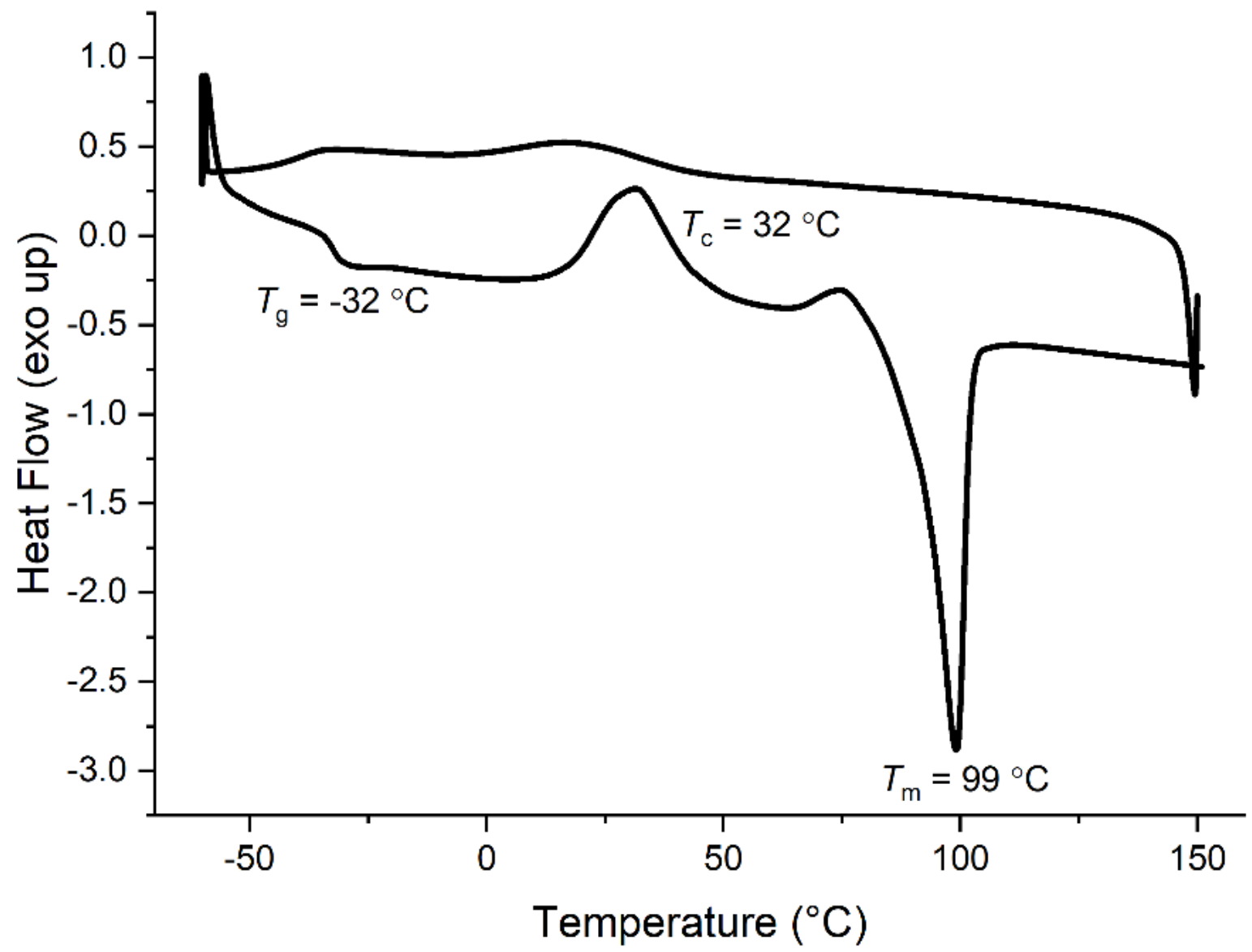

Figure S65. DSC curves of 1st cooling and 2nd heating scans of P(BL^{allyl}) (M_n = 15.1 kDa, $Đ$ = 1.20, P_r = 0.95). Scan rate: 10 °C/min.

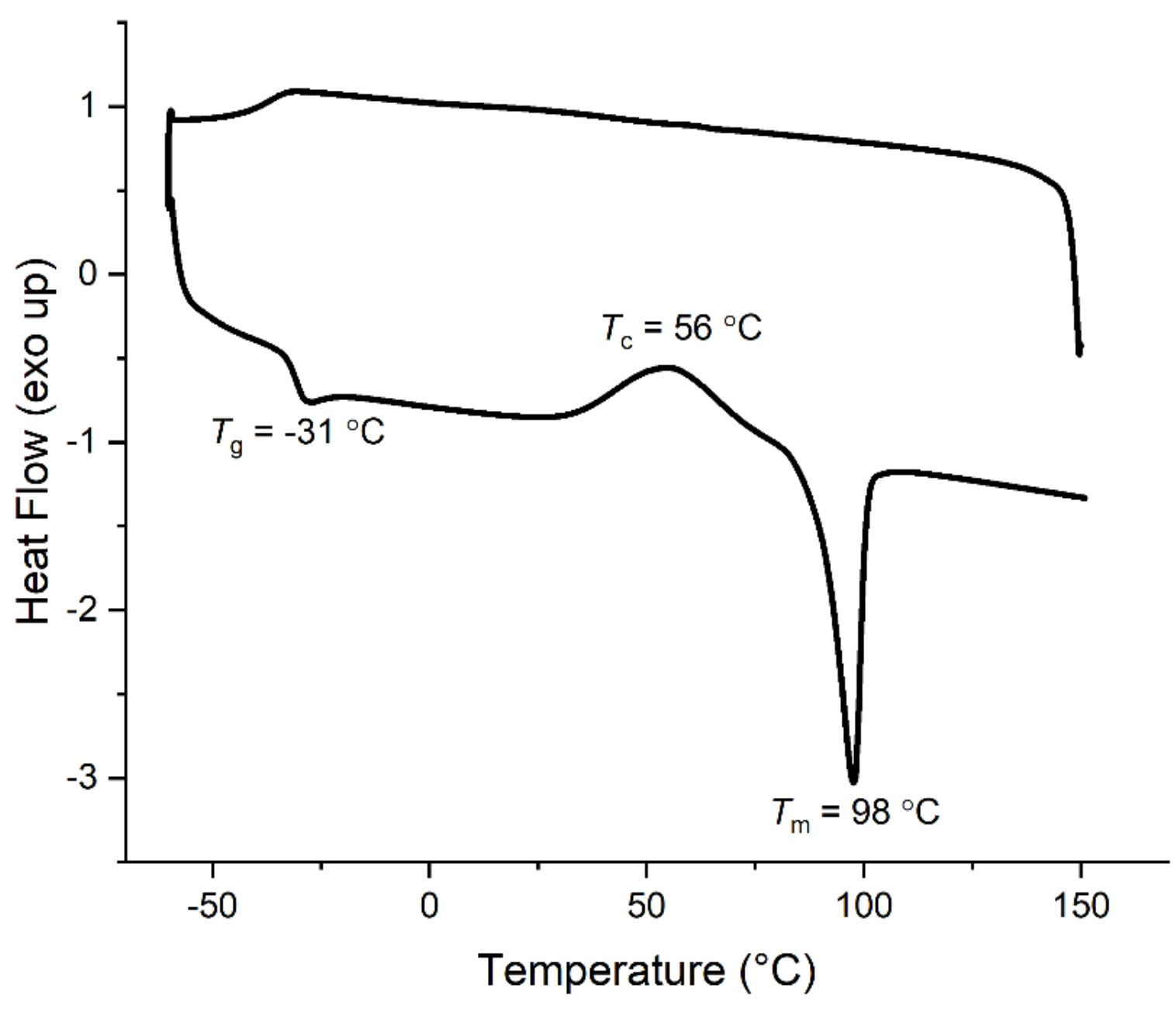

Figure S66. DSC curves of 1st cooling and 2nd heating scans of P(BL^{allyl}) (M_n = 65.1 kDa, $Đ$ = 1.03, P_r = 0.93). Scan rate: 10 °C/min.

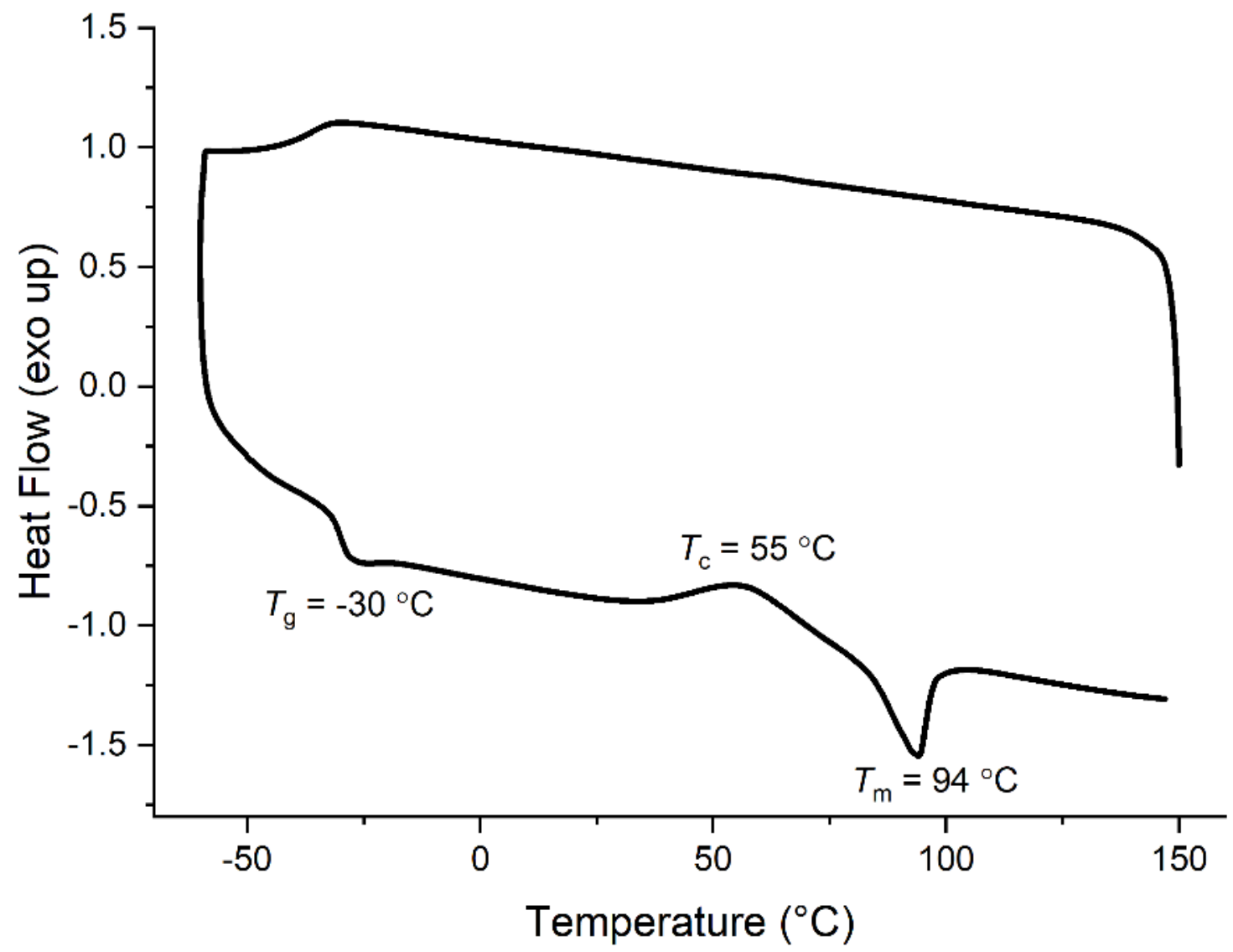

Figure S67. DSC curves of 1st cooling and 2nd heating scans of P(BL^{allyl}) (M_n = 103 kDa, $Đ$ = 1.08, P_r = 0.95). Scan rate: 10 °C/min.

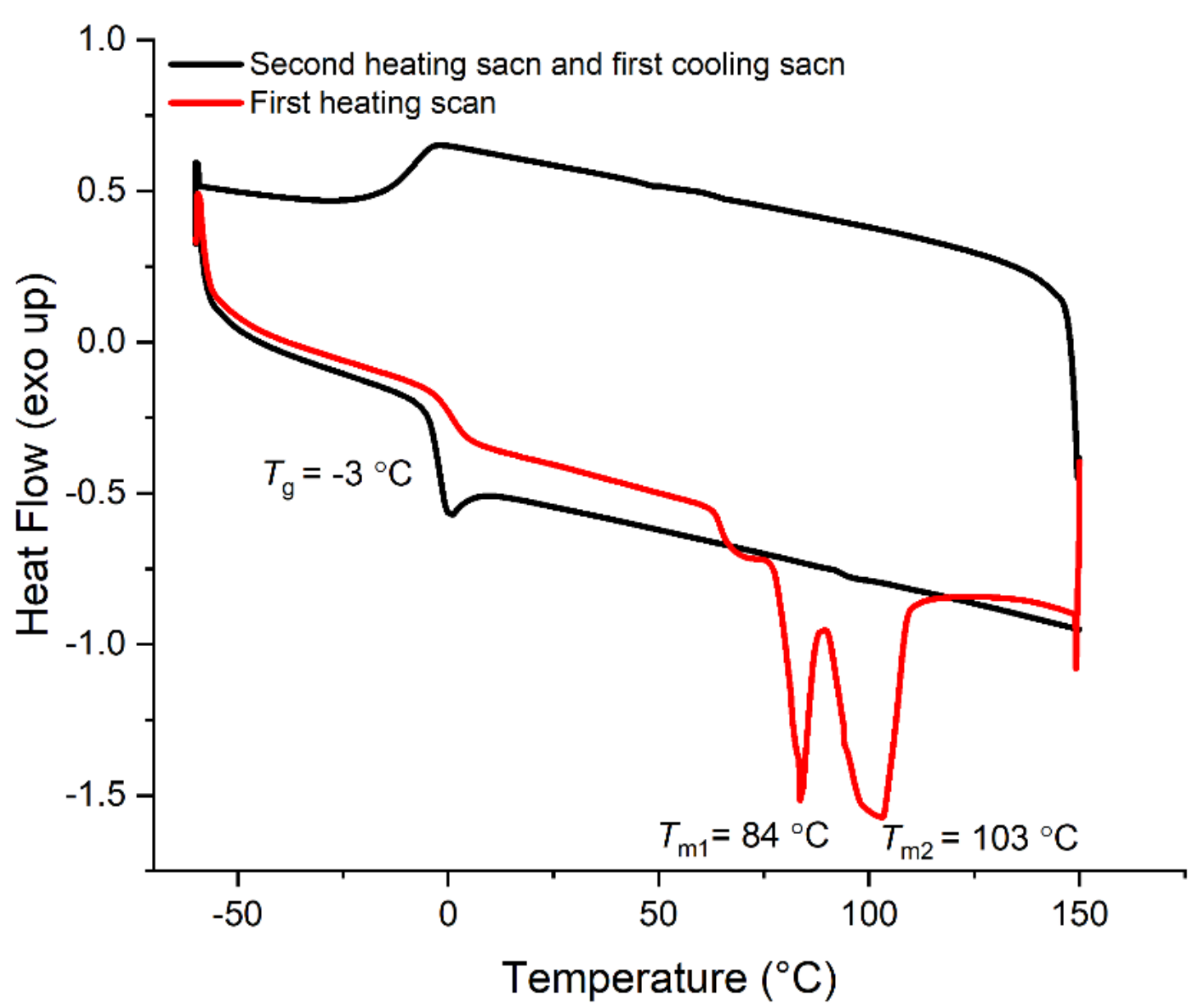

Figure S68. DSC curves of P(BL^{hPg}) (M_n = 7.58 kDa, $Đ$ = 1.03, P_r = 0.81). Scan rate: 10 °C/min.

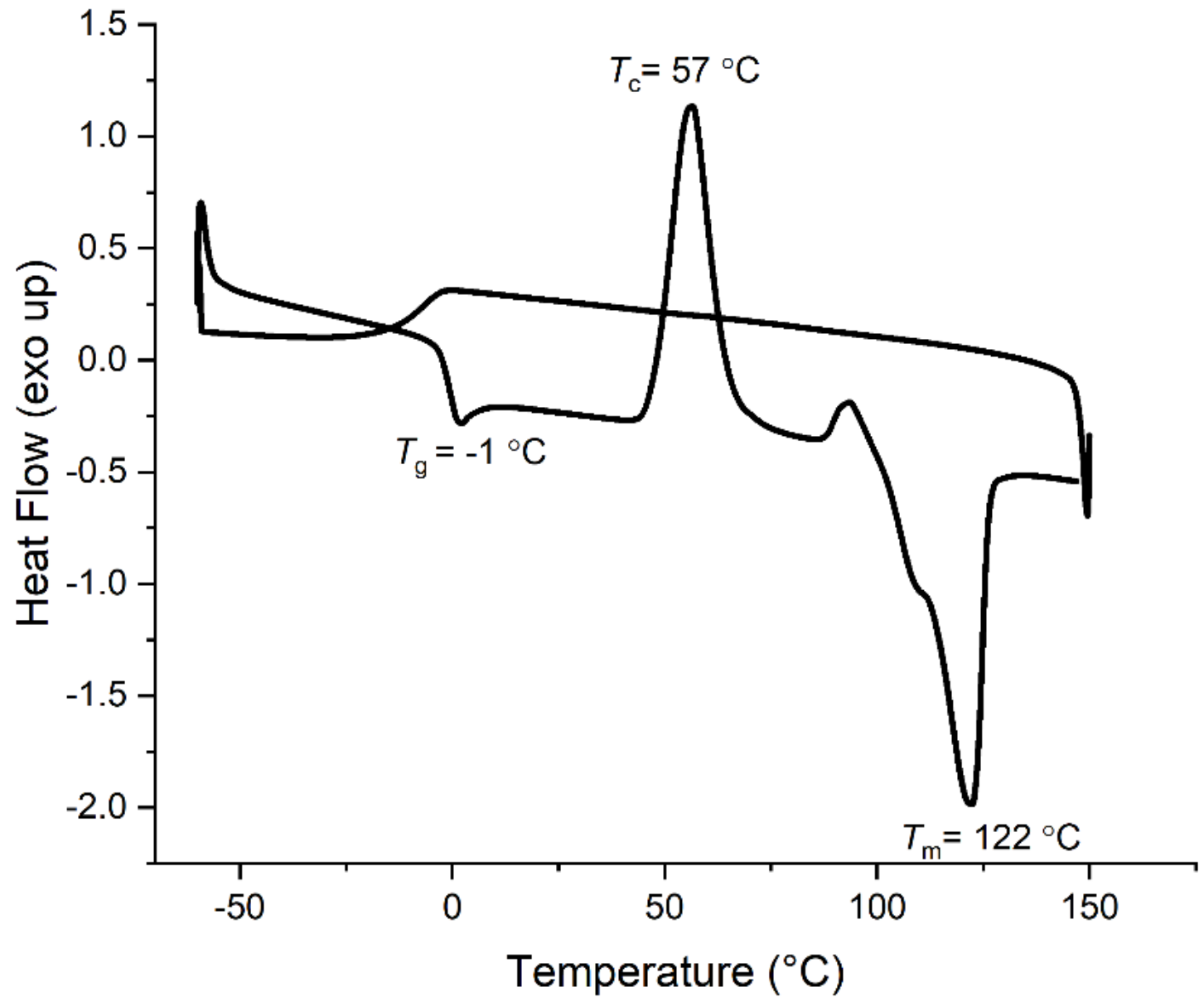

Figure S69. DSC curves of 1st cooling and 2nd heating scans of P(BL^{hPg}) (M_n = 8.30 kDa, $Đ$ = 1.04, P_r = 0.86). Scan rate: 10 °C/min.

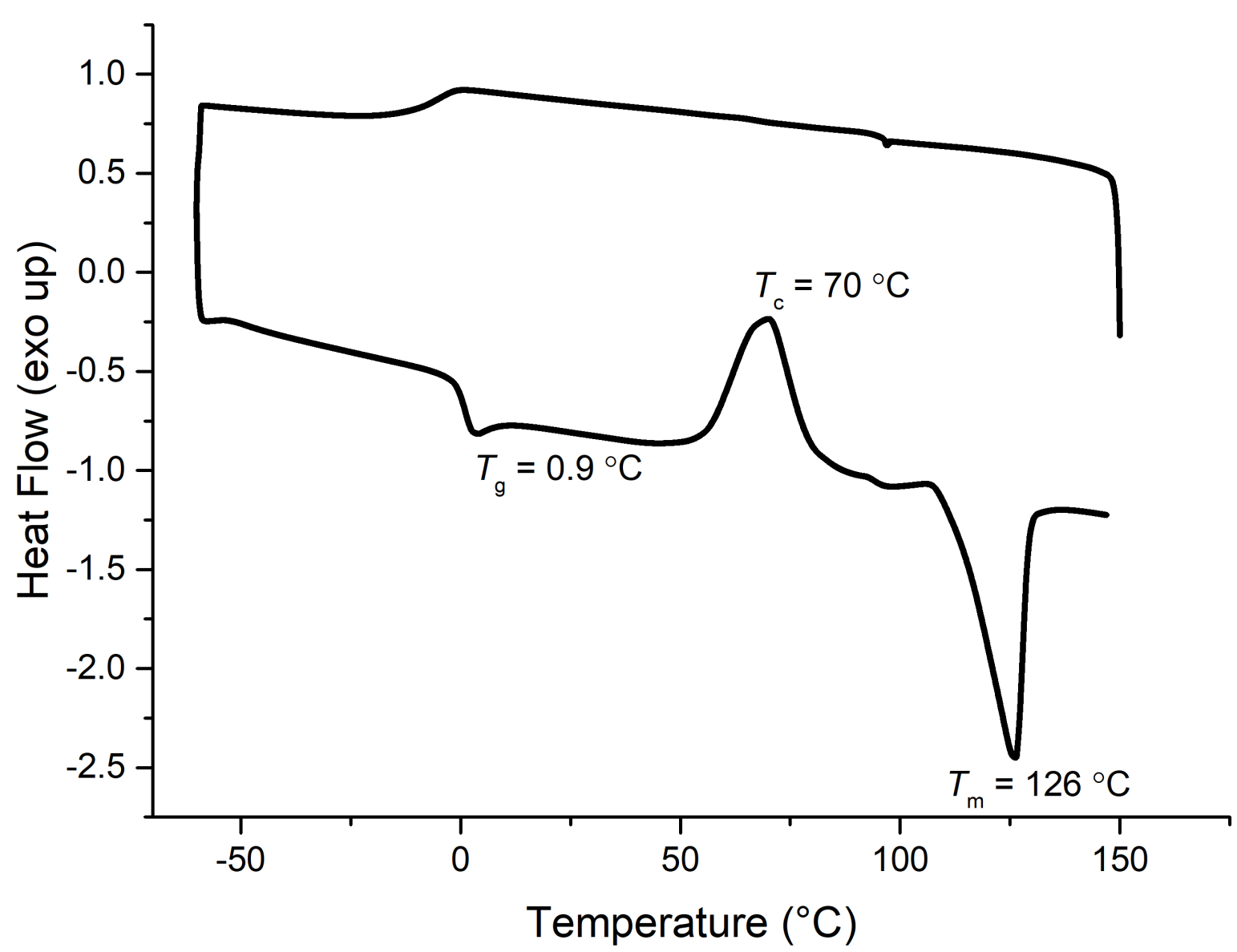

Figure S70. DSC curves of 1st cooling and 2nd heating scans for P(BL^{hPg}) (M_n = 70.5 kDa, $Đ$ = 1.23, P_r = 0.91). Scan rate: 10 °C/min.

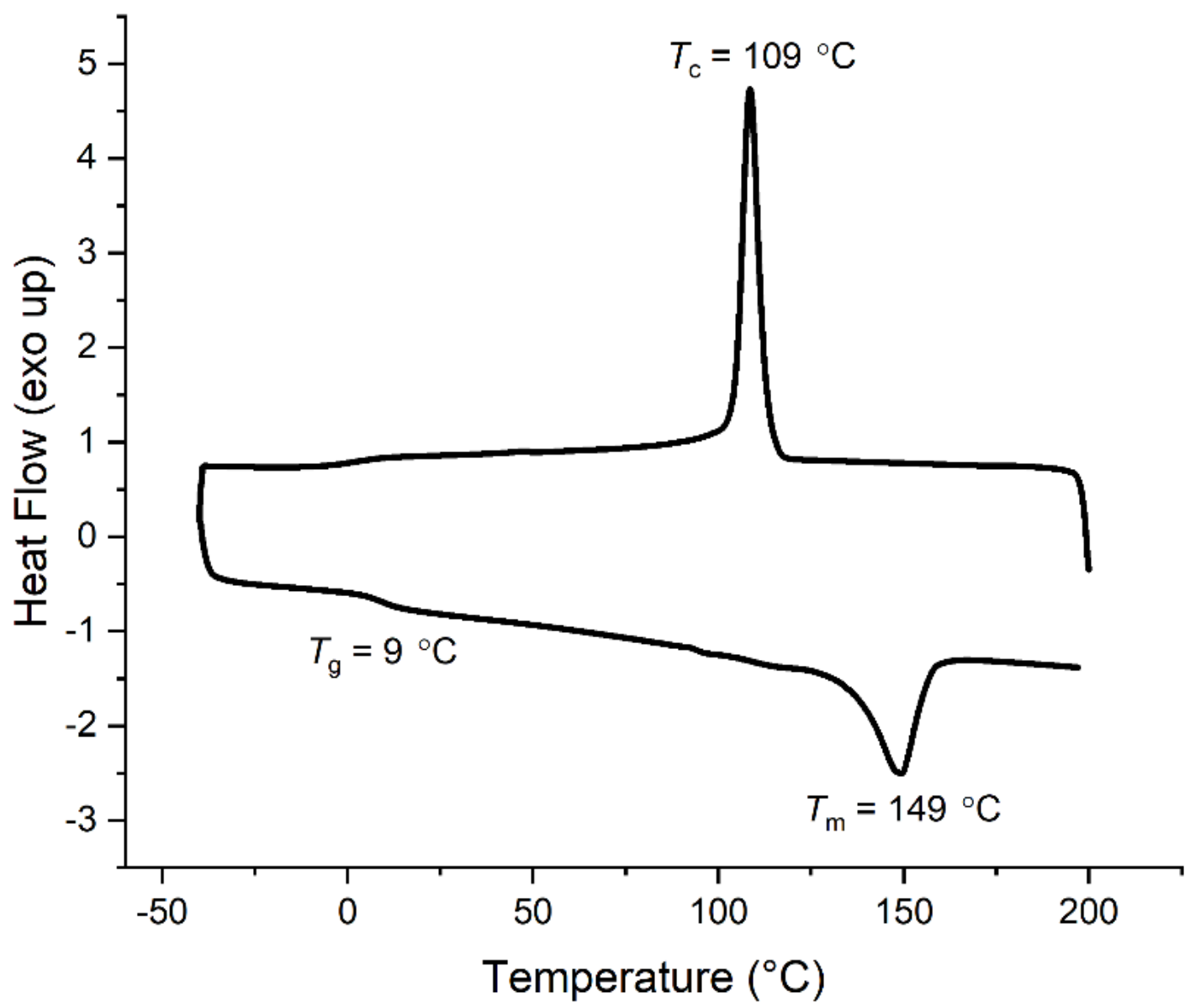

Figure S71. DSC curves of 1st cooling and 2nd heating scans of P(BL^{Me}$_{96}$-*co*-BL^{vinyl}$_{4}$) (M_n = 66.0 kDa, $Đ$ = 1.10). Scan rate: 10 °C/min.

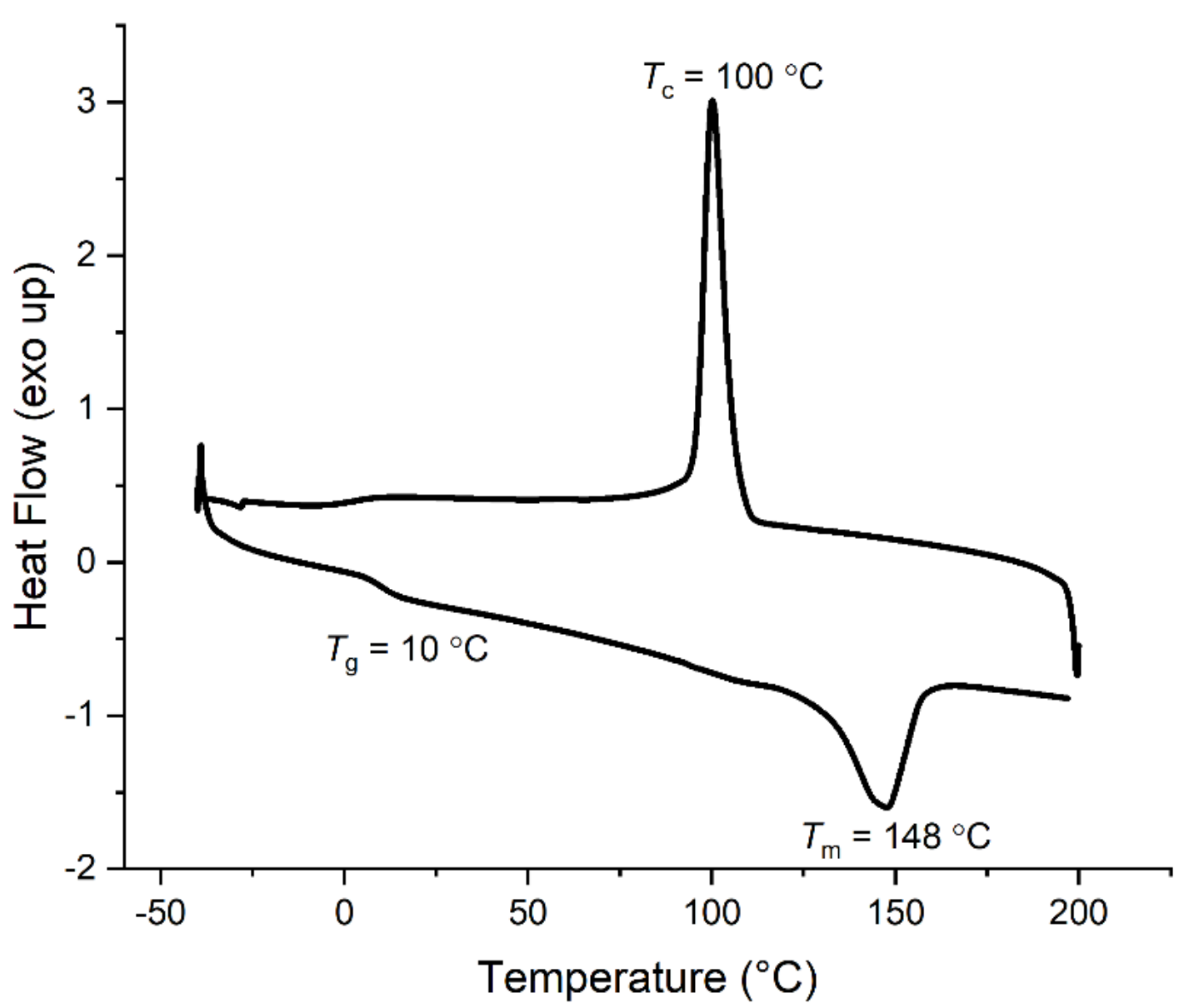

Figure S72. DSC curves of 1st cooling and 2nd heating scans of P(BL^{Me}$_{95}$-*co*-BL^{vinyl}$_{5}$) (M_n = 169 kDa, $Đ$ = 1.08). Scan rate: 10 °C/min.

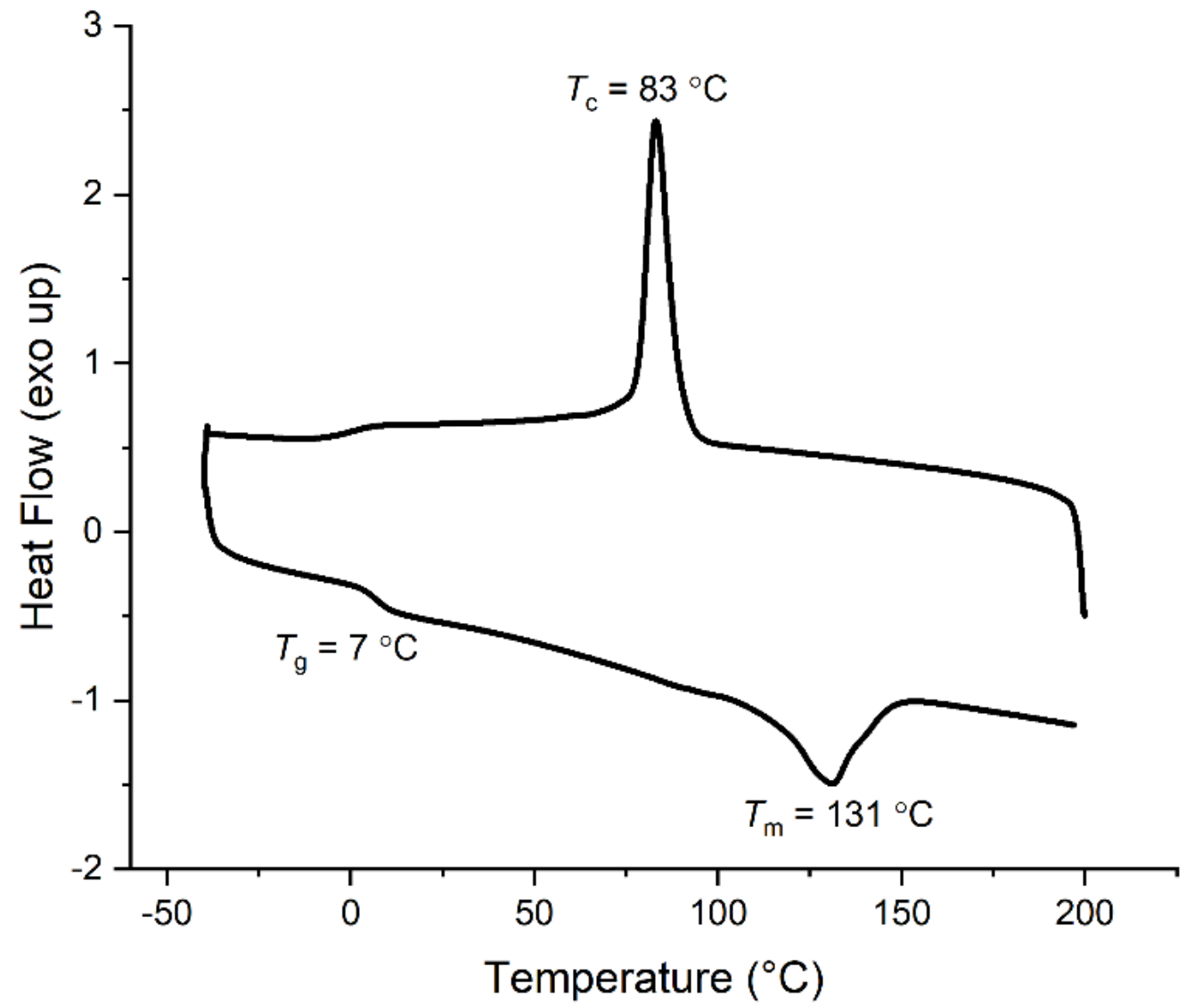

Figure S73. DSC curves of 1st cooling and 2nd heating scans of P($BL^{Me}{}_{90}$-*co*-$BL^{vinyl}{}_{10}$) (M_n = 159 kDa, $Đ$ = 1.05). Scan rate: 10 °C/min.

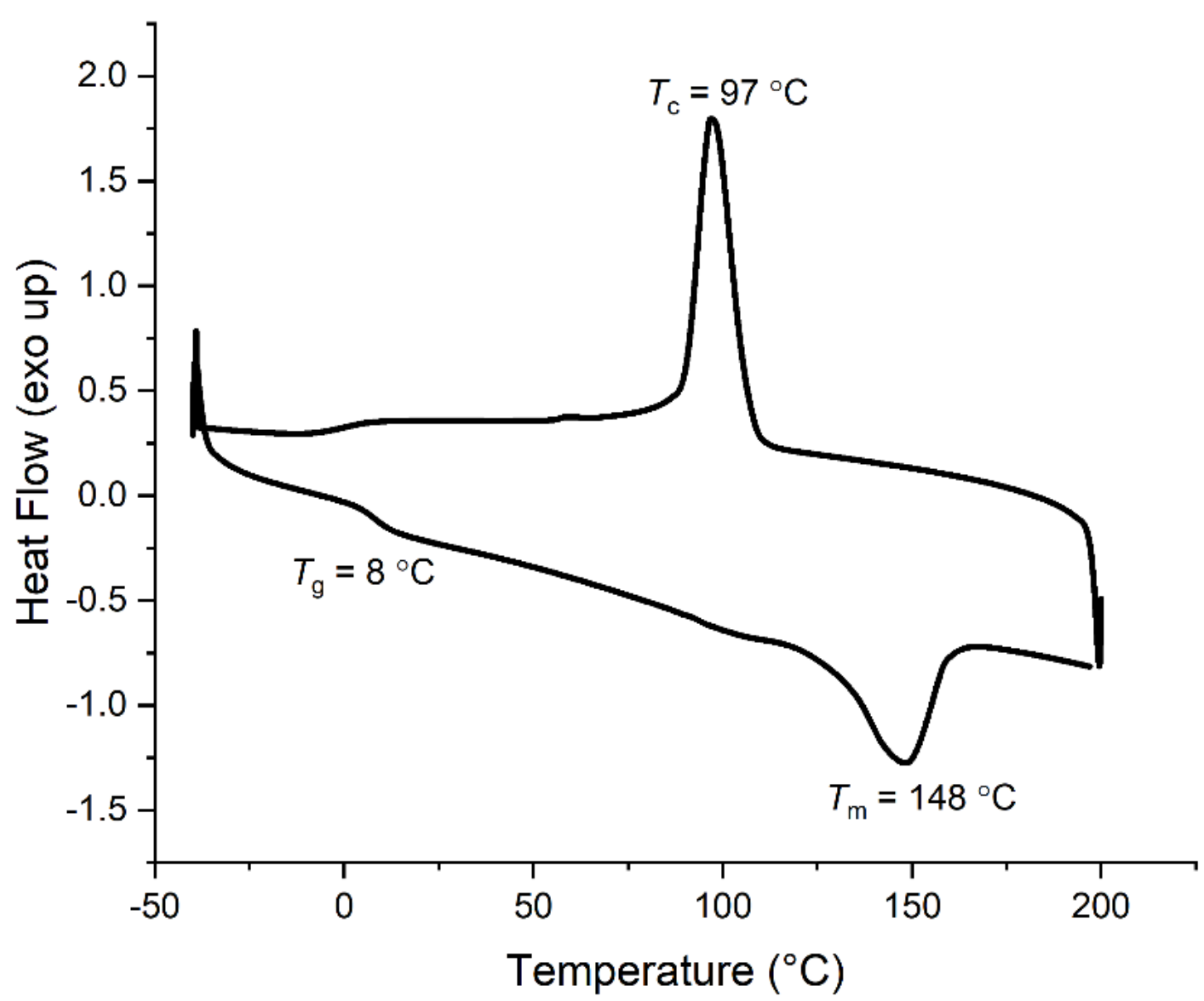

Figure S74. DSC curves of 1st cooling and 2nd heating scans of P($BL^{Me}{}_{96}$-*co*-$BL^{allyl}{}_{4}$) (M_n = 161 kDa, $Đ$ = 1.13). Scan rate: 10 °C/min.

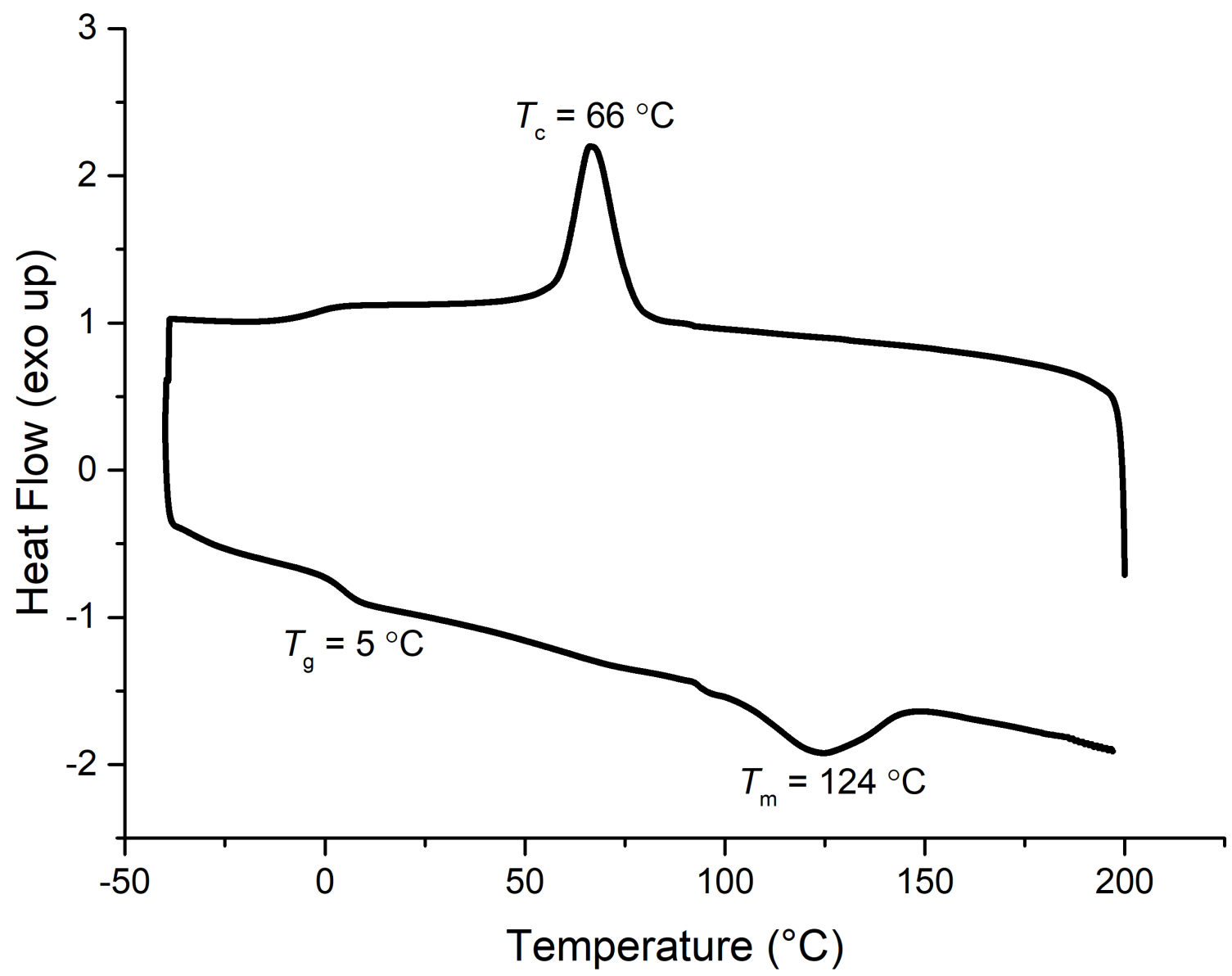

Figure S75. DSC curves of 1st cooling and 2nd heating scans of P(BL$^{Me}_{92}$-*co*-BL$^{allyl}_{8}$) (M_n = 65 kDa, $Đ$ = 1.13). Scan rate: 10 °C/min.

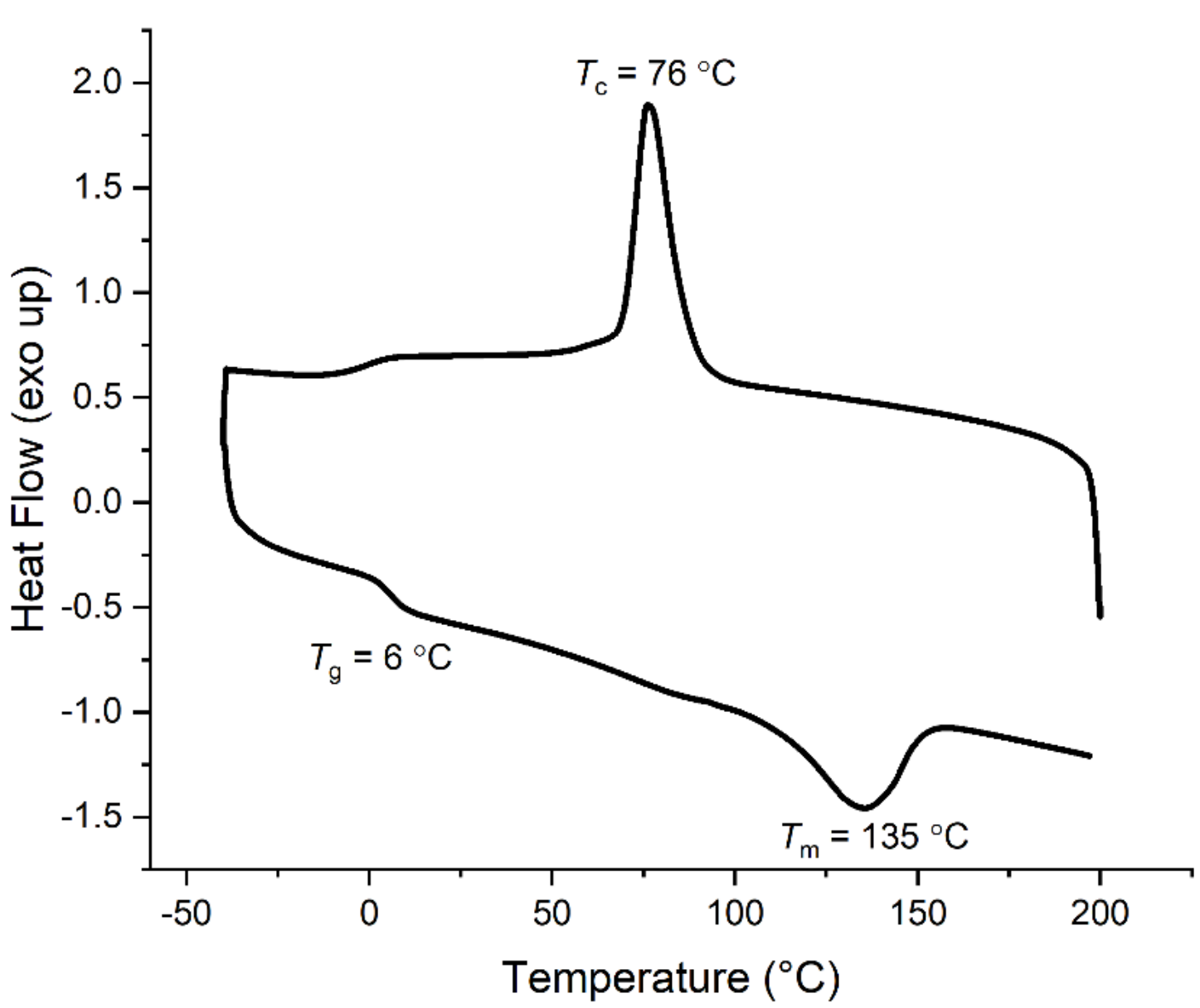

Figure S76. DSC curves of 1st cooling and 2nd heating scans of P(BL$^{Me}_{92}$-*co*-BL$^{allyl}_{8}$) (M_n = 202 kDa, $Đ$ = 1.05). Scan rate: 10 °C/min.

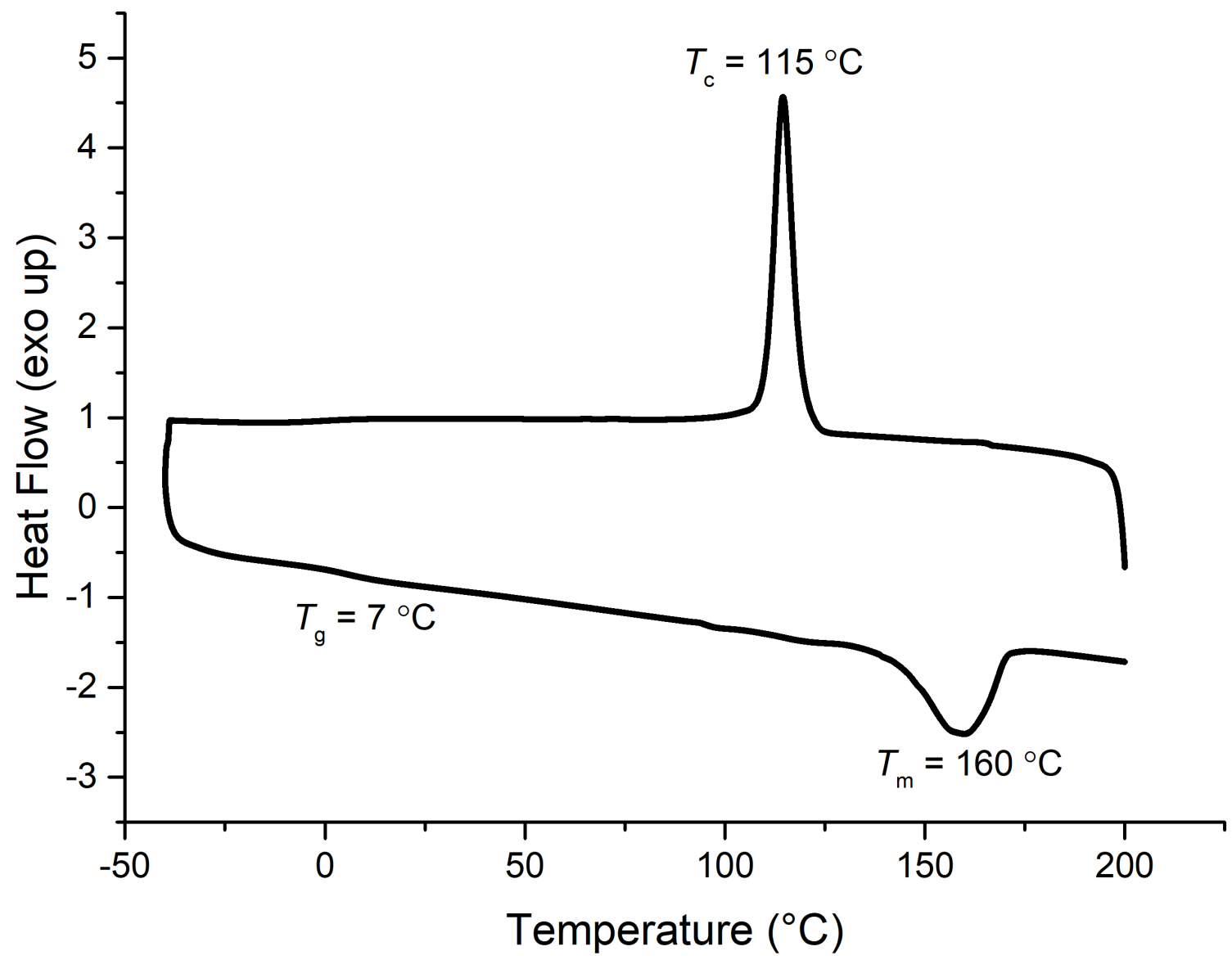

Figure S77. DSC curves of 1st cooling and 2nd heating scans of P($BL^{Me}{}_{92}$-*co*-$BL^{allyl}{}_{8}$) prepared with **Y4** (M_n = 121 kDa, $Đ$ = 1.01). Scan rate: 10 °C/min.

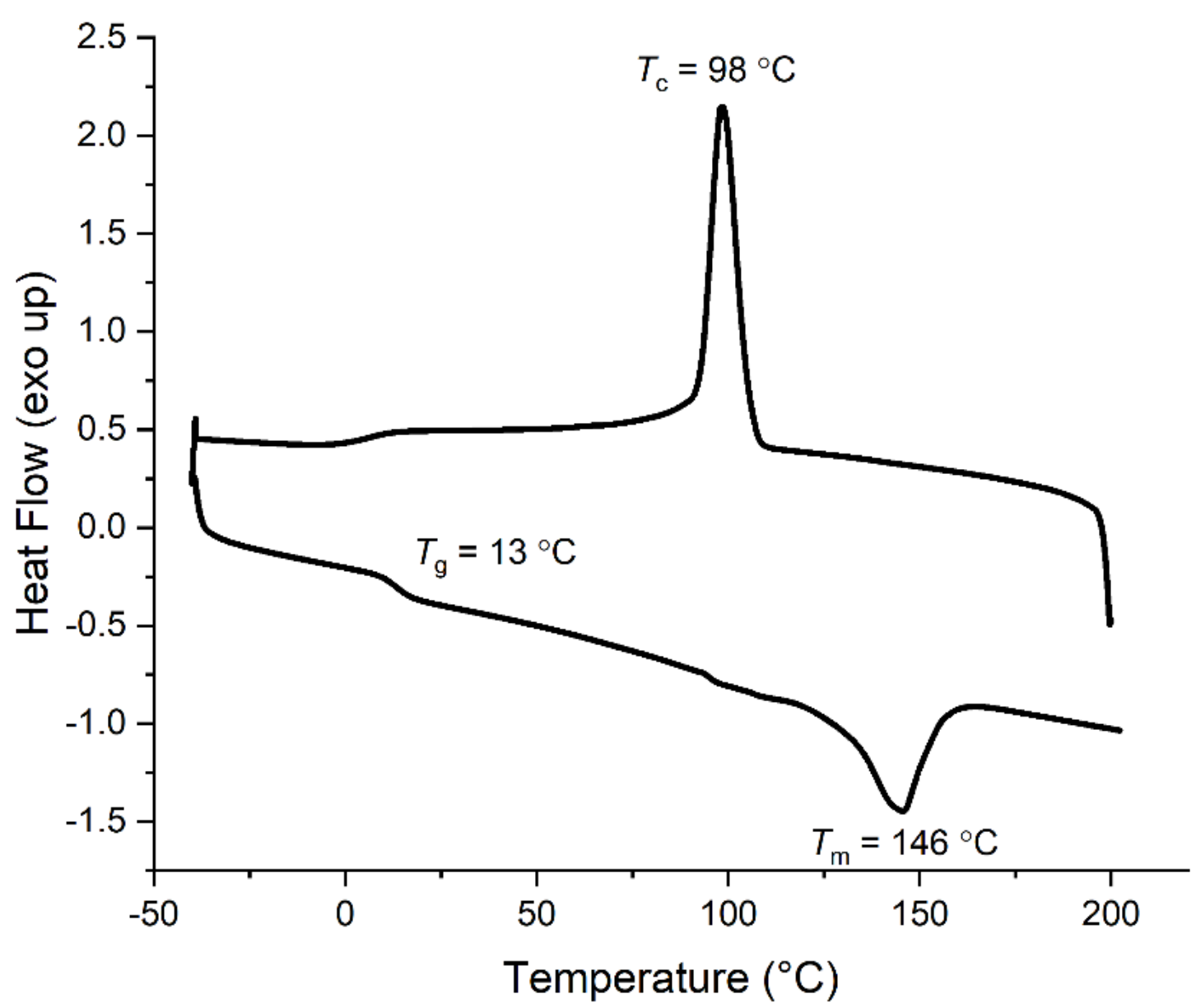

Figure S78. DSC curves of 1st cooling and 2nd heating scans of P($BL^{Me}{}_{95}$-*co*-$BL^{hPg}{}_{5}$) (M_n = 90.5 kDa, $Đ$ = 1.08). Scan rate: 10 °C/min.

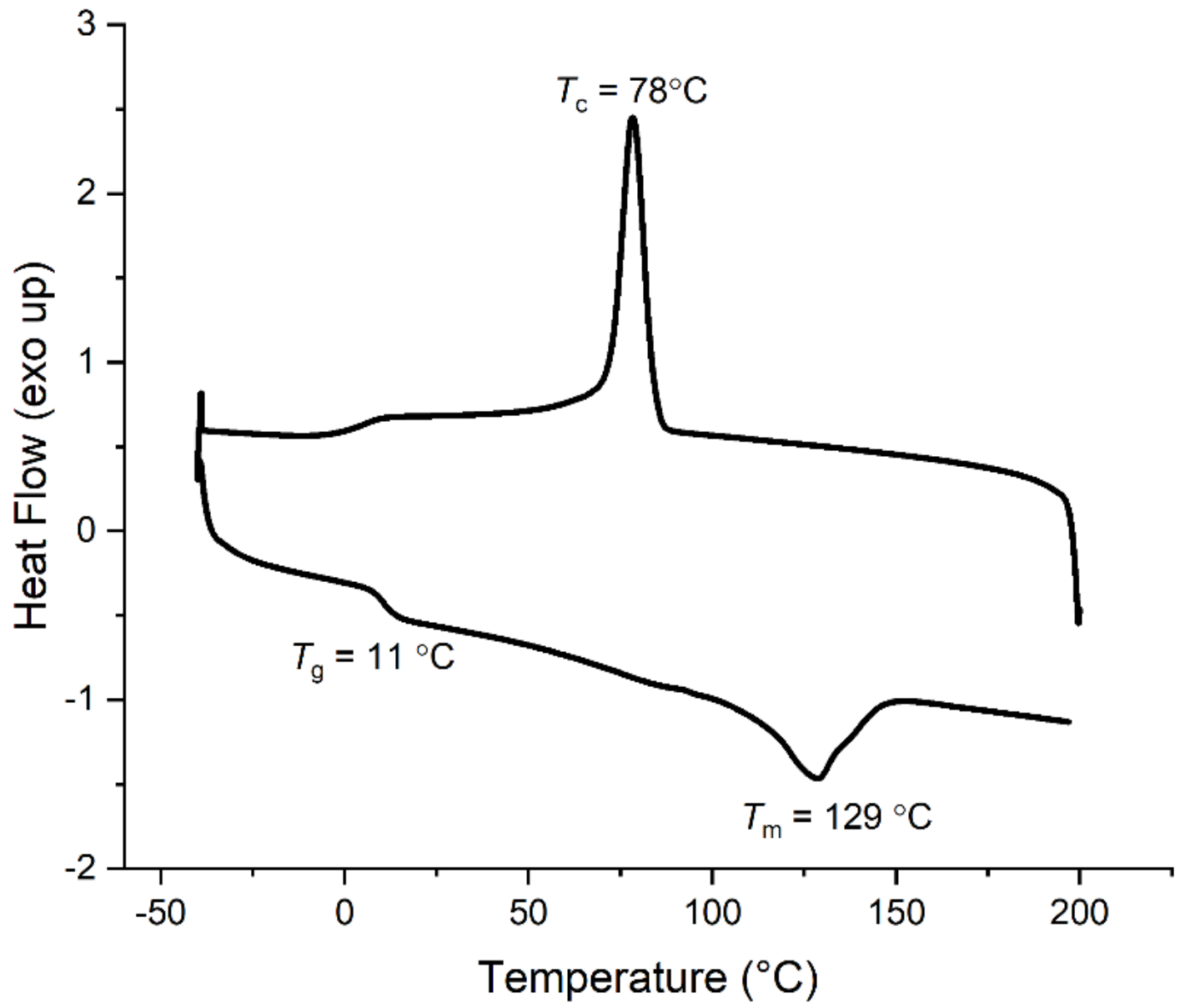

Figure S79. DSC curves of 1st cooling and 2nd heating scans of P(BL^{Me}$_{90}$-*co*-BL^{hPg}$_{10}$) (M_n = 79.8 kDa, $Đ$ = 1.07). Scan rate: 10 °C/min.

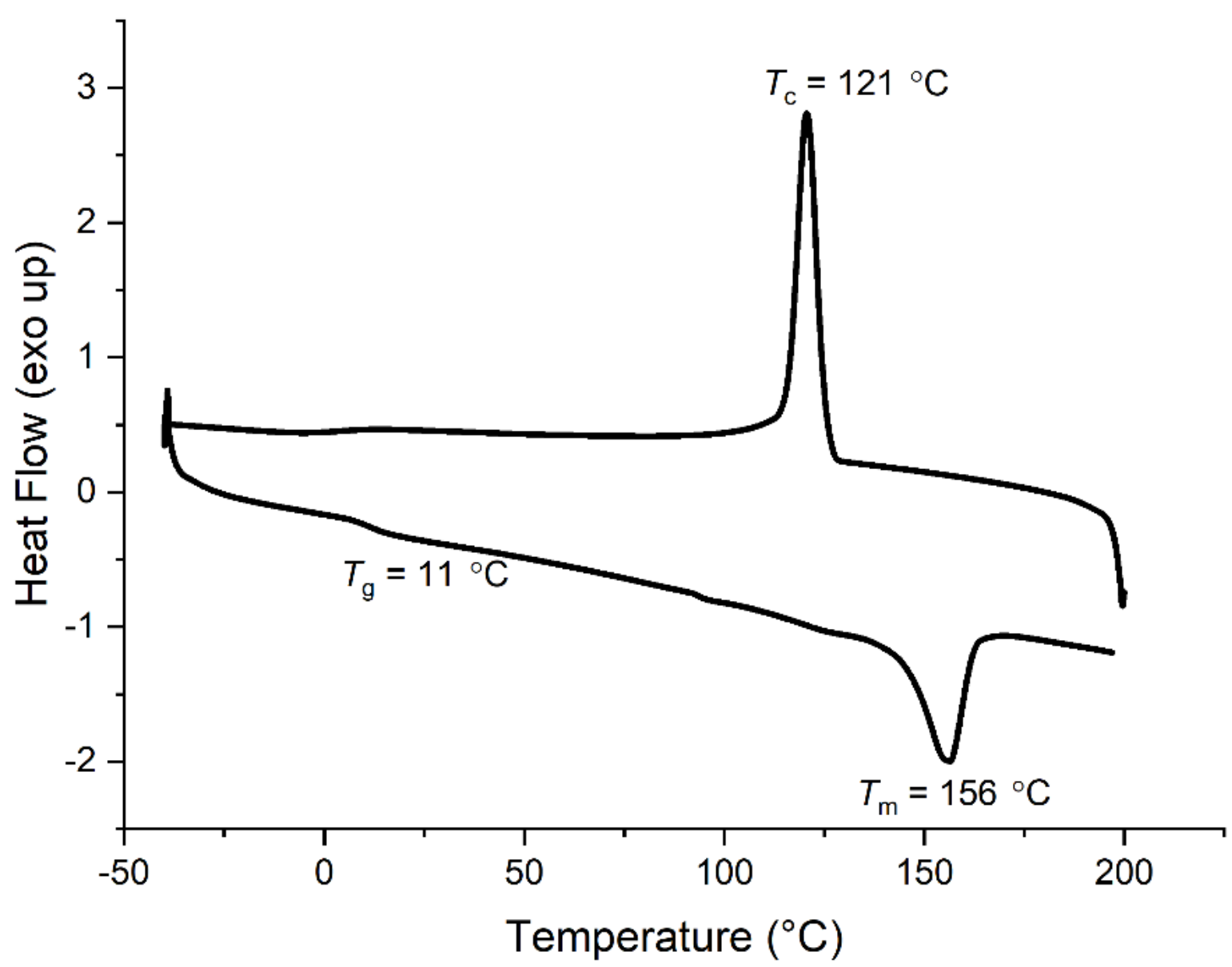

Figure S80. DSC curves of 1st cooling and 2nd heating scans of P3HB (M_n = 56.0 kDa, $Đ$ = 1.06, P_r = 0.84). Scan rate: 10 °C/min.

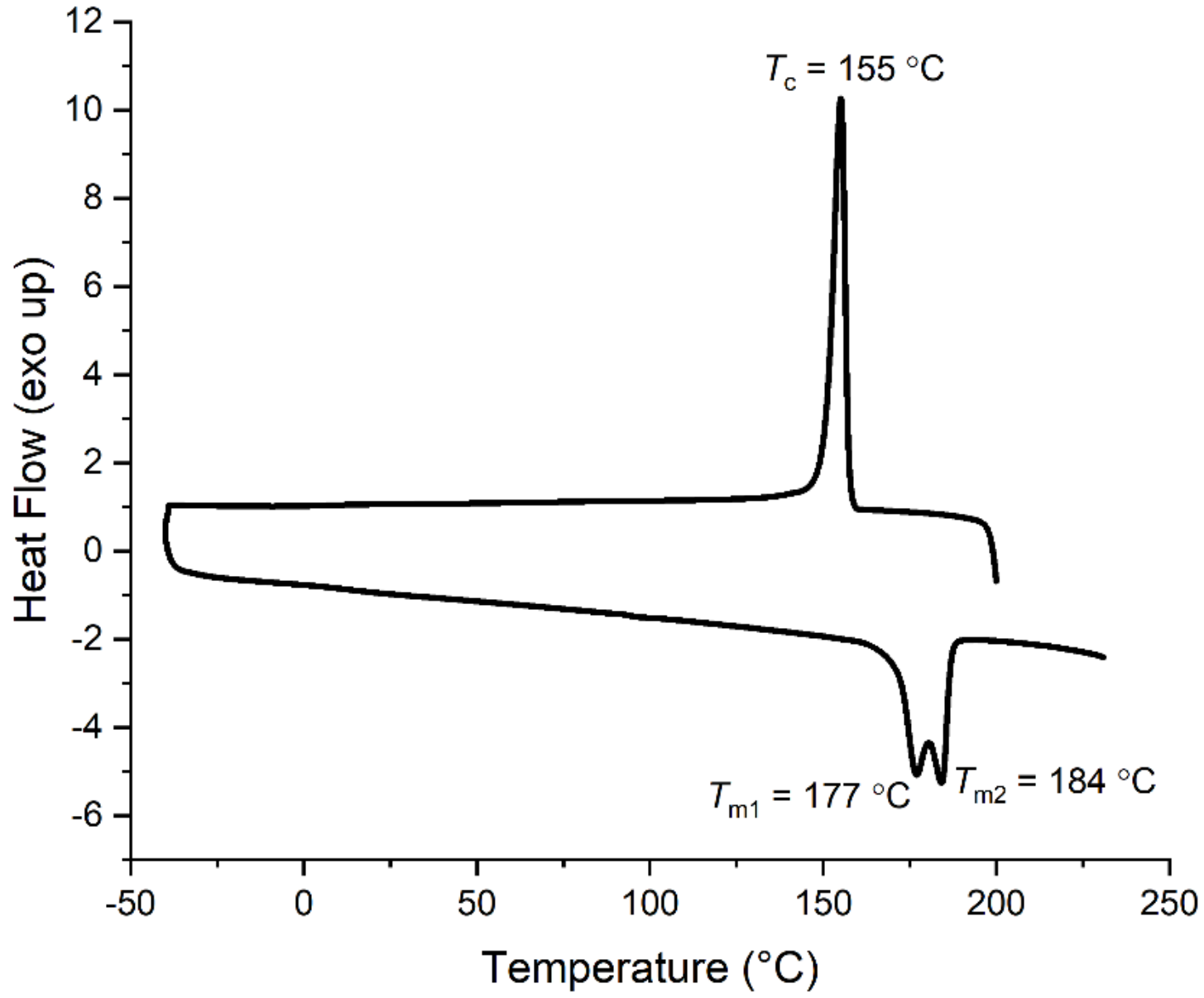

Figure S81. DSC curves of 1st cooling and 2nd heating scans of P3HB (M_n = 11.3 kDa, $Đ$ = 1.20, P_r = 0.91). Scan rate: 10 °C/min.

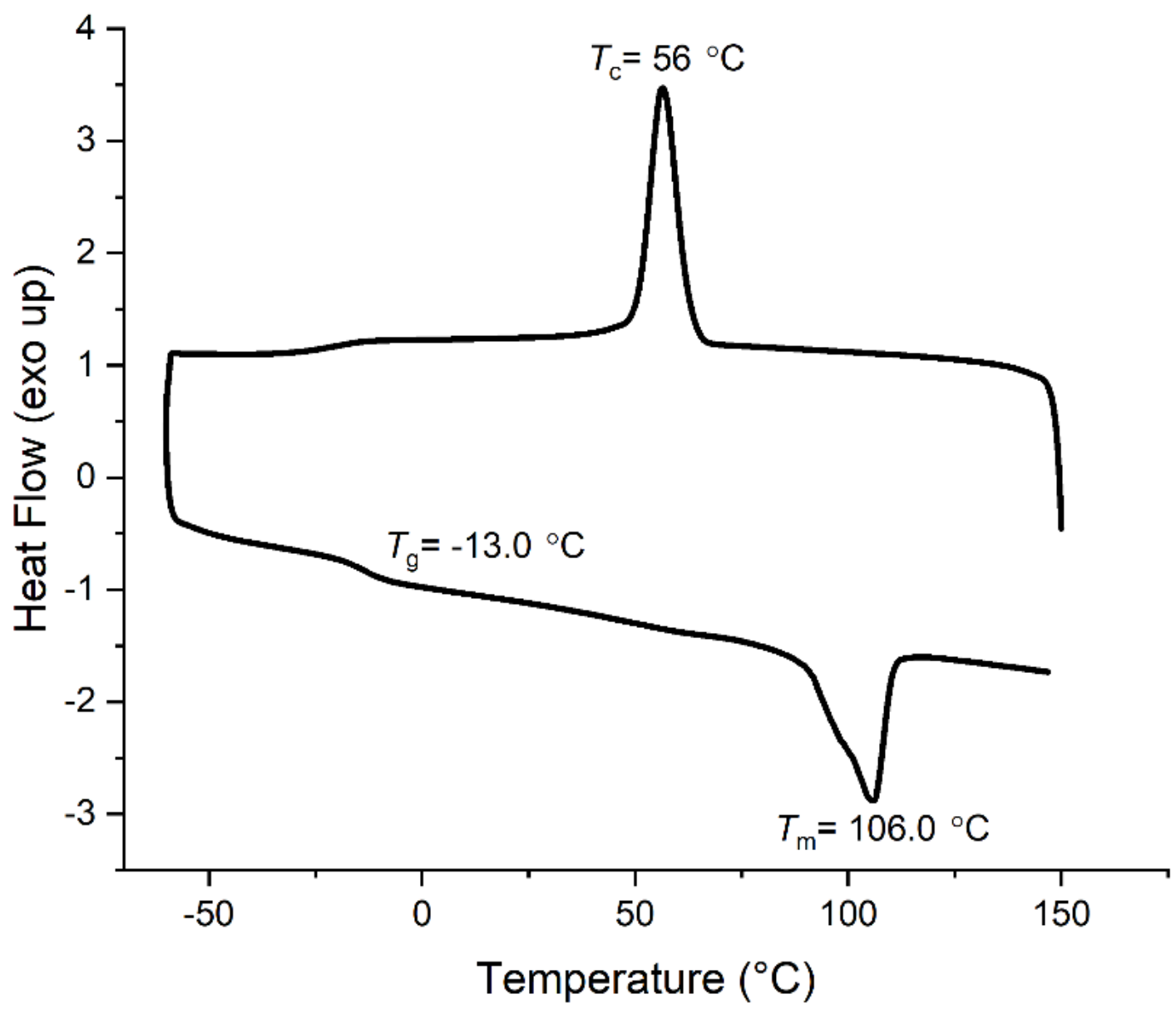

Figure S82. DSC curves of 1st cooling and 2nd heating scans of P(BL^{vinyl})×2%. Scan rate: 10 °C/min.

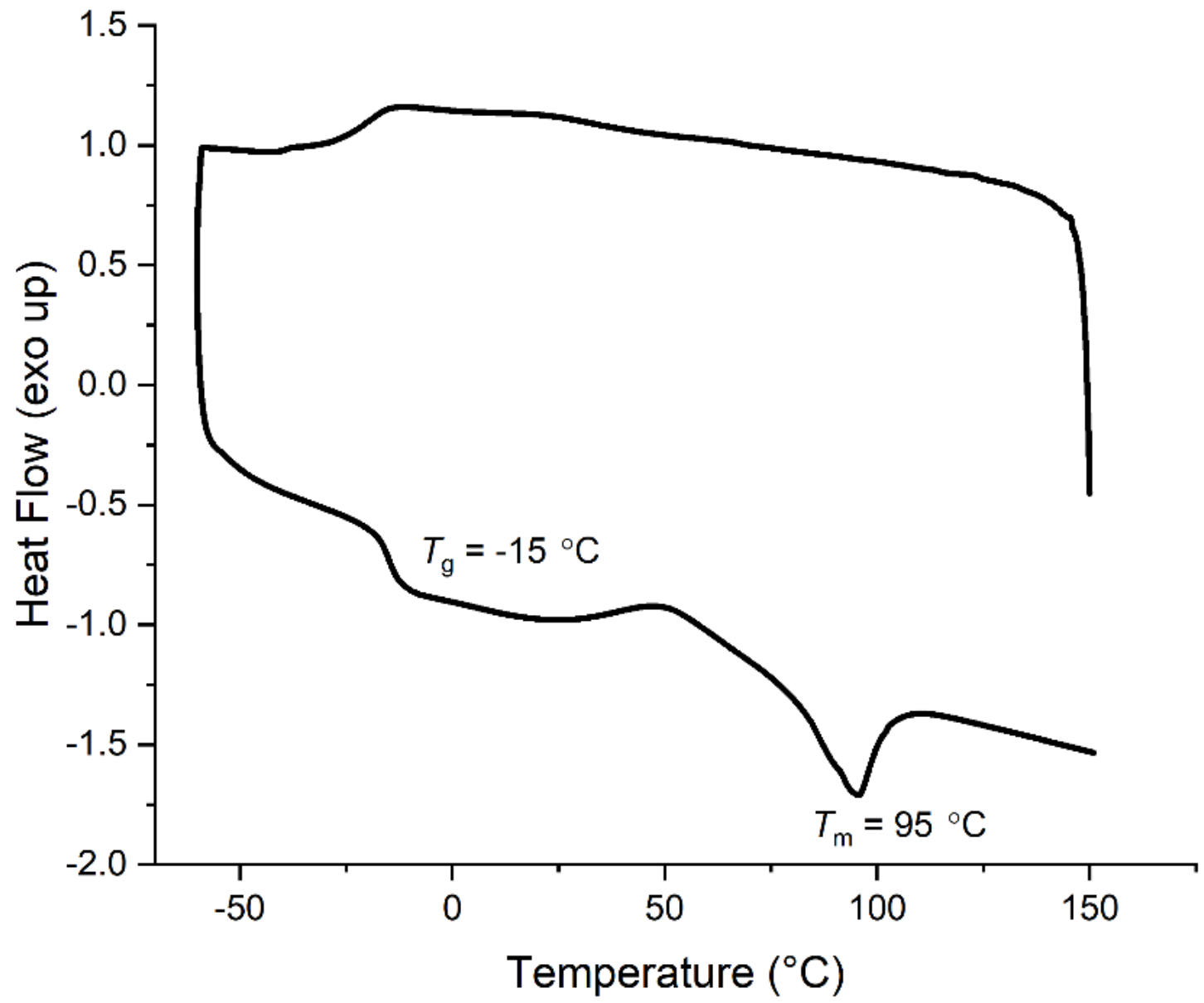

Figure S83. DSC curves of 1st cooling and 2nd heating scans of P(BL^{vinyl})×5%. Scan rate: 10 °C/min.

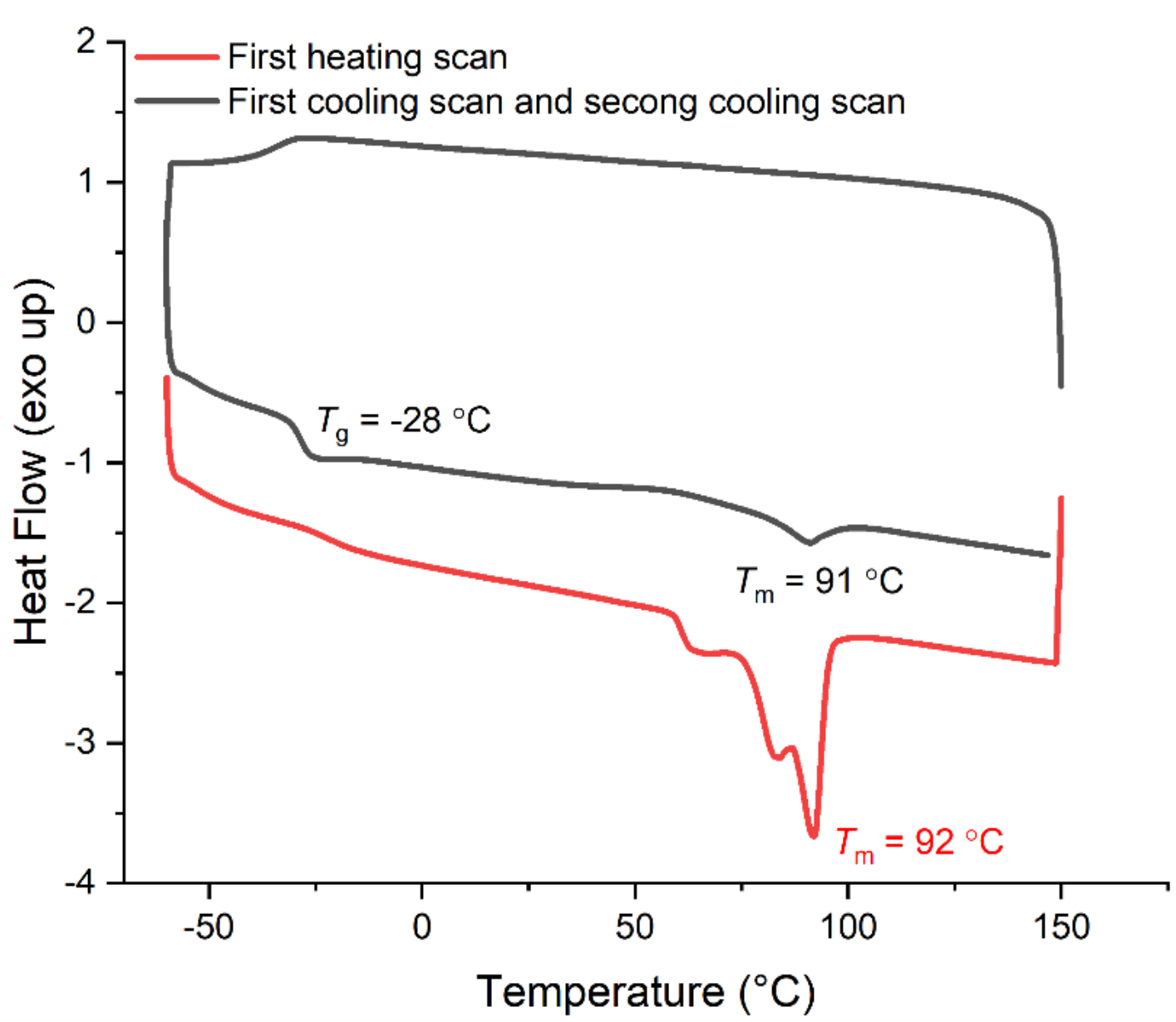

Figure S84. DSC curves of P(BL^{allyl})×2%. Scan rate: 10 °C/min.

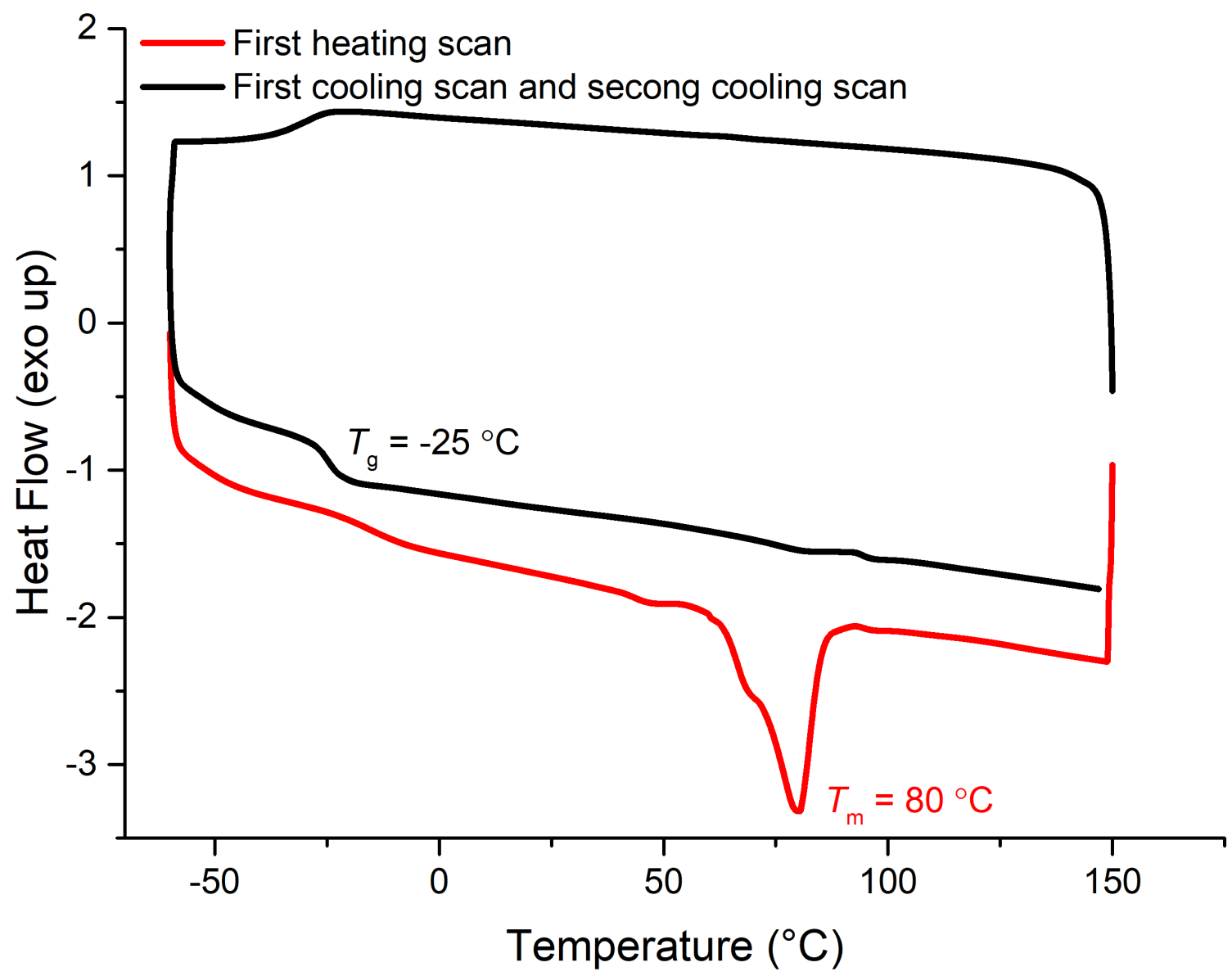

Figure S85. DSC curves of P(BL^{allyl})×5%. Scan rate: 10 °C/min.

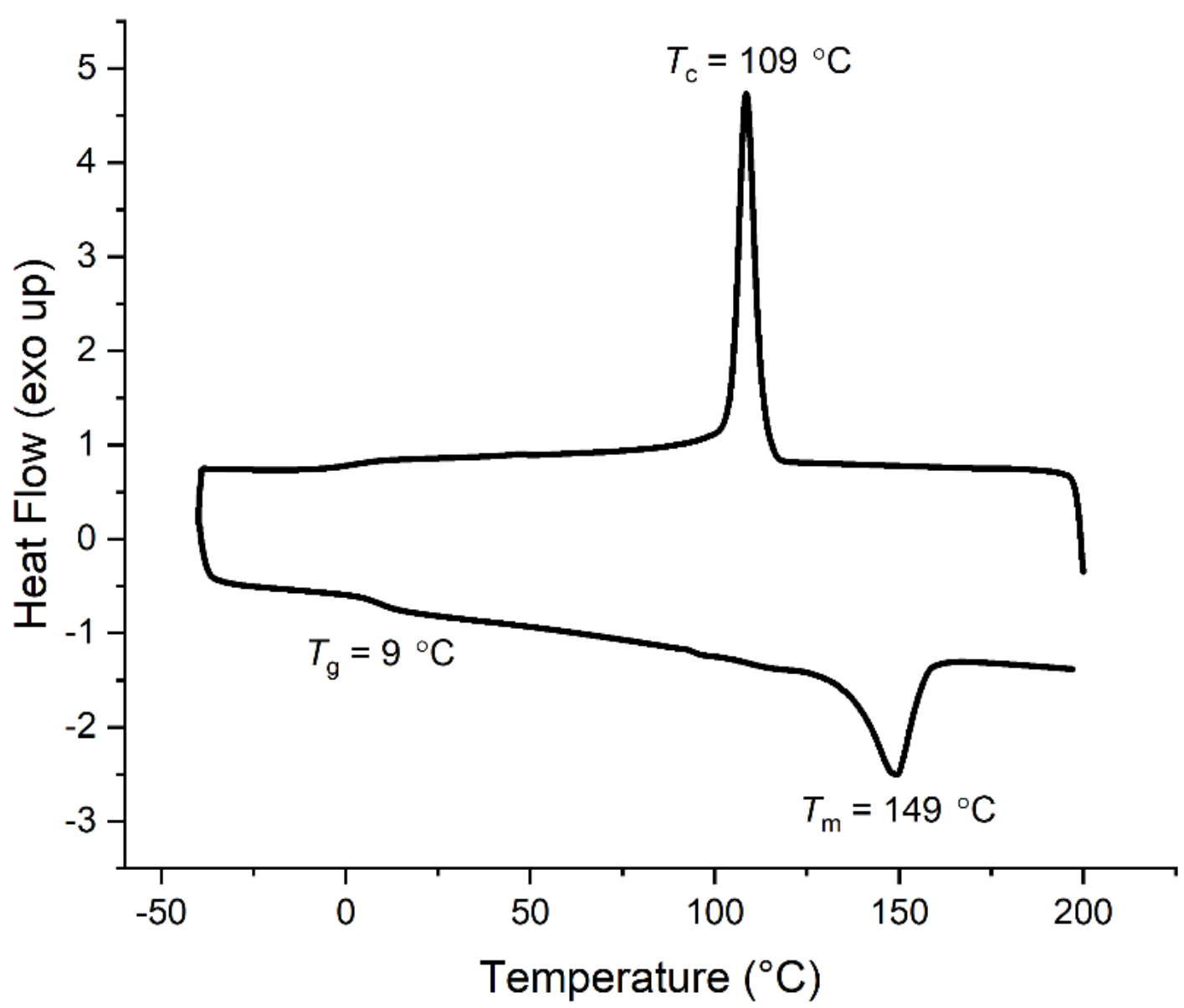

Figure S86. DSC curves of 1st cooling and 2nd heating scans of P(BL$^{Me}{}_{96}$-*co*-BL$^{vinyl}{}_{4}$)-PETMP. Scan rate: 10 °C/min.

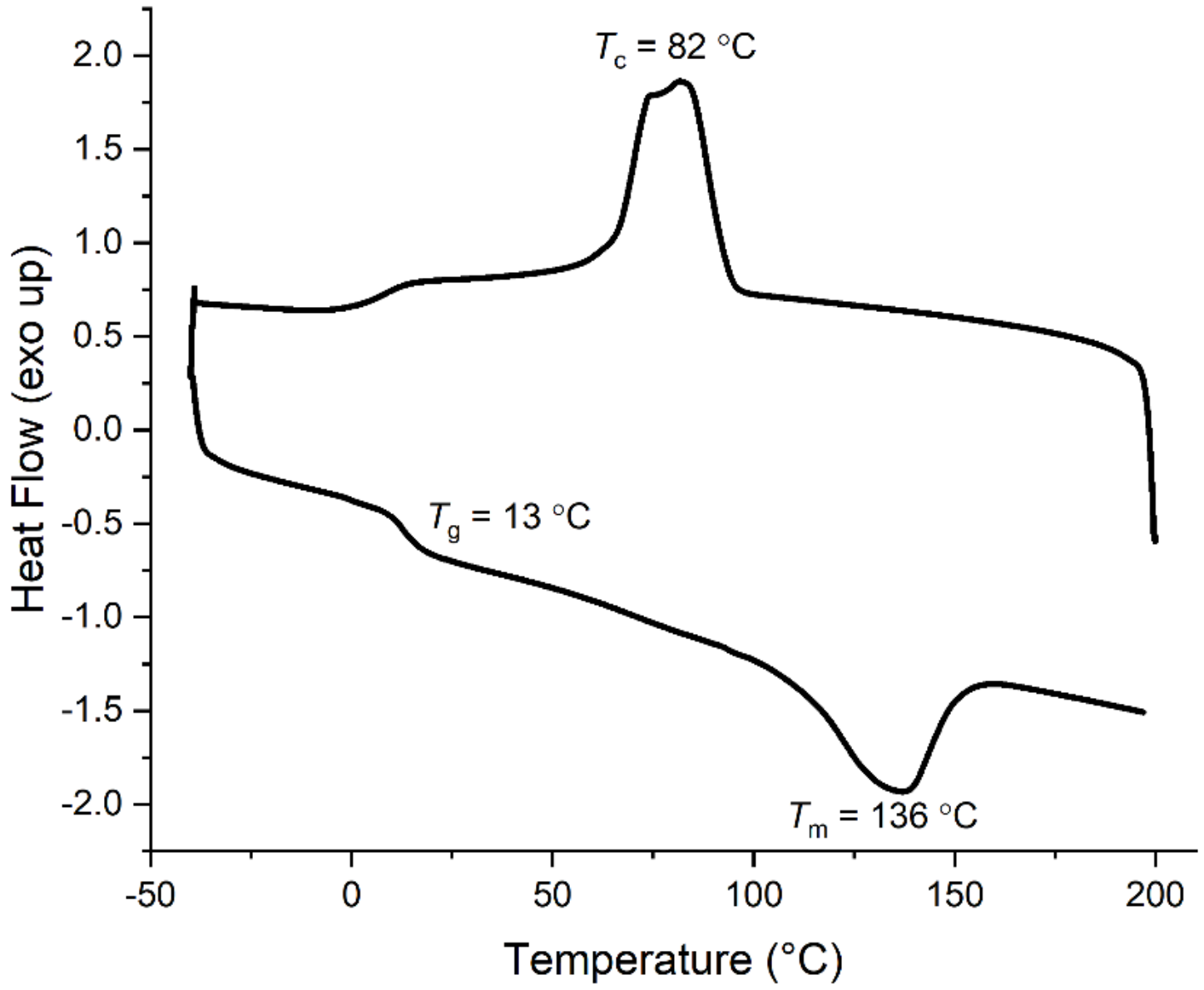

Figure S87. DSC curves of 1st cooling and 2nd heating scans of P(BL^{Me}_{96}-*co*-BL^{allyl}_{4})-PETMP. Scan rate: 10 °C/min.

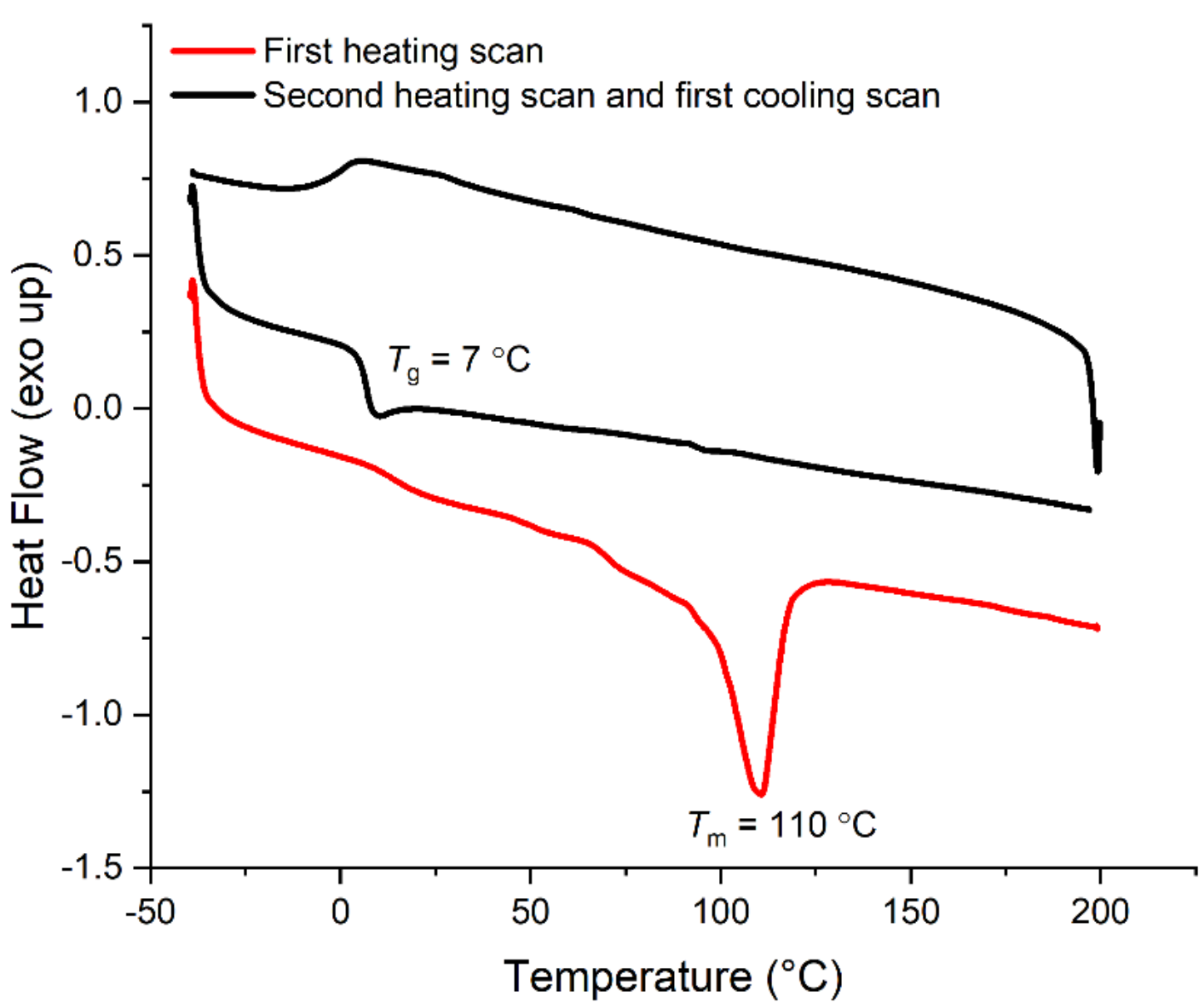

Figure S88. DSC curves of P(BL^{Me}_{90}-*co*-BL^{allyl}_{10})-UPy. Scan rate: 10 °C/min

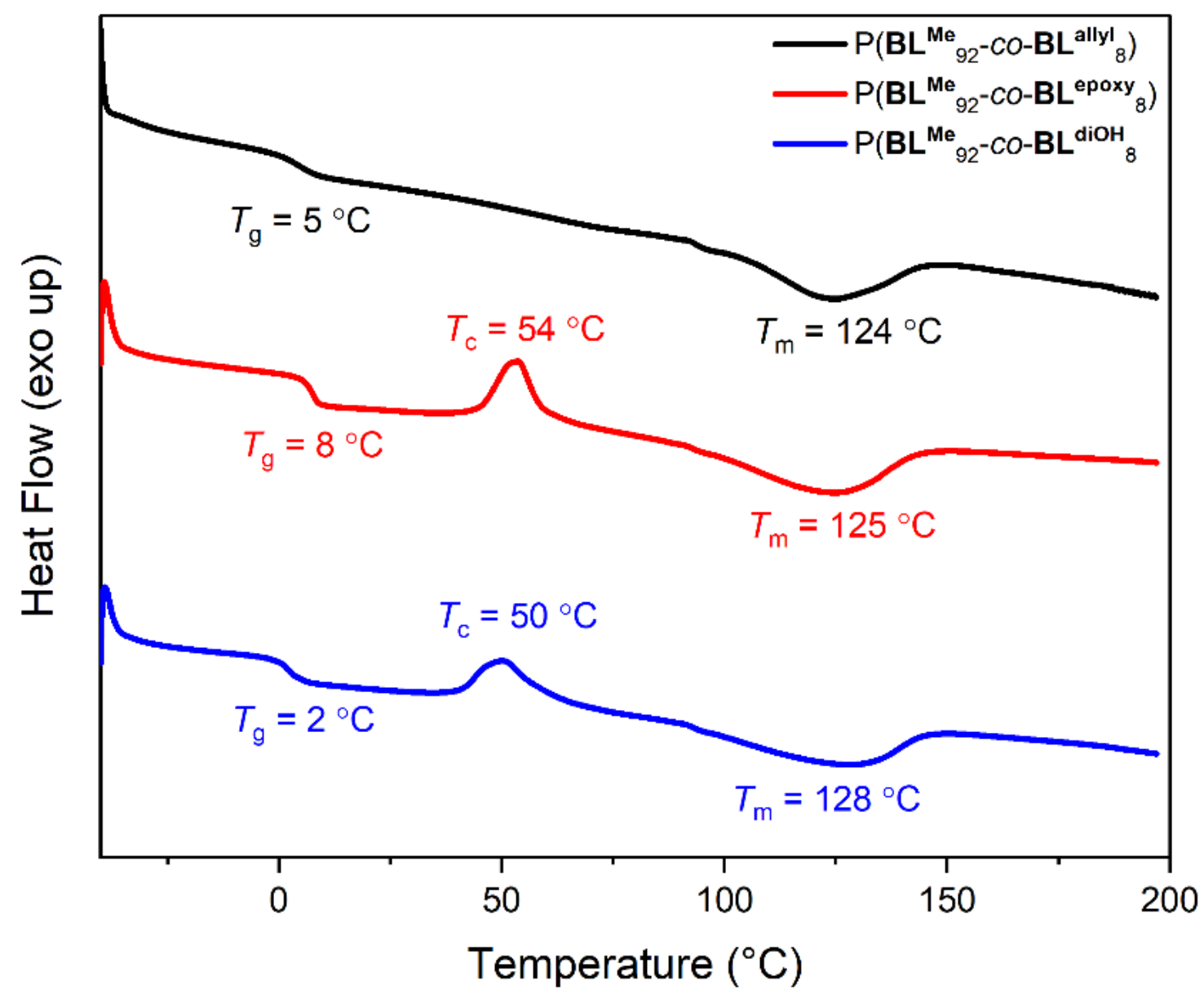

Figure S89. Overlayed DSC curves of 2nd heating scans for P(BL^{Me}$_{92}$-*co*-BL^{allyl}$_{8}$), P(BL^{Me}$_{92}$-*co*-BL^{epoxy}$_{8}$) and P(BL^{Me}$_{92}$-*co*-BL^{diOH}$_{8}$). Scan rate: 10 °C/min.

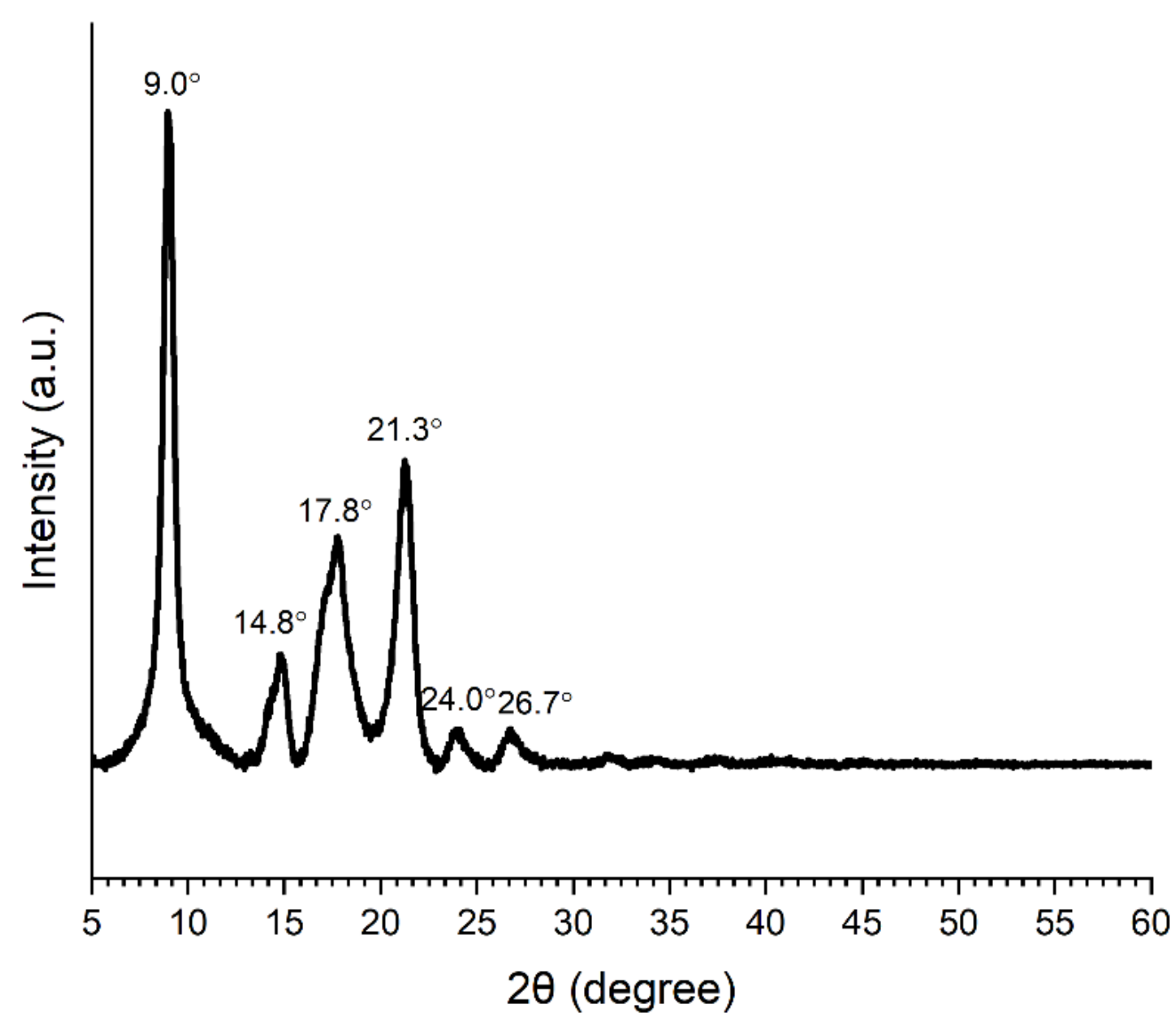

Figure S90. pXRD profile of P(BL^{vinyl}), $P_r = 0.91$.

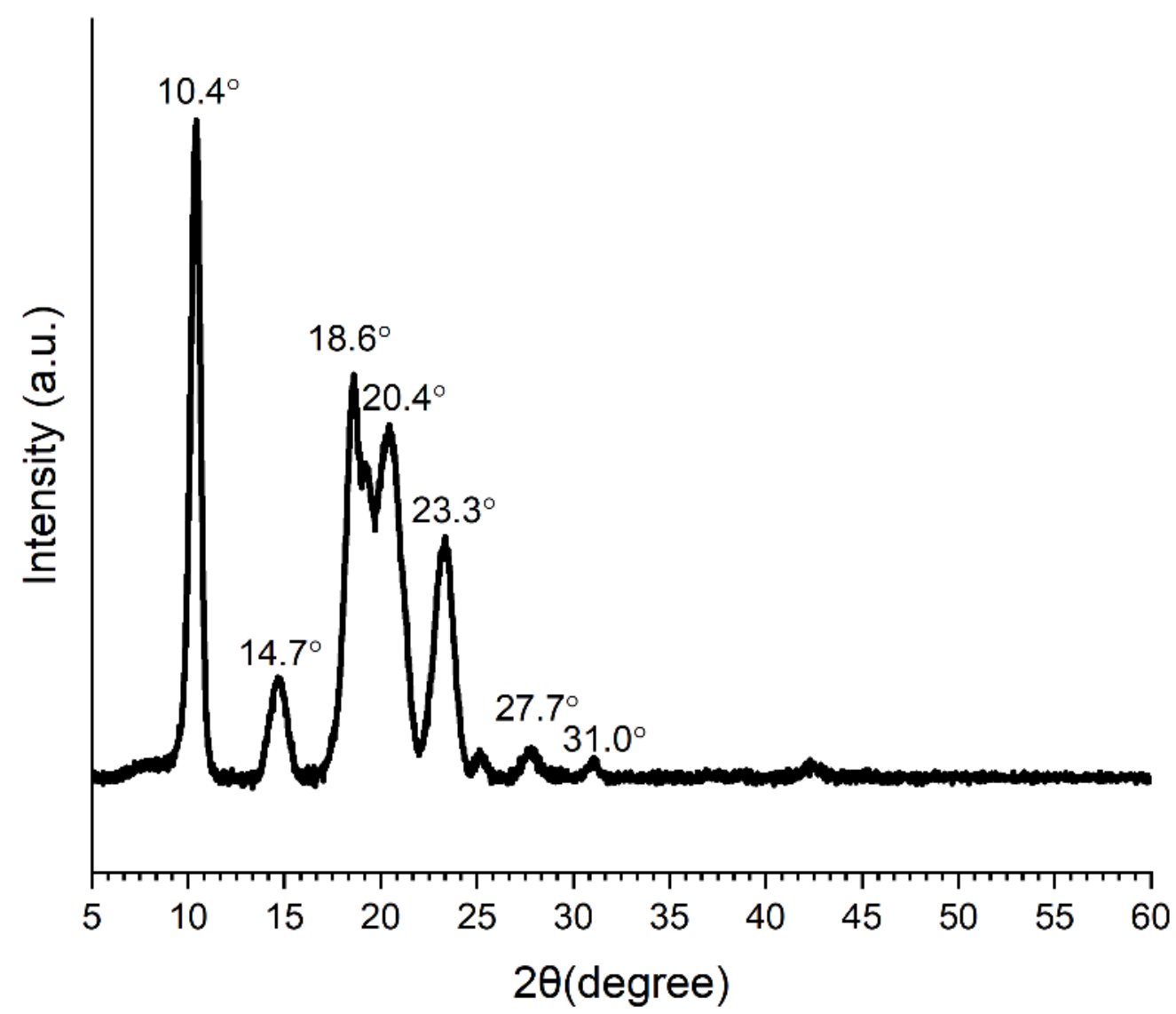

Figure S91. pXRD profile of P(BL^{allyl}), P_r = 0.93.

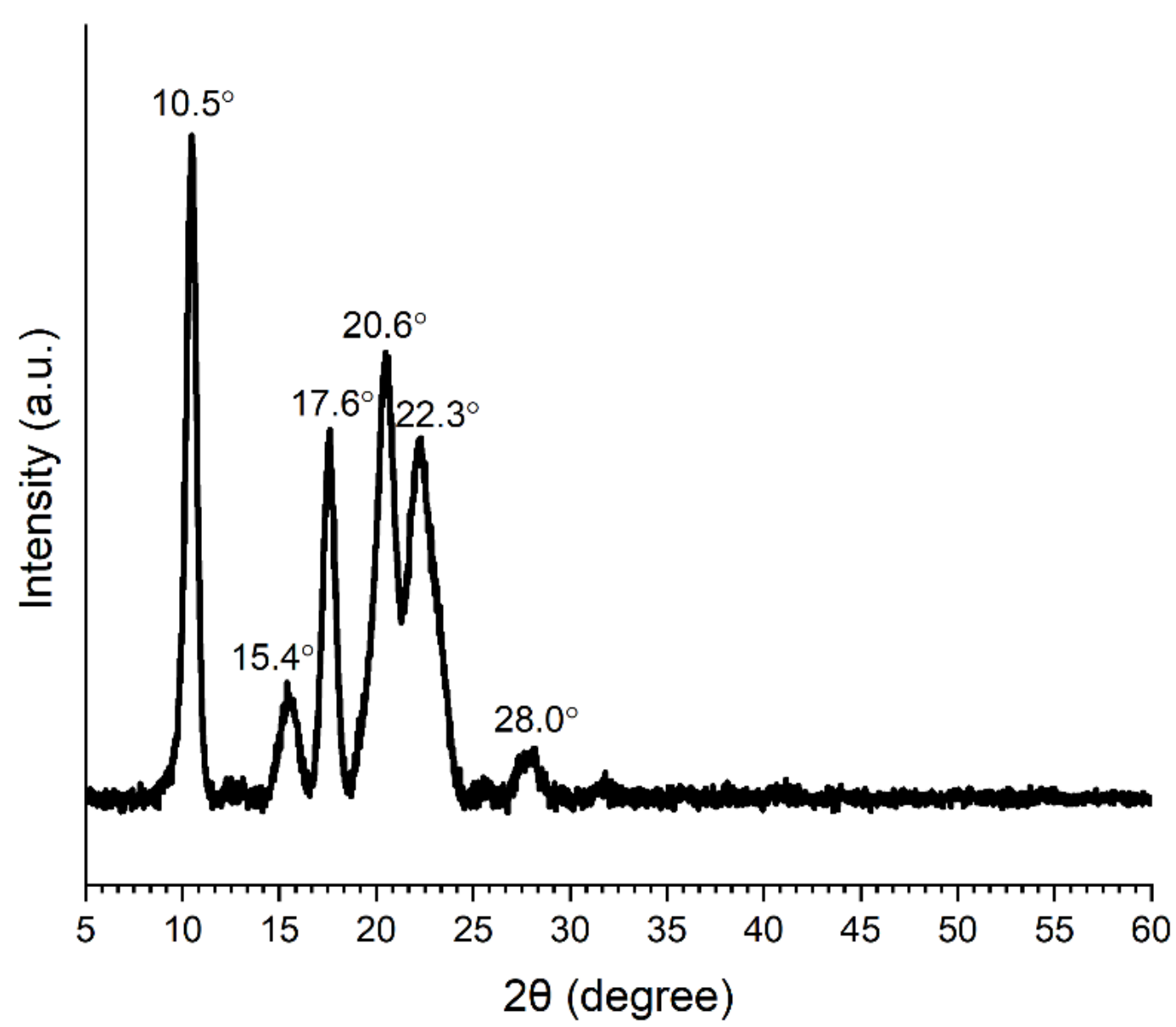

Figure S92. pXRD profile of P(BL^{hPg}), P_r = 0.91.

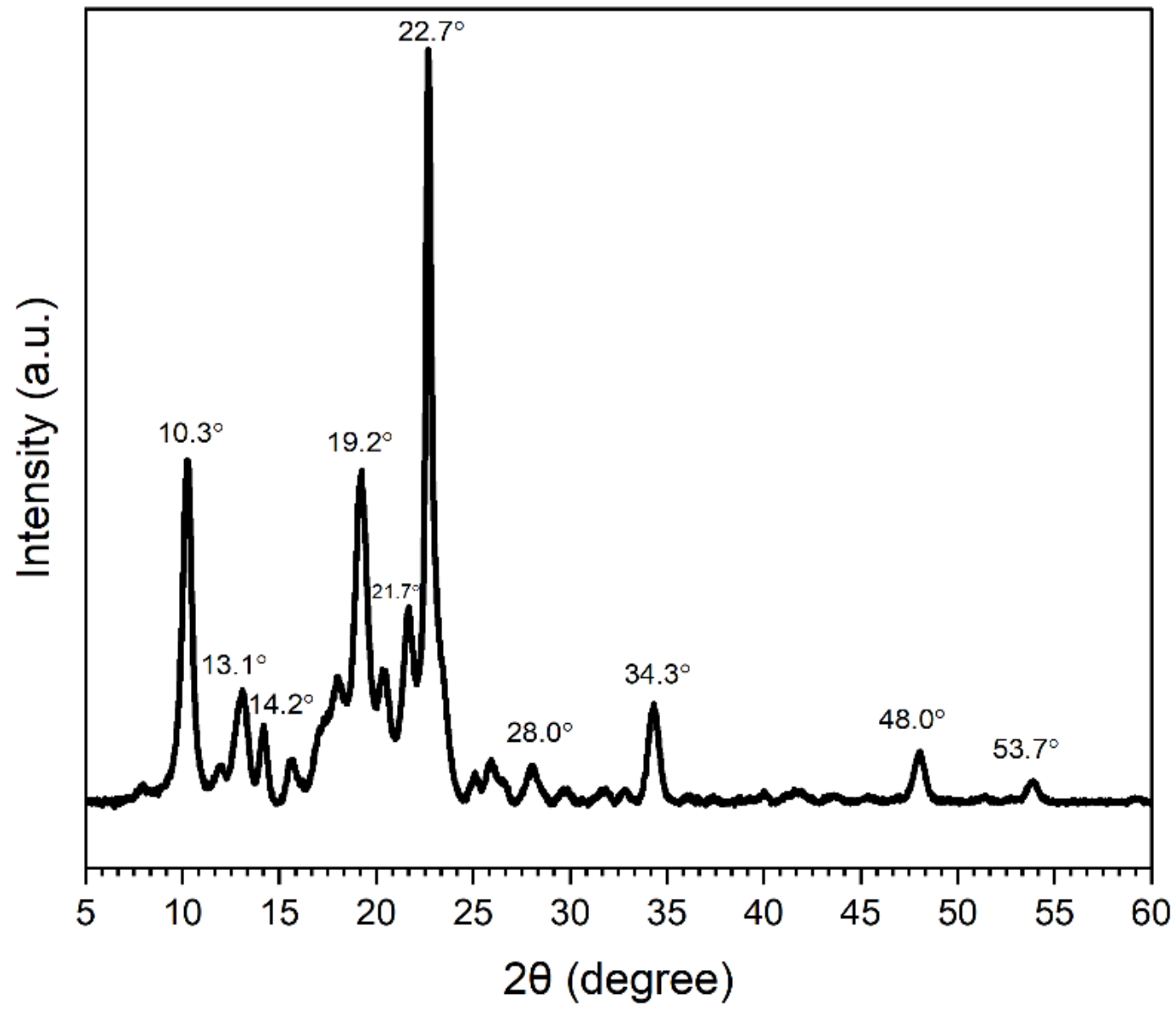

Figure S93. pXRD profile of *sc*-P(BL^{vinyl}).

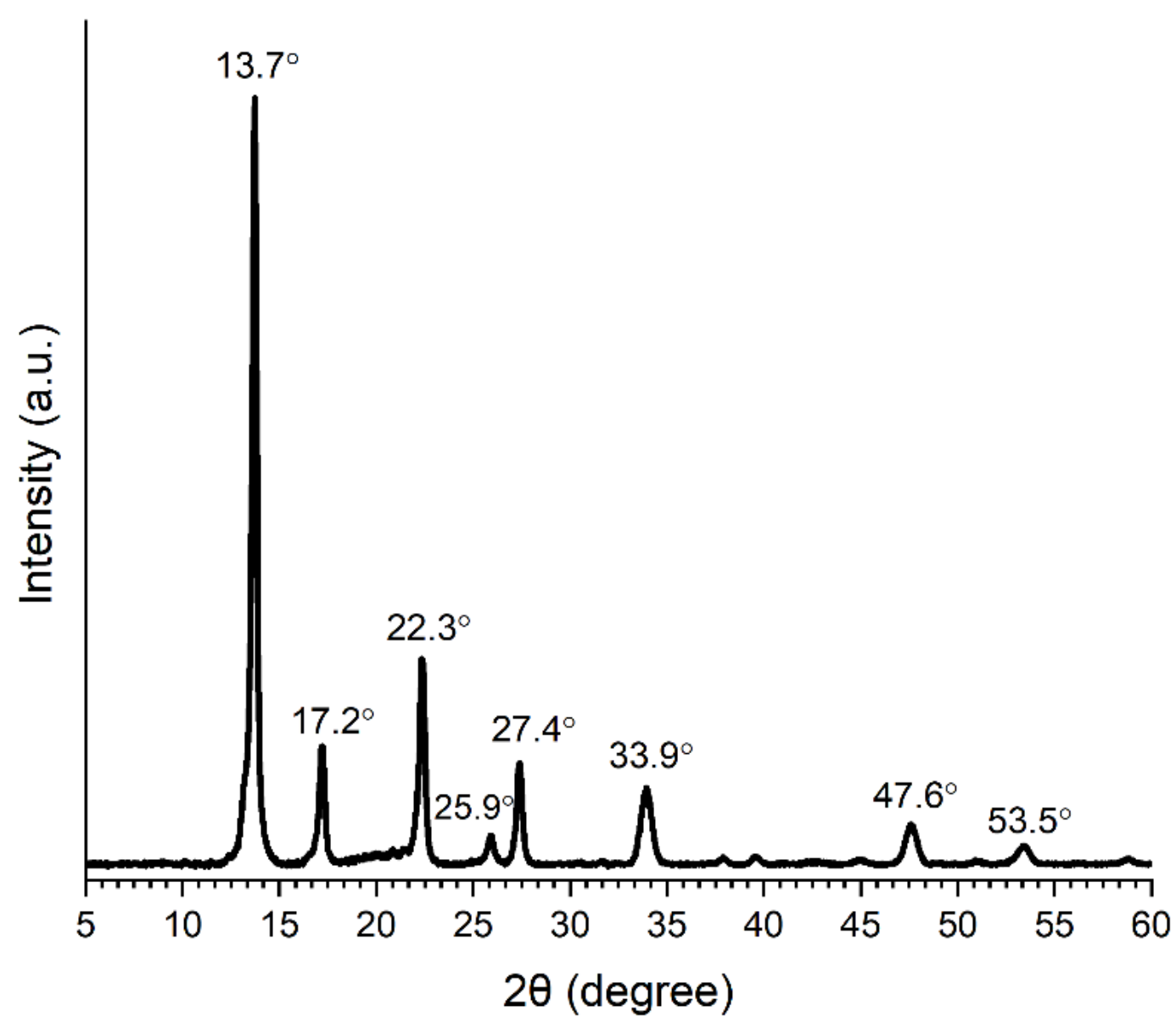

Figure S94. pXRD profile of P(BL$^{Me}_{90}$-*co*-BL$^{allyl}_{10}$)-UPy.

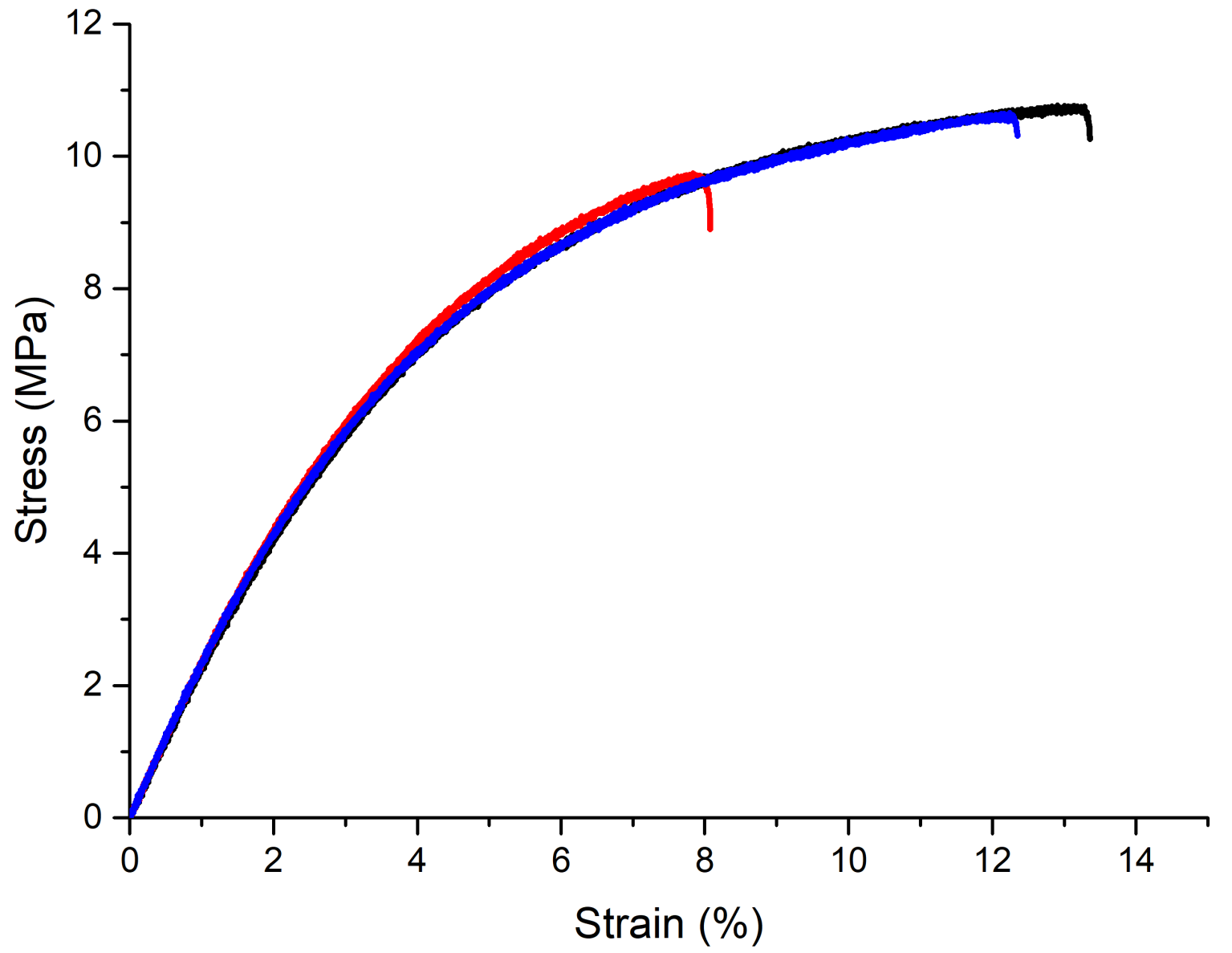

Figure S95. Tensile curves for P(BL^{vinyl}) (M_n = 42.6 kDa, $Đ$ = 1.02, P_r = 0.90), strain rate = 5 mm/min, ambient condition.

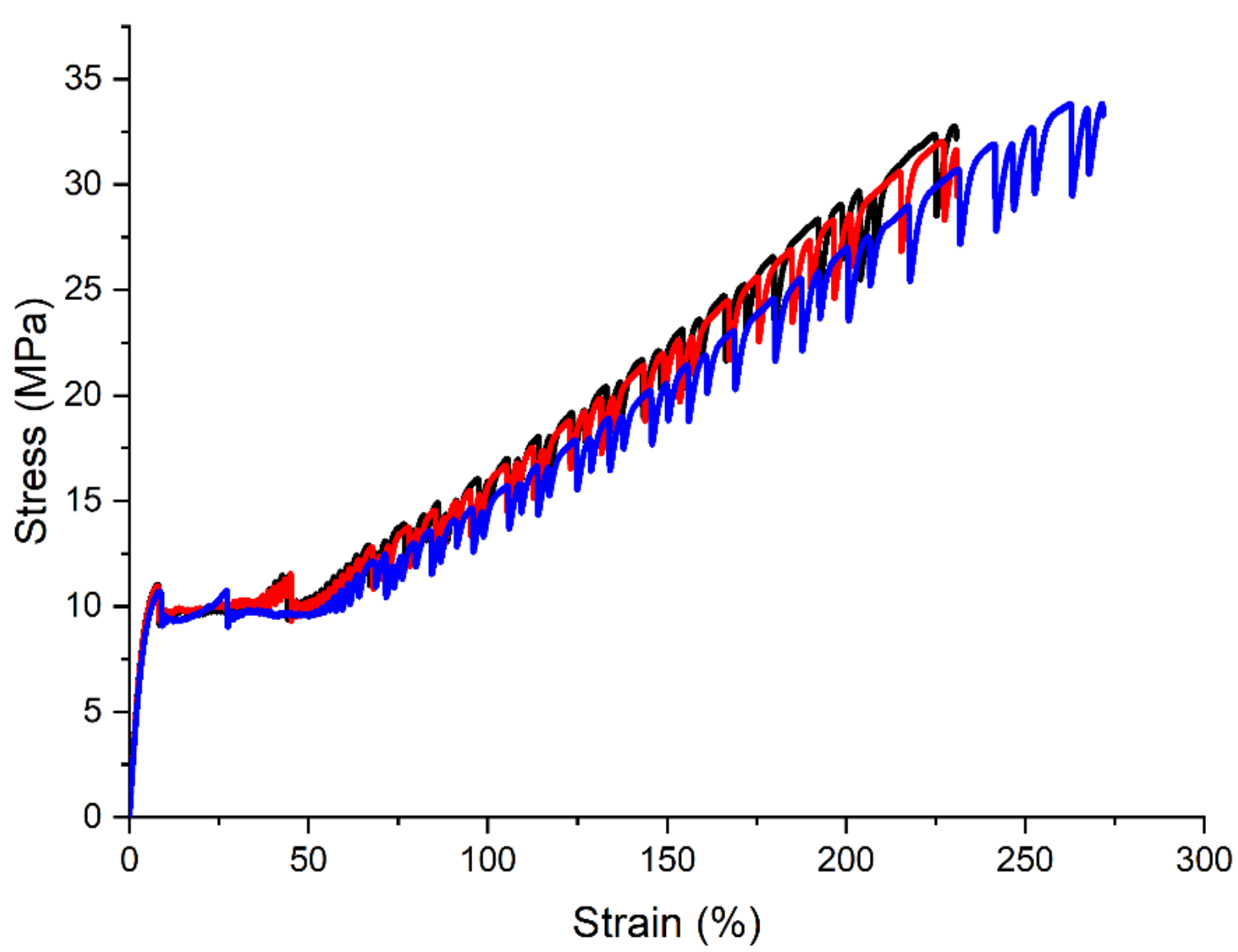

Figure S96. Tensile curves for P(BL^{allyl}) (M_n = 245 kDa, $Đ$ = 1.23, P_r = 0.95), strain rate = 5 mm/min, ambient condition.

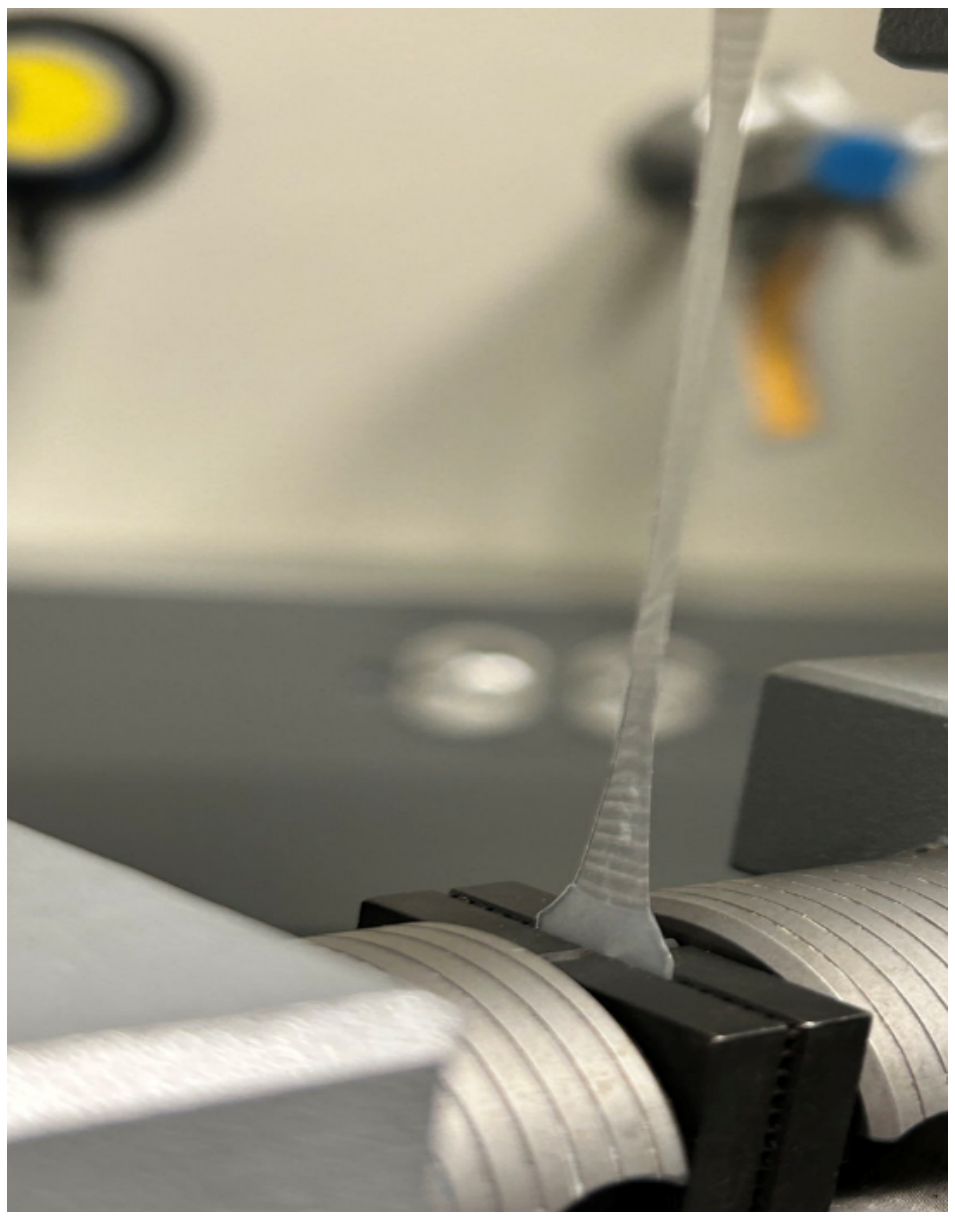

Figure S97. Photograph of a P(BL^{allyl}) dogbone specimen during tensile testing, showing periodic transparent/opaque banding in the neck region characteristic of the stress oscillation phenomenon.

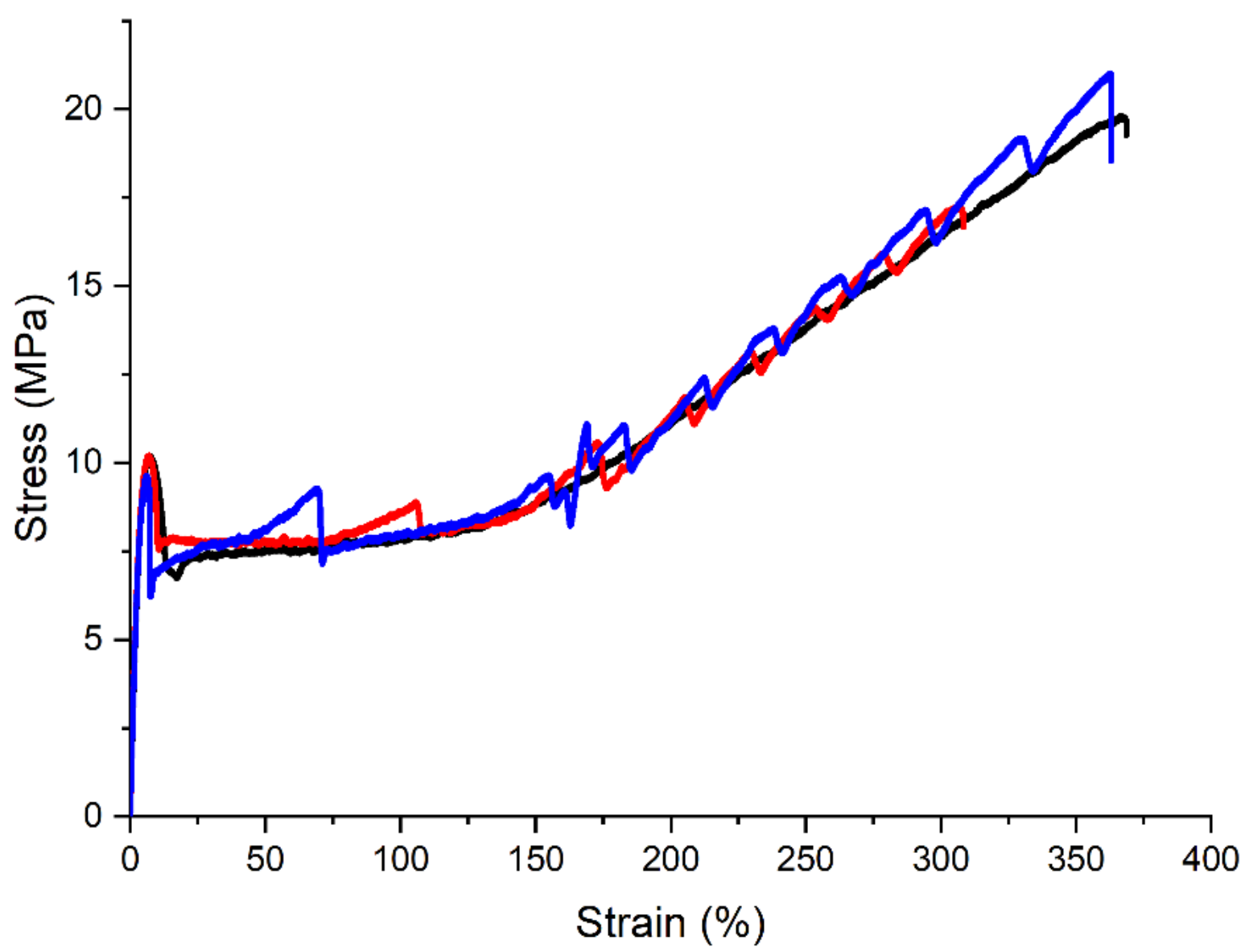

Figure S98. Tensile curves for P(BL^{hPg}) (M_n = 72.9 kDa, $Đ$ = 1.03, P_r = 0.91), strain rate = 5 mm/min, ambient condition.

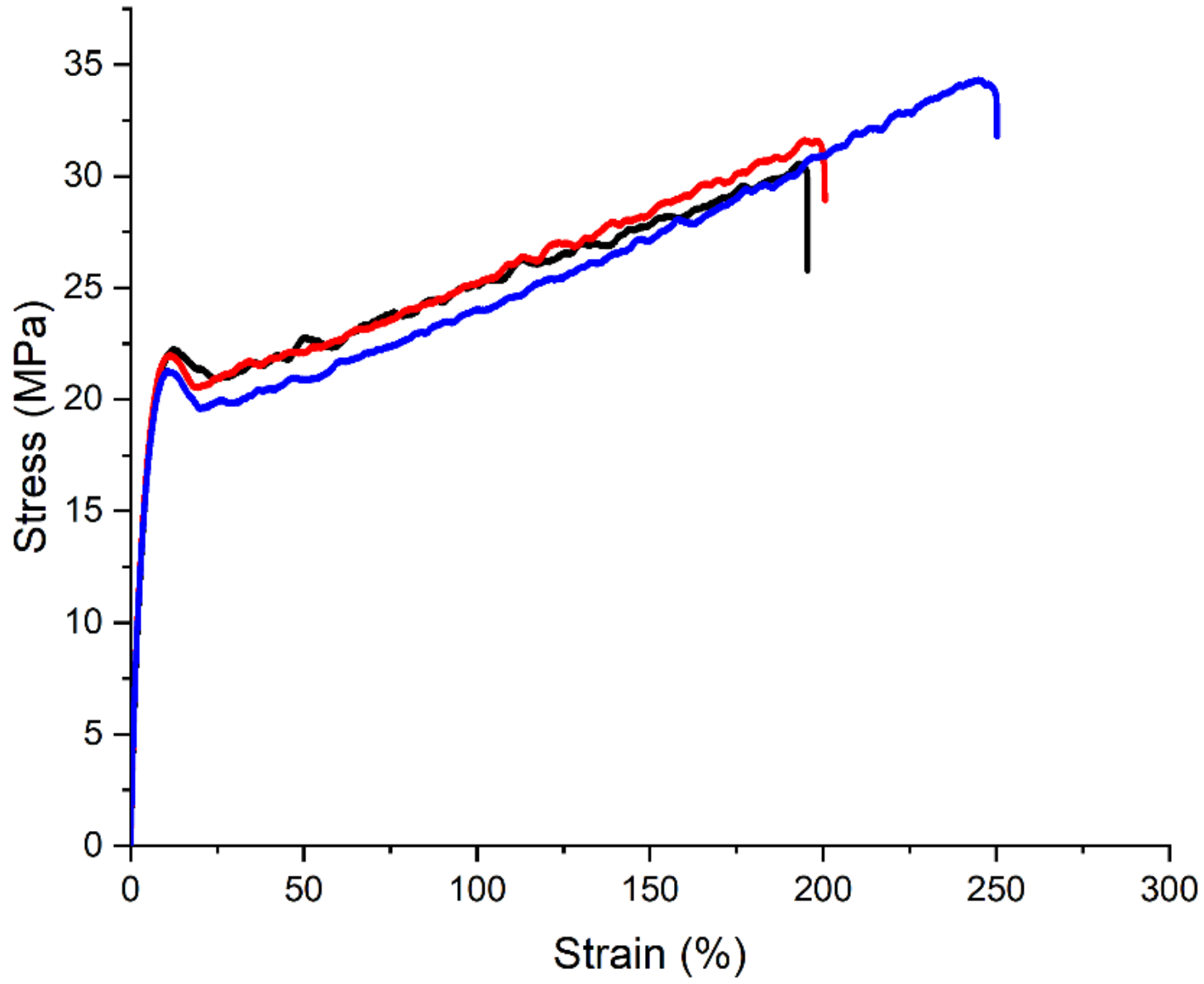

Figure S99. Tensile curves for P(BL^{Me}$_{95}$-*co*-BL^{vinyl}$_{5}$) (M_n = 169 kDa, $Đ$ = 1.08), strain rate = 5 mm/min, ambient condition.

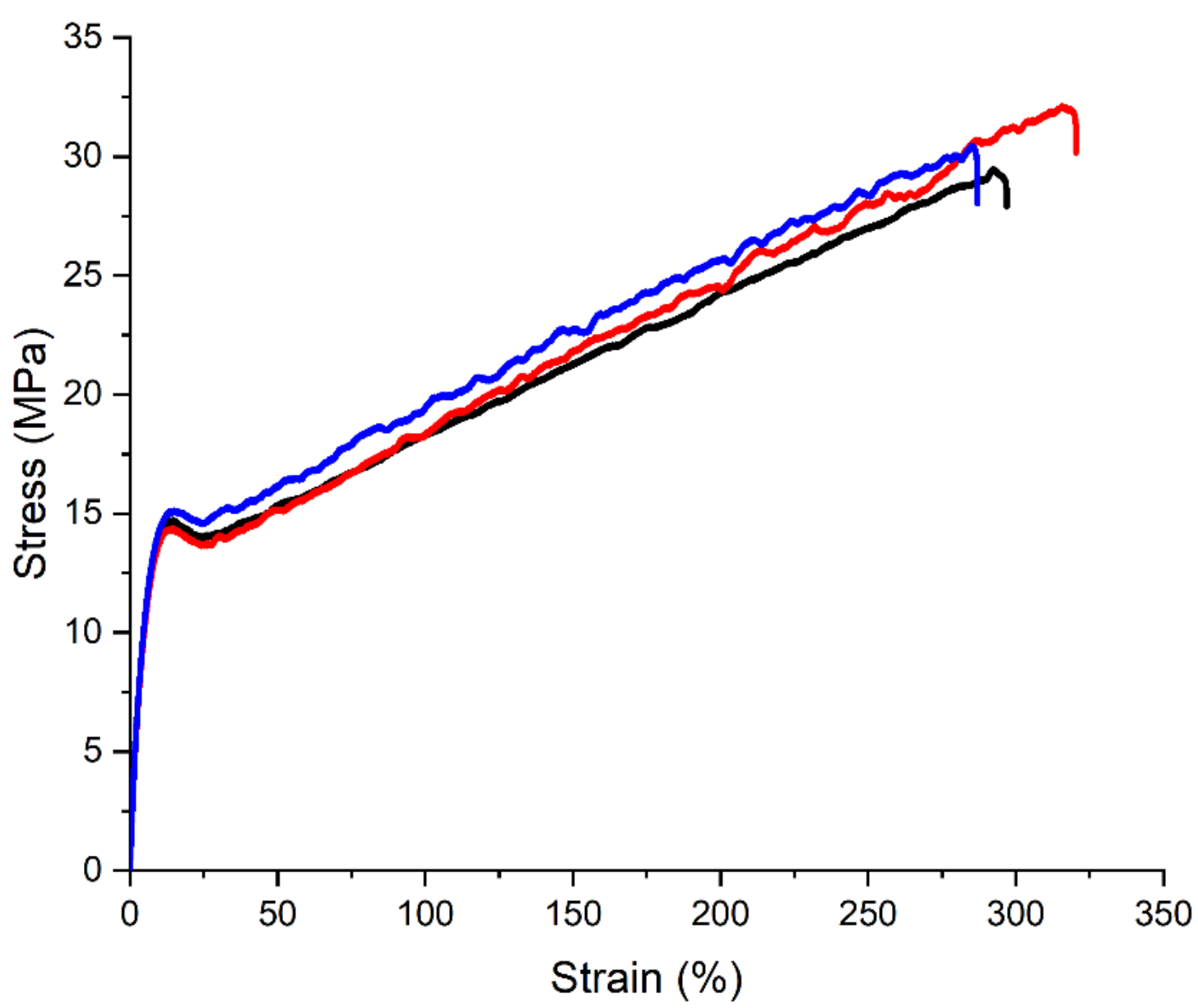

Figure S100. Tensile curves for P(BL^{Me}$_{90}$-*co*-BL^{vinyl}$_{10}$) (M_n = 159 kDa, $Đ$ = 1.05), strain rate = 5 mm/min, ambient condition.

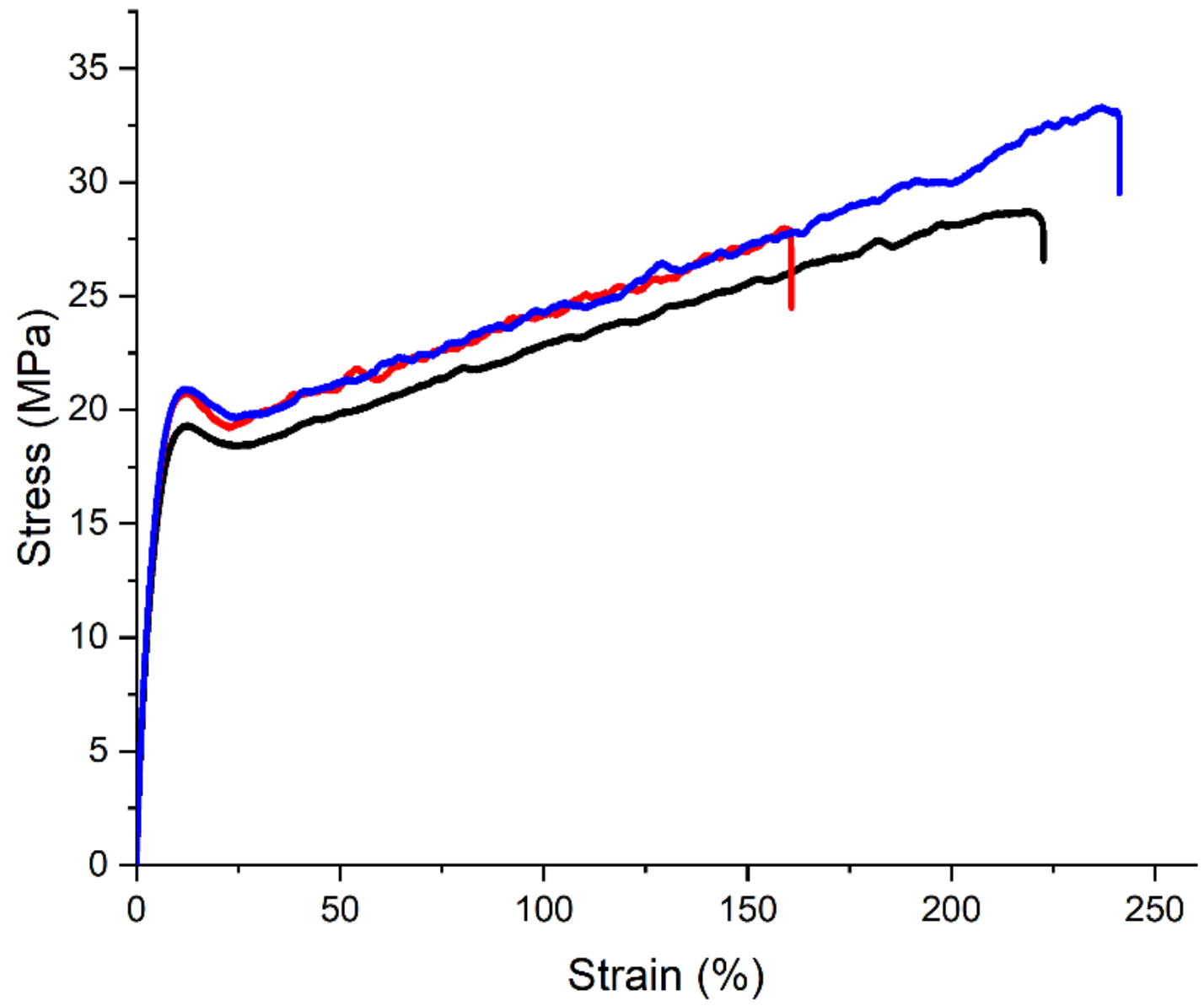

Figure S101. Tensile curves for P(BL$^{Me}{}_{96}$-*co*-BL$^{allyl}{}_{4}$) (M_n = 161 kDa, $Đ$ = 1.13), strain rate = 5 mm/min, ambient condition.

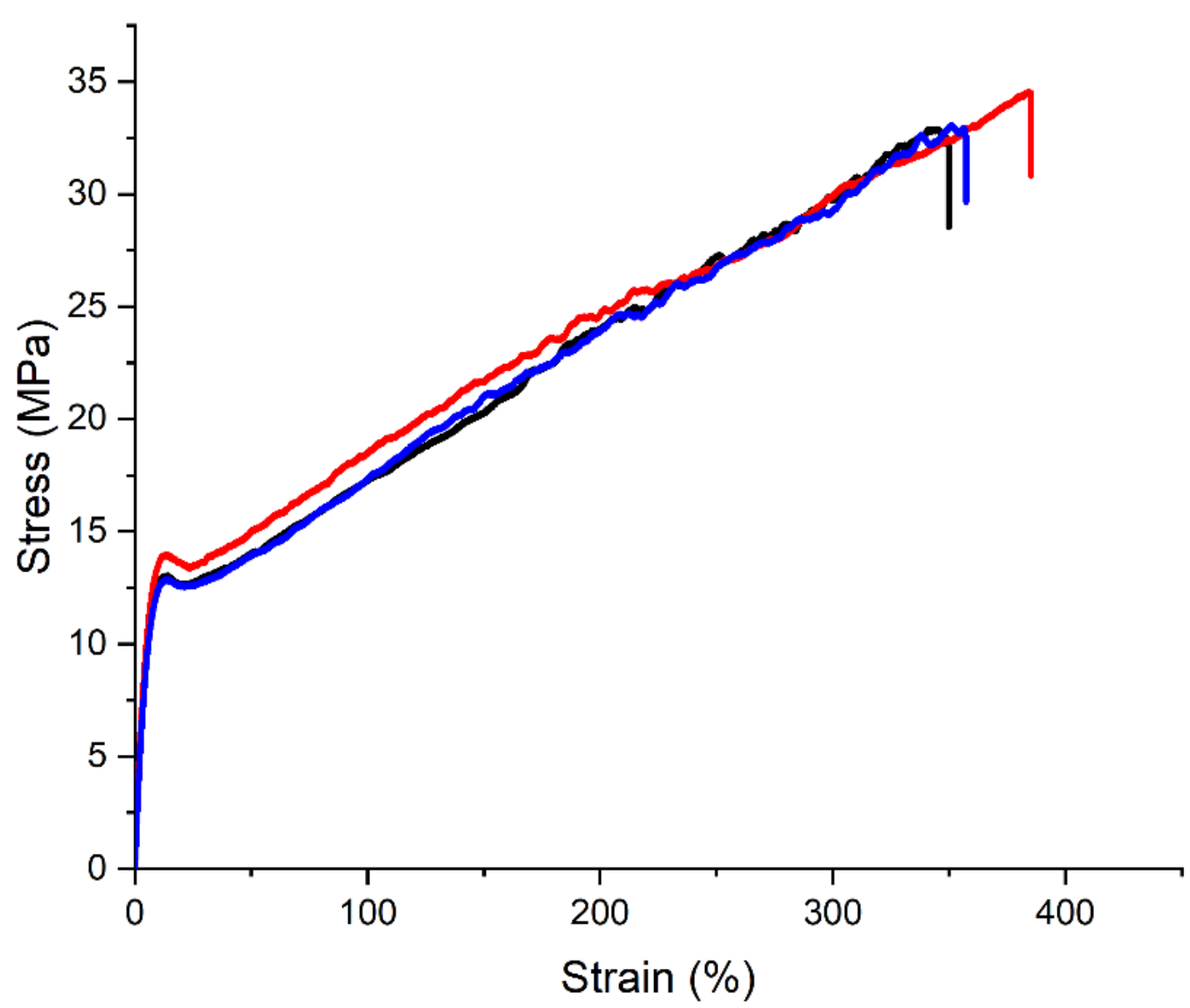

Figure S102. Tensile curves for P(BL$^{Me}{}_{92}$-*co*-BL$^{allyl}{}_{8}$) (M_n = 202 kDa, $Đ$ = 1.05), strain rate = 5 mm/min, ambient condition.

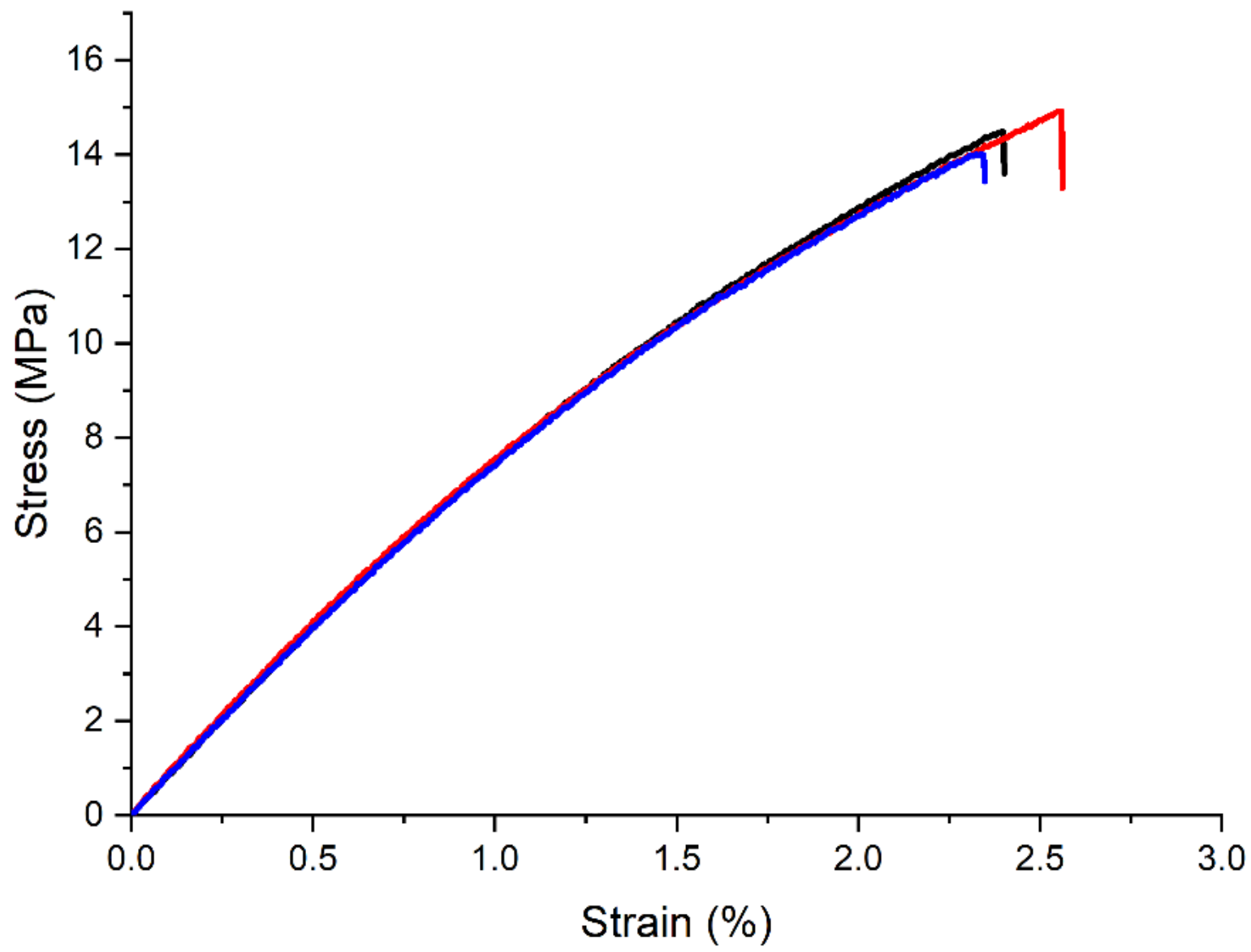

Figure S103. Tensile curves for P($BL^{Me}{}_{92}$-*co*-$BL^{allyl}{}_{8}$) prepared with **Y4** catalyst. (M_n = 121 kDa, $Đ$ = 1.01), strain rate = 5 mm/min, ambient condition.

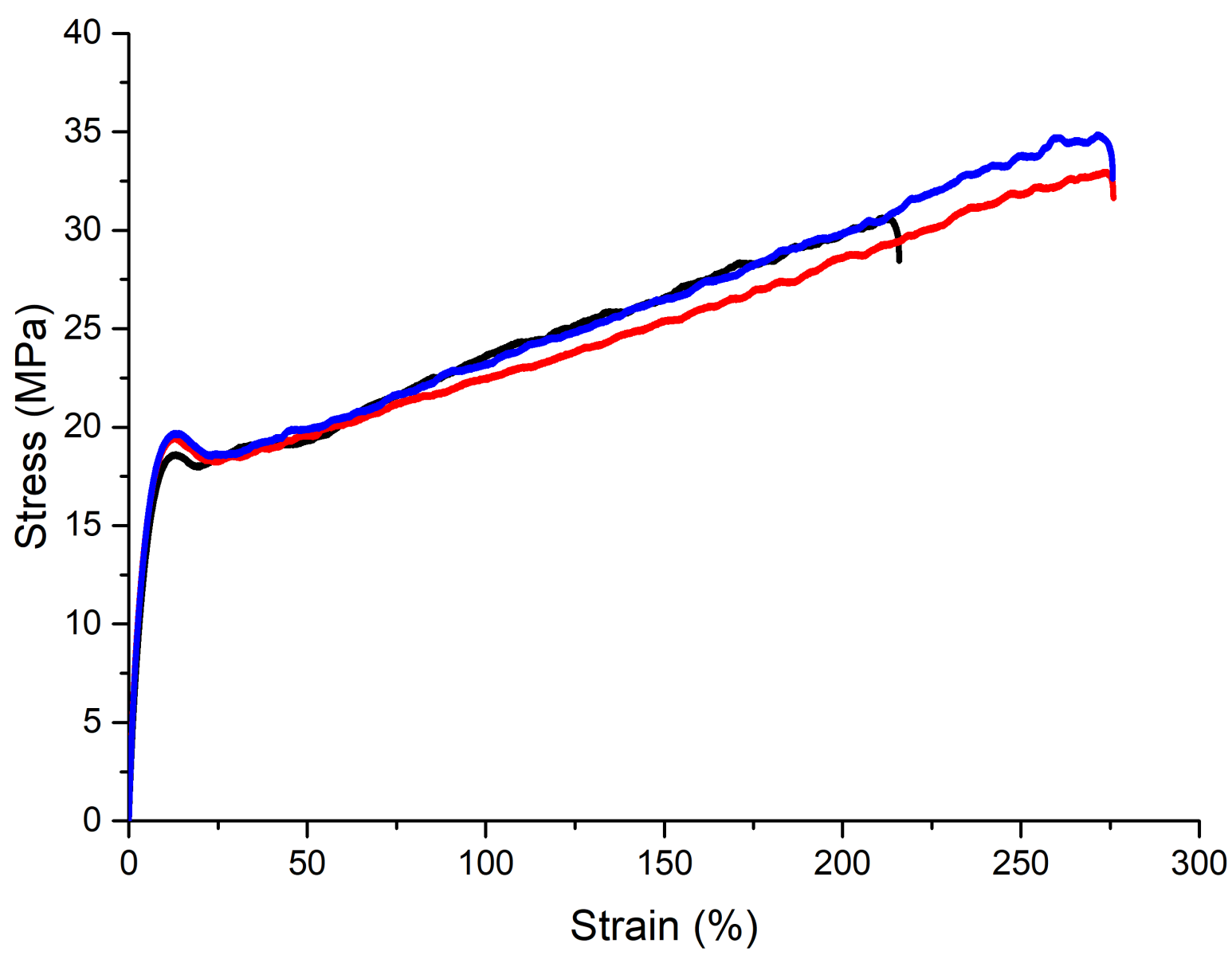

Figure S104. Tensile curves for P($BL^{Me}{}_{95}$-*co*-$BL^{hPg}{}_{5}$) (M_n = 90.5 kDa, $Đ$ = 1.08), strain rate = 5 mm/min, ambient condition.

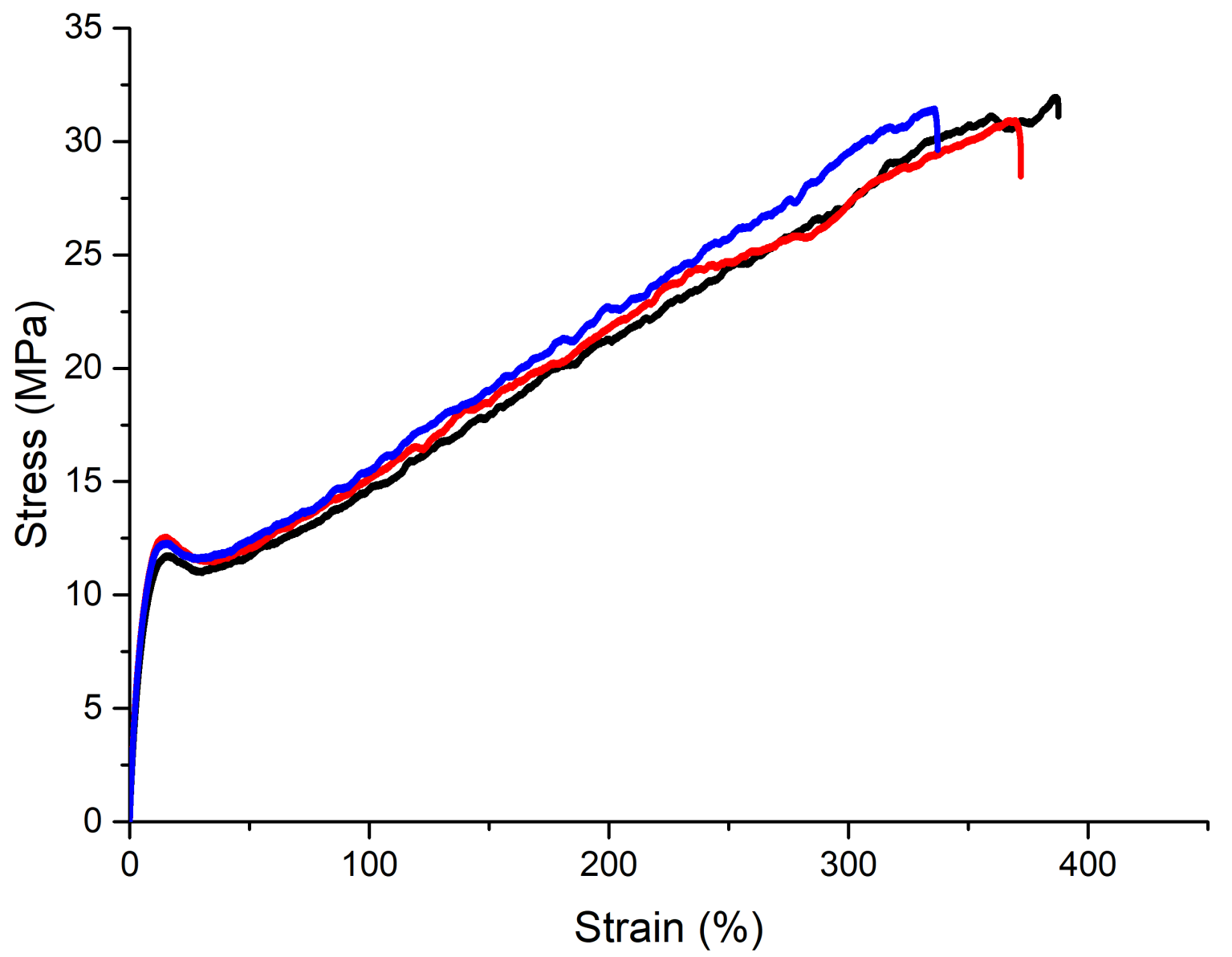

Figure S105. Tensile curves for P(BL$^{Me}{}_{90}$-*co*-BL$^{hPg}{}_{10}$) (M_n = 79.8 kDa, $Đ$ = 1.07), strain rate = 5 mm/min, ambient condition.

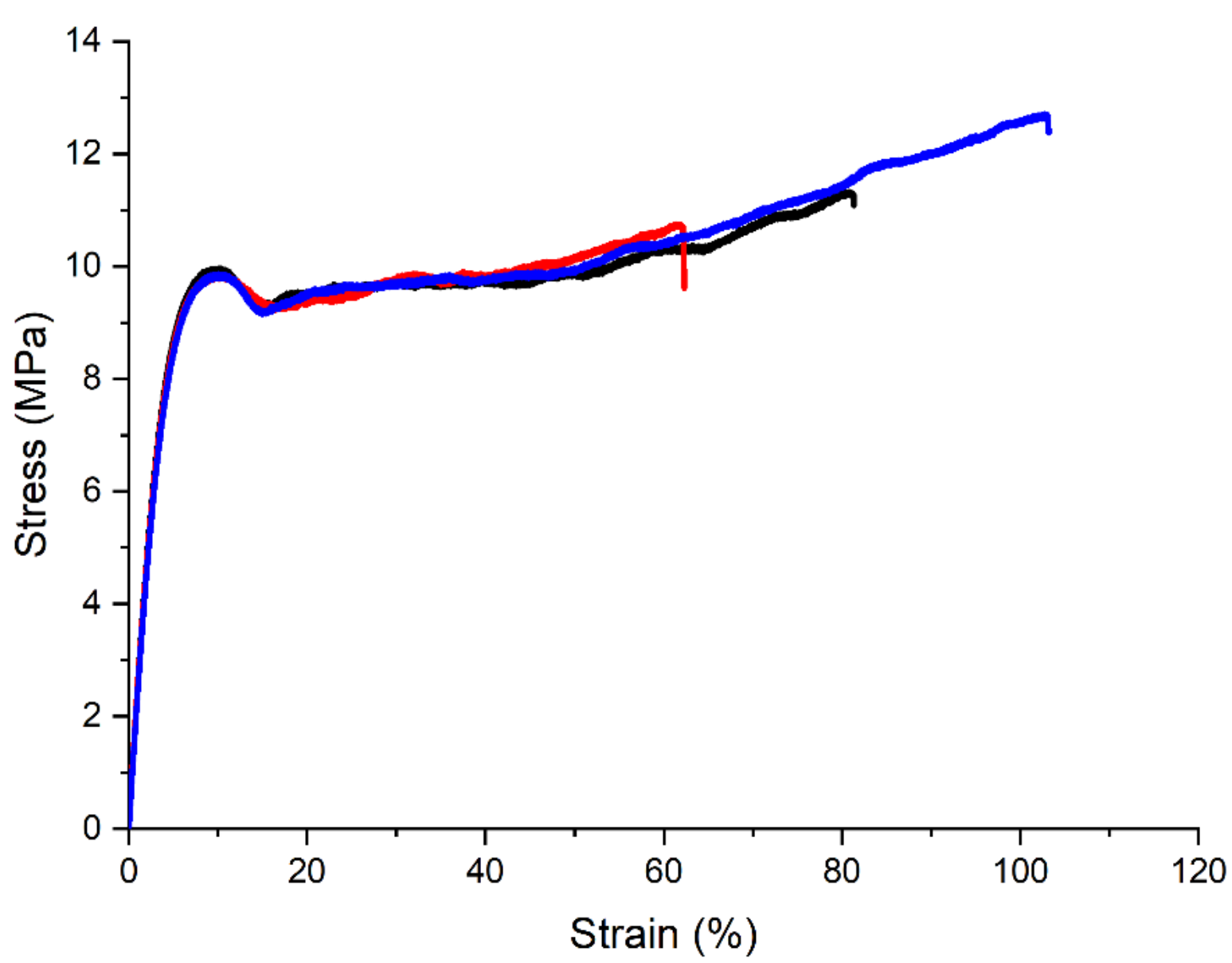

Figure S106. Tensile curves for P(BL^{vinyl})×2%, strain rate = 5 mm/min, ambient condition.

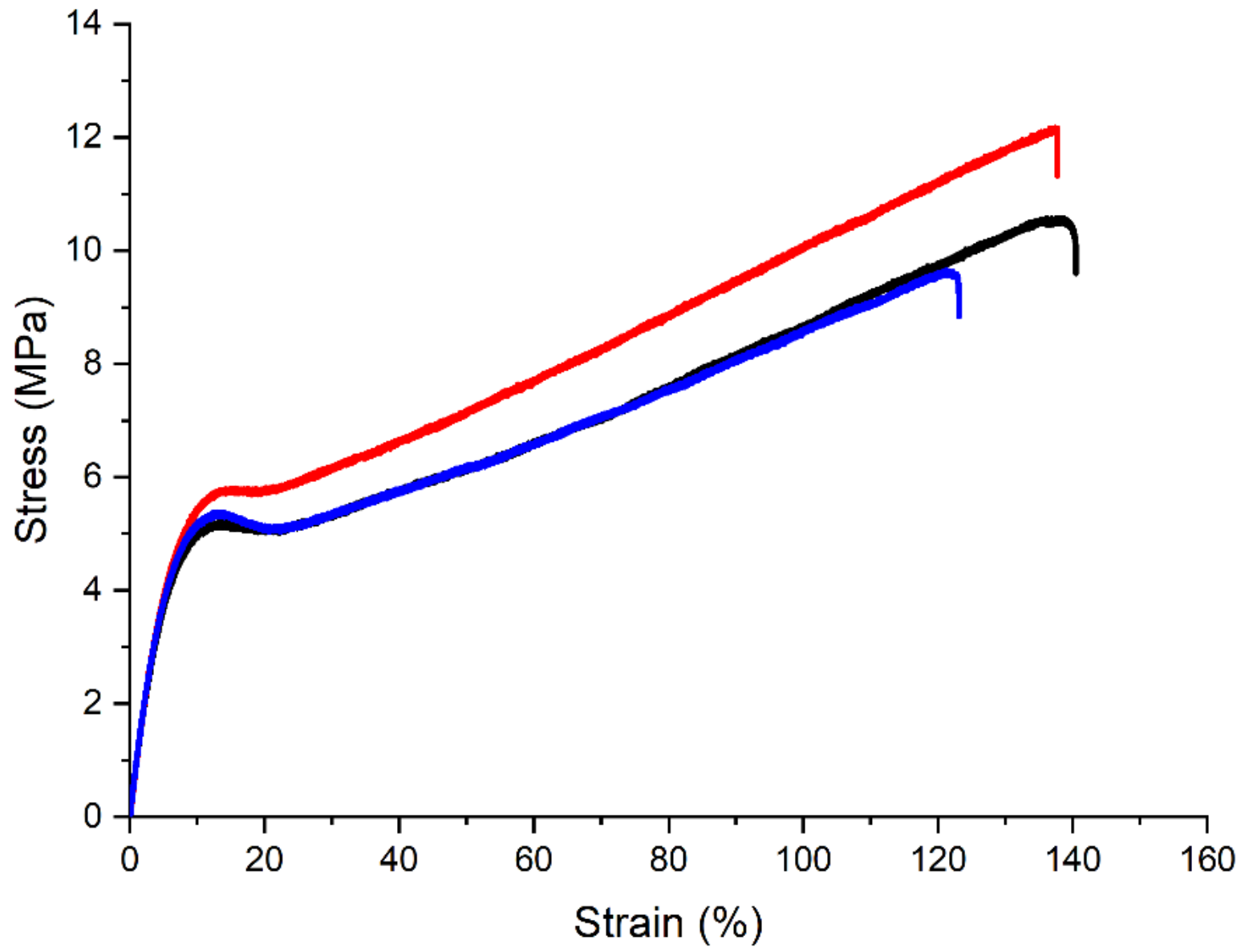

Figure S107. Tensile curves for P(BL^{vinyl})×5%, strain rate = 5 mm/min, ambient condition.

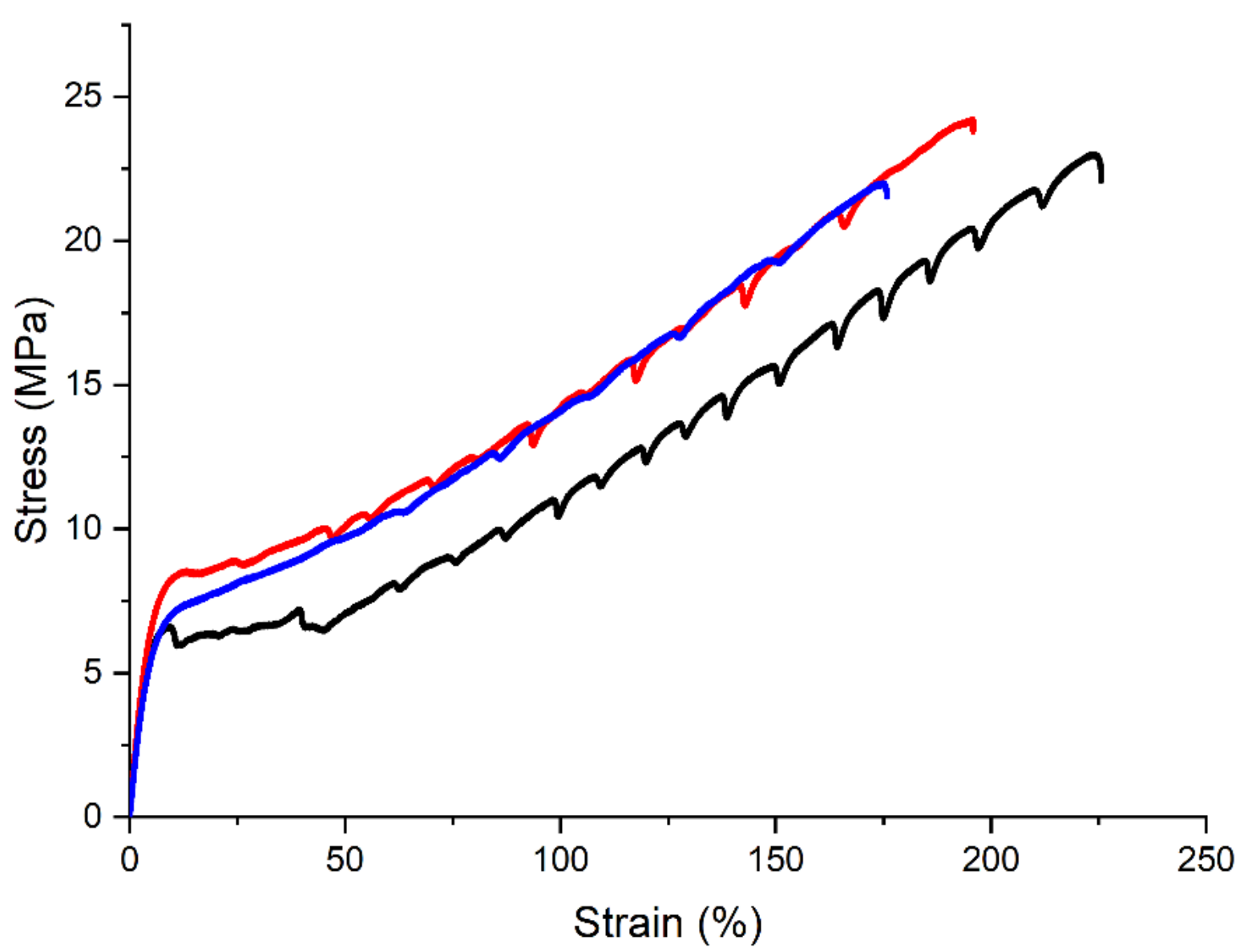

Figure S108. Tensile curves for P(BL^{allyl})×2%, strain rate = 5 mm/min, ambient condition.

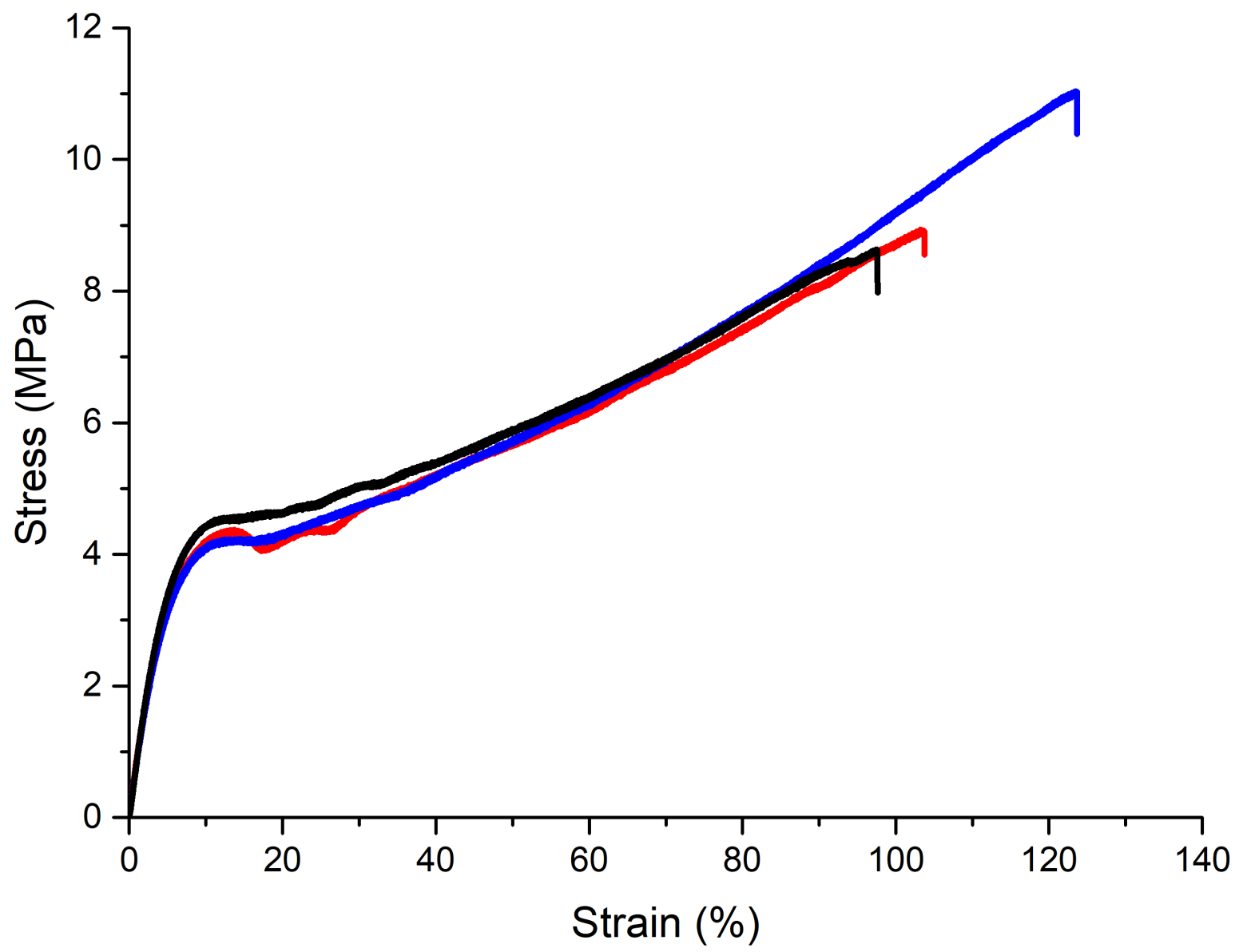

Figure S109. Tensile curves for P(BL^{allyl})×5%, strain rate = 5 mm/min, ambient condition.

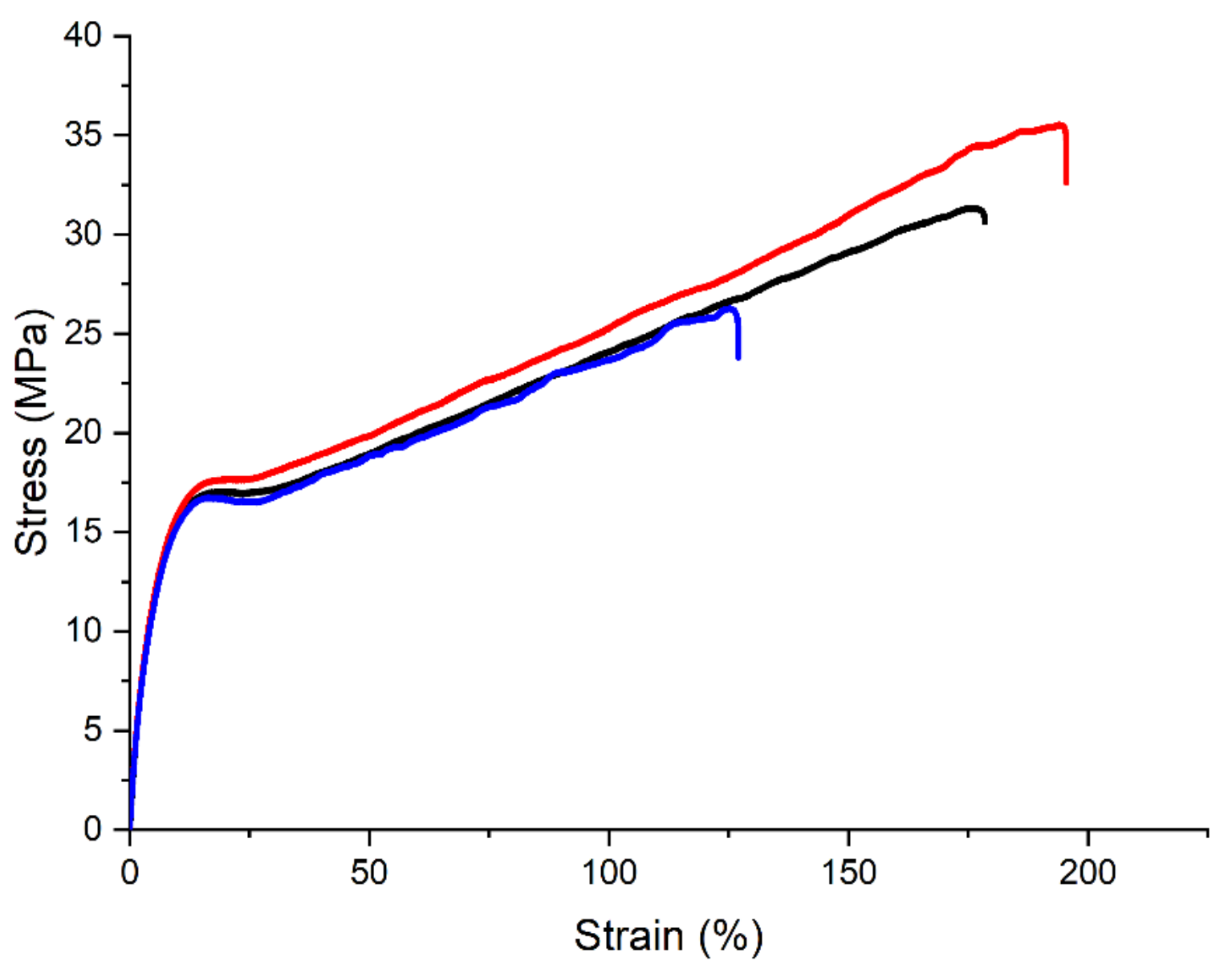

Figure S110. Tensile curves for P(BL$^{Me}{}_{96}$-*co*-BL$^{vinyl}{}_{4}$)-PETMP, strain rate = 5 mm/min, ambient condition.

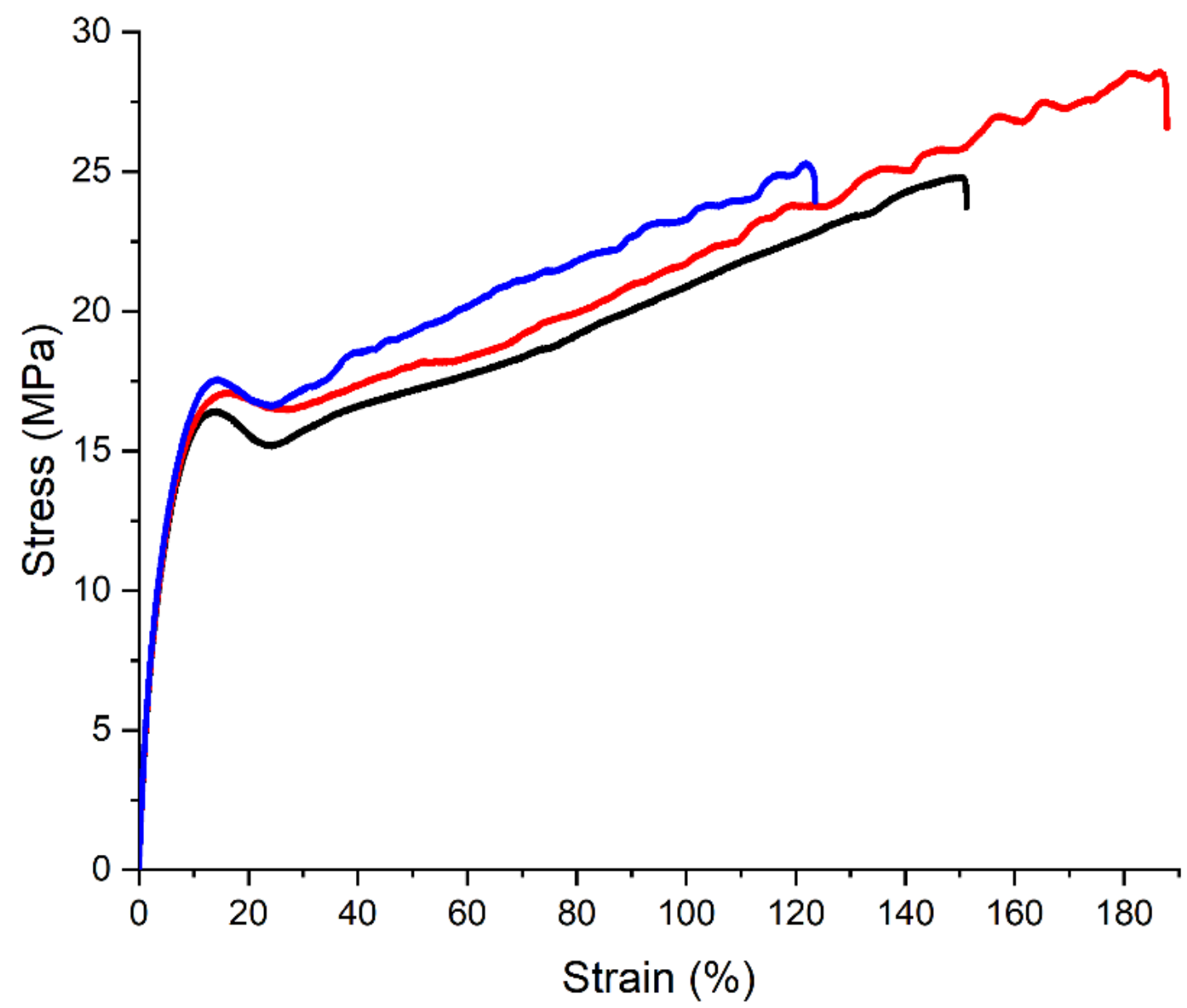

Figure S111. Tensile curves for P(BL$^{Me}_{96}$-*co*-BL$^{allyl}_{4}$)-PETMP, strain rate = 5 mm/min, ambient condition.

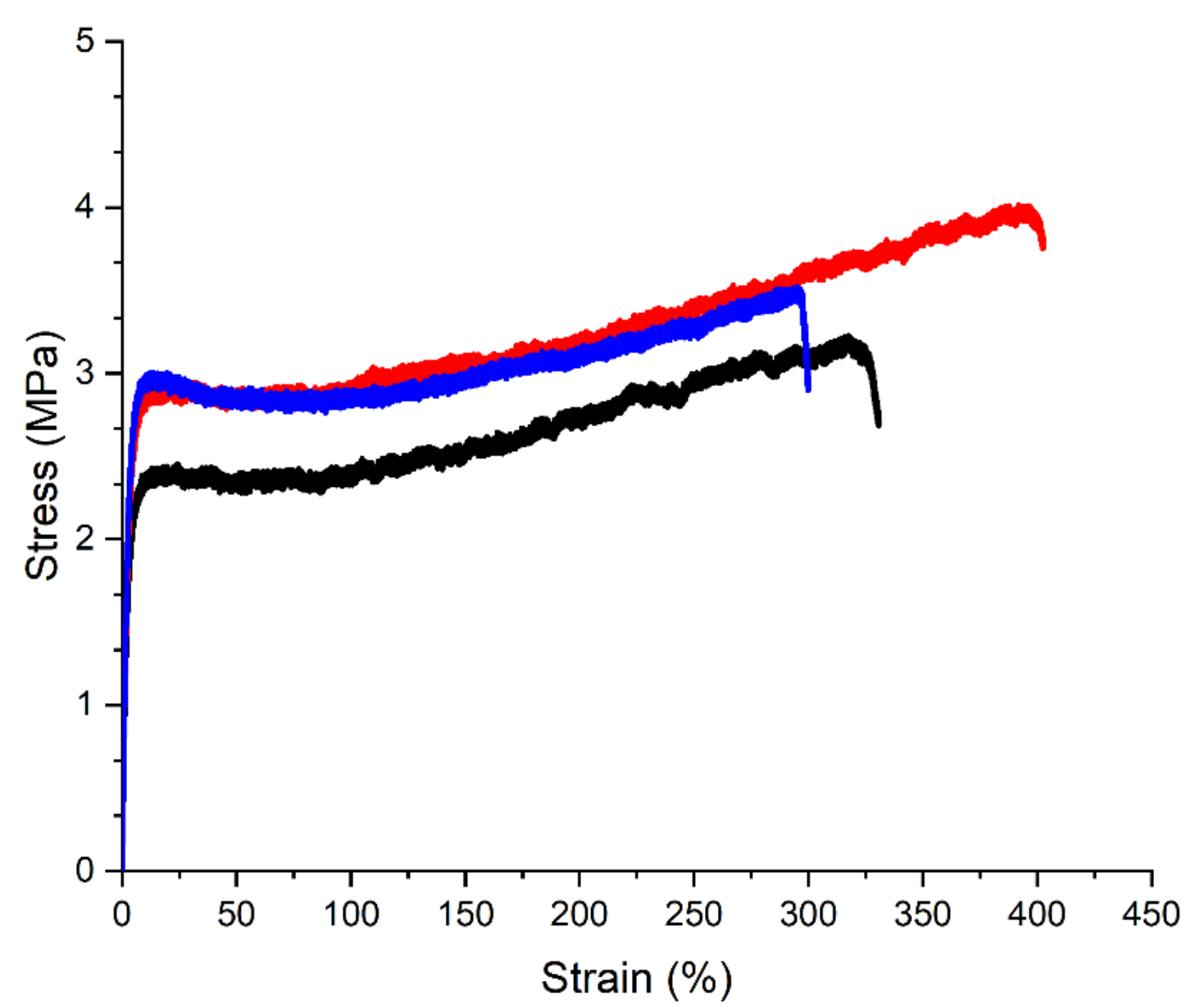

Figure S112. Tensile curves for *iso*-rich P(BL$^{Me}_{90}$-*co*-BL$^{allyl}_{10}$) (M_n = 170 kDa, $Đ$ = 1.12), strain rate = 5 mm/min, ambient condition.

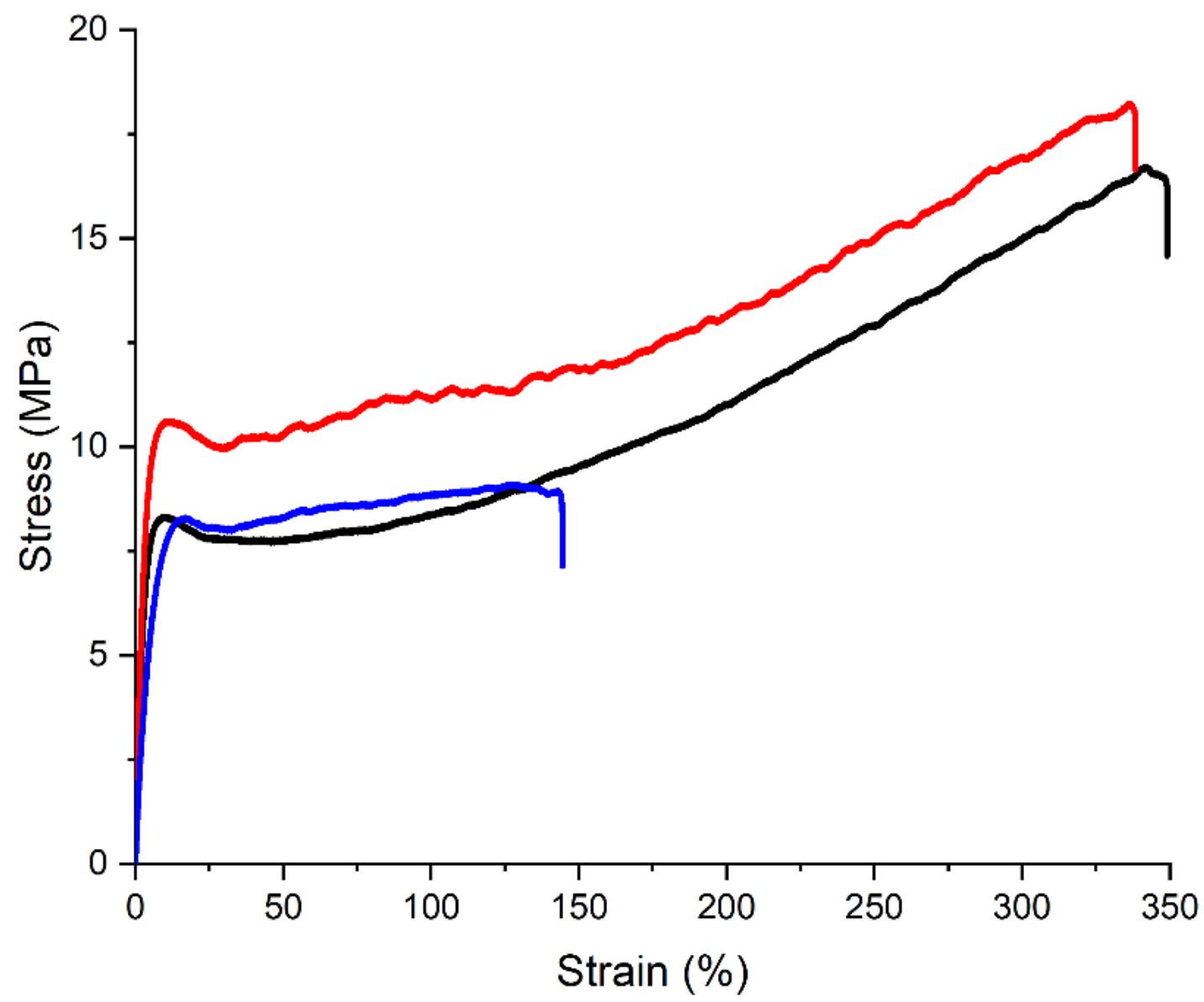

Figure S113. Tensile curves for P(BL$^{Me}{}_{90}$-*co*-BL$^{allyl}{}_{10}$)-UPy, strain rate = 5 mm/min, ambient condition.

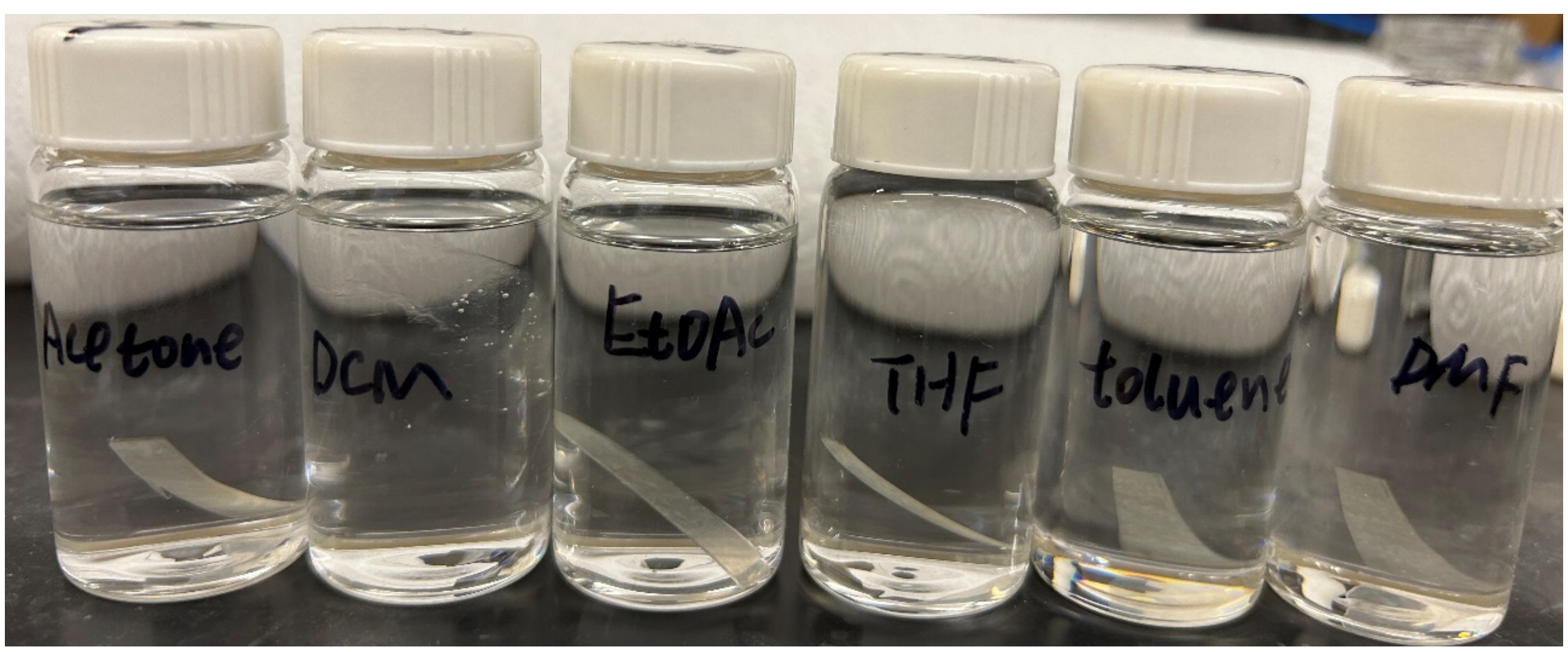

Figure S114. Solubility test of the crosslinked network in common organic solvents. Photographs of crosslinked P(BL$^{me}{}_{96}$-*co*-BL$^{allyl}{}_{4}$)-PETMP specimens immersed in acetone, dichloromethane (DCM), ethyl acetate (EtOAc), tetrahydrofuran (THF), toluene, and *N*,*N*-dimethylformamide (DMF) after one year at room temperature, demonstrating complete insolubility in all tested solvents.

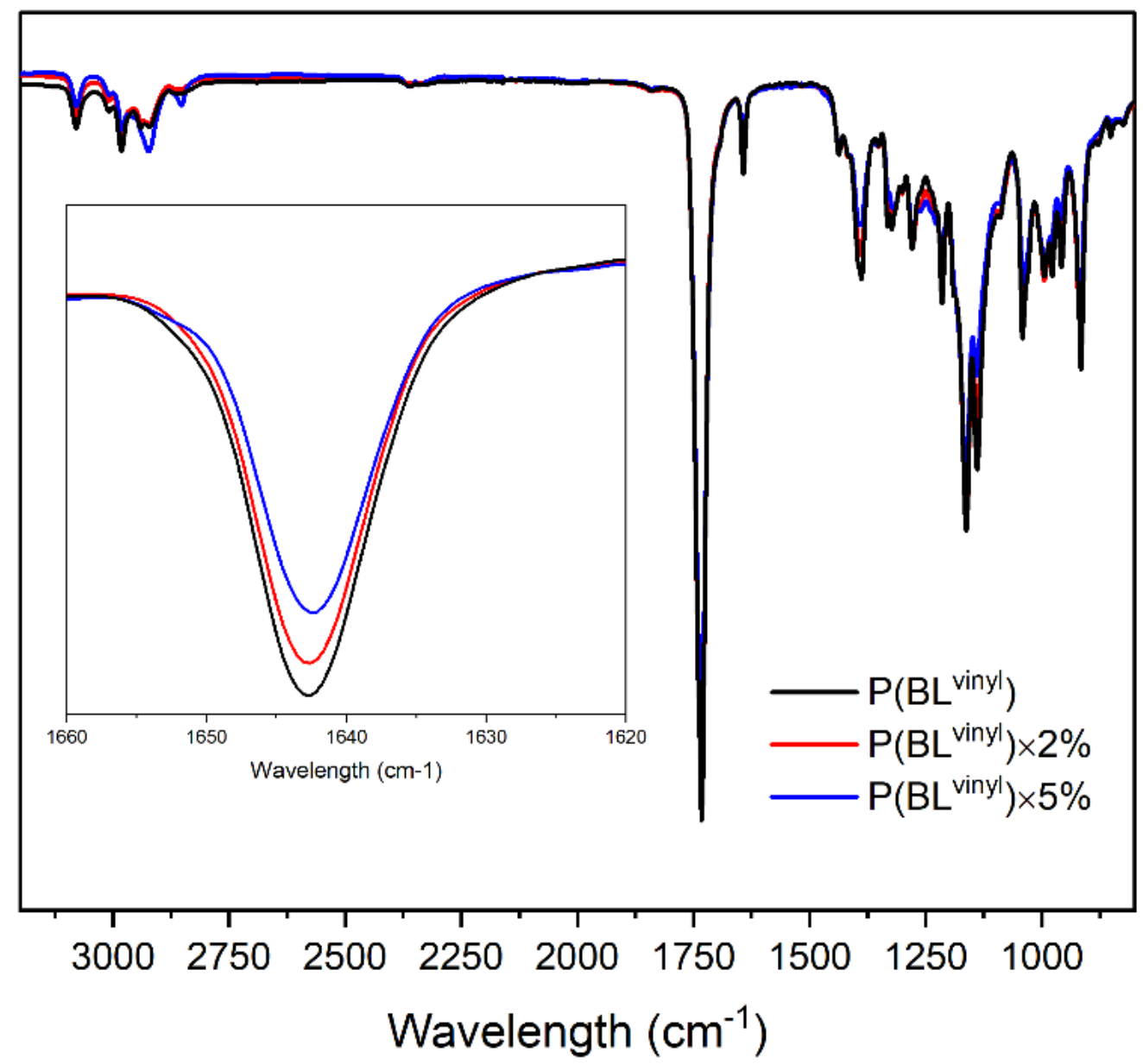

Figure S115. FT-IR spectra of crosslinked P(BL^{vinyl}). The extent of cross-linking was controlled by concentration of thiol cross-linker, as evident by the decrease of the alkene signal at 1641 cm^{-1}.

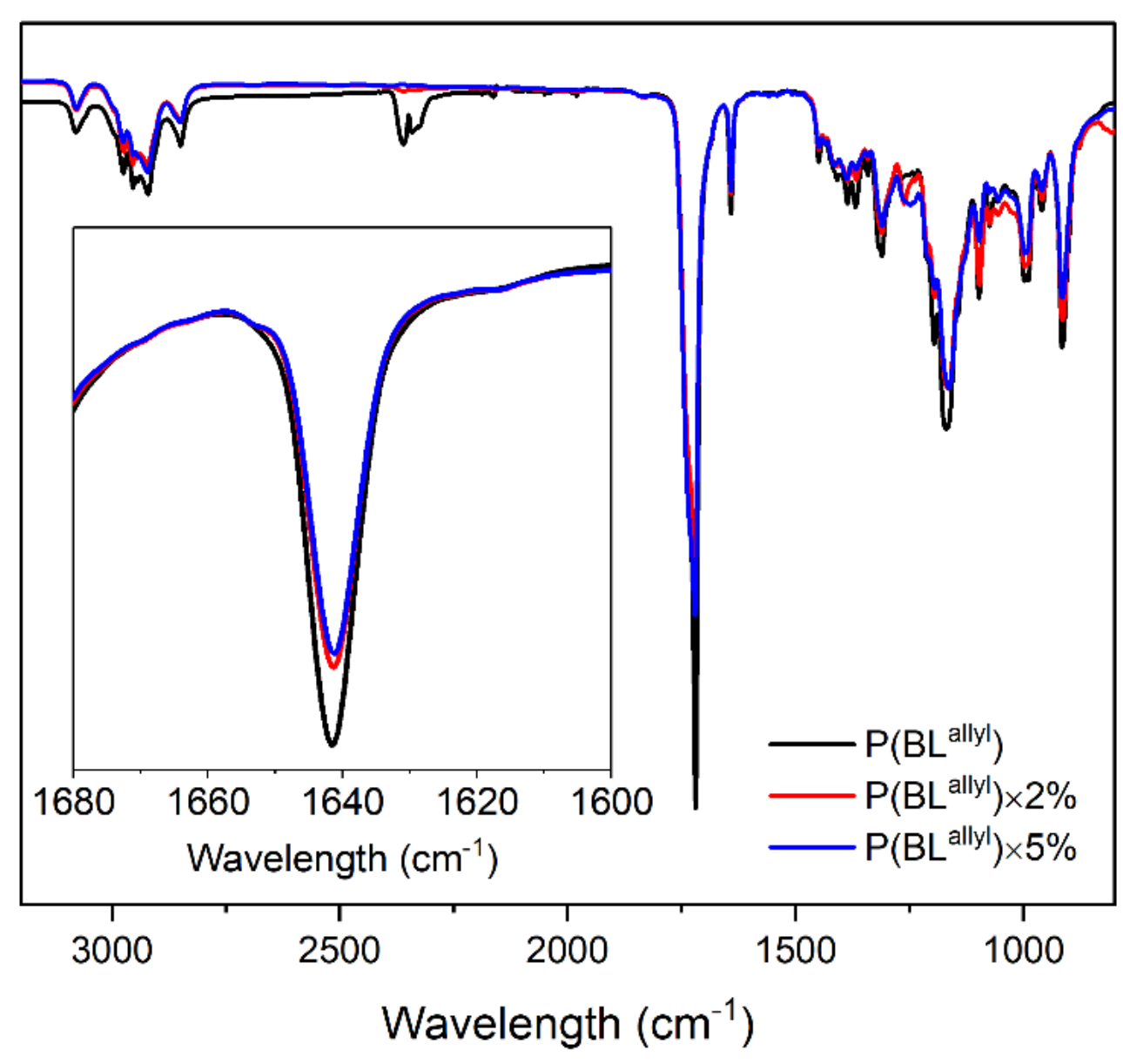

Figure S116. FT-IR spectra of crosslinked P(BL^{allyl}). The extent of cross-linking was controlled by concentration of thiol cross-linker, as evident by the decrease of the alkene signal at 1641 cm^{-1}.

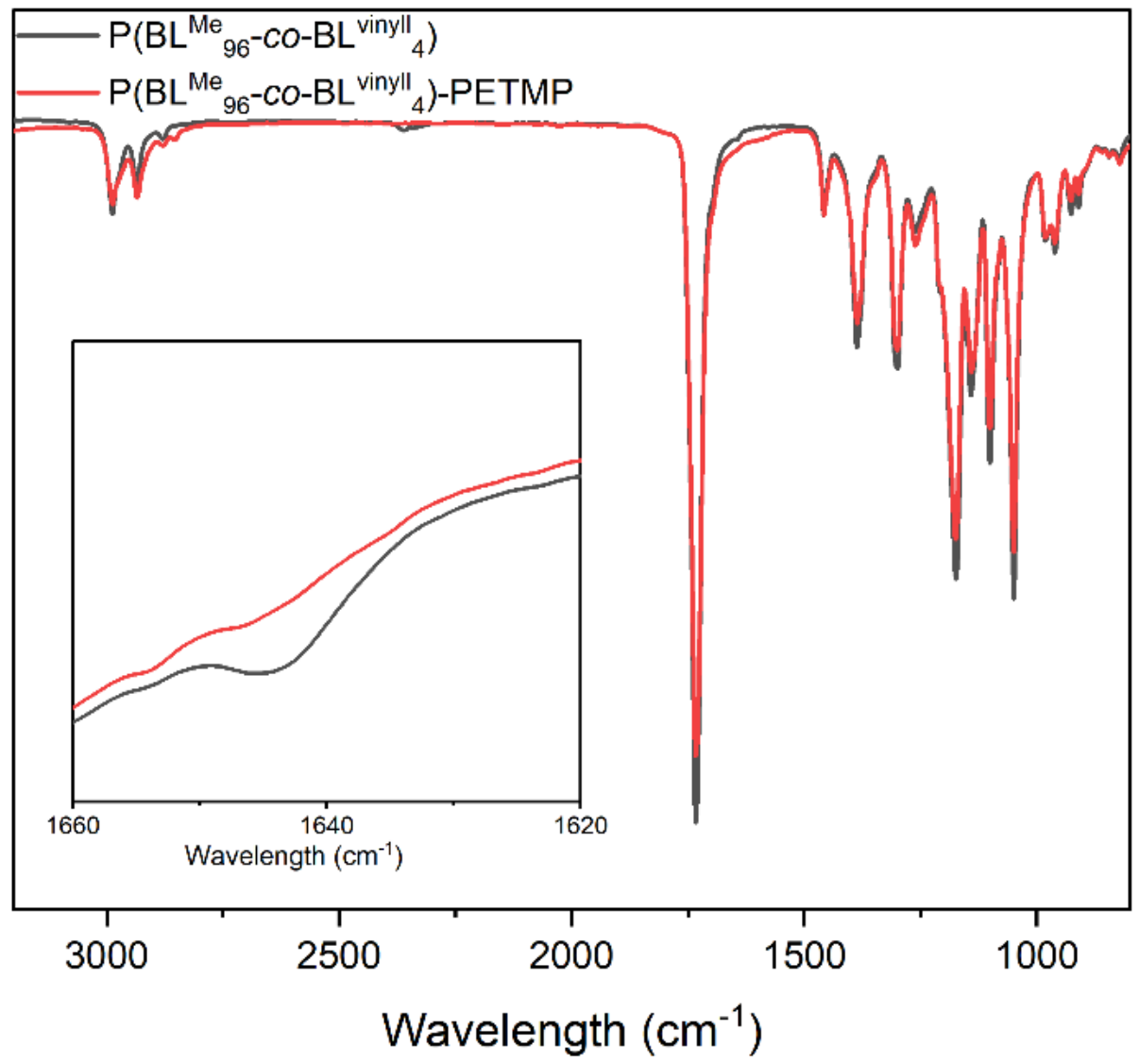

Figure S117. FT-IR spectra of P(BL^{Me}_{96}-*co*-BL^{vinyl}_4) and P(BL^{Me}_{96}-*co*-BL^{vinyl}_4)-PETMP. The extent of cross-linking was controlled by concentration of thiol cross-linker, as evident by the decrease of the alkene signal at 1641 cm^{-1}.

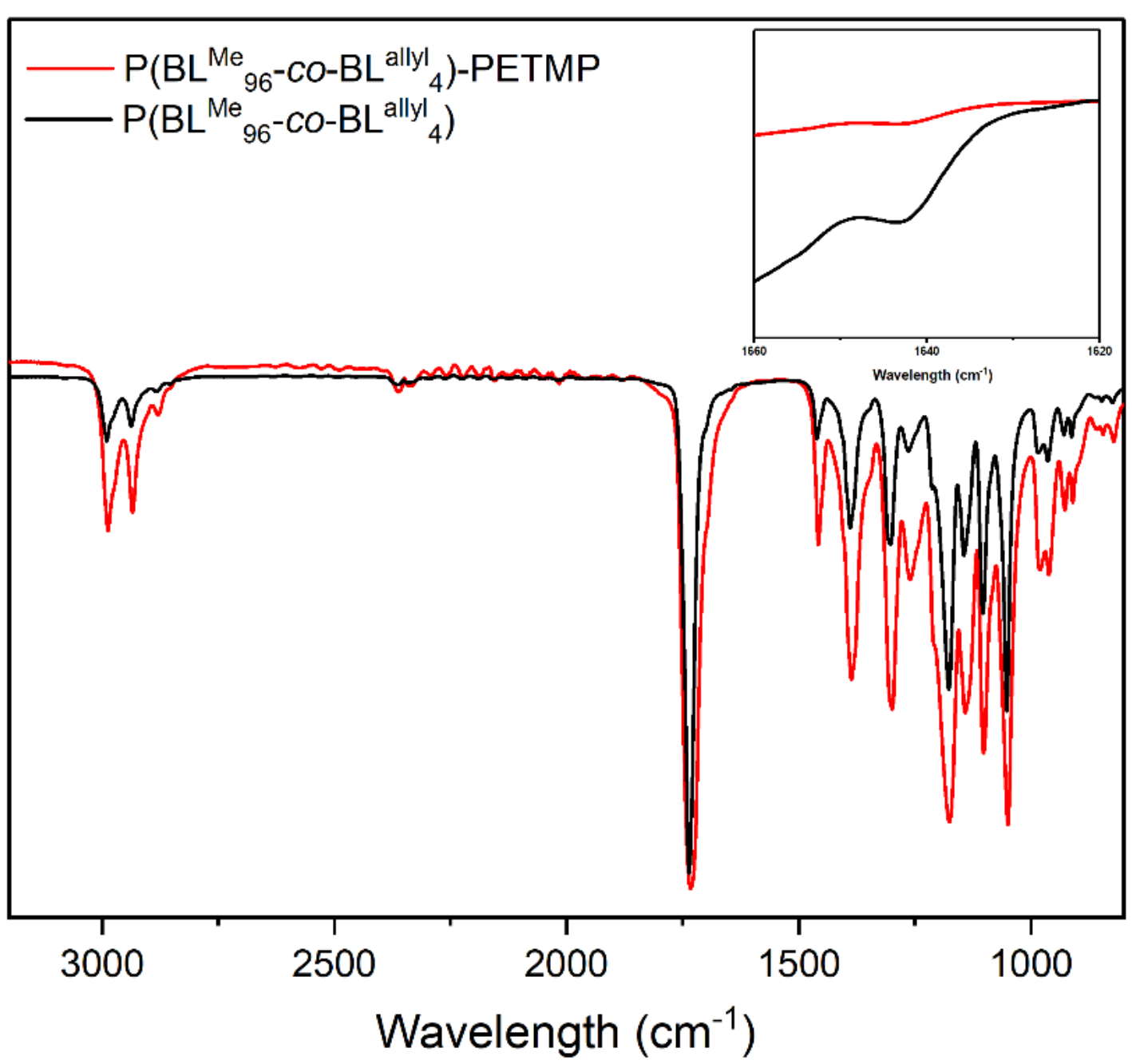

Figure S118. FT-IR spectra of P(BL^{Me}_{96}-*co*-BL^{allyl}_4) and P(BL^{Me}_{96}-*co*-BL^{allyl}_4)-PETMP. The extent of cross-linking was controlled by concentration of thiol cross-linker, as evident by the decrease of the alkene signal at 1641 cm^{-1}.

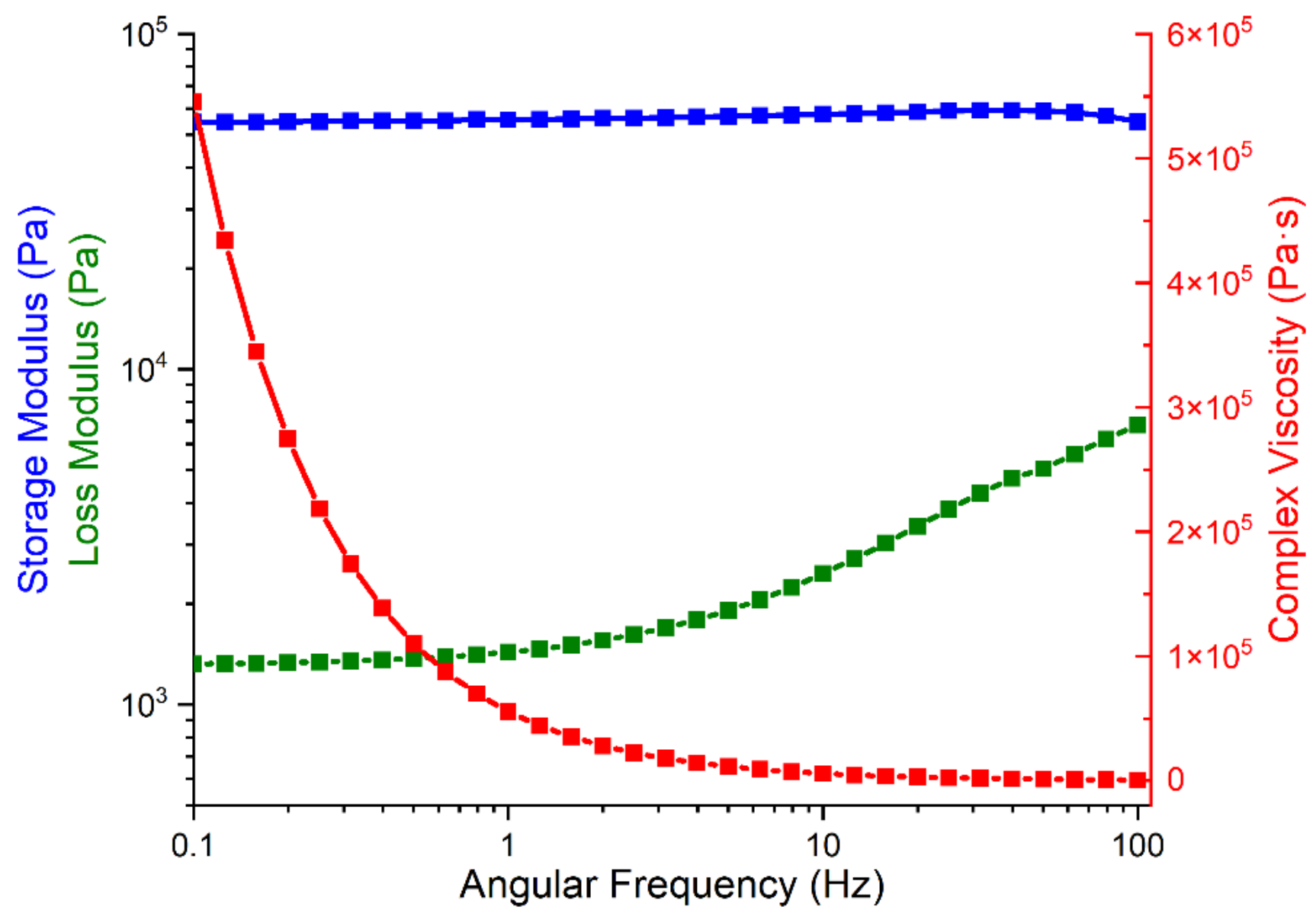

Figure S119. Frequency-dependent viscoelastic properties of the P(BL^{me}_{96}-*co*-BL^{vinyl}_{4})-PETMP. Temperature: 180 °C.

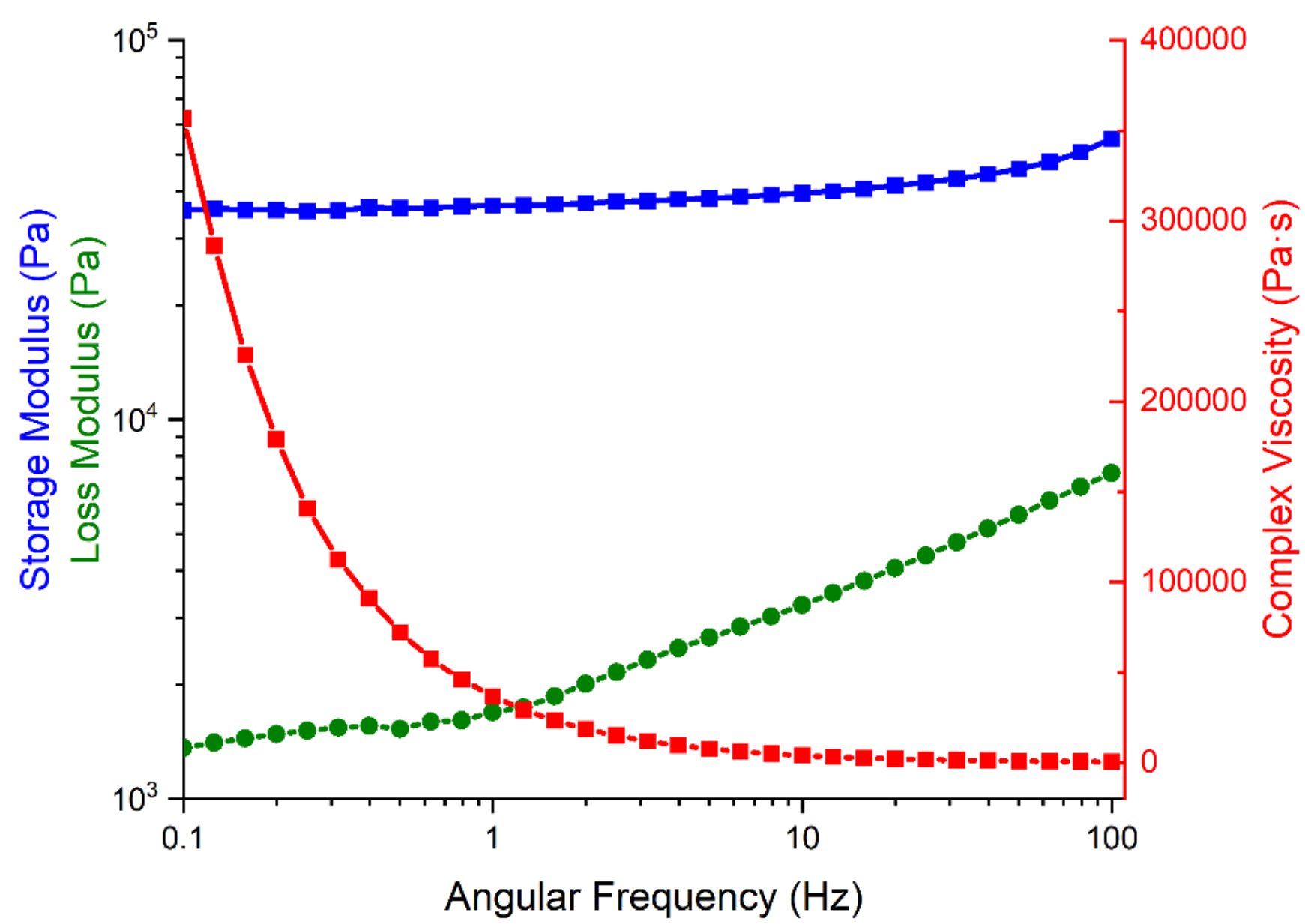

Figure S120. Frequency-dependent viscoelastic properties of the P(BL^{me}_{96}-*co*-BL^{allyl}_{4})-PETMP. Temperature: 180 °C.

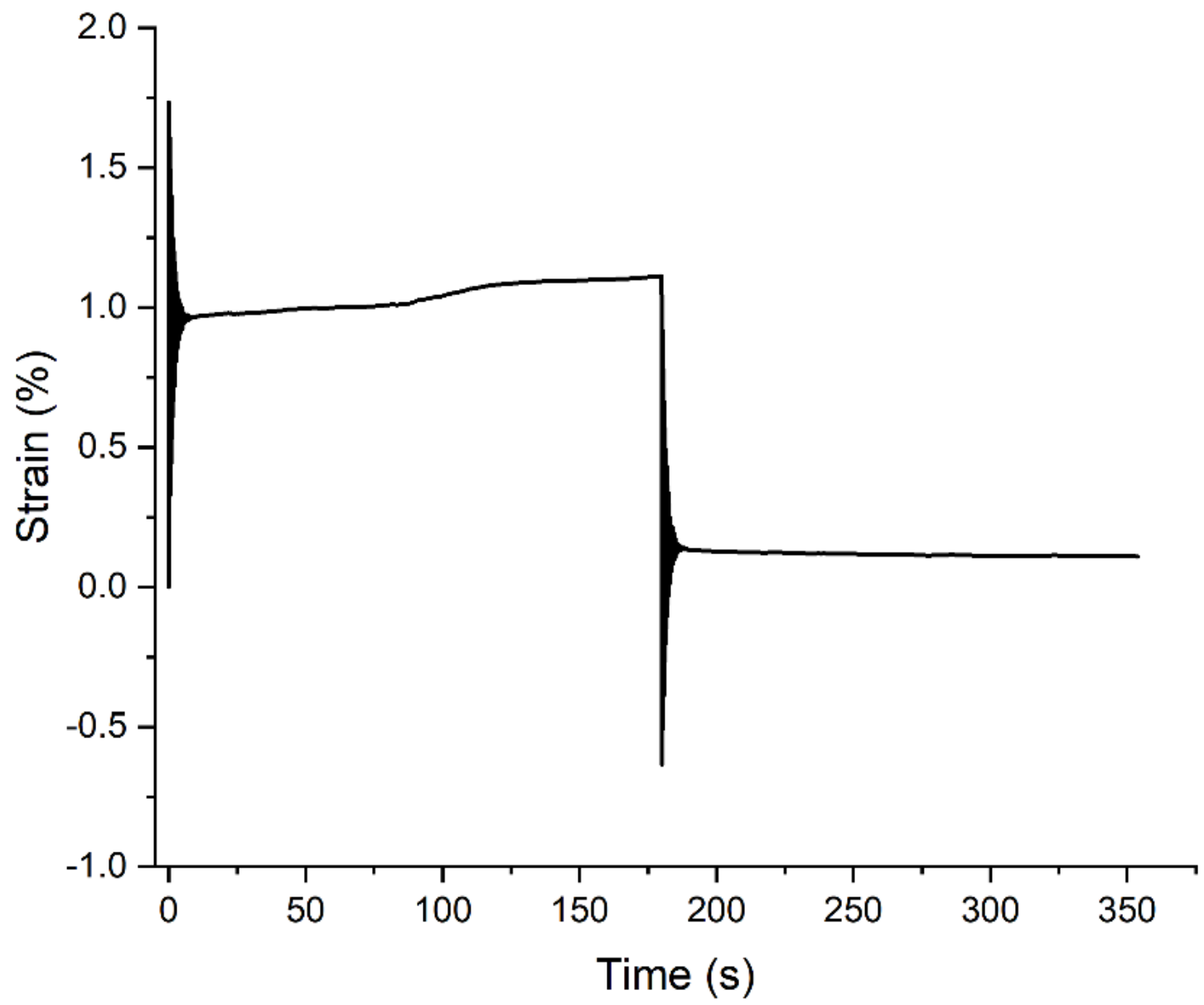

Figure S121. Creep and recovery test of P(BL$^{me}_{96}$-*co*-BL$^{vinyl}_{4}$)-PETMP at 180 °C.

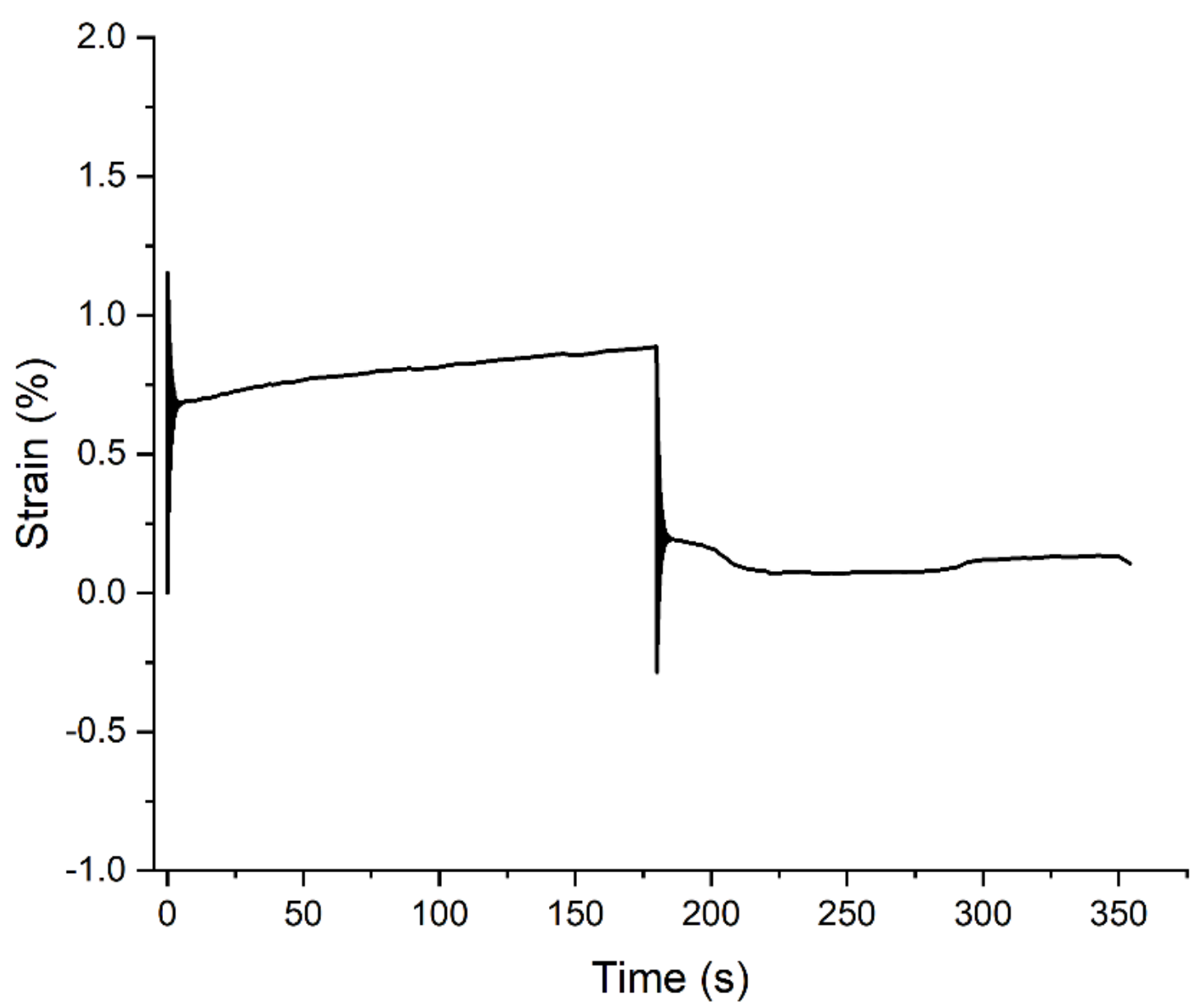

Figure S122. Creep and recovery test of P(BL$^{me}_{96}$-*co*-BL$^{allyl}_{4}$)-PETMP at 180 °C.

Supplementary Tables

Table S1. Syndioselective ROP of *rac*-BL^{vinyl}. [a]

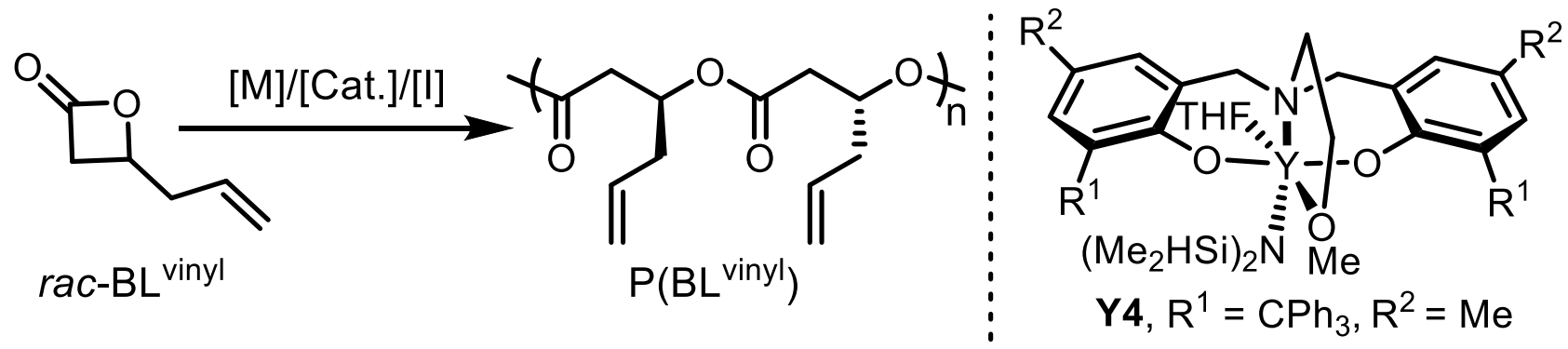

Runs	Cat.	[M]/[Cat.]/[I]	[I]	Sol.	Conv. [%]	M_n[b] [kDa]	*Đ*
**1**	**Y4**	1000:1:1	BnOH	DCM (2 M)	>99	26.9	1.02
**2**	**Y4**	1000:1:1	^{i}PrOH	DCM (2 M)	>99	34.0	1.02
**3**	**Y4**	1000:1:0.5	$C_6H_4(CHOH)_2$	PhMe (2 M)	92	23.9	1.10
**4**[c]	**Y4**	1500:1:1	^{i}PrOH	PhMe (2 M)	>99	46.9	1.09
**5**[d]	**Y4**	2000:1:1	^{i}PrOH	PhMe (5 M)	99	36.6	1.26
**6**[c]	**Y4**	2000:1:1	^{i}PrOH	PhMe (2 M)	>99	42.6	1.02
**7**	**Y4**	500:0.5:1	BnOH	DCM (2 M)	>99	19.8	1.13
**8**	**Y4**	500:0.5:1	^{i}PrOH	DCM (2 M)	>99	27.3	1.11
**9**	**Y4**	500:0.5:1	Ph_2CHOH	DCM (2 M)	>99	23.7	1.14
**10**	**Y4**	500:0.5:0.4	^{i}PrOH	PhMe (2 M)	>99	24.9	1.06
**11**	**Y4**	500:0.5:0.3	^{i}PrOH	PhMe (2 M)	>99	22.2	1.10
**12**	**Y4**	1000:1	-	DCM (2 M)	>99	40.3	1.03
**13**	**Y4**	1000:1	-	PhMe (2 M)	>99	64.2	1.03
**14**[c]	**Y4**	1500:1	-	DCM (2 M)	79	50.7	1.04
**15**[c]	**Y4**	1500:1	-	PhMe (2 M)	>99	56.7	1.08

[a] Monomer conversion (Conv.) determined by ^{1}H NMR in $CDCl_3$ at 40 °C, [*rac*-BL^{vinyl}] (560 mg, 5 mmol), polymerization time = 24 h. [b] Weight-average (M_w) and number-average (M_n) molar mass and dispersity ($Đ = M_w/M_n$) determined via size exclusion chromatography (SEC) at 40 °C in $CHCl_3$ coupled with a Wyatt DAWN HELEOS II multi (18)-angle light scattering detector and a Wyatt Optilab TrEX dRI detector for absolute molar mass. [c] [*rac*-BL^{vinyl}] (7.5 mmol, 840 mg). [d] [*rac*-BL^{vinyl}] (20 mmol, 2.24 g). [c] [*rac*-BL^{vinyl}] (7.5 mmol, 840 mg)

Table S2. ROP of *chiral* BL^{vinyl} [a]

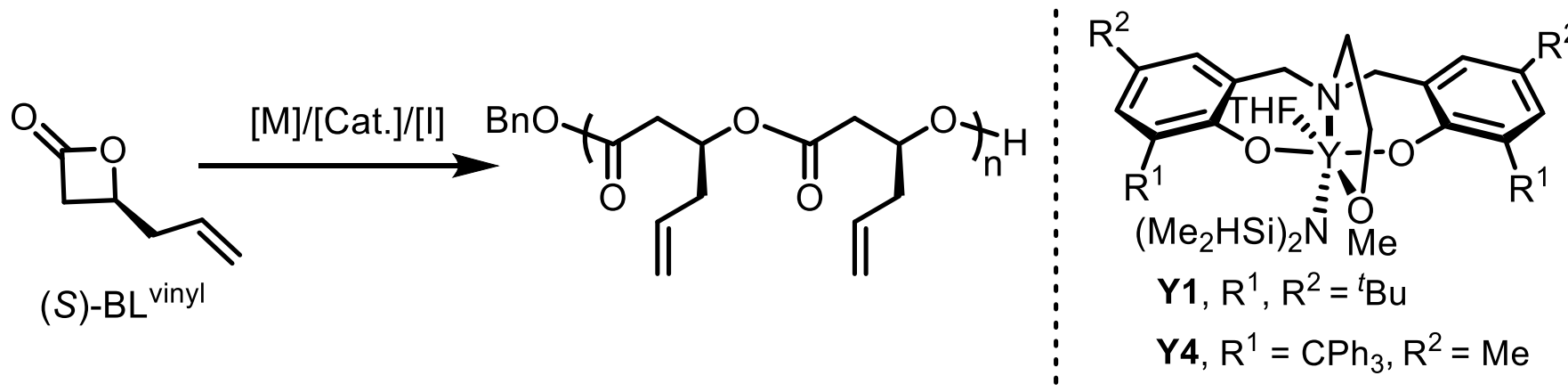

Runs	Cat.	[M]/[Cat.]/[I]	Conv. [%]	M_n[b] [kDa]	*Đ*	[*mr*] (%)	[*rr*] (%)	[*mm*] (%)	[*rm*] (%)	P_m[c]	T_g[d] (°C)
**1**	**Y1**	100:1:1	>99	5.3	1.08	6	7	88	0	0.91	-28
**2**	**Y4**	250:1:1	>99	7.3	1.12	4	5	90	1	0.93	-28
**3***d*	**Y4**	250:1:1	>99	10.3	1.09	3	6	90	0	0.92	-28

[a] Monomer conversion (Conv.) determined by ^{1}H NMR in $CDCl_3$, [(*S*)-BL^{vinyl}] (112 mg, 1.0 mmol), initiator (I) = $PhCH_2OH$, polymerization time = 12 h. [b] Weight-average (M_w) and number-average (M_n) molar mass and dispersity ($Đ = M_w/M_n$) determined via size exclusion chromatography (SEC) at 40 °C in $CHCl_3$ coupled with a Wyatt DAWN HELEOS II multi (18)-angle light scattering detector and a Wyatt Optilab TrEX dRI detector for absolute molar mass. [c] P_m is the probability of *meso* linkages between BL^{vinyl} units. [d] Observed from second heating scan of DCS curves. [e] Replace (*S*)-BL^{vinyl} with (*R*)-BL^{vinyl}.

Table S3. Syndioselective ROP of *rac*-BL^{allyl}. [a]

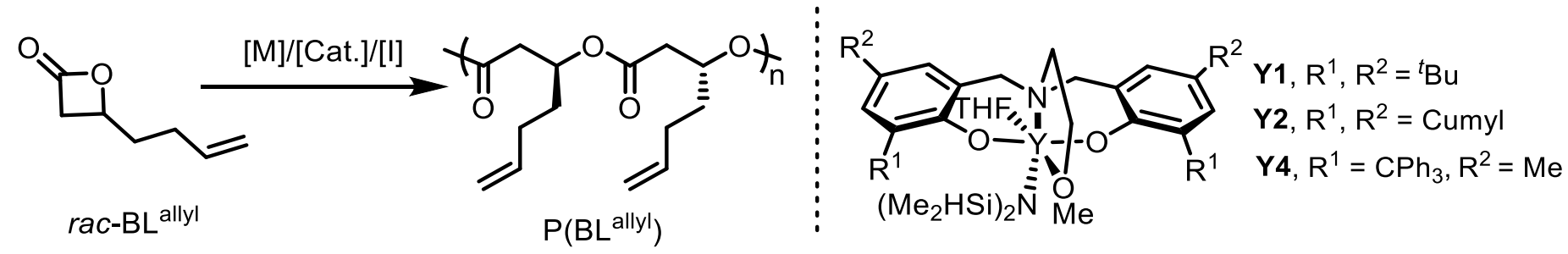

Runs	[M]/[Cat.]/[I]	Cat.	Sol.	[I]	Temp. [°C]	*t* [h]	Conv. [%]	M_n[b] [kDa]	*Đ*[b]	P_r[c]
**1**	200:1:1	**Y1**	PhMe (2 M)	^{i}PrOH	23	8	>99	14.9	1.02	0.80
**2**	200:1:1	**Y2**	PhMe (2 M)	^{i}PrOH	23	8	>99	15.5	1.01	0.85
**3**	100:1:1	**Y4**	PhMe (2 M)	BnOH	23	8	>99	15.1	1.20	0.95
**4**	100:1:1	**Y4**	PhMe (2 M)	^{i}PrOH	23	8	>99	10.0	1.05	0.95
**5**[d]	1000:1:1	**Y4**	PhMe (2 M)	^{i}PrOH	40	24	>99	65.1	1.03	0.93

**6[e]**	1500:1:1	**Y4**	PhMe (2.5M)	^{i}PrOH	40	24	95	118	1.03	0.95
**7[f]**	1500:1:1	**Y4**	PhMe (3.5M)	^{i}PrOH	40	24	95	103	1.08	0.95
**8[d]**	2000:1:1	**Y4**	PhMe (2.5M)	^{i}PrOH	40	24	98	245	1.23	0.95

[a] Monomer conversion (Conv.) determined by ^{1}H NMR in $CDCl_3$, [*rac*-BL^{allyl}] (126 mg, 1 mmol). [b] Weight-average (M_w) and number-average (M_n) molar mass and dispersity ($Đ = M_w/M_n$) determined via size exclusion chromatography (SEC) at 40 °C in $CHCl_3$ coupled with a Wyatt DAWN HELEOS II multi (18)-angle light scattering detector and a Wyatt Optilab TrEX dRI detector for absolute molar mass. [c] P_r is probability of *racemic* linkages between BL^{allyl} units. [d] [*rac*-BL^{allyl}] (630 mg, 5 mmol). [e] [*rac*-BL^{allyl}] (1.26 g, 10 mmol). [f] [*rac*-BL^{allyl}] (1.89 g, 15 mmol).

Table S4. Syndioselective ROP of *rac*-BL^{hPg}. [a]

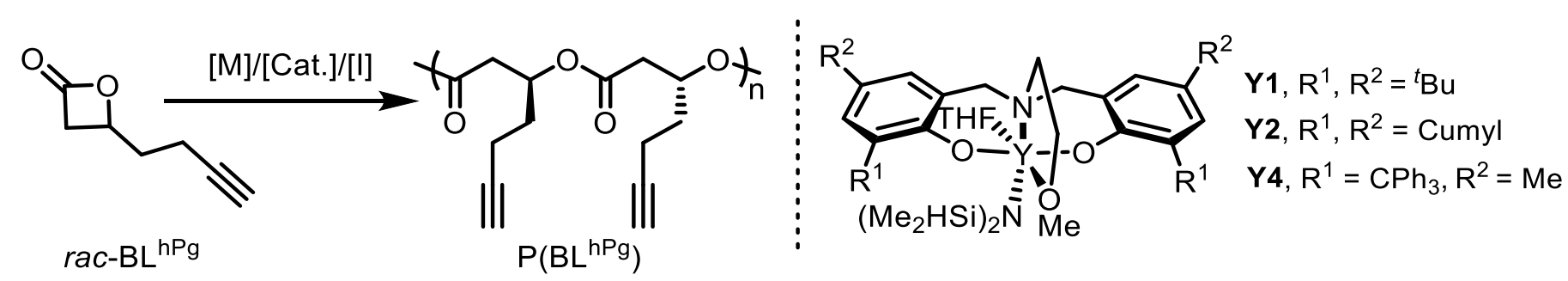

**Runs**	**[M]/[Y]/[I]**	**Cat.**	**Sol.**	**[I]**	**Temp. [°C]**	***t* [h]**	**Conv. [%]**	**M_n[b] [kDa]**	**$Đ$[b]**	**P_r[c]**
**1**	200:1:1	**Y1**	PhMe (2 M)	^{i}PrOH	23	8	>99	7.58	1.03	0.81
**2**	200:1:1	**Y2**	PhMe (2 M)	^{i}PrOH	23	8	>99	8.30	1.04	0.86
**3**	100:1:1	**Y4**	PhMe (2 M)	BnOH	23	8	>99	11.8	1.07	0.90
**4[d]**	1000:1:1	**Y4**	neat	BnOH	23	48	69	55.1	1.13	0.91
**5[d]**	1000:1:1	**Y4**	neat	^{i}PrOH	40	24	63	36.7	1.24	0.93
**6[d]**	1000:1:1	**Y4**	PhMe (2 M)	^{i}PrOH	40	24	98	70.5	1.23	0.91
**7[e]**	2000:1:1	**Y4**	PhMe (3 M)	^{i}PrOH	40	24	83	72.9	1.03	0.90

[a] Monomer conversion (Conv.) determined by ^{1}H NMR in $CDCl_3$, [*rac*-BL^{hPg}] (124 mg, 1 mmol). [b] Weight-average (M_w) and number-average (M_n) molar mass and dispersity ($Đ = M_w/M_n$) determined via size exclusion chromatography (SEC) at 40 °C in $CHCl_3$ coupled with a Wyatt DAWN HELEOS II multi (18)-angle light scattering detector and a Wyatt Optilab TrEX dRI detector for absolute molar mass. [c] P_r is probability of *racemic* linkages between BL^{hPg} units. [d] [*rac*-BL^{hPg}] (620 mg, 5 mmol). [e] [*rac*-BL^{hPg}] (1.24 g, 10 mmol).

Table S5. Synthesis of Atactic Functionalized Homopolymer.[a]

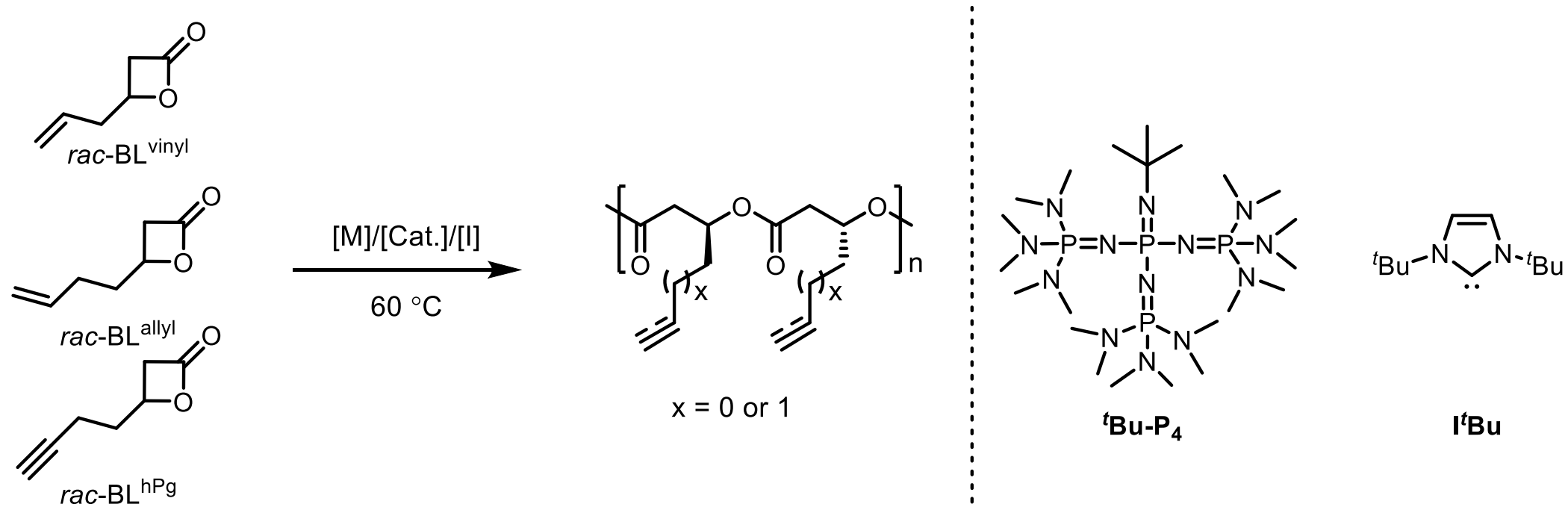

Runs	Cat.	[M]	[M]/[Cat.]/[I]	Sol.	Conv. [%]	M_n[b] [kDa]	$Đ$[b]
**1**	^{t}Bu-P$_4$	*rac*-BL^{vinyl}	100:1:1	PhMe (5 M)	80	3.57	1.12
**2**	^{t}Bu-P$_4$	*rac*-BL^{allyl}	100:1:1	PhMe (5 M)	97	7.03	1.17
**3**	I^{t}Bu	*rac*-BL^{hPg}	100:1:1	THF (5 M)	>99	9.50	1.06

[a] Monomer conversion determined by ^{1}H NMR in $CDCl_3$, [M] (1.0 mmol), initiator (I) = $PhCH_2OH$, 12 h; [b] Weight-average (M_w) and number-average (M_n) molar mass and dispersity ($Đ = M_w/M_n$) determined via size exclusion chromatography (SEC) at 40 °C in $CHCl_3$ coupled with a Wyatt DAWN HELEOS II multi (18)-angle light scattering detector and a Wyatt Optilab TrEX dRI detector for absolute molar mass.

Table S6. Copolymerization of *rac*-β-Butyrolactone and *rac*-Vinyl-β-Butyrolactone.[a]

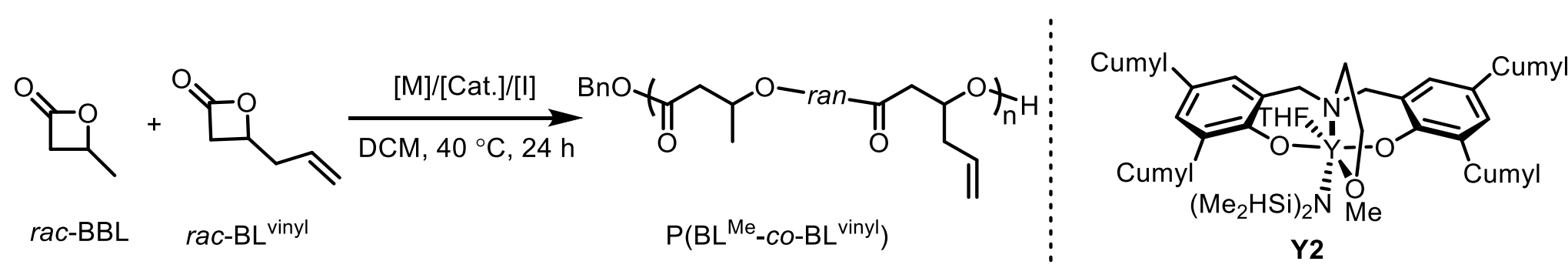

Runs	[*rac*-BBL]/[Cat.]/[I]	*rac*-BBL:*rac*-BL^{vinyl}	[*rac*-BBL] Conv.[%]	[*rac*-BL^{vinyl}] Conv.[%]	Sol.	Vinyl Content[b] [mol%]	M_n[c] [kDa]	$Đ$[c]
**1**[d]	1000:1:1	95:5	100	100	DCM (2 M)	4	66.0	1.10
**2**[e]	1000:1:1	95:5	79	78	DCM (6 M)	6	56.7	1.10
**3**[f]	2000:1:1	95:5	97	78	DCM (3.5M)	5	169	1.08
**4**[g]	1000:1:1	90:10	74	70	DCM (6 M)	9	46.7	1.11
**5**[h]	2000:1:1	90:10	93	90	DCM (3.5M)	10	159	1.05

[a] Monomer conversion (Conv.) determined by ^{1}H NMR in $CDCl_3$, initiator (I) = initiator (I)=$PhCH_2OH$, 24 h, 40 °C. [b] The content of vinyl group was determined by ^{1}H NMR analysis of the isolated copolymer. [c] Weight-average (M_w) and number-average (M_n) molar mass and dispersity ($Đ = M_w/M_n$) determined via size exclusion chromatography (SEC) at 40 °C in $CHCl_3$ coupled with a Wyatt DAWN HELEOS II multi (18)-angle light scattering detector and a Wyatt Optilab TrEX dRI detector for absolute molar mass. [d] [*rac*-BBL] (860 mg, 10

mmol), [*rac*-BL^{vinyl}] (58.2 mg, 0.52 mmol). [e] [*rac*-BBL] (2.0 g, 23.25 mmol), [*rac*-BL^{vinyl}] (137.74 mg,1.23 mmol). [f] [*rac*-BBL] (1.72 g, 20 mmol), [*rac*-BL^{vinyl}] (117.9 mg, 1.06 mmol). [g] [*rac*-BBL] (2.0 g, 23.25 mmol), [*rac*-BL^{vinyl}] (275.48 mg, 2.46 mmol). [h] [*rac*-BBL] (1.72 g, 20 mmol), [*rac*-BL^{vinyl}] (248.9 mg, 2.22 mmol).

Table S7. Copolymerization of *rac*-β-Butyrolactone and *rac*-Allyl-β-Butyrolactone. [a]

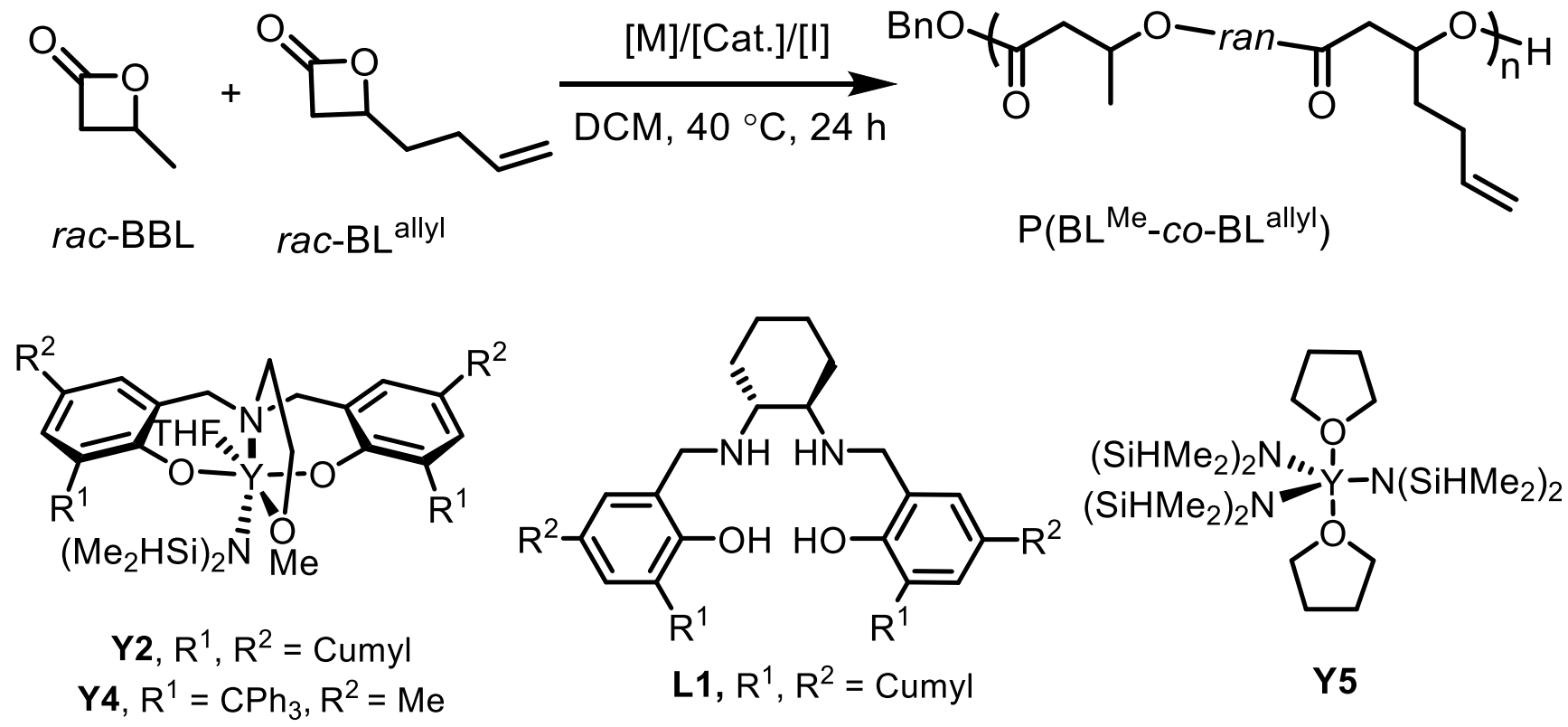

Runs	[*rac*-BBL]/[Cat.]/[I]	Cat.	*rac*-BBL:*rac*-BL^{allyl}	[*rac*-BBL] Conv.[%]	[*rac*-BL^{allyl}] Conv.[%]	Sol.	Allyl Content[b] [mol%]	M_n[c] [kDa]	$Đ$[c]
**1**[d]	1000:1:1	**Y2**	95:5	86	45	DCM (4 M)	4	70.5	1.08
**2**[e]	2000:1:1	**Y2**	95:5	85	55	DCM (3.5M)	4	161	1.13
**3**[f]	1000:1:1	**Y2**	90:10	65	47	DCM (6 M)	8	65.0	1.13
**4**[g]	2000:1:1	**Y2**	90:10	87	66	DCM (3.5M)	8	202	1.05
**5**[h]	2000:1:1	**Y4**	90:10	96	75	DCM (3.5M)	8	121	1.01
**6**[i]	500:1	**L1+Y5**	90:10	>99	>99	Tol (2 M)	10	170	1.12

[a] Monomer conversion (Conv.) determined by ^{1}H NMR in $CDCl_3$, initiator (I) = $PhCH_2OH$, 24 h, 40 °C. [b] The content of allyl group was determined by ^{1}H NMR analysis of the isolated copolymer. [c] Weight-average (M_w) and number-average (M_n) molar mass and dispersity ($Đ$ = M_w/M_n) determined via size exclusion chromatography (SEC) at 40 °C in $CHCl_3$ coupled with a Wyatt DAWN HELEOS II multi (18)-angle light scattering detector and a Wyatt Optilab TrEX dRI detector for absolute molar mass. [d] [*rac*-BBL] (1.15 g, 13.39 mmol), [*rac*-BL^{allyl}] (88 mg, 0.69 mmol). [e] [*rac*-BBL] (1.72 g, 20 mmol), [*rac*-BL^{allyl}] (133.6 mg, 1.06 mmol). [f] [*rac*-BBL] (2.0 g, 23.25mmol), [*rac*-BL^{allyl}] (325.5 mg, 2.58 mmol). [g] [*rac*-BBL] (1.72 g, 20 mmol), [*rac*-BL^{allyl}] (280.0 mg, 2.22 mmol). [h] [*rac*-BBL] (860 g, 10 mmol), [*rac*-BL^{allyl}] (140 mg, 1.11 mmol). [i] [*rac*-BBL] (1.72 g, 20 mmol), [*rac*-BL^{allyl}] (280.0 mg, 2.22 mmol).

Table S8. Copolymerization of *rac*-β-Butyrolactone and *rac*-hPg-β-Butyrolactone.[a]

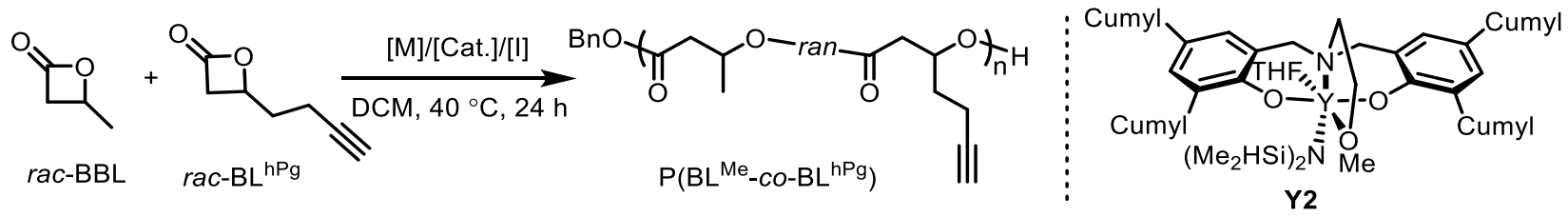

Runs	[*rac*-BBL]/[Cat.]/[I]	*rac*-BBL:*rac*-BL^{hPg}	[*rac*-BBL] Conv.[%]	[*rac*-BL^{hPg}] Conv.[%]	Sol.	hPg Content[b] [mol%]	M_n[c] [kDa]	$Đ$[c]
1[d]	2000:1:1	90:5	93	90	DCM (3.5 M)	5	90.5	1.08
2[e]	2000:1:1	90:10	91	87	DCM (3.5 M)	10	79.8	1.07

[a] Monomer conversion (Conv.) determined by ^{1}H NMR in $CDCl_3$, initiator (I) = $PhCH_2OH$, 24 h, 40 °C. [b] The content of hPg group was determined by ^{1}H NMR analysis of the isolated copolymer. [c] Weight-average (M_w) and number-average (M_n) molar mass and dispersity ($Đ$ = M_w/M_n) determined via size exclusion chromatography (SEC) at 40 °C in $CHCl_3$ coupled with a Wyatt DAWN HELEOS II multi (18)-angle light scattering detector and a Wyatt Optilab TrEX dRI detector for absolute molar mass. [d] [*rac*-BBL] (860 mg, 10 mmol), [*rac*-BL^{hPg}] (65.3 mg, 0.526 mmol). [e] [*rac*-BBL] (860 mg, 10 mmol), [*rac*-BL^{hPg}] (137.6 mg, 1.11 mmol).

Table S9. Synthesis of Atactic Functionalized Copolymer.[a]

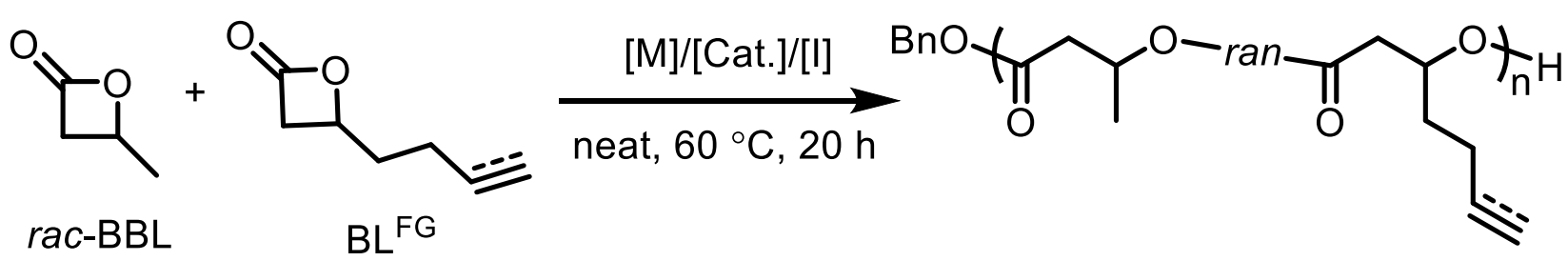

Runs	[*rac*-BBL]/[Cat.]/[I]	[Cat.]	*rac*-BBL: BL^{FG}	[*rac*-BBL] Conv.[%]	[*rac*-BL^{FG}] Conv.[%]	BL^{FG} Content[b] [mol%]	M_n[c] [kDa]	$Đ$[c]
1[d]	400:1:2	$Zn(HMDS)_2$	90:5	>99	>99	5	17.0	1.15
2[e]	400:1:2	$Zn(HMDS)_2$	90:5	94	>99	5	11.5	1.26

[a] Monomer conversion (Conv.) determined by ^{1}H NMR in $CDCl_3$, FG = Functional Group (allyl or hPg), initiator (I) = $PhCH_2OH$, 20 h, 60 °C. [b] The content of functional group was determined by ^{1}H NMR analysis of the isolated copolymer. [c] Weight-average (M_w) and number-average (M_n) molar mass and dispersity ($Đ$ = M_w/M_n) determined via size exclusion chromatography (SEC) at 40 °C in $CHCl_3$ coupled with a Wyatt DAWN HELEOS II multi (18)-angle light scattering detector and a Wyatt Optilab TrEX dRI detector for absolute molar mass. [d] [*rac*-BBL] (860 mg, 10 mmol), [*rac*-BL^{allyl}] (65.3 mg, 0.526 mmol). [e] [*rac*-BBL] (860 mg, 10 mmol), [*rac*-BL^{hPg}] (65.2 mg, 0.526 mmol).

Table S10. ROP of *rac*-β-Butyrolactone.[a]

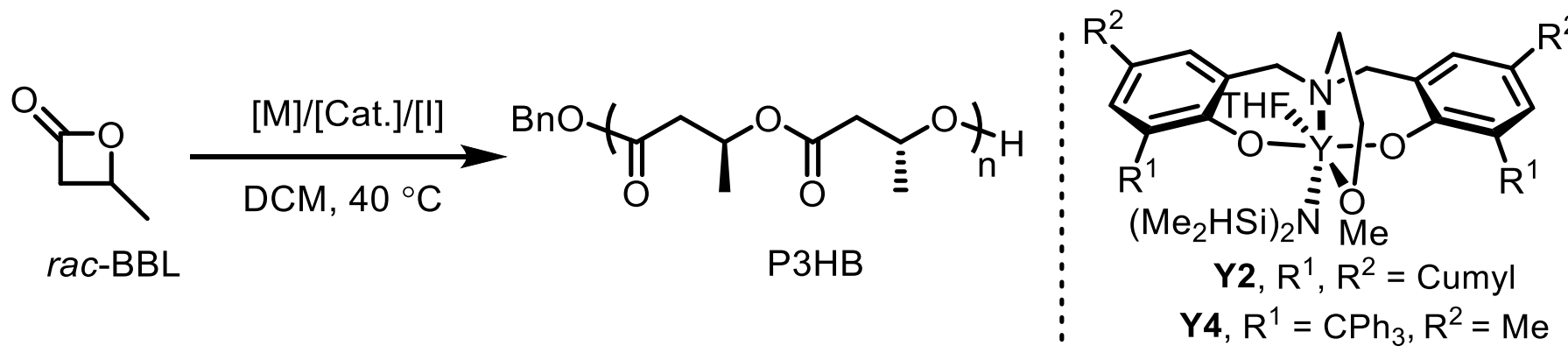

Runs	[*rac*BBL]/[Cat.]/[I]	[Cat.]	Conv.[%]	Sol.	M_n[b] [kDa]	*Đ*[b]	P_r[c]
1[d]	1000:1:1	**Y2**	96	DCM (5M)	56.0	1.06	0.84
2[d]	2000:1:1	**Y2**	98	DCM (3.5M)	127	1.09	0.84
3[f]	200:1:1	**Y4**	>99	DCM (2M)	11.3	1.20	0.91

[a] Monomer conversion (Conv.) determined by ^{1}H NMR in $CDCl_3$, initiator (I) = $PhCH_2OH$, 12 h, 40 °C. [b] Weight-average (M_w) and number-average (M_n) molar mass and dispersity ($Đ = M_w/M_n$) determined via size exclusion chromatography (SEC) at 40 °C in $CHCl_3$ coupled with a Wyatt DAWN HELEOS II multi (18)-angle light scattering detector and a Wyatt Optilab TrEX dRI detector for absolute molar mass. [c] P_r is the probability of *racemic* linkages between *rac*-BBL units [d] [*rac*-BBL] (860 mg, 10 mmol), 24 h. [e] [*rac*-BBL] (86.0 mg,1.0 mmol).

Table S11. Gel Fraction analysis of crosslinked P(BL^{me}_{96}-*co*-BL^{allyl}_{4})-PETMP[a]

Solvent	DCM	Acetone	EtOAc
Gel Fraction (%)	75.0	79.8	89.9

[a] For the given sample, a piece of crosslinked sample (60−70 mg) was put in a nylon teabag and swollen in 20 mL of anhydrous solvent (DCM: 50 °C; Acetone and EtOAc: 80 °C) for 48 h. The swollen gel was then isolated and dried under reduced pressure and mild heating (60 °C) until the weight became constant. Gel fraction was calculated as a percentage of the final mass of the dried material divided by its initial mass.

Table S12. Tensile stress-strain data of P(BL^{vinyl}) (M_n = 42.6 kDa, *Đ* = 1.02) dog-bone-shaped specimens

Entry	Young's modulus (MPa)	elongation at break (%)	ultimate tensile strength (MPa)	toughness (MJ m^{-3})
1	237	10.6	12.3	0.9
2	238	7.9	9.7	0.5
3	228	13.3	10.7	1.1
Average	234	10.6	10.9	0.8
Standard deviation	5.5	2.8	1.3	0.3

Table S13. Tensile stress-strain data of P(BL$^{allyl)}$ (M_n = 245 kDa, $Đ$ = 1.23) dog-bone-shaped specimens

Entry	Young's modulus (MPa)	elongation at break (%)	yield stress (MPa)	ultimate tensile strength (MPa)	toughness (MJ m^{-3})
1	267	271.5	10.7	33.9	52.5
2	258	230.4	11.0	32.6	42.6
3	254	230.7	10.9	31.7	42.0
Average	260	244.2	10.9	32.7	45.7
Standard deviation	6.7	23.6	0.2	1.1	5.9

Table S14. Tensile stress-strain data of P(BL$^{hPg)}$ (M_n = 72.9 kDa, $Đ$ = 1.03) dog-bone-shaped specimens

Entry	Young's modulus (MPa)	elongation at break (%)	yield stress (MPa)	ultimate tensile strength (MPa)	toughness (MJ m^{-3})
1	297	362.8	9.6	21.0	43.1
2	279	367.3	10.2	19.8	42.9
3	303	307.1	10.2	17.2	32.4
Average	293	345.7	10.0	19.3	38.9
Standard deviation	12.5	33.5	0.3	1.9	5.7

Table S15. Tensile stress-strain data of P(BL$^{Me}{}_{95}$-*co*-BL$^{vinyl}{}_{5}$) (M_n = 169 kDa, $Đ$ = 1.08) dog-bone-shaped specimens

Entry	Young's modulus (MPa)	elongation at break (%)	yield stress (MPa)	ultimate tensile strength (MPa)	toughness (MJ m^{-3})
1	609	195.3	22.2	30.6	48.5
2	640	198.7	21.9	31.6	50.6
3	649	249.5	21.3	33.8	64.7

Average	633	214.5	21.8	32.0	54.6
Standard deviation	21.0	30.3	0.5	1.6	8.8

Table S16. Tensile stress-strain data of P(BL^{Me}_{90}-*co*-BL^{vinyl}_{10}) (M_n = 159 kDa, $Đ$ = 1.05) dog-bone-shaped specimens

Entry	Young's modulus (MPa)	elongation at break (%)	yield stress (MPa)	ultimate tensile strength (MPa)	toughness (MJ m^{-3})
1	114	285.6	15.1	30.4	63.0
2	102	318.7	14.4	32.0	71.1
3	110	295.8	14.6	29.1	62.4
Average	109	300.0	14.7	30.5	65.5
Standard deviation	6.1	17.0	0.4	1.5	4.9

Table S17. Tensile stress-strain data of P(BL^{Me}_{96}-*co*-BL^{allyl}_{4}) (M_n = 161 kDa, $Đ$ = 1.13) dog-bone-shaped specimens

Entry	Young's modulus (MPa)	elongation at break (%)	yield stress (MPa)	ultimate tensile strength (MPa)	toughness (MJ m^{-3})
1	506	222.1	19.3	28.5	51.5
2	532	239.7	20.9	33.0	61.4
3	538	159.8	20.7	27.8	36.6
Average	525	207.2	20.3	29.8	49.8
Standard deviation	17.0	42.0	0.9	2.8	12.5

Table S18. Tensile stress-strain data of P(BL^{Me}$_{92}$-*co*-BL^{allyl}$_{8}$) (M_n = 202 kDa, $Đ$ = 1.05) dog-bone-shaped specimens

Entry	Young's modulus (MPa)	elongation at break (%)	yield stress (MPa)	ultimate tensile strength (MPa)	toughness (MJ m^{-3})
1	216	356.6	12.8	32.9	79.3
2	285	384.6	13.9	34.5	90.9
3	272	349.6	12.9	32.8	77.1
Average	257	363.6	13.2	33.4	82.4
Standard deviation	36.7	18.5	0.6	0.95	7.4

Table S19. Tensile stress-strain data of P(BL^{Me}$_{92}$-*co*-BL^{allyl}$_{8}$) prepared with catalyst **Y4** (M_n = 121 kDa, $Đ$ = 1.01) dog-bone-shaped specimens

Entry	Young's modulus (MPa)	elongation at break (%)	ultimate tensile strength (MPa)	toughness (MJ m^{-3})
1	823	2.6	14.9	0.22
2	801	2.4	14.5	0.20
3	797	2.3	14.0	0.19
Average	807	2.4	14.5	0.20
Standard deviation	14.0	0.2	0.5	0.02

Table S20. Tensile stress-strain data of P(BL^{Me}$_{95}$-*co*-BL^{hPg}$_{5}$) (M_n = 90.5 kDa, $Đ$ = 1.08) dog-bone-shaped specimens

Entry	Young's modulus (MPa)	elongation at break (%)	yield stress (MPa)	ultimate tensile strength (MPa)	toughness (MJ m^{-3})
1	533	274.2	19.4	32.8	68.3
2	513	272.1	19.7	34.7	71.1
3	486	213.6	18.6	30.6	51.0

Average	511	253.3	19.2	32.7	63.5
Standard deviation	23.6	34.4	0.6	2.1	10.9

Table S21. Tensile stress-strain data of P(BL$^{Me}_{90}$-*co*-BL$^{hPg}_{10}$) (M_n = 79.8 kDa, $Đ$ = 1.07) dog-bone-shaped specimens

Entry	Young's modulus (MPa)	elongation at break (%)	yield stress (MPa)	ultimate tensile strength (MPa)	toughness (MJ m^{-3})
1	533	387.3	11.7	31.9	80.3
2	513	336.2	12.3	31.4	68.7
3	486	369.6	12.5	30.8	76.5
Average	511	364.4	12.2	31.4	75.2
Standard deviation	23.6	25.9	0.4	0.6	5.9

Table S22. Tensile stress-strain data of P(BL^{vinyl})×2% dog-bone-shaped specimens

Entry	Young's modulus (MPa)	elongation at break (%)	yield stress (MPa)	ultimate tensile strength (MPa)	toughness (MJ m^{-3})
1	248	102.7	9.8	12.7	10.6
2	248	61.9	9.8	10.7	5.9
3	260	81.1	10.0	11.3	7.9
Average	255	81.9	9.9	11.6	8.1
Standard deviation	6.4	20.4	0.1	1.0	2.4

Table S23. Tensile stress-strain data of P(BL^{vinyl})×5% dog-bone-shaped specimens

Entry	Young's modulus (MPa)	elongation at break (%)	yield stress (MPa)	ultimate tensile strength (MPa)	toughness (MJ m^{-3})
1	99	122.3	5.3	9.6	8.3
2	92	137.3	5.7	12.2	11.3

3	98	138.8	5.2	10.5	10.1
Average	96	132.8	5.4	10.7	9.9
Standard deviation	3.9	9.1	0.3	1.3	1.5

Table S24. Tensile stress-strain data of P(BL^{allyl})×2% dog-bone-shaped specimens

Entry	Young's modulus (MPa)	elongation at break (%)	yield stress (MPa)	ultimate tensile strength (MPa)	toughness (MJ m^{-3})
1	130	175.1	7.2	22.0	23.4
2	161	195.4	8.5	24.1	28.6
3	142	224.2	6.6	23.0	28.9
Average	144	198.2	7.4	23.0	27.0
Standard deviation	15.6	24.7	1.0	1.1	3.1

Table S25. Tensile stress-strain data of P(BL^{allyl})×5% dog-bone-shaped specimens

Entry	Young's modulus (MPa)	elongation at break (%)	yield stress (MPa)	ultimate tensile strength (MPa)	toughness (MJ m^{-3})
1	86	97.4	4.5	8.6	5.8
2	74	123.6	4.1	11.0	8.2
3	89	103.4	4.3	8.9	6.1
Average	83	108.1	4.3	9.5	6.7
Standard deviation	7.9	13.7	0.2	1.3	1.3

Table S26. Tensile stress-strain data of cross-linked P(BL$^{me}{}_{96}$-*co*-BL$^{vinyl}{}_{4}$)-PETMP dog-bone-shaped specimens

Entry	Young's modulus (MPa)	elongation at break (%)	yield stress (MPa)	ultimate tensile strength (MPa)	toughness (MJ m^{-3})
1	363	125.5	16.9	26.2	25.3
2	367	176.4	17.5	31.4	40.7

3	312	194.4	16.7	35.6	48.9
Average	347	165.4	17.0	31.1	38.3
Standard deviation	30.7	35.7	0.4	4.7	12.0

Table S27. Tensile stress-strain data of cross-linked P(BL^{me}_{96}-*co*-BL^{allyl}_{4})-PETMP dog-bone-shaped specimens

Entry	Young's modulus (MPa)	elongation at break (%)	yield stress (MPa)	ultimate tensile strength (MPa)	toughness (MJ m^{-3})
1	434	150.9	16.4	24.7	28.7
2	434	186.7	17.0	28.6	40.1
3	451	122.0	17.5	25.3	24.6
Average	440	153.2	17.0	26.2	24.9
Standard deviation	9.5	32.4	0.6	2.1	13.7

Table S28. Tensile stress-strain data of P(BL^{me}_{90}-*co*-BL^{allyl}_{10}) (M_n = 170 kDa, $Đ$ = 1.12) dog-bone-shaped specimens

Entry	Young's modulus (MPa)	elongation at break (%)	yield stress (MPa)	ultimate tensile strength (MPa)	toughness (MJ m^{-3})
1	120	336.3	2.5	3.2	3.2
2	110	440.3	3.0	4.0	3.9
3	112	317.7	3.0	3.5	3.0
Average	114	364.8	2.8	3.6	3.3
Standard deviation	5.19	66.1	0.3	0.4	0.5

Table S29. Tensile stress-strain data of P(BL^{me}_{90}-*co*-BL^{allyl}_{10})-UPy dog-bone-shaped specimens

Entry	Young's modulus (MPa)	elongation at break (%)	yield stress (MPa)	ultimate tensile strength (MPa)	toughness (MJ m^{-3})
1	315	368.9	9.5	16.7	34.2
2	354	437.2	11.4	18.2	33.6
3	165	152.5	17.0	9.1	12.7
Average	284.3	319.5	12.9	14.7	26.8
Standard deviation	103.8	148.6	3.6	4.9	12.2

References

[1] M.-H. Lin, T. V. RajanBabu, *Org. Lett.* **2002**, *4*, 1607–1610.

[2] A. Amgoune, C. M. Thomas, T. Roisnel, J.-F. Carpentier, *Chem. Eur. J.* **2006**, *12*, 169–179.

[3] M. Bouyahyi, N. Ajellal, E. Kirillov, C. M. Thomas, J.-F. Carpentier, *Chem. Eur. J.* **2011**, *17*, 1872–1883.

[4] J. Zhu, E. Y.-X. Chen, *Angew. Chem. Int. Ed.* **2019**, *58*, 1178–1182.

[5] M. North, S. C. Z. Quek, N. E. Pridmore, A. C. Whitwood, X. Wu, *ACS Catal.* **2015**, *5*, 3398–3402.

[6] R. M. Cywar, C. Ling, R. W. Clarke, D. H. Kim, C. M. Kneucker, D. Salvachúa, B. Addison, S. A. Hesse, C. J. Takacs, S. Xu, M. U. Demirtas, S. P. Woodworth, N. A. Rorrer, C. W. Johnson, C. J. Tassone, R. D. Allen, E. Y.-X. Chen, G. T. Beckham, *Sci. Adv.* **2023**, *9*, eadi1735.

[7] A. Kremsmair, H. R. Wilke, J. H. Harenberg, B. R. G. Bissinger, M. M. Simon, N. Alandini, P. Knochel, *Angew. Chem. Int. Ed.* **2023**, 62, e202214377.

[8] P. Gupta, P. Kumar, *Tetrahedron: Asymmetry* **2007**, *18*, 1688–1692.

[9] Y.-T. Li, H.-Y. Yu, W.-B. Li, Y. Liu, X.-B. Lu, *Macromolecules* **2021**, *54*, 4641–4648.

[10] A. Patel, G. A. Barcan, O. Kwon, K. N. Houk, *J. Am. Chem. Soc.* **2013**, *135*, 4878–4883.
